# Clinical Pharmacokinetics of Fexofenadine: A Systematic Review

**DOI:** 10.3390/pharmaceutics16121619

**Published:** 2024-12-20

**Authors:** Maryam Batool, Ammara Zamir, Faleh Alqahtani, Tanveer Ahmad, Hamid Saeed, Muhammad Fawad Rasool

**Affiliations:** 1 Department of Pharmacy Practice, Faculty of Pharmacy, Bahauddin Zakariya University, Multan 60800, Pakistan; maryambatool1818@gmail.com (M.B.); ammarazamir20@gmail.com (A.Z.); 2 Department of Pharmacology and Toxicology, College of Pharmacy, King Saud University, Riyadh 11451, Saudi Arabia; 3 Institute for Advanced Biosciences (IAB), CNRS UMR5309, INSERM U1209, Grenoble Alpes University, 38700 La Tronche, France; 4 Section of Pharmaceutics, University College of Pharmacy, Allama Iqbal Campus, University of the Punjab, Lahore 54000, Pakistan; hamid.pharmacy@pu.edu.pk

**Keywords:** fexofenadine, second generation, pharmacokinetics, humans, systematic review, clearance, AUC, allergic rhinitis

## Abstract

**Background/Objectives:** Fexofenadine hydrochloride is a widely prescribed drug for treating histamine-mediated allergic reactions. This review systematically collates existing research on the clinical pharmacokinetics (PK) of fexofenadine, with a copious emphasis on examining the impact of stereoisomerism, disease states, and drug interactions. **Methods:** The search engines PubMed, Science Direct, Google Scholar, and Cochrane were scanned systematically for articles concerning the clinical PK of fexofenadine in humans. The extensive literature search yielded 85 articles meeting the inclusion standards. **Results:** The PK parameters of fexofenadine showed a linear correlation between increasing doses and proportional elevations in PK parameters such as area under the curve from time 0 to infinity (AUC_0–∞_) and maximum plasma concentration (C_max_). Under fed conditions, its bioavailability was reduced by approximately 50%. Findings from patients with end-stage renal disease (ESRD) displayed a 63% decline in oral clearance (CL/F) of fexofenadine. A drug–food interaction study has displayed that grapefruit juice decreased C_max_ (201 ng/mL vs. 128 ng/mL), accompanied by a 30% reduction in the bioavailability of fexofenadine. Furthermore, a drug–herb interaction study with St John’s Wort (SJW) has reported a reduction in CL/F by 10% after a single dose, but long-term administration reversed this effect, resulting in elevated CL/F by 17% of fexofenadine. **Conclusions:** Since no prior systematic review on the PK of this drug exists, this review amalgamates all pertinent PK parameters in humans by pooling up-to-date data from published studies. This detailed literature review can be advantageous for researchers who want to develop and assess PK models.

## 1. Introduction

Fexofenadine hydrochloride is a second-generation, peripheral histamine H_1_- receptor antagonist drug [1,2]. It was developed by Hoechst Marion Roussel and granted a patent approval in 1979 [3,4]. The drug was originally synthesized in 1993 by the Massachusetts-based biotechnology company Sepracor [5] and approved by the US Food and Drug Administration (FDA) on 30 July 1996 [6] for the relief of chronic idiopathic urticaria and seasonal allergic rhinitis [7]. Fexofenadine is commonly considered an ‘inverse agonist’ because it stabilizes the inactive configuration of the H_1_ receptor, which in turn helps to alleviate the histamine-induced allergic responses. It is a non-sedating drug because of its negligible impact on cognitive performance [8]. In patients with chronic urticaria, it appeared to reduce the levels of tryptase, vascular cell adhesion molecule-1 (VCAM-1), and endothelial leukocyte adhesion molecule-1 (ELAM-1) suggesting anti-inflammatory benefits through cytokine regulation [9]. This medication can be used off-label to treat allergic conjunctivitis, angioedema, eczema, and reactions to insect bites and stings [10]. The drug is available as tablets, suspension, and orally disintegrating tablets and is administered at a dose of 60 mg, 120 mg, and 180 mg [11].

Fexofenadine is a carboxylate metabolite of terfenadine that had been backed off from the US market due to potential cardiotoxic effects, including ventricular dysrhythmias and QTc interval prolongation [10]. Unlike terfenadine, fexofenadine is a safer drug and does not cause detrimental cardiac effects even when used in conjunction with cytochrome P450 (CYP3A) inhibitors such as erythromycin or ketoconazole [11].

Fexofenadine is categorized as a Biopharmaceutics Classification System (BCS) class III drug with low permeability and high solubility [12]. It has a low bioavailability of 33% [13] and is quickly absorbed after oral absorption, achieving peak plasma concentration (C_max_) in approximately 1–3 h [14]. The volume of distribution (V_d_) of the drug is reported to be 5.4–5.8 L/kg [15]. It predominantly binds to albumin and α_1_-acid glycoprotein, constituting 60% to 70% of plasma protein binding [16]. It is a racemic blend of equipotent (R)- and (S)- enantiomers and has a renal clearance (CL_R_) of 4.32 L/h [12,16]. About 5% of the drug is metabolized by intestinal mucosa [17], and the remaining 92% of the administered drug is excreted unchanged in urine (12%) and feces (80%) [18]. Fexofenadine serves as a substrate for various transporters like P-glycoprotein (P-gp), organic anion-transporting polypeptides (OATPs), and multi-drug resistance proteins (MRP2). Due to negligible metabolism by CYP450, these transporters play a requisite role in the disposition of the drug [19].

Fexofenadine hydrochloride has an empirical formula C_32_H_39_NO_4_. HCl, with a molecular weight of 538.1 g/mol [20]. It is a hydrophilic zwitterion having a Log-*p* value of 0.49 and water solubility of approximately 0.81 mg/mL [21]. The effective jejunal permeability (P_eff_) of the drug is 0.06 ± 0.07 × 10^−4^ cm/s [22]. Moreover, the octanol-to-water partition coefficient is reported to be 2.81 [23].

Fexofenadine belongs to pregnancy category C, and it does not cross the blood-brain barrier [17,24]. The adverse effects linked to the drug include back pain, dyspepsia, dysmenorrhea, dizziness, nausea, fatigue, drowsiness, dry mouth, and the most commonly stated effect is headache. The drug does not cause any cardiotoxicity and entails minimal hepatotoxicity [17,25,26]. Rare side effects of Stevens-Johnson syndrome (SJS) and toxic epidermal necrolysis (TEN) have been reported in the post-marketing surveillance [27]. It is contraindicated in patients with a known hypersensitivity to fexofenadine or its ingredients [17], and caution is recommended in phenylketonuria patients [11].

The rationale of this research is to systematically assess all the pertinent pharmacokinetic (PK) parameters of fexofenadine in humans by pooling up-to-date data from published studies so that unexplored gaps involving the clinical utilization of the drug can be addressed. Different review articles have been published shedding light on fexofenadine usage, which focused on safety and efficacy in children [28], management of allergic disorders [9,29], and pharmacokinetic, pharmacodynamic, and biochemical properties [30]. Two systematic reviews have been reported on the antihistamine effects and safety of fexofenadine, and one on the efficacy of fexofenadine [26,31]. Although extensive literature is available regarding the PK of the drug, there is no report of a single systematic review that integrates all the factors influencing its clinical PK, such as genetic variability, transporters, stereoisomerism, co-existing disease states, physiological changes in special populations and interactions with other drugs, food, and herbs. The objective of this review is to critically evaluate all the PK aspects of fexofenadine up to the present and execute a unified overview exploring variations in drug PK exposure among different populations and anticipating potential drug interactions alongside the influence of transporters, stereoisomerism, and genetic variations, which may assist clinicians in dose optimization and clinical decision making. Additionally, this may also aid researchers in developing and evaluating PK models.

## 2. Methodology

### 2.1. Study Design

This systematic review was meticulously executed in alignment with the Cochrane Handbook guidelines [32] and Preferred Reporting Items for Systematic Reviews and Meta-Analyses (PRISMA) guidelines [33] and has been registered in PROSPERO with the registration number “CRD42024530096”. The PRISMA Checklist is explicated in Supplementary Table S1.

### 2.2. Search Strategy

**Figure 1:**
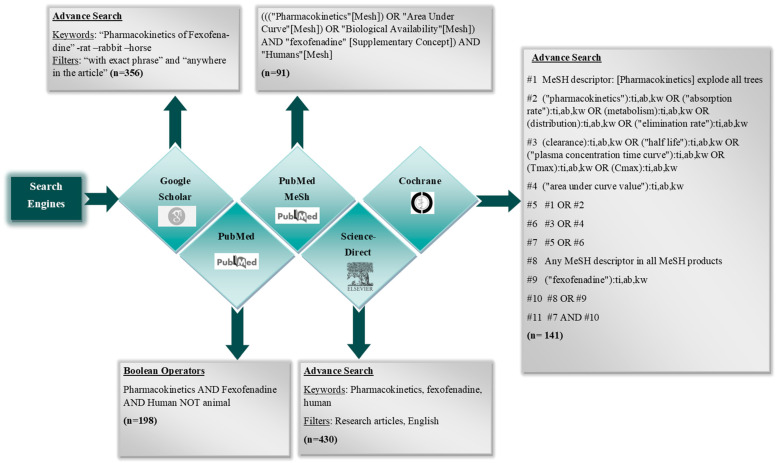
Literature Search Strategy.

The online search engines (PubMed, Google Scholar, ScienceDirect, Cochrane Library) were scrutinized comprehensively to ensure a thorough retrieval of all published studies detailing the PK of fexofenadine in humans. The search strategy was initiated by selecting the keywords based on Medical subject headings (MeSH), Boolean operators, and additional keywords from published articles. The searches were carried out from 13 September 2023 to 9 October 2023. The search methodologies are depicted in Figure 1.

### 2.3. Eligibility Criteria

The inclusion criteria dictate that an original research article should present at least one PK parameter of fexofenadine. No restrictions were imposed on the publication year, and only articles written in English were deemed eligible.

### 2.4. Data Extraction and Study Selection

After conducting comprehensive searches through different search engines, all retrieved articles were imported into EndNote version 20, and duplicates were eradicated using the “remove duplicate” option. Then screening was executed based on titles, abstracts, animal studies, and non-accessibility. Letters to the editor, investigative reports, and short reviews were also omitted from the analysis. Articles that met the eligibility criteria underwent additional screening through full-text reading, resulting in the selection of the pertinent studies. The process of data extraction and eligibility evaluation was further finalized by two independent reviewers. The excluded articles are documented in Supplementary Table S2. The PK parameters to be assessed included area under the curve from time 0 to infinity (AUC_0-∞_), maximum plasma concentration (C_max_), time to reach maximum plasma concentration (T_max_), half-life (t_1/2_), and clearance (CL). The standardization of PK parameter units was carried out to facilitate consistency in data comparison.

### 2.5. Quality Assessment

The quality assessment of all relevant studies was conducted using Jadad scoring, the Critical Appraisal Skills Programme (CASP) scoring system, and the Critical Appraisal Clinical Pharmacokinetic Tool (CACPK). Jadad scoring assigns a score from 0 to 5 based on the reporting of randomization, blinding, and drop-outs in the relevant article [34]. Studies having scores < 3, 3–4, and >4 are categorized as low, moderate, and high quality, respectively. CASP scoring, based on a 10-point scale, with a score of >6 signifies high quality, 4–6 implies moderate quality, and <4 shows low quality [35]. The CACPK, a 21-question checklist, categorizes articles into low, high, or moderate quality based on their score < 12, >13, or 12–13, respectively [36]. The Cochrane Collaboration tool was apprenticed to assess the risk of bias, and studies were categorized based on their scores, which indicated either a high, moderate, or low risk of bias. Scores falling < 3 indicated a high risk, 3–4 suggested a moderate risk, and >4 signified a low risk of bias [37]. The detailed scoring is documented in Supplementary Tables S3–S6.

## 3. Results

### 3.1. Literature Search Results

**Figure 2:**
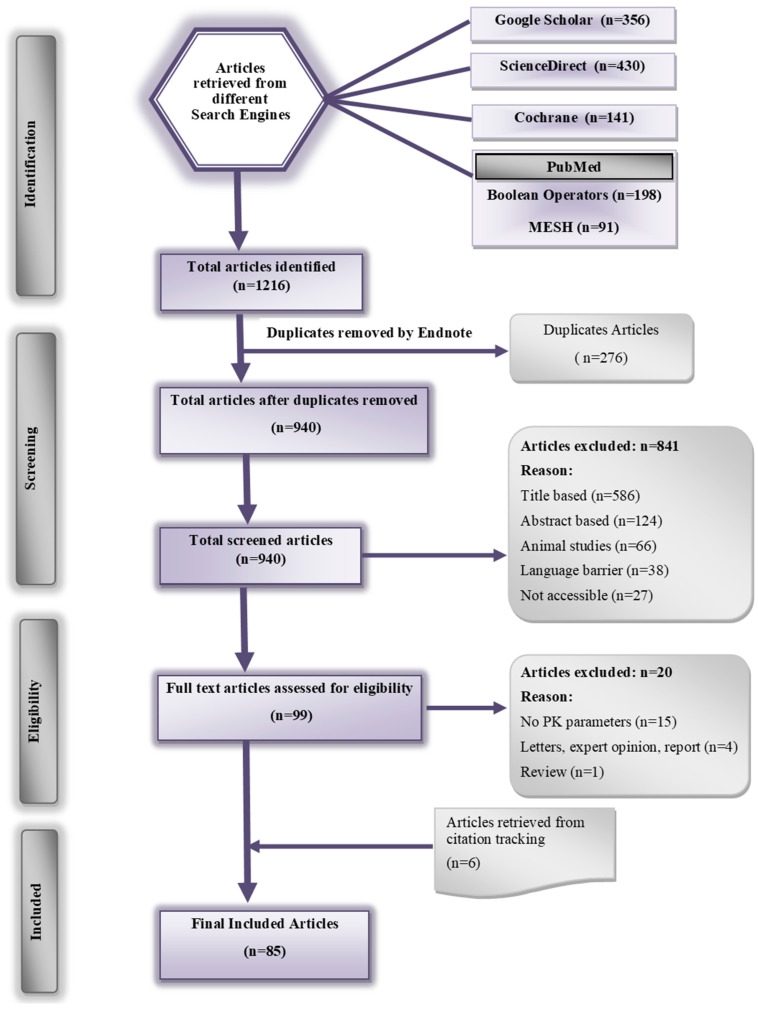
PRISMA Flow Diagram.

By utilizing different search engines, 1216 articles were identified, of which 276 were duplicates. The remaining 940 articles were subjected to further screening by applying both inclusion and exclusion criteria, resulting in a final inclusion of 85 articles. The PRISMA flow diagram displaying the final count of studies is illustrated in Figure 2.

**Figure 3:**
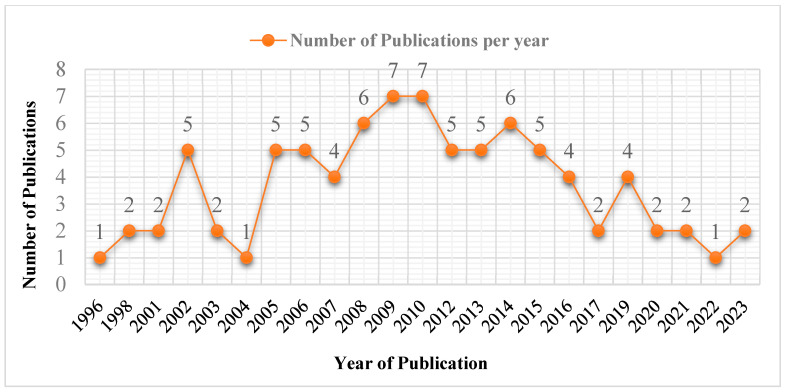
Number of articles published per year.

The first article discussing the PK of fexofenadine was published in 1996 [38], and then the number of related publications increased gradually throughout the ensuing years. The included articles are further arranged by the year of publication in Figure 3.

### 3.2. Quality of Included Studies

A total of 85 articles were scrutinized for quality assessment using Jadad scoring, CASP scoring, and the CACPK tool. Jadad scoring categorized 72 articles as low quality and 13 as moderate quality. CASP scoring revealed that all articles are of high quality. In CACPK scoring, high, moderate, and poor quality were indicated by 60, 21, and 4 studies, respectively. According to Cochrane Collaboration, 21, 40, and 24 articles have a high, moderate, or low risk of bias, respectively.

### 3.3. Study Characteristics

The demographic characteristics of studies are delineated in Table 1, incorporating various aspects such as population, age, drug, dose, frequency, dosage form, and analytical method.

### 3.4. Studies Including Healthy Population

#### 3.4.1. Intravenous Administration

A single study was carried out after intravenous (IV) administration of fexofenadine. The study aimed to investigate the disposition kinetics between two 100 μg intravenous non-radiolabeled fexofenadine doses. One IV microdose dose was administered alone, while the other was given alongside a 120 mg oral therapeutic dose. Both doses showed closely similar CL values of 13 L/h and 16 L/h, respectively [21].

#### 3.4.2. Oral Administration

A total of 22 oral studies were conducted with a healthy population. The PK of fexofenadine displayed linearity within the therapeutic dose range of 20–120 mg but elicited a minor disproportional increase in AUC_0–∞_ by 9% and C_max_ by 25% at 240 mg dose [40]. A study has reported that C_max_ varied between 45.6 ng/mL and 6383 ng/mL following a single dose range of 10–800 mg and between 57.9 ng/mL and 4677 ng/mL with multiple doses ranging from 20–690 mg [39]. In a clinical study, phenylalanine-free taste-masked orodispersible tablet (ODT) of fexofenadine demonstrated significantly higher AUC_0-∞_ of 1856.098 ± 692.314 ng.h/mL as compared to immediate release (IR) tablet [56]. One of the studies compared the PK of a therapeutic dose (120 mg) and microdose (100 μg), depicting a C_max_ of 318 ng/mL in the former and 0.31 ng/mL in the latter dose [21]. In another clinical study, the PK of fexofenadine/pseudoephedrine combination tablet was evaluated in the fasted and fed states which indicated a 1.2-fold decrease in drug exposure in the latter [48].

Moreover, a study has narrated that the (R)-enantiomer displayed considerably greater AUC_0–∞_ as compared to the (S)-enantiomer—i.e., 843 ± 153 ng.h/mL vs. 496 ± 131 ng.h/mL, respectively [46].

#### 3.4.3. Effect of Genes Encoding Drug Transporters

Two studies have explored the influence of MDR1 gene polymorphism on fexofenadine disposition by analyzing variant alleles for the G2677T polymorphism in exon 21 and the C3435T polymorphism in exon 26 after an oral dose of 180 mg [41,43]. A study preceded the activity of variant A allele based on genotype combinations of single nucleotide polymorphism (SNPs) revealed that C_max_ values were 494 ± 81 ng.h/mL for 2677AA/3435CC and 958 ± 408 ng.h/mL for 2677TT/3435TT [43]. Upon stratification by the C3435T, individuals homozygous for the TT genotype showed elevated AUC**_0–∞_** values of 3910.1 ± 1894.8 ng.h/mL in comparison to the CC genotype, which displayed values of 3567.1 ± 1535.5 ng.h/mL [41]. Moreover, a study involving an in vivo cocktail approach to investigate drug transporter P-gp and CYP isoforms activity revealed varied CL/F of 205.87 L/h, 293.56 L/h, and 106.21 L/h for EM (all CYPs extensive metabolizer), CYP2C9 *3/*3, and CYP2D6 *4/*4 genotypes, respectively [60].

Furthermore, a clinical study investigating the association of drug-transporter polymorphisms has reported that (S)-enantiomer carrying the SLCO2B1*1/*1 allele exhibited significantly reduced AUC_0–24_ relative to the SLCO2B1*1/*3 + *3/*3 allele, i.e., 446 ng.h/mL vs. 675 ng.h/mL, respectively [51].

The PK parameters in healthy populations, segregated by the different groups, are depicted in Table 2.

### 3.5. Studies with Diseased Population

The PK of fexofenadine remained consistent between cystic fibrosis patients and age-mates healthy participants when it was administered alone, but probenecid coadministration significantly augmented its C_max_ to 470–1210 ng/mL [18]. One study was conducted on patients with advanced non–small cell lung cancer (NSCLC) that exhibited a surge in C_max_ by 76% and 25% following single and multiple dose administration, respectively [59]. Moreover, a study executed on patients suffering from end-stage renal disease (ESRD) has displayed a significant decline in CL/F from 102.8 ± 37.9 L/h to 37.9 ± 19.5 L/h [50]. Furthermore, another study has reported no significant alterations in fexofenadine PK between the “before hemodialysis” and “after hemodialysis” investigations depicting that hemodialysis did not alter the AUC_0–∞_, i.e., 2355 ng.h/mL vs. 2785 ng.h/mL [62].

The remaining PK parameters of diseased populations are summarized in Table 3.

### 3.6. Studies with Special Population

A clinical study revealed that children suffering from allergic rhinitis aged 2–5 years had a C_max_ value of 224 ng/mL following a 30 mg dose of oral suspension [49]. Another study entailed the enantioselective disposition of fexofenadine in parturient women and displayed AUC_0-∞_ values of 267.67 ng.h/mL for (S)-fexofenadine and 423.2 ng.h/mL for (R)-fexofenadine [61].

The related PK parameters of special populations are explicated in Table 4. 

### 3.7. Drug–Drug Interactions (DDI) of Fexofenadine

A higher potential for drug interactions is associated with increased polytherapy. In 1999, a report indicated that drug transporters like OATP and P-gp potentially mediate drug interactions of antihistamines, including fexofenadine [66]. The mechanism underpinning drug interactions involves the induction or inhibition of drug transporters that facilitate the cellular efflux and uptake of the xenobiotics. The majority of drug–drug interactions (DDI) of fexofenadine were conducted using the probe substrate-based cocktail approach, which involves concurrent administration of subtherapeutic doses of probe substrates to evaluate the P-gp transport activity [60].

A total of 31 studies analyzed the interaction between fexofenadine and various drugs. A study was undertaken to assess the collective effect of fexofenadine and azalide antibiotic azithromycin, indicating an elevation in mean C_max_ from 199 ng/mL to 349 ng/mL [66]. One of the studies looked into the impact of transporting inhibitors on fexofenadine and displayed a 1.5-fold increase in its AUC_0–∞_ with probenecid treatment and a 2.9-fold increase in its C_max_ with verapamil treatment. Additionally, cimetidine’s inhibitory effect led to a decrease in CL_R_ of fexofenadine from 13.8 ± 4.68 L/h to 9.12 ± 4.2 L/h without any discernible changes in other PK parameters [74].

Another study has highlighted a substantial drop in fexofenadine C_max_ from 133 ± 67 ng/mL to 100 ± 43 ng/mL on simultaneous administration with nelfinavir [85]. A study analyzing the in vivo effects of selective serotonin reuptake inhibitors (SSRIs) on fexofenadine has demonstrated that co-administration with fluvoxamine led to a notable increase in its C_max_ by 57% and an elevation in its AUC_0–∞_ by 38% was observed with paroxetine [93]. One of the studies has depicted that both acute and steady-state ritonavir caused a time and dose-dependent spike in fexofenadine AUC_0–∞_ by 2.8-fold and 1.4-fold, respectively [83].

Furthermore, a clinical study has reported that the combined impact of Vitamin D3 with fexofenadine showed a significant elevation in fexofenadine’s C_max_ from 184.5 nmol/L to 317.5 nmol/L [106]. No discernible changes in the PK of fexofenadine were depicted with diltiazem [78], sertraline [93], breviscapine [113], and metronidazole [89].

#### 3.7.1. Effect of Enantiomers on DDI of Fexofenadine

A clinical study has reported that concomitant use of carbamazepine induced a substantial decrease in C_max_ from 100 ng/mL to 68 ng/mL for (S)-fexofenadine and from 132 ng/mL to 85 ng/mL for (R)-fexofenadine [16]. One of the clinical studies has narrated the cumulative effect of fexofenadine with a single dose of rifampicin and displayed a reduction in CL/F of both (S)- and (R)-enantiomer by 77% and 73%, respectively [19]. Another study has delineated that multiple doses of rifampicin markedly elevated the AUC_0–24_ of (R)- and (S)-fexofenadine by 3.1-fold and 3.48-fold, respectively [104].

#### 3.7.2. Effect of Genotypes on DDI of Fexofenadine

An oral study involving MDR1 genetic polymorphism has exhibited a significant escalation in C_max_ values from 713.8 ± 311.4 ng/mL to 2136.2 ± 897.9 ng/mL for 2677TT/3435TT genotype and from 510.8 ± 262.6 ng/mL to 1376.3 ± 340.5 ng/mL for 2677GG/3435CC genotype when itraconazole was co-administered with fexofenadine [72]. Another study has concluded that the breviscapine effect on fexofenadine PK was independent of MDR1 C3435T genetic polymorphism [113].

All the remaining PK parameters of DDI are listed in Table 5.

### 3.8. Drug–Food Interactions (DFI) of Fexofenadine

#### 3.8.1. Interactions of Fexofenadine with Fruit Juices

A clinical study has investigated the outcomes concerning the administration of 60 mg fexofenadine with grapefruit juice (GFJ) and showed a decrease in C_max_ from 201 ng/mL to 128 ng/mL accompanied by a 30% reduction in the bioavailability of fexofenadine [69]. Another study has reported a volume-dependent decline in AUC_0–∞_ by 53% and 33% after consuming 300 mL and 1200 mL of GFJ, respectively [75]. An interaction study focusing on dietary constituents of GFJ has reported different C_max_ values of 269 ng/mL, 380 ng/mL, and 463 ng/mL when fexofenadine was consumed with GFJ, naringin solution, and water, respectively [81].

One of the studies has highlighted that fexofenadine showed a decrease in AUC**_0-∞_**, i.e., 1598 ± 496 ng.h/mL, 1072 ± 429 ng.h/mL, and 668 ± 163 ng.h/mL with increasing apple juice (AJ) volume of 150 mL, 300 mL, and 600 mL [108].

A clinical study has mentioned the combined effect of different fruit juices with fexofenadine and displayed varied C_max_ values of 288 ± 24 ng/mL, 110 ± 14 ng/mL, 96 ± 7 ng/mL, and 81 ± 13 ng/mL for water, GFJ, orange juice, and AJ, respectively. However, CL_R_, T_max_, and t_1⁄2_ remain unchanged across all treatments [70].

#### 3.8.2. Interactions of Fexofenadine with Green Tea Extract (GTE)

The effect of GTE containing (−)-epigallocatechin gallate on the fexofenadine PK has demonstrated a lower C_max_ value compared to water—i.e., 82.6 ng/mL vs. 278.7 ng/mL. No disparities were observed in the CL_R_, T_max_, and t_1/2_ between the water and GTE phases [114].

#### 3.8.3. Effect of Enantiomers on DFI of Fexofenadine

A clinical study has depicted a reduction in AUC_0–24_ of both (R)- and (S)-enantiomers by 59% and 49%, respectively, after a single intake of AJ [101]. Another study has reported that co-administration with GFJ resulted in increased CL/F of both (R)-fexofenadine—i.e., 41 L/h vs. 75 L/h—and (S)-fexofenadine—i.e., 62 L/h vs. 143 L/h [105].

#### 3.8.4. Effect of Transporter Genotypes on DFI of Fexofenadine

An interaction study focusing on the genotype-dependent effect of the SLCO2B1 c.1457C > T polymorphism has revealed significantly reduced C_max_ values of 43.6 ± 9.8 ng/mL, 44.7 ± 16.4 ng/mL, 46.2 ± 18.6 ng/mL for CC allele, CT allele, and TT allele, respectively, when fexofenadine was administered with AJ [91].

All the necessary PK parameters of the DFI of fexofenadine are expounded in Table 6.

### 3.9. Drug–Herb Interactions (DHI) of Fexofenadine

An interaction study has depicted that concurrent intake of St John’s Wort (SJWs) caused an escalation in C_max_ from 163 ± 43 ng/mL to 236 ± 96 ng/mL after a single dose, but long-term administration led to the reversal of these changes with a decrease in C_max_ value of 154 ± 75 ng/mL [68]. A study investigating quercetin’s inhibitory effect on fexofenadine has shown a significant decline in CL/F by 37% [86]. Another study was executed to assess the collective impact of fexofenadine and resveratrol, resulting in increased AUC_0–∞_ values from 2541.65 ± 527.18 ng.h/mL to 4512.33 ± 1265.17 ng.h/mL [100]. An in vivo study investigating the effect of multiple doses of danshen ethanol extract on MDR1 activity has resulted in enhanced CL/F of fexofenadine—i.e., 44.35 ± 25.57 L/h vs. 77.88 ± 31.20 L/h [102]. All the related PK parameters of DHI studies are reported in Table 7.

## 4. Discussion

This systematic review aimed to compile and assess human PK data of fexofenadine from published literature concerning the effects of dosage, age, stereoisomerism, disease states, genotypes, and drug interactions. Within the pool of retrieved articles, 21 examined healthy populations, 7 pertained to diseased populations, 4 delved into specific populations, 31 examined drug–drug interactions, 10 addressed drug–food interactions, and 14 investigated drug–herb interactions. The included studies have consistently cited C_max_ and AUC_0-∞_ as primary PK parameters. Merely one report exists on the IV administration of fexofenadine in humans, which has shown comparable PK parameters to an oral therapeutic dose, suggesting a possibility of the potential use of this research in future studies [21].

A study conducted on oral administration of fexofenadine has reported that the drug exhibited dose-proportional PK at doses ranging from 20–120 mg. However, following 240 mg, there was a little disproportional increase in exposure due to the saturation of transporters, which led to decreased permeability efficiency with elevating doses [40]. A clinical study has demonstrated that the bioavailability of fexofenadine drops by approximately 50% under fed conditions; hence, the drug is suggested to be taken on an empty stomach [48]. Another study has narrated that phenylalanine-free taste-masked fexofenadine ODT exhibited a greater extent of drug absorption probably because of quick disintegration and dissolution in saliva even without water; hence, it is cogitated to be appropriate for both geriatric and pediatric patients [56]. Furthermore, one study has revealed that subjects with the 2677AA/3435CC genotype combination had lower C_max_ values than those with other combinations of the 2677TT/3435TT SNPs. This difference was attributed to the fact that 2677TT/3435TT carriers had a higher concentration of P-gp substrate relative to 2677AA/3435CC carriers, indicating a significant interethnic variability in MDR1 haplotypes [43].

A clinical study elucidating the stereoselectivity of fexofenadine in healthy subjects has shown a significantly greater AUC_0-∞_ for the R-enantiomer relative to the S-enantiomer, likely due to the potential of chiral discrimination by P-glycoprotein [46]. Another study has displayed considerably reduced AUC_0–24_ values for S-fexofenadine in persons with the SLCO2B1*1/*1 allele relative to the SLCO2B1*1/*3 + *3/*3 allele. This indicates that the association of SLCO (encoding OATP) affects drug exposure in the liver and small intestine, resulting in different enantiomer dispositions [51]. Furthermore, a probe substrate-based cocktail study evaluating CYP enzymes and transporters activity influenced by genetic factors has revealed decreased CL/F of fexofenadine related to the CYP2D6 polymorphisms. This might be because individuals with the non-functional CYP2D64/*4 PM genotype lack CYP2D6 activity, which may lead to the accumulation of P-gp inhibitors, ultimately decreasing fexofenadine CL/F by impeding its efflux [60].

In patients suffering from advanced non-small cell lung cancer (NSCLC), the concurrent use of fexofenadine with single or multiple doses of osimertinib has resulted in increased fexofenadine exposure. Close monitoring is advised for patients receiving osimertinib alongside disposition-dependent P-gp drugs, as there is a risk of increased exposure to the concomitant medication, potentially leading to changes in tolerability [59]. Another study has depicted that PK of fexofenadine remained consistent in cystic fibrosis patients and age-mate healthy participants, but probenecid co-administration considerably augmented its C_max_ due to OAT3 inhibition in the kidneys [18,74]. Moreover, a study carried out in patients with ESRD undergoing conventional hemodialysis has reported a 63% decline in CL/F, and this decrease could impact the PK disposition via nonrenal clearance pathways, leading to reduced hepatic OATP and intestinal P-gp activity [50].

A study assessing the tolerability of fexofenadine oral suspension in children aged 2–5 years has exhibited a similar exposure level compared to children aged 6–11 years and adults. Therefore, it was considered a well-tolerated nonsedating treatment for children who find difficulty in swallowing tablets [49]. In parturient women, maternal-fetal PK of fexofenadine enantiomers has revealed approximately 18% transplacental transfer for both enantiomers, with the (R)-enantiomer exhibiting higher AUC_0–∞_ due to the interplay of efflux P-gp and uptake OATP transporters for chiral discrimination [61].

Most drug–drug interactions (DDI) of fexofenadine occur as a result of induction or inhibition of intestinal efflux or hepatic uptake transporters. A research study has demonstrated that ritonavir, as well as combination tablets of lopinavir/ritonavir and ritonavir/indinavir, substantially raised the systemic drug exposure of fexofenadine possibly by inhibiting hepatic and intestinal P-gp activity [76,87,96,99]. A different study has described that nelfinavir potentially lowered fexofenadine C_max_ as a result of elevated intestinal efflux and gastrointestinal P-gp activity; thus, dose adjustment is advisable to prevent potential side effects [85]. Another study was carried out in which cimetidine, a potent OCT inhibitor, when administered with fexofenadine, showed no significant plasma PK changes, indicating that alterations in OCT activity do not impact the in vivo disposition of fexofenadine [74]. Moreover, some research findings have highlighted that simultaneous use with azithromycin, Vitamin D3, and SSRI—i.e., paroxetine and fluvoxamine—resulted in a notable elevation of fexofenadine exposure, possibly due to the inhibition of P-gp activity; however, these changes were perceived to be well-tolerated [66,93,106]. Moreover, no anticipated PK changes were observed when fexofenadine was administered with diltiazem, sertraline, breviscapine, and metronidazole, suggesting that no dose adjustment is necessary [78,89,93,113].

A genotype-based DDI study has indicated that the T/T haplotype has 40% higher C_max_ than the G/C haplotype, and itraconazole co-administration boosts the C_max_ of both haplotypes by 3-fold due to P-gp inhibition with no association with MDR1 genetic variations [72]. A stereoisomerism-based DDI study has explicated that CL/F of both enantiomers of fexofenadine was significantly decreased by a single dose of rifampicin, likely by inhibiting hepatic or renal OATP1B3 and intestinal P-gp; thus, the dose adjustment is recommended to attain the desired therapeutic effect [19].

Drug–food interactions (DFI) play a vital role in clinical practice by altering drug effects. A clinical study has exhibited that AJ induced a volume-dependent decline in fexofenadine exposure through intestinal OATP2B1 activity; hence, taking the drug with water is preferred for optimal antihistamine effects [108]. Another study has narrated that co-administration of fexofenadine with GTE resulted in reduced exposure due to inhibition of intestinal OATP1A2 activity; hence, refraining the drug with green tea is suggested to prevent the possibility of therapeutic failure [114].

**Figure 4:**
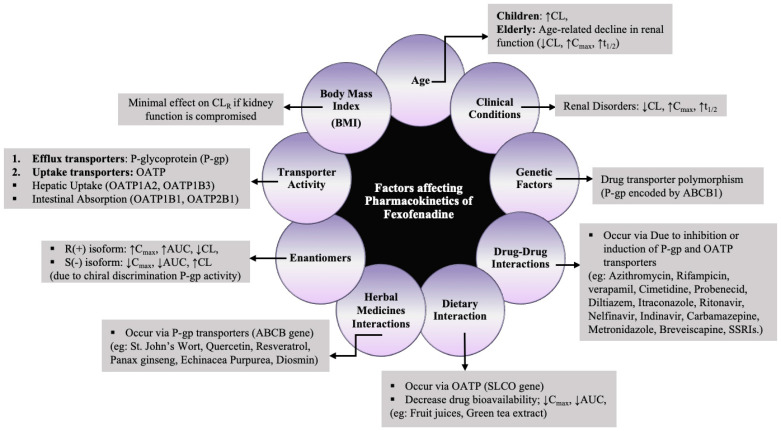
Factors affecting PK of fexofenadine. (**↓** Relative decrease in a specific parameter; ↑ Relative increase in a specific parameter).

In general, drug–herb interactions (DHI) pose a serious safety concern as they may lead to potentially fatal consequences. A study has revealed that the C_max_ of fexofenadine increased with SJWs over short-term use due to P-gp inhibition, while long-term use resulted in decreased C_max_ due to P-gp induction [68]. Different studies have reported that fexofenadine’s PK was unaffected by ginkgo biloba extract, Echinacea purpurea, Panax ginseng, and radix astragali extract granules; hence, these substances can be used together without any dosage adjustments [82,90,92,98]. Furthermore, a clinical study evaluating the inter-ethnic disparities in interaction with SJW has illustrated diverse CL/F of fexofenadine in various populations, possibly stemming from genetic variations and allelic differences in CYP3A4 and P-gp functionality [73]. In summary, all the factors influencing the PK of fexofenadine are elucidated in Figure 4.

The strength of the study lies in its comprehensive inclusion of PK studies from 1996 to 2023, spanning healthy, diseased, and special populations, alongside detailed analyses of drug interactions. The retrieval of 85 articles from four separate search engines reduces the potential for overlooking relevant studies. The limitation of this systematic review is the inclusion of only English language papers, which may overlook important research from other linguistic sources. Moreover, the presence of only one article focusing on parturient women limits the extent to which strong conclusions can be drawn regarding the pregnant population.

## 5. Conclusions

This review presents a comprehensive overview of the clinical PK of fexofenadine by compiling up-to-date information from all pertinent published studies. The analysis of the studies indicates a correlation between increasing doses and proportional elevations in PK parameters such as C_max_ and AUC_0–∞_. The PK variations were significant between healthy and diseased populations, exhibiting reduced CL/F values in the case of renal disease. Racemic fexofenadine displayed notably higher drug exposure for the R-enantiomer relative to the S-enantiomer. The induction or inhibition of drug transporters like P-gp and OATP potentiate the clinical relevance of fexofenadine interactions with other drugs, food, and herbs. Various fruit juices also influenced the fexofenadine PK, but the impact was deemed clinically important following the interactions with grapefruit juice and apple juice. Genetic variations also perpetrate drug interactions, resulting in changes to its effectiveness. The knowledge of these PK parameters and disparities in fexofenadine PK among healthy, diseased, and special populations, alongside the impact of stereoisomerism and genetic polymorphism, can assist clinicians in developing and evaluating the fexofenadine PK model. Moreover, the understanding of alterations in PK parameters regarding drug-drug, drug-food, and drug–herb interactions offers practitioners valuable insights into preempting adverse reactions, refining dosage strategies, and choosing appropriate treatments to achieve the intended outcomes.

## Figures and Tables

**Table 1:** Study Characteristics.

**Sr**	Population	Age (Years)	Drug	Dose (mg)	Frequency	Dosage Form	Analytical Method	Refs
1	Healthy	9.8 ± 1.8	Fexo	30, 60	SD	Susp	N/N	[38]
2	Healthy	18–51 ^a^	Fexo	10, 20, 40, 80, 130, 200, 280, 360, 480, 640, 800 ^a^	SD	Tab	HPLC-FD	[39]
		20–47 ^b^		20, 40, 80, 160, 260, 390, 520, 690 ^b^	BID			
3	Healthy	19–45	Fexo	20, 60, 120, 240	OD ^c^	Tab	HPLC-MS	[40]
					BID ^d^			
4	Healthy	28–32.4	Fexo	180	SD	Tab	LC-MS	[41]
5	Healthy	N/N	Fexo	180	SD	Tab	LC-MS	[42]
6	Healthy	21–39	Fexo	180	SD	Tab	HPLC-FD	[43]
7	Healthy	20–53	Fexo	60	BID	Tab	LC-MS-MS	[44]
8	Healthy	18–50	Fexo	180	SD	Tab	HPLC	[45]
9	Healthy	20–22	Fexo	60	SD	Tab	HPLC	[46]
10	Healthy	N/N	Fexo	120	SD	Tab	LC-MS-MS	[47]
11	Healthy	N/N	Fexo	180	SD	Tab	LC-MS-MS	[48]
12	Healthy	2–5	Fexo	30	SD	Susp	LC-MS-MS	[49]
13	Healthy	29–38	Fexo	180	SD	Tab	LC-MS	[18]
	Diseased (Cystic fibrosis)	25–28	Probenecid	1000	BID			
14	Healthy	20–37	Fexo	180	SD	Tab	HPLC	[20]
15	Healthy	45.9 ± 13.2	Fexo	120	SD	Tab	LC–MS–MS	[50]
	Diseased (End stage renal disease)	51.5 ± 15.6						
16	Healthy	22–36	Fexo	60	SD	Tab	HPLC	[51]
17	Healthy	18–27	Fexo	60, 120	SD	Tab	LC-MS-MS	[52]
18	Healthy	20–34	Fexo	100 ^e^	SD	Sol	LC-MS-MS	[13]
				60		Tab		
19	Healthy	18–55	Fexo	120	N/N	Tab	HPLC-FD	[21]
				100 ^e^		IV		
20	Healthy	N/N	Fexo	120	SD	Tab	LC-MS-MS-ESI	[53]
21	Healthy	23.5 ± 2.9	Fexo	180	SD	Tab	HPLC	[54]
22	Diseased (Glomerulonephritis)	41 ± 17	Fexo	60	SD	Tab	LC–MS-MS	[55]
23	Healthy	24–30	Fexo	30	N/N	Tab	HPLC	[56]
24	Healthy + Diseased (Kidney Disease)	>18	Fexo	120	SD	Tab	N/N	[57]
25	Healthy	21–29	Fexo	120	SD	Tab	HPLC	[58]
26	Diseased (EFGR mutated Non-Small-Cell-Lung-Cancer)	44–87	Fexo	120	SD	Tab	RP-HPLC-MS-MS	[59]
			Osimertinib	80	SD, MD			
27	Healthy	30 ± 5	Fexo	10	SD	Sol	LC–MS-MS	[60]
28	Healthy (Parturient women)	N/N	Fexo	60	SD	Tab	LC-MS-MS	[61]
29	Diseased (Hemodialysis Patients)	47–78	Fexo	120	SD	Tab	UPLC-MS-MS	[62]
30	Healthy	18–55	Fexo	120	SD	Tab	HPLC-MS-MS	[63]
31	Healthy	18–55	Fexo	30	SD	Susp	N/N	[64]
32	Diseased (Coeliac disease)	17–79	Fexo	120	SD	Tab	HPLC	[65]
33	Healthy	19–46	Fexo	60	BID	Tab	LC-MS-MS	[66]
			Azithromycin	500	OD			
34	Healthy	22–35 ^f^	Fexo	60	OD	Tab	HPLC-FD	[67]
		65–76 ^g^	Rifampin	600				
35	Healthy	32 ± 7	Fexo	60	SD	Tab	HPLC-FD	[68]
			St John’s wort	900				
36	Healthy	19–44	Fexo	60	SD	Tab	LC-MS-MS	[69]
37	Healthy	19–40 ^h^	Fexo	60	SD	Tab	HPLC	[70]
		19–28 ^i^						
38	Healthy	22–34	Fexo	40	N/N	Tab	HPLC-UV	[22]
			Verapamil	100				
39	Healthy	19–31 ^h^	Fexo	180	SD	Tab	HPLC	[71]
		20–55 ^i^	St John’s wort	300	TID			
40	Healthy	21–28	Fexo	180	SD	Tab	UV	[72]
			Itraconazole	200				
41	Healthy	19–51	Fexo	60	SD	Tab	HPLC (LC-MS-MS)	[73]
			St John’s wort	300	TID			
42	Healthy	20–40	Fexo	120	SD	Tab	HPLC	[74]
			Verapamil	240	TID			
			Cimetidine	800	BID			
			Probenecid	2000	BID			
43	Healthy	23–47	Fexo	120	SD	Tab	HPLC	[75]
44	Healthy	19–48	Fexo	120	SD	Tab	LC-MS-MS	[76]
			Lopinavir	40				
			Ritonavir	100				
45	Healthy	29 ± 9	Fexo	60	SD	Tab	HPLC-MS	[77]
			Verapamil	240				
46	Healthy	21–25	Fexo	120	SD	Tab	HPLC	[78]
			Itraconazole	50	BID			
			Diltiazem	100				
47	Healthy	20–24	Fexo	60	SD	Tab	HPLC	[79]
			Itraconazole	50, 100, 200				
48	Healthy	21–34	Fexo	60	OD	Tab	HPLC	[80]
			Itraconazole	200				
49	Healthy	19–51 ^j^	Fexo	120	SD	Tab	HPLC	[81]
		20–52 ^k^						
50	Healthy	23–48	Fexo	120	SD	Tab	LC-MS-MS	[82]
			Ginkgo biloba extract	120				
51	Healthy	19–34	Fexo	60	N/N	Tab	SPE-LC-MS	[83]
			Ritonavir	200				
52	Healthy	21–24	Fexo	60	SD	Tab	HPLC	[84]
			Itraconazole	200				
53	Healthy	19–34	Fexo	60	N/N	Tab	SPE-LC-MS	[85]
			Nelfinavir	1250	BID			
54	Healthy	24–31	Fexo	60	SD	Tab	HPLC	[86]
			Quercetin	500	TID			
55	Healthy	18–34	Fexo	60	SD	Tab	MS	[87]
			Ritonavir	100	BID			
			Indinavir	800				
56	Healthy	22–36	Fexo	120	SD	Tab	HPLC	[88]
			Verapamil	80	TID			
57	Healthy	21–39	Fexo	60	SD	Tab	HPLC	[7]
			Carbamazepine	100	TID			
58	Healthy	22–30	Fexo	120	SD	Tab	HPLC	[89]
			Metronidazole	500	TID			
59	Healthy	18–50	Fexo	120	SD	Tab	UPLC-MS-MS	[90]
			Echinacea purpurea	500	TID			
60	Healthy	20–40	Fexo	60	SD	Tab	HPLC-SPE	[91]
61	Healthy	18–50	Fexo	120	SD	Tab	UPLC-MS-MS	[92]
			Panax ginseng	500	BID			
62	Healthy	28.6	Fexo	60	SD	Tab	HPLC	[93]
			Fluvoxamine	50	OD			
			Paroxetine	20				
			Sertraline	50				
63	Healthy	25.2 ± 5.9	Fexo	60	SD	Tab	HPLC	[16]
			Carbamazepine	100	TID			
64	Healthy	18–31	Fexo	60	SD	Tab	SPE- LC-MS	[94]
			Indinavir	800	TID			
65	Healthy	26–51	Fexo	25 ^e^	SD	Tab	HPLC-AMS	[95]
			Fluvoxamine/Ketoconazole	400/100				
66	Healthy	20–40	Fexo	60	SD	Tab	SPE- LC-MS	[96]
			Lopinavir	400	BID			
			Ritonavir	100				
67	Healthy	23–54 ^h^	Fexo	120	SD	Tab	HPLC-MS	[97]
		23–60 ^i^						
68	Healthy	25–28	Fexo	120	SD	Tab	LC-FD	[98]
			Radix Astragali extract	4000	BID	Gran		
69	Healthy	20–41	Fexo	60	SD	Tab	LC-MS-MS	[19]
			Rifampin	600	OD			
70	Healthy	22–27	Fexo	100 ^e^	SD	Tab	LC-MS-MS	[99]
			Ritonavir	20, 100				
71	Healthy	26–31	Fexo	120	SD	Tab	LC-MS-MS	[100]
			Resveratrol	500	OD			
72	Healthy	20–42	Fexo	60	SD	Tab	HPLC	[101]
73	Healthy	25–30	Fexo	60	SD	Tab	LC-MS-MS	[102]
			Danshen ethanol extract	1000	TID			
74	Healthy	20–40	Fexo	50 ^e^	N/N	Tab	LC-MS-MS	[103]
			Cremophor EL	720, 1440				
75	Healthy	21–39	Fexo	60	SD	Tab	HPLC	[104]
			Rifampin	450	OD			
76	Healthy	25.0 ± 4.9	Fexo	60	SD	Tab	HPLC	[105]
77	Healthy	26 ± 2	Fexo	120	OD	Tab	LC-MS-MS	[106]
			Vitamin D3	0.5 ^e^				
78	Healthy	25.6 ± 2.6	Fexo	30	SD	Tab	LC-MS-MS	[107]
			Fermented red ginseng	70 ^l^	OD			
79	Healthy	20–35	Fexo	60	SD	Tab	HPLC	[108]
80	Healthy	27–32	Fexo	120	SD	Tab	LC-MS-MS	[109]
			Diosmin	500				
81	Healthy	26–32	Fexo	120	SD	Tab	LC-MS-MS	[110]
			Piperine	20	OD			
82	Healthy	31.5 ± 5	Fexo	10	SD	Tab	LC-MS-MS	[111]
			Propolis extract	125	TID			
83	Healthy	18–36	Fexo	25	SD	Tab	HPLC-MS-MS	[112]
84	Healthy	20–28	Fexo	120	SD	Tab	UHPLC-MS-MS	[113]
			Breviscapine	120	OD			
85	Healthy	21–45	Fexo	60	SD	Tab	UPLC-FD	[114]

Fexo: Fexofenadine; N/N: Not Narrated; SD: Single dose; MD: Multiple dose; Refs: References; OD: Once daily; BID: Twice daily; TID: Thrice daily; IV: Intravenous infusion; Tab: Tablet; Gran: Granules; Sol: Solution; Susp: Suspension; HPLC: High-Performance Liquid Chromatography; UV: Ultraviolet detection; HPLC-FD: (HPLC) Fluorescence detection; HPLC-MS: HPLC-mass-spectrometry; HPLC-UV: HPLC with ultraviolet detection; LC-MS: Liquid chromatography-mass spectrometry; LC-MS-MS: Liquid chromatography with tandem mass spectrometric detection methods; LC-MS-MS-ESI: Liquid Chromatography-Electrospray Ionization-Mass Spectrometry; RP-HPLC-MS/MS: Reversed-phase high-performance liquid chromatography-tandem mass spectrometry; HPLC-AMS: HPLC with sensitive accelerator mass spectrometry; MS: Mass spectrometry; SPE-LC-MS: Solid-phase extraction and electrospray liquid chromatography-mass spectrometry; UHPLC-MS-MS: Ultra-high performance liquid chromatography-tandem mass spectrometry; UPLC-MS-MS: Ultra-performance liquid chromatography with detection by tandem mass spectrometry; ^a^ Single dose group; ^b^ Multiple dose group; ^c^ Day 1; ^d^ Day 3–7; ^e^ μg; ^f^ Young volunteers; ^g^ Elderly Volunteers; ^h^ Male; ^i^ Female; ^j^ Naringin-fexofenadine group; ^k^ Furanocoumarin-fexofenadine group; ^l^ mL.

**Table 2:** Clinical Pharmacokinetics of Fexofenadine in Healthy Population.

Sr.	Dosage (mg)		C_max_ (ng/mL)	T_max_ (h)	t_1/2_ (h)	AUC_0–∞_ (ng.h/mL)	CL/F (L/h)	CL_R_ (L/h)	Refs
Intravenous administration									
1	100 ^a^		4.7 (11)	0.5 (0)	8.10 (25)	7.96(18)	13 (12) ^q^	N/N	[21]
	100 ^a^ + 120 ^b^		3.97 (24)	0.50 (0)	10 (27)	7.37(24)	16 (24) ^q^	N/N	
Oral administration									
2	10 ^c^		45.6 (88.8)	1.17 (22.13)	N/N	22.9 (65.5)	55.96 (46.9)	N/N	[39]
	20 ^c^		72.8 (28.9)	1.08 (18.8)	N/N	415.3 (37.6)	53.34 (54.3)	N/N	
	40 ^c^		176.4 (28.3)	1.00 (31.6)	N/N	866.1 (18.1)	44.33 (19.2)	N/N	
	80 ^c^		502.3 (36.8)	1.08 (18.8)	N/N	2400 (37.8)	34.86 (37.8)	N/N	
	130 ^c^		846.2 (34.7)	1.17 (22.1)	N/N	3747 (28.4)	34.47 (26.4)	N/N	
	200 ^c^		1267 (34.7)	1.17 (22.1)	N/N	5994 (29.0)	33.36 (28.4)	N/N	
	280 ^c^		1908 (41.9)	0.98 (6.3)	N/N	8156 (34.5)	36.55 (46.9)	N/N	
	360 ^c^		31344 (48.3)	1.17 (22.1)	9.4 (23.2)	13,814 (43.5)	27.67 (35.2)	N/N	
	480 ^c^		3258 (41.2)	1.00 (0.00)	11.2 (23.4)	14,075 (33.3)	34.44 (28.3)	N/N	
	640 ^c^		4028 (26.1)	1.25 (21.9)	14.0 (12.1)	18,070 (25.1)	34.71 (23.7)	N/N	
	800 ^c^		6383 (48.4)	1.33 (30.6)	7.7 (19.2)	28,396 (47.5)	29.84 (31.3)	N/N	
	20 ^d^		57.9 (59.5)	1.17 (65.5)	N/N	N/N	85.39 (64.7)	N/N	
	40 ^d^		219.6 (73.2)	0.83 (69.3)	N/N	N/N	41.73 (45.5)	N/N	
	80 ^d^		327.0 (12.8)	1.33 (21.7)	N/N	N/N	43.33 (6.3)	N/N	
	160 ^d^		785.3 (38.6)	1.17 (24.7)	10.53 (69.4)	N/N	43.44 (20.1)	N/N	
	260 ^d^		1567 (25.0)	1.00 (0.00)	12.41 (71.0)	N/N	39.74 (29.4)	N/N	
	390 ^d^		3369 (16.2)	1.00 (0.00)	12.11 (19.2)	N/N	25.53 (21.8)	N/N	
	520 ^d^		3075 (75.0)	1.00 (0.00)	12.92 (38.1)	N/N	48.54 (49.3)	N/N	
	690 ^d^		4677 (21.0)	1.17 (24.7)	8.80 (35.9)	N/N	31.83 (30.7)	N/N	
3	20 ^c^		57 (46)	1.35 (53)	16.2 (51)	416 (36)	50.4 (35)	N/N	[40]
	60 ^c^		209 (45)	1.42 (50)	13.1 (30)	1348 (41)	50.6 (53)	N/N	
	120 ^c^		427 (40)	1.44 (47)	13.1 (43)	2682 (34)	47.8 (42)	N/N	
	240 ^c^		1119 (49)	1.52 (41)	14.0 (46)	6571 (35)	38.0 (33)	N/N	
	20 ^d^		93 (45)	1.08 (32)	14.7 (39)	N/N	42.2 (34)	N/N	
	60 ^d^		286 (50)	1.31 (45)	14.4 (39)	N/N	43.6 (45)	N/N	
	120 ^d^		602 (42)	1.33 (45)	11.3 (33)	N/N	39.0 (30)	N/N	
	240 ^d^		1530 (36)	1.02 (35)	14.0 (40)	N/N	35.4 (30)	N/N	
4	180		734.5 ± 261.3 ^n^	1.5 ± 0.6	19.1 ± 7.0	4107.5 ± 1837.4	51.468 ± 26.016	N/N	[42]
5	60		255.91 (145.39)	2.00 (0.71)	N/N	N/N	N/N	N/N	[44]
6	60		152.62 ± 74.18	4.10 ± 0.88	11.14 ± 4.95	978.19 ± 411.06	72.24 ± 31.01	N/N	[52]
	120		365.98 ± 168.02	3.58 ± 1.17	9.29 ± 3.61	2437.5 ± 885.9	55.64 ± 21.32	N/N	
7	100 ^e^		0.632 ± 0.245	1.5 (0.5–2.0)	3.2 ± 0.4	N/N	N/N	N/N	[13]
	60		275 ± 145	2.0 (1.0–4.0)	2.9 ±0.3	N/N	N/N	N/N	
8	180		703.76 ± 298.94	1.90 ± 0.81	12.18 ± 3.61	4582.52 ± 1812.59	N/N	N/N	[54]
9	60		179.083 ± 27.064	2.666 ± 0.516	5.229 ± 3.699	1628.622 ± 928.477	N/N	N/N	[56]
	60 ^f^		199.297 ± 29.071	1.833 ± 0.408	6.639 ± 2.830	1856.098 ± 692.314	N/N	N/N	
10	120		300 ± 50	1.5 ± 0.7	12.0 ± 4.3	1800 ± 810	73.0 ± 29.5 ^m^	N/N	[58]
11	100 ^e^		0.31 (21)	1.2 (59)	16 (45)	2.77 (18)	N/N	N/N	[21]
	100 ^a^ + 120 ^b^		318 (32)	2.7 (70)	12 (27)	2210 (33)	N/N	N/N	
Bioequivalence Studies									
12	180	TF	625	1.84 ± 0.87	2.97 ± 0.32	2954	N/N	N/N	[45]
		RF	629.5	1.86 ± 0.77	2.94 ± 0.29	3012	N/N	N/N	
13	120	TF	507.5 ± 151.5	2.6 ± 0.8	7.85 ± 2.0 5	2699 ± 737	N/N	N/N	[47]
		RF	475.3 ± 209.8	2.4 ± 0.9	7.48 ± 1.13	2725 ± 950	N/N	N/N	
14	180	TF	1206.3 ± 619.0	2.6 ± 1.7	7.2 ± 4.0	8911.4 ± 3870.0	N/N	N/N	[20]
		RF	1172.6 ± 493.7	2.0 ± 1.0	9.9 ± 3.1	9363.9 ± 2668.0	N/N	N/N	
15	120	TF ^g^	453 ± 168	3.74 ± 1.28	10.7 ± 2.53	3164 ± 1388	N/N	N/N	[53]
		RF ^g^	463 ± 182	3.72 ± 1.23	10.7 ± 3.29	3175 ± 1118	N/N	N/N	
16	120	RF ^g^	376.441 ± 202.552	2.66	5.326	2228.951 ± 1094.014	N/N	N/N	[63]
		TF ^g^	368.247 ± 190.075	3	5.044	2080.045 ± 988.172	N/N	N/N	
Bioequivalence Study of Fasted state and Fed state									
17	180 ^i^	TF ^h^	655 ± 395	2.00 ± 1.14	6.94 ± 4.13	4323 ± 1578	N/N	N/N	[48]
		RF	763 ± 454	1.67 ± 0.85	6.60 ± 4.39	4938 ± 2133	N/N	N/N	
	180 ^j^	TF ^h^	464 ± 252	2.50 ± 1.18	5.11 ± 6.55	3188 ± 2289	N/N	N/N	
		RF	404 ± 185	2.75 ± 1.59	3.95 ± 1.32	2453 ± 770	N/N	N/N	
Enantiomers									
18	60	R (+)	153 ± 17	2.4 ± 1.1	3.4 ± 0.6	843 ± 153 ^a^	0.648 ± 0.174 ^m^	0.084 ± 0.024	[46]
		S (−)	101 ± 27	2.4 ± 1.1	2.9 ± 0.8	496 ± 131	1.122 ± 0.3 ^m^	0.162 ± 0.048	
Genotypes encoding drug transporters									
19	180	G2677T (Exon 21) ^k^							[41]
		GG	701.9 ± 308.0	0.6/1.5/3.0	14.6 ± 5.7	3864.3 ± 1531.6	81.948 ± 23.376	4.152 ± 0.846	
		GT	450.0 ± 127.4	1.5/2.5/5.0	15.6 ± 2.6	2969.1 ± 1175.2	66.168 ± 25.974	5.16 ± 1.17	
		TT	663.4 ± 252.6	0.5/2.0/3.0	15.9 ± 4.7	4114.9 ± 2137.2	49.464 ± 18.144	3.834 ± 0.396	
		C3435T (Exon 26) ^k^							
		CC	642.7 ± 308.8	0.6/1.3/3.0	14.2 ± 5.2	3567.1 ± 1535.5	57.462 ± 26.478	4.482 ± 1.014	
		TT	620.3 ± 222.9	0.5/2.0/5.0	16.0 ± 4.2	3910.1 ± 1894.8	50.472 ± 16.998	4.188 ± 0.96	
20	180	G2677T/C3435T (Exon 21/Exon 26) ^k^							[43]
		GG/CC	628 ± 189	2.4 ± 1.2	5.0 ± 0.9	N/N	N/N	N/N	
		GT/CT	927 ± 128	2.0 ± 1.1	4.2 ± 1.2	N/N	N/N	N/N	
		TT/TT	958 ± 408	2.4 ± 2.1	4.5 ± 0.5	N/N	N/N	N/N	
		GA/CC	782 ± 280	1.4 ± 0.6	4.7 ± 0.7	N/N	N/N	N/N	
		TA/CT	829 ± 255	2.3 ± 1.2	5.6 ± 1.7	N/N	N/N	N/N	
		AA/CC	494 ± 81	1.7 ± 0.3	4.8 ± 0.6	N/N	N/N	N/N	
		C3435T (Exon 26) ^k^							
		CC	673 ± 242	1.8 ± 0.9	4.8 ± 0.7	N/N	N/N	N/N	
		CT	878 ± 199	2.2 ± 1.1	4.9 ± 1.6	N/N	N/N	N/N	
		TT	958 ± 408	2.4 ± 2.1	4.5 ± 0.5	N/N	N/N	N/N	
21	10	CYP EM	8.12	N/N	N/N	48.58	205.87	N/N	[60]
		CYP2C9 PM	5.44	N/N	N/N	34.06	293.56	N/N	
		CYP2D6 PM	14.39	N/N	N/N	94.15	106.21	N/N	
22	60 R (+)	SLCO1B1 ^l^							
		1a/1a + 1a/1b + 1b/1b	144 (40–269) ^o^	N/N	3.3 (2.5–5.7)	812 (241–1366) ^p^	N/N	N/N	[51]
		1a/*15 + 1b/*15 + *15/*15	136 (61–159)	N/N	4.5 (2.8–6.2)	848 (592–1004) ^p^	N/N	N/N	
		SLCO1B3 ^l^							
		334T/T + T/G	138 (40–269)	N/N	3.7 (2.5–5.3)	832 (241–1328) ^p^	N/N	N/N	
		334G/G	140 (76–182)	N/N	3.5 (2.5–6.2)	860 (493–1366) ^p^	N/N	N/N	
		SLCO2B1 ^l^							
		*1/*1	148 (40–269)	N/N	3.3 (2.5–5.7)	764 (241–1113) ^p^	N/N	N/N	
		*1/*3 + *3/*3	133 (61–179)	N/N	4 (2.5–6.2)	916 (496–1366) ^p^	N/N	N/N	
	60 S (−)	SLCO1B1 ^l^							
		1a/1a + 1a/1b + 1b/1b	104 (27–186)	N/N	2.8 (1.8–4.9)	469 (112–1081) ^p^	N/N	N/N	
		1a/*15 + 1b/*15 + *15/*15	122 (50–135)	N/N	3.6 (2.4–7.7)	546 (310–1123) ^p^	N/N	N/N	
		SLCO1B3 ^l^							
		334T/T + T/G	122 (27–186)	N/N	3.3 (2.2–4.9)	519 (112–777) ^p^	N/N	N/N	
		334G/G	104 (49–152)	N/N	3.1 (1.8–7.7)	424 (298–1123) ^p^	N/N	N/N	
		SLCO2B1 ^l^							
		*1/*1	111 (27–186)	N/N	2.6 (2.0–4.9)	446 (112–643) ^p^	N/N	N/N	
		*1/*3 + *3/*3	113 (53–152)	N/N	3.6 (1.8–7.7)	675 (298–1123) ^p^	N/N	N/N	

Refs: References; R (+): R (+) enantiomer of fexofenadine; S (–): S (–) enantiomer of fexofenadine; N/N: Not Narrated; AUC_0–∞_: Area under the curve from time 0 to infinity; CL/F: Oral Clearance; CL_R_: Renal clearance; C_max_: Maximum plasma concentration; t_1/2_: half-life; T_max_: Time to reach maximum plasma concentration; EM: Extensive Metabolizers; PM: Poor Metabolizers; SLCO1B1: Solute Carrier Organic Anion Transporter Family Member 1B1; SLCO1B3: Solute Carrier Organic Anion Transporter Family Member 1B3; SLCO2B1: Solute Carrier Organic Anion Transporter Family Member 2B1; CC, CT, TT, TA, AA, GA, GT, GG, 1a/1a + 1a/1b + 1b/1b, 1a/*15 + 1b/*15 + *15/*15, 334T/T + T/G, 334G/G, *1/*1, *1/*3 + *3/*3: Alleles; 2677GG/3435CC: Genotype polymorphism; ^a^ Intravenous microdose (μg); ^b^ Oral therapeutic dose (mg); ^c^ Single Oral Dose; ^d^ Multiple Oral Dose; ^e^ μg; ^f^ Phenylalanine-free taste-masked Orodispersible tablet; ^g^ A fixed-dose combination tablet of Fexofenadine/montelukast 120 mg/10 mg; ^h^ A fixed-dose combination tablet of Fexofenadine/Pseudoephedrine 180 mg/240 mg; ^i^ Fasted state; ^j^ Fed state; ^k^ Genotype group; ^l^ Transporter genotype group; ^m^ L/h/kg; ^n^ Data presented in mean ± standard deviation; ^o^ Data presented in median (range); ^p^ AUC_0–24_: Area under the curve from time 0 to 24 h; ^q^ Total Body Clearance.

**Table 3:** Clinical Pharmacokinetics of Fexofenadine in Diseased Population.

Sr.	Dose (mg)	Population	C_max_ (ng/mL)	T_max_ (h)	t_1/2_ (h)	AUC_0–∞_ (ng.h/mL)	CL/F (L/h)	CL_R_ (L/h)	Refs
1	180	Healthy	390 (300–650)	2 (1.4–3.0)	N/N	N/N	73.27 (50.50–123.20)	5 (4.28–6.27) ^j^	[18]
		Cystic fibrosis ^a^	500 (320–630)	2 (2.0–3.0)	N/N	N/N	81.16 (72.04–106.0)	5.47 (3.90–6.32) ^j^	
		Cystic fibrosis ^a+b^	700 (470–1210)	2 (1.5–2.5)	N/N	N/N	53.31 (34.02–69.09)	1.69 (1.10–2.20) ^j^	
2	120	Healthy	267.2 ± 130.3 ^i^	2 (1–4)	3.4 ± 0.9	1380.2 ± 674.1	102.8 ± 37.9	N/N	[50]
		ESRD	464.2 ± 194.7	2 (1–6)	4.6 ± 1.3	3926.2 ± 1842.8	37.9 ± 19.5	N/N	
3	60	Glomerulonephritis	140 ± 83	4.6 ± 3.0	11.5 ± 5.6	1351 ± 723	58.8 ± 34.4	N/N	[55]
4	120	ESRD ^c^	320 (222–761)	3 (1.5–7.0)	N/N	2355 (1516–67497)	72.7 (33.4–152.8)	N/N	[62]
		ESRD ^d^	267 (157–618)	2	N/N	2785 (1274–11945)	78.6 (37.1–164.6)	N/N	
5	120	Healthy	453 ± 32	2.1 ± 0.2	3.0 ± 0.2	2508 ± 190	N/N	N/N	[65]
		Coeliac disease ^e^	440 ± 73	2.0 ± 0.3	3.1 ± 0.3	2558 ± 354	N/N	N/N	
		Coeliac disease ^f^	513 ± 96	2.7 ± 0.4	4.0 ± 0.6	3256 ± 684	N/N	N/N	
		Coeliac disease ^g^	523 ± 104	3.2 ± 0.5	4.4 ± 0.8	2997 ± 596	N/N	N/N	
6	120	Healthy	246.7 ± 135.2	1.8 (1.0–5.0)	N/N	N/N	N/N	N/N	[57]
		NDD-CKD	591.0 ± 278.8	2.5 (1.5–5.0)	N/N	N/N	N/N	N/N	
		Hemodialysis	531.1 ± 406.2	4.0 (1.0–8.0)	N/N	N/N	N/N	N/N	
		Peritoneal dialysis	413.9 ± 170.5	4 (2.0–6.0)	N/N	N/N	N/N	N/N	
7	120	NSCLC ^a^	497.7 (66.9)	2 (0.97–8.00)	12.6 ± 7.6	3291 (56.9)	38.6 ± 19.3	N/N	[59]
		NSCLC ^a+h^	890.9 (55.6)	2.96 (1.08–6.02)	9.7 ± 5.6	5081 (53.1)	24.5 ± 10.7	N/N	
		NSCLC ^a+h^	615 (39.6)	2.93(1.00–8.13)	8.7 ± 4.8	3996 (33.5)	29.4 ± 9.4	N/N	

N/N: Not Narrated; Refs: Reference; AUC_0–∞_: Area under the curve from time 0 to infinity; CL/F: Oral Clearance; CL_R_: Renal clearance; C_max_: Maximum plasma concentration; t_1/2_: half-life; T_max_: Time to reach maximum plasma concentration; ESRD: End-stage renal disease; NSCLC: Non–Small Cell Lung Cancer; NDD-CKD: Non-dialysis-dependent chronic kidney disease; ^a^ Fexofenadine; ^b^ Probenecid; ^c^ After hemodialysis; ^d^ Before hemodialysis; ^e^ group A (normal villous blunting), ^f^ group B + C (mild villous blunting), ^g^ group D (moderate to severe villous blunting); ^h^ Osimertinib; ^i^ Data presented in mean ± standard deviation; ^j^ L/h/1.72 m^2^.

**Table 4:** Clinical Pharmacokinetics of Fexofenadine in Special Population.

Sr.	Dose (mg)		C_max_ (ng/mL)	T_max_ (h)	t_1/2_ (h)	AUC_0–∞_ (ng.h/mL)	CL/F (L/h)	CL_R_ (L/h)	Refs
1. Children									
1	30		178 ± 22	2.4 ± 0.2	18.3 ± 2.0	1090 ± 125	14.4 ± 2 ^a^	N/N	[38]
	60		286 ± 34	2.4 ± 0.2	17.6 ± 1.0	1892 ± 129	18.4 ± 2.4 ^a^	N/N	
2	30		224 (110–437) ^d^	1 (1.0–4.0)	N/N	N/N	N/N	N/N	[49]
3	TF	30 ^b^	123 (64)	1 (0.49–3.95)	12.7 (6.2)	685 (326)	N/N	N/N	[64]
		30 ^c^	109 (56)	0.98 (0.48–1.99)	12.7 (6.2)	635 (288)	N/N	N/N	
	RF	30 ^b^	108 (58)	0.99 (0.49–3.11)	11.6 (4.9)	610 (277)	N/N	N/N	
		30 ^c^	102 (53)	0.99 (0.47–3.02)	12.4 (5.8)	587 (258)	N/N	N/N	
2. Parturient Women (Enantiomers)									
4	S (−)	60	22.81 (13.87–36.02)	3.07 (2.04–4.07)	3.8 (2.59–5.37)	267.67 (156.79–302.68)	105.05 (92.65–182.89)	8.57 (5.87–8.87)	[61]
	R (+)	60	36.53 (24.62–53.66)	2.47 (2.02–3.23)	3.89 (2.99–5.59)	423.2 (265.21–458.23)	66.2 (61.18–111.26)	5.06 (3.73–5.61)	

N/N: Not Narrated; Refs: Reference; AUC_0–∞_: area under the curve from time 0 to infinity; CL/F: Oral Clearance; CL_R_: Renal clearance; C_max_: Maximum plasma concentration; t_1/2_: half-life; T_max_: Time to reach maximum plasma concentration; TF: Test Formulation; RF: Reference formulation; R (+): R (+) enantiomer of fexofenadine; S (–): S (–) enantiomer of fexofenadine; ^a^ mL/min/kg; ^b^ First administration; ^c^ Second administration; ^d^ Data presented in mean (range).

**Table 5:** Pharmacokinetic Parameters of DDI of fexofenadine.

Sr.	Fexo Dose (mg)	Drugs		C_max_ (ng/mL)	T_max_ (h)	t_1/2_ (h)	AUC_0–∞_ (ng.h/mL)	CL/F (L/h)	CL_R_ (L/h)	Refs
1	60	Fexo		199 (52)	N/N	N/N	N/N	N/N	N/N	[66]
		Fexo + Azithromycin		349 (56) → ↑75%	N/N	N/N	N/N	N/N	N/N	
2	40	Fexo		N/N	N/N	2.23 ± 0.31	161 ± 181 ^t^	N/N	N/N	[22]
		Fexo + Verapamil		N/N	N/N	N/N	664 ± 537 ^s^→ ↑3-fold	N/N	N/N	
3	120	Fexo		611 ± 206	1.5	11.0 ± 5.1	3637 ± 1199	N/N	13.8 ± 4.68	[74]
		Fexo + Verapamil		1807 ± 692 → ↑2-fold	1.5	7.9 ± 2.4	9136 ± 3573 → ↑1.5-fold	N/N	13.44 ± 5.58	
		Fexo + Cimetidine		609 ± 318	2	9.7 ± 3.1	4124 ± 2019	N/N	9.12 ± 4.2	
		Fexo + Probenecid		767 ± 490 → ↑25%	2	8.5 ± 1.6	6150 ± 3972 → ↑69%	N/N	4.44 ± 3.12	
4	120	Fexo		289	2.5	5.1	1568	77	N/N	[76]
		Fexo + Ritonavir		635 → ↑1.2-fold	2	5.7	4208 → ↑1.7-fold	29 → ↓62%	N/N	
		Fexo + Lopinavir/Ritonavir ^e^		1115 → ↑3-fold	2	4.9	6498 → ↑3-fold	18 → ↓77%	N/N	
		Fexo + Lopinavir/Ritonavir ^f^		888 → ↑2-fold	2.3	5	5456 → ↑2.5-fold	22 → ↓71%	N/N	
5	60	Fexo		114 ± 45	2.3 ± 0.6	3.1 ± 1.2	N/N	156 ± 69	12 ± 5.1	[77]
		Fexo + Verapamil (Day 1)		165 ± 42 → ↑45%	2.4 ± 0.7	3 ± 0.5	N/N	98 ± 54 → ↓37%	9.8 ± 4.8	
		Fexo + Verapamil (Day 10)		148 ± 39 → ↑30%	2.7 ± 0.9	2.9 ± 0.5	N/N	102 ± 40 → ↓35%	14.5 ± 5.7	
		Fexo + Verapamil (Day 38)		126 ± 43 → ↑11%	2.3 ± 0.3	2.9 ± 0.6	N/N	129 ± 89 → ↓17%	16.6 ± 9.6	
6	120	Fexo		N/N	1 (0.5–6)	10.1 ± 5.3	N/N	0.5916 ± 0.236 ^t^	N/N	[78]
		Fexo + Diltiazem		N/N	1 (1–4)	10.4 ± 4.4	N/N	0.554 ± 0.2576 ^t^	N/N	
		Fexo + Itraconazole		N/N	2 (1–4)	10.3 ± 4.3	N/N	0.2234 ± 0.112 ^t^ → ↓62%	N/N	
7	60	Fexo		292 ± 173	2 (1–4)	6.2 ± 2.7	1701 ± 960	0.819 ± 0.402 ^t^	63.5 ± 15.8	[79]
		Fexo + Itraconazole ^g^		525 ± 168 → ↑80%	2 (1–4)	6.5 ± 2.0	3554 ± 1220 → ↑50%	0.344 ± 0.155 ^t^ → ↓58%	58.9 ± 19.6	
		Fexo + Itraconazole ^h^		598 ± 194 → ↑1-fold	2 (1–4)	6.6 ± 1.7	4308 ± 1517 → ↑1.5-fold	0.275 ± 0.90 ^t^ → ↓66%	65.9 ± 27.0	
		Fexo + Itraconazole ^i^		592 ± 185 → ↑1-fold	2 (1–4)	6.9 ± 2.0	4107 ± 1363 → ↑1.4-fold	0.280 ± 0.89 ^t^ → ↓66%	64.1 ± 24.6	
8	60	Fexo		332 ± 252	2 (0.5–4)	6.6 ± 3.1	1801 ± 979	0.75 ± 0.47 ^t^	55.6 ± 21.8	[80]
		Fexo + Itraconazole (Day 1)		709 ± 287 → ↑1.13-fold	2 (1–4)	4.9 ± 0.8	4108 ± 1429→↑1.3-fold	0.28 ± 0.14 ^t^ → ↓63%	50.4 ± 27.3	
		Fexo + Itraconazole (Day 3)		679 ± 167 → ↑1-fold	2(1.5–4)	4.6 ± 0.4	4231 ± 75 → ↑1.3-fold	0.24 ± 0.05 ^t^ → ↓68%	53.4 ± 24.3	
		Fexo + Itraconazole (Day 6)		595 ± 257 → ↑79%	2.5(1–4)	5.3 ± 0.8	3859 ± 1057 → ↑1-fold	0.28 ± 0.08 ^t^ → ↓63%	58.6 ± 23.9	
9	60	Fexo		194 ± 116	N/N	10.6 ± 1.9	1010 ± 460	0.93 ± 0.336 ^t^	N/N	[83]
		Fexo + Ritonavir ^j^		311 ± 102 → ↑60%	N/N	11.3 ± 2.5	2780 ± 920 → ↑1.75-fold	0.324 ± 0.114 ^t^ → ↓65%	N/N	
		Fexo + Ritonavir ^k^		191 ± 95	N/N	10.4 ± 3.9	1400 ± 700 → ↑39%	0.642 ± 0.39 ^t^ → ↓31%	N/N	
10	180	Fexo		390 (300–650)	2(1.4–3.0)	N/N	N/N	73.2 (50.50–123.20)^u^	5 (4.28–6.27) ^u^	[18]
		Fexo + Probenecid		510 (330–850) → ↑31%	2 (2.0–2.0)	N/N	N/N	42.11 (26.88–80.78) ^u^ → ↓42%	1.31 (1.03–2.63) ^u^	
11	60	Fexo		133 ± 67	N/N	11.1 ± 3.5	744 ± 282	1.368 ± 0.618 ^t^	N/N	[85]
		Fexo + Nelfinavir		100 ± 43 → ↓25%	N/N	8.2 ± 2.7	737 ± 192	1.296 ± 0.54 ^t^	N/N	
12	60	Fexo		164 ± 125	N/N	12.4 ± 2.2	859 ± 438	1.248 ± 0.498 ^t^	N/N	[87]
		Fexo + Ritonavir/Indinavir ^l^		404 ± 191 → ↑1.5-fold	N/N	8.3 ± 3.2	4160 ± 1510 → ↑3.8-fold	0.132 ± 0.24 ^t^ → ↓89%	N/N	
		Fexo + Ritonavir/Indinavir ^m^		464 ± 228 → ↑1.8-fold	N/N	7.5 ± 0.7	3540 ± 1530 → ↑3-fold	0.12 ± 0.396 ^t^ → ↓90%	N/N	
13	60	Fexo		176.6 ± 82.1	1.5 (1–4)	3.4 ± 0.7	1058.4 ± 528.7	67.3 ± 24.8	8.8 ± 3.5	[7]
		Fexo + Carbamazepine		103.2 ± 33.6 → ↓41%	1.5 (0.5–1.5)	3.0 ± 0.9	604.8 ± 255.9 → ↓43%	117.8 ± 55.5→↑75%	9.2 ± 6.9	
14	60	Fexo		304.4 ± 139.6	2.2 ± 1.1	4.7 ± 1.0	2159.2 ± 573.0	60.1 ± 19.7	N/N	[89]
		Fexo + Metronidazole		293.2 ± 137.7	2.4 ± 1.1	5.4 ± 2.8	2141.7 ± 538.2	59.7 ± 17.5	N/N	
15	60	Fexo		147.2 (63.3)	2 (1.0–4.0)	3.4 (0.83)	807.8 (338.5)	74.3 (29.9)	8.2 (3.4)	[93]
		Fexo + Fluvoxamine		231.0 (96.6) → ↑57%	2 (0.5–4.0)	3.3 (2.91)	1434.0 (666.1) → ↑77%	41.8 (24.0) → ↓44%	7.8 (6.3)	
		Fexo+ Paroxetine		195.0 (90.3) → ↑33%	1.5 (0.5–4.0)	4.8 (3.02)	1114.4 (626.6) → ↑38%	53.8 (30.4) → ↓28%	8.4 (6.6)	
		Fexo + Sertraline		127.1 (56.3) → ↓16%	1.25 (0.5–6.0)	2.5 (0.95)	675.6 (390.0) → ↓16%	88.8 (73.1) → ↑20%	10.0 (12.2)	
16	60	Fexo		134 ± 61	N/N	13.6 ± 2.7	729 ± 185	1.176 ± 0.336 ^t^	N/N	[94]
		Fexo + Indinavir		453 ± 298 → ↑2.4-fold	N/N	10.3 ± 3.6	2413 ± 1344 → ↑2.3-fold	0.438 ± 0.21 ^t^ → ↓63%	N/N	
17	25 ^n^	Fexo		0.11 ± 0.03	1.00 ± 0.55	5.75 ± 2.10	0.50 ± 0.17	N/N	N/N	[95]
		Fexo + Fluvoxamine/Ketoconazole		0.28 ± 0.15→↑1.5-fold	0.88 ± 0.14	13.78 ± 9.68	1.58 ± 0.46	N/N	N/N	
18	60	Fexo		172 ± 90	N/N	9.6 ± 4.8	1180 ± 620	0.864 ± 0.366 ^t^	N/N	[96]
		Fexo + Ritonavir/Lopinavir ^o^		440 ± 192 → ↑1.6-fold	N/N	7.8 ± 2.7	4420 ± 2180 → ↑2.7-fold	0.24 ± 0.114 ^t^ → ↓72%	N/N	
		Fexo + Ritonavir/Lopinavir ^p^		303 ± 175 → ↑76%	N/N	10.1 ± 3.0	2470 ± 1360	0.444 ± 0.228 ^t^→↓49%	N/N	
19	100 ^n^	Fexo		0.489 ± 0.183	1.13 ± 0.23	7.75 ± 1.59	N/N	28.6 ± 4.87	N/N	[99]
		Fexo + Ritonavir (2 mg)		0.761 ± 0.281 → ↑56%	1.13 ± 0.44	7.03 ± 1.43	N/N	22.0 ± 7.26→↓23%	N/N	
		Fexo + Ritonavir (100 mg)		1.250 ± 0.452 → ↑1.6-fold	1.31 ± 0.46	7.03 ± 0.88	N/N	13.8 ± 3.35→↓52%	N/N	
20	50	Fexo		0.195 ± 0.0840	1	N/N	N/N	N/N	N/N	[103]
		Fexo + Cremophor EL (1440 mg)		0.240 ± 0.0881 → ↑23%	1	N/N	N/N	N/N	N/N	
		Fexo + Cremophor EL (720 mg)		0.302 ± 0.123 ^i^ → ↑55%	1.5	N/N	N/N	N/N	N/N	
21	120	Fexo		169.8	1.9 (1.4)	5.2 (0.8)	1313.6	N/N	N/N	[106]
		Fexo + Vitamin D3		222.5 → ↑31%	2.1 (1.0)	4.9 (0.7)	1521.3	N/N	N/N	
22	25	Fexo		59.6 ± 27.0	1.60 ± 0.66	2.46 ± 0.30	270.4 ± 102.3	103.9 ± 33.7	N/N	[112]
		Fexo + Geneva Cocktail		32.9 ± 21.8 → ↓45%	2.01 ± 0.98	2.64 ± 0.55	165.7 ± 84.4 → ↓39%	189.1 ± 95.1 → ↑82%	N/N	
23	120	Fexo		710 ± 331	1.0 (0.5–2.5)	6.12 ± 5.76	3460.5 ± 2630.4	49.6 ± 26.3	N/N	[113]
		Fexo + Breviscapine		699 ± 321	1.0 (0.5–3.0)	6.06 ± 4.17	2972.5 ± 1965.3	51.3 ± 23.8	N/N	
Effect of Age and Gender										
24	60	Fexo ^a^		77 ± 31	2.167± 0.383	3.217 ± 0.533	N/N	177.3 ± 90.96 l	5.7 ± 3.06	[67]
		Fexo ^b^		72 ± 19	2.75 ± 0.39	3.25 ± 0.633	N/N	157.92 ± 59.76	8.4 ± 1.14	
		Fexo ^c^		106 ± 42	2.833 ± 0.55	3.833 ± 0.795	N/N	105.6 ± 42.5 l	5.82 ± 2.4	
		Fexo ^d^		76 ± 23	3.417± 0.667	3.017 ± 0.617	N/N	132.6 ± 33.24 l	6.06 ± 1.92	
		Fexo + Rifampicin ^a^		52 ± 17 → ↓32%	2.75 ± 0.25	3.033 ± 1.167	N/N	331.44 ± 204.6 → ↑1.5-fold	7.68 ± 3.78	
		Fexo + Rifampicin ^b^		36 ± 14 → ↓50%	2.917± 0.783	3.017 ± 1.433	N/N	425.46 ± 3227.4 → ↑3-fold	9.18 ± 4.32	
		Fexo + Rifampicin ^c^		52 ± 14 → ↓1-fold	2.583± 0.567	2.867 ± 0.556	N/N	276.48 ± 68.22 → ↑1-fold	7.74 ± 3	
		Fexo + Rifampicin ^d^		46 ± 19 → ↓39%	3 ± 0.733	2.5 ± 0.967	N/N	290.7 ± 96.0 → ↑1.2-fold	5.76 ± 1.92	
Enantiomers										
25	60	R (+)	Fexo	160 (75, 245) ^w^	1.5 (1–4)	3.9 (3.3, 4.5)	N/N	50 (32, 68)	4.6 (3.4, 5.7)	[84]
			Fexo + Itraconazole	290 (195, 384) → ↑81%	3.0 (1.5–4)	4.2 (3.7, 4.8)	N/N	17 (13, 21) → ↓66%	4.4 (3.3, 5.5)	
		S (−)	Fexo	111 (39, 182)	2.0 (1–4)	3.4 (2.7, 4.2)	N/N	95 (55, 135)	9.0 (6.4, 11.7)	
			Fexo + Itraconazole	236 (147, 326) → ↑1-fold	3.0 (1–4)	3.4 (3.0, 3.9)	N/N	25 (19, 31) → ↓74%	8.4 (6.1,10.8)	
26	120	R (+)	Fexo	223 (194, 252)	1.5 (0.5–4)	3.3 (2.8, 3.7)	1202 (1007,1396)	56 (47, 64)	N/N	[88]
			Fexo + Verapamil	480 (359, 600) → ↑54%	1.0 (1–4)	3.4 (2.8, 4.0)	2632 (2131, 3132) → ↑1.2-fold	26 (20, 32) → ↓53%	N/N	
		S (−)	Fexo	179 (149, 209)	1.8 (0.5–4)	3.0 (2.4, 3.7)	700 (577, 823)	93 (70, 116)	N/N	
			Fexo + Verapamil	392 (263, 520) → ↑1.2-fold	1.5 (1–4)	3.5 (2.8, 3.7)	2006 (1617, 2394) → ↑1.9-fold	33 (27, 39) → ↓65%	N/N	
27	60	R (+)	Fexo	132 (103, 161)	1.4 (1.1–1.7)	4.2 (3.4, 4.9)	749 (656, 842)	42 (36, 48)	4.7 (3.3, 6.0)	[16]
			Fexo + Carbamazepine	85 (64, 107) → ↓35%	1.1 (0.8–1.5)	3.3 (2.8, 3.9)	359 (303, 415) → ↓52%	86 (63, 108) → ↑1-fold	6.1(4.5, 7.7)	
		S (−)	Fexo	100 (83, 118)	1.5 (1.0–2.0)	3.7 (2.7, 4.7)	481 (410, 552)	67 (56, 77)	7.5 (5.5, 9.6)	
			Fexo + Carbamazepine	68 (47, 88) → ↓32%	1.1 (0.7–1.4)	2.5 (2.1, 2.8)	187 (153, 222) → ↓61%	174 (145, 202) → ↑1.6-fold	13.6 (10.0, 17.3)	
28	60	R (+)	Fexo	160 (117–202)	1.8 (0.5–4.0)	3.8 (3.3–4.4)	1011 (695–1327)	37 (28–46)	3.9 (2.5–5.4)	[19]
			Fexo + Rifampicin ^q^	502 (427–577) → ↑2-fold	2.0 (1.0–4.0)	3.5 (3.2–3.9)	3243 (2759–3727) → ↑2.2-fold	10 (8–12) → ↓79%	1.5 (1.1–2.0)	
			Fexo + Rifampicin ^r^	331 (261–401) → ↑1-fold	2.0 (1.0–4.0)	3.2 (2.7–3.6)	1922 (1635–2210) → ↑90%	16 (14–19) → ↓57%	1.8 (1.3–2.2)	
		S (−)	Fexo	127 (92–163)	1.8 (1.0–4.0)	3.3 (2.8–3.8)	698 (459–937)	56 (41–70)	6.6 (4.2–8.9)	
			Fexo + Rifampicin ^q^	443 (371–515) → ↑2.5-fold	1.8 (1.0–3.0)	3.1 (2.8–3.5)	2489 (2100–2877) → ↑2.6-fold	13 (10–17) → ↓77%	2.5 (1.6–3.4)	
			Fexo + Rifampicin ^r^	318 (252–385) → ↑1.5-fold	2.0 (1.0–4.0)	2.9 (2.5–3.3)	1674 (1400–1949) → ↑1.4-fold	19 (16–22) → ↓66%	3.0 (2.3–3.7)	
29	60	R (+)	Fexo	126 (100, 152)	1.4 (0.5–3.0)	4.1 (3.3, 5.0)	739 (638, 840) ^v^	43 (36, 49)	4.5 (3.0, 6.0)	[104]
			Fexo + Rifampicin	364 (300, 428) → ↑1.9-fold	1.8 (1.0–4.0)	3.5 (2.9, 4.0)	2205 (1386, 3023) ^v^ → ↑2-fold	17 (12, 22) → ↓60%	1.4 (1.0, 1.7)	
		S (−)	Fexo	104 (83, 125)	1.6 (1.0, 4.0)	3.4 (2.3, 4.5)	522 (366, 677) ^v^	67 (52, 82)	7.3 (4.5, 10.1)	
			Fexo + Rifampicin	326 (266, 387) → ↑2-fold	1.8 (1.0, 4.0)	2.8 (2.5, 3.0)	1563 (1069, 2057) ^v^ → ↑2-fold	23 (17, 30) → ↓66%	2.4 (1.6, 3.1)	
Genotypes										
30	180	Fexo (2677GG/3435CC)		510.8 ± 262.6	3.0 ± 1.6	5.0 ± 1.7	4040.4 ± 1832.2	0.8060 ± 0.3553 ^t^	0.0357 ± 0.030.0 ^t^	[72]
		Fexo (2677TT/3435TT)		713.8 ± 311.4	3.5 ± 2.0	5.0 ± 0.8	5194.0 ± 1910.8	0.5309 ± 0.1911 ^t^	0.0288 ± 0.0253 ^t^	
		Fexo + Itraconazole (2677GG/3435CC)		1376.3 ± 340.5 → ↑1.7-fold	3.4 ± 0.9	4.4 ± 0.6	9252.9 ± 2044.1 → ↑78%	0.2923 ± 0.0422 ^t^ → ↓64%	0.0258 ± 0.0155 ^t^	
		Fexo + Itraconazole (2677TT/3435TT)		2136.2 ± 897.9 → ↑2-fold	3.1 ± 1.5	4.6 ± 0.6	15,630.6 ± 5070.0 → ↑2-fold	0.167.0 ± 0.0333 ^t^ → ↓69%	0.0242 ± 0.0219 ^t^	
31	120	MDR1 C3435T genotype group								[113]
		Fexo (CC)		753 ± 408	1 (0.5–1.5)	7.52 ± 6.74	4365.3 ± 3958.1	47.4 ± 34.0	N/N	
		Fexo (CT)		675 ± 366	1 (0.5–2.0)	5.69 ± 2.54	3004.0 ± 2209.8	56.6 ± 29.7	N/N	
		Fexo (TT)		702 ± 263	1.75 (0.5–2.5)	4.01 ± 1.45	3012.3 ± 1225.1	44.8 ± 15.3	N/N	
		Fexo + Breviscapine (CC)		647 ± 288 → ↓14%	1.25 (0.5–1.5)	7.86 ± 6.85	3713.6 ± 3254.0 → ↓15%	49.6 ± 29.6	N/N	
		Fexo + Breviscapine (CT)		571 ± 376 → ↓15%	1.25 (0.5–3.0)	5.30 ± 2.01	2226.6 ± 943.5 → ↓26%	62.9 ± 26.8	N/N	
		Fexo + Breviscapine (TT)		879 ± 254 → ↑25%	1 (0.5–1.5)	5.02 ± 1.49	2977.3 ± 571.6	41.5 ± 7.4	N/N	

Fexo: Fexofenadine; Refs: Reference; R (+): R (+) enantiomer of fexofenadine; S (−): S (−) enantiomer of fexofenadine; N/N: Not Narrated; AUC_0–∞:_ Area under the curve from time 0 to infinity; CL/F: Oral Clearance; CL_R_: Renal clearance; C_max_: Maximum plasma concentration; t_1/2_: half-life; T_max_: Time to reach maximum plasma concentration; MDR1: Multi-drug resistance mutation 1; CC, CT, TT, GG: Alleles; 2677GG/3435CC: Genotype polymorphism; ^a^ Young men; ^b^ Young women; ^c^ Elderly men; ^d^ Elderly women; ^e^ Acute state of Lopinavir/ritonavir combination tablet; ^f^ Steady state of Lopinavir/ritonavir combination tablet; ^g^ 50 mg Itraconazole; ^h^ 100 mg Itraconazole; ^i^ 200 mg Itraconazole; ^j^ Acute ritonavir; ^k^ Steady state ritonavir; ^l^ Acute state of ritonavir/Indinavir combination tablet; ^m^ Steady state of ritonavir/Indinavir combination tablet; ^n^ μg; ^o^ Acute state of Ritonavir/Lopinavir combination tablet; ^p^ steady state of Ritonavir/Lopinavir combination tablet; ^q^ Single Dose; ^r^ Multiple Doses; ^s^ ng/mL.min; ^t^ L/h/kg; ^u^ L/h/1.73 m^2^; ^v^ AUC_0–24_: area under the curve from time 0 to 24 h; ↓ Relative decrease in a specific parameter; ↑ Relative increase in a specific parameter; ^w^ Data presented in mean and 95% confidence interval.

**Table 6:** Pharmacokinetic Parameters of DFI of fexofenadine.

Sr.	Fexo Dose (mg)	Treatment Groups		C_max_ (ng/mL)	T_max_ (h)	t_1/2_ (h)	AUC_0–∞_ (ng.h/mL)	CL/F (L/h)	Refs
1	60	Fexo + Water		201 (66)	2.28 (53)	11.0 (44)	N/N	N/N	[69]
		Fexo + GFJ		128 (35) → ↓36%	2.57 (27)	14.6 (58)	N/N	N/N	
2	60	Fexo + Water		288 ± 24	2.4 ± 0.3	2.6 ± 0.3	1617 ± 120	N/N	[70]
		Fexo + 25% GFJ		228 ± 28	2.6 ± 0.2	2.7 ± 0.2	1244 ± 111 → ↓23%	N/N	
		Fexo + GFJ		110 ± 14 → ↓62%	3.2 ± 0.4	3.1 ± 0.2	593 ± 67 → ↓63%	N/N	
		Fexo + OJ		96 ± 7 → ↓58%	2.7 ± 0.5	3.4 ± 0.3	494 ± 16 → ↓69%	N/N	
		Fexo + AJ		81 ± 13 → ↓64%	3.1 ± 0.5	3.5 ± 0.4	434 ± 53 → ↓73%	N/N	
3	120	Fexo + 300 mL Water		436 ± 74 ^a^	2.0 ± 0.4	3.0 ± 0.5	2167 ± 283	N/N	[75]
		Fexo + 1200 mL Water		326 ± 37	2.1 ± 0.3	3.2 ± 0.2	1747 ± 184	N/N	
		Fexo + 300 mL GFJ		233 ± 25 → ↓46%	3.3 ± 0.6	3.1 ± 0.2	491 ± 28 → ↓77%	N/N	
		Fexo + 1200 mL GFJ		109 ± 8 → ↓66%	2.9 ± 0.4	3.5 ± 0.2	677 ± 41 → ↓61%	N/N	
4	120	Fexo + Water ^a^		463 ± 62	2.3 ± 0.3	2.9 ± 0.2	2545 ± 327	N/N	[81]
		Fexo + GFJ ^a^		269 ± 47 → ↓42%	2.6 ± 0.3	2.9 ± 0.2	1465 ± 196 → ↓42%	N/N	
		Fexo + Naringin ^a^		380 ± 60 → ↓18%	1.8 ± 0.3	3.1 ± 0.3	1993 ± 278 → ↓22%	N/N	
		Fexo + Water ^b^		456 ± 62	1.8 ± 0.3	2.4 ± 0.2	2516 ± 373	N/N	
		Fexo + GFJ ^b^		276 ± 39 → ↓39%	2.8 ± 0.3	2.6 ± 0.2	1490 ± 140 → ↓41%	N/N	
		Fexo+ Furanocoumarin (0 h) ^b^		474 ± 49	2.4 ± 0.4	2.6 ± 0.2	2464 ± 243	N/N	
		Fexo + Furanocoumarin (−2 h) ^b^		433 ± 57	2.2 ± 0.4	2.2 ± 0.2	2379 ± 304	N/N	
5	120	Fexo + Water		0.57 (52.2)^c^	3 (1–6)	11.9 (36.5)	4.22 (40.0) ^d^	52.8 (40.0)	[97]
		Fexo + GFJ		0.45 (43.7) ^c^ → ↓21%	3.5 (1.5–5)	10.3 (37.6)	3.22 (33.5) ^d^ → ↓24%	69.3 (33.5) → ↑31%	
		Fexo + mGFJ		0.44 (32.4) ^c^	4 (2–6)	10.3 (29.7)	3.15 (28.6) ^d^ → ↓25%	70.9 (29.6) → ↑34%	
6	60	Fexo + Water		303 ± 132	2.0 (1.5–6.0)	N/N	1736 ± 462	0.6 ± 0.2 ^e^	[108]
		Fexo + 150 mL AJ		257 ± 119 → ↓15%	2.5 (1.5–6.0)	N/N	1598 ± 496	0.7 ± 0.1 ^e^ → ↑17%	
		Fexo + 300 mL AJ		158 ± 56 → ↓48%	3.0 (2.0–6.0)	N/N	1072 ± 429	1.1 ± 0.4 ^e^ → ↑83%	
		Fexo + 600 mL AJ		108 ± 31 → ↓64%	3.0 (3.0–6.0)	N/N	668 ± 163 → ↓61%	1.7 ± 0.3 ^e^ → ↑1.8-fold	
7	60	Fexo + Water		278.7	2	5.2	1765	N/N	[114]
		Fexo + GTE		82.6 → ↓70%	2	5.7	521.9 → ↓70%	N/N	
Enantiomers									
8	60	R (+)	Fexo + Water	131	1.5	3.8	774 ^g^	41	[101]
			Fexo + AJ	62 → ↓53%	2.9	3.8	364 ^g^ → ↓53%	95 → ↑1.3-fold	
		S (−)	Fexo + Water	110	1.6	3	530 ^g^	64	
			Fexo + AJ	41 → ↓63%	2.8	2.7	185 ^g^ → ↓65%	205 → ↑2.2-fold	
9	60	R (+)	Fexo + Water	131	1.4	4	777 ^g^	41	[105]
			Fexo + GFJ	68 → ↓48%	2	4.3	461 ^g^ → ↓41%	75 → ↑83%	
		S (−)	Fexo + Water	110	1.5	3.3	562 ^g^	62	
			Fexo + GFJ	45 → ↓59%	2.1	3.5	244 ^g^→↓57%	143 → ↑1.3-fold	
Genotypes									
10	60	SLCO2B1 c.1457C > T genotype							[91]
		Fexo	CC	343 ± 127 ^f^	1.5	3.2 ± 0.6	1762 ± 542	0.6 ± 0.2	
			CT	224 ± 139	1.5	3.0 ± 0.4	1088 ± 449	1.0 ± 0.4	
			TT	179 ± 42.0	1.8	3.9 ± 1.1	1136 ± 225	0.8 ± 0.2	
			CT + TT	204 ± 104	1.5	3.4 ± 0.9	1110 ± 347	0.9 ± 0.3	
		Fexo + AJ	CC	43.6 ± 9.8 → ↓87%	2.5	3.9 ± 1.1	263 ± 33.2 → ↓85%	3.7 ± 0.5 → ↑5-fold	
			CT	44.7 ± 16.4 → ↓80%	1.5	4.0 ± 1.1	253 ± 79.8 → ↓77%	3.9 ± 1.0 → ↑2.9-fold	
			TT	46.2 ± 18.6 → ↓74%	1.8	4.7 ± 0.9	352 ± 92.8 → ↓69%	2.6 ± 0.3 → ↑2.2 fold	
			CT + TT	45.4 ± 16.3 → ↓78%	1.5	4.3 ± 1.0	297 ± 95.8 → ↓73%	3.3 ± 1.0 → ↑2.66 fold	

Fexo: Fexofenadine; N/N: Not Narrated; Refs: Reference; AUC_0–∞_: Area under the curve from time 0 to infinity; CL/F: Oral Clearance; C_max_: Maximum plasma concentration; t_1/2_: half-life; T_max_: Time to reach maximum plasma concentration; SLCO2B1: Solute Carrier Organic Anion Transporter Family Member 2B1; CC, CT, TT: alleles; ^a^ Naringin-Fexofenadine group; ^b^ Furanocoumarin-Fexofenadine group; ^c^ µmol/L; ^d^ µmol.h/L; ^e^ L/h/kg; ^f^ values expressed as mean ± standard deviation (SD); ^g^ AUC_0–24_: Area under the curve from time 0 to 24 h; AJ: Apple Juice; GFJ: Grapefruit juice; GTE: Green Tea Extract; mGFJ: Modified Grapefruit juice; OJ: Orange Juice; **↓** Relative decrease in a specific parameter; ↑ Relative increase in a specific parameter.

**Table 7:** Pharmacokinetic Parameters of DHI of fexofenadine.

Sr.	Fexo Dose (mg)	Treatment Groups	C_max_ (ng/mL)	T_max_ (h)	t_1/2_ (h)	AUC_0–∞_ (ng.h/mL)	CL/F (L/h)	CL_R_ (L/h)	Refs
1	60	Fexo	163 ± 43	2.5 (2–3)	3.5 ± 0.9	965 ± 325	77 ± 23	3.3 ± 1.1	[68]
		Fexo + St John’s Wort ^a^	236 ± 96 → ↑45%	2.5 (1–3)	3.6 ± 1.6	1261 ± 507 → ↑31%	62 ± 26 → ↓19%	3.4 ± 1.7	
		Fexo + St John’s Wort ^b^	154 ± 75	3 (2–3)	3.7 ± 1.2	871 ± 447	91 ± 32 → ↑18%	3.2 ± 2.2	
2	180	Fexo	330 ± 144	N/N	4.8 ± 1.3	N/N	117.66 ± 43.56	N/N	[71]
		Fexo + St John’s Wort	202 ± 101 → ↓39%	N/N	5.3 ± 2.0	N/N	219.66 ± 115.56 → ↑87%	N/N	
3	120	Fexo	179	2.5	5.44	1121	107	N/N	[82]
		Fexo + Gingko Biloba Extract	175	2	5.98	925 → ↓18%	130	N/N	
4	60	Fexo	295.3 ± 135.4	2.0 (0.5–5)	4.5 ± 0.8	N/N	61.4 ± 18.4	4.72 ± 1.13	[86]
		Fexo + Quercetin	480.3 ± 163.7 → ↑63%	2 (1.5–3)	4.7 ± 1.5	N/N	38.7 ± 8.3 → ↓36%	4.29 ± 1.40	
5	120	Fexo	256	2	5.6	1569	0.076	N/N	[90]
		Fexo + Echinacea purpurea	232	2	5.5	1543	0.078	N/N	
6	120	Fexo	305	2.3	5.2	2036	0.059	N/N	[92]
		Fexo + Panax Ginseng	258 → ↓15%	2.8	4.8	1860	0.065	N/N	
7	120	Fexo	745.11 ± 137.41	2.25 ± 0.47	3.75 ± 1.47	3993.84 ± 912.97	N/N	N/N	[98]
		Fexo + Radix Astragali Extract	709.44 ± 170.03	2.21 ± 0.51	4.00 ± 1.24	3983.53 ± 1019.83	N/N	N/N	
8	120	Fexo	415.08 ± 67.63	2.37 ± 0.37	8.27 ± 1.82	2541.65 ± 527.18	49.46 ± 12.27	N/N	[100]
		Fexo + Resveratrol	685.58 ± 184.24 → ↑65%	2.41 ± 0.36	8.48 ± 2.15	4512.33 ± 1265.17 → ↑78%	28.37 ± 7.03 → ↓43%	N/N	
9	30	Fexo	71.851	2	4.899	467.806	N/N	N/N	[107]
		Fexo + Fermented red Ginseng	84.767 → ↑18%	2	4.725	611.566 → ↑30%	N/N	N/N	
10	60	Fexo	223.92 ± 74.36	3	5.97 ± 0.82	1717.66 ± 815.21	44.35 ± 25.57	N/N	[102]
		Fexo + Danshen Ethanol Extract	48.05 ± 60.93 → ↓78%	2	5.96 ± 1.00	889.99 ± 353.11 → ↓48%	77.88 ± 31.20 → ↑75%	N/N	
11	120	Fexo	523.28 ± 173.52	2.21 ± 1.03	9.26 ± 1.62	3507.80 ± 972.56	37.03 ± 11.67	6.41 ± 2.16	[109]
		Fexo + Diosmin	780.63 ± 150.41 → ↑49%	2.62 ± 0.98	9.45 ± 1.89	5815.76 ± 1430.72 → ↑66%	21.75 ± 5.04 → ↓41%	6.73 ± 3.02	
12	120	Fexo	406.9 ± 71.4	2.4 ± 0.4	12.1 ± 3.2	3571.1 ± 882.7	35.4 ± 8.1	6.2 ± 2.1	[110]
		Fexo + Piperine	767.0 ± 149.0 → ↑88%	2.4 ± 0.4	13.1 ± 1.8	6086.8 ± 1390.2 → ↑70%	20.7 ± 4.7 → ↓42%	6.5 ± 2.9	
13	10	Fexo	11.12	N/N	N/N	62.2	161	N/N	[111]
		Fexo + propolis extract	10.05	N/N	N/N	51 → ↓18%	196 → ↑22%	N/N	
Oral clearance (CL/F) across six different ethnic groups									
Sr.	PK	Treatment	African/American	Caucasian	Hispanic	Chinese	Indian	Malay	Refs
14	Cl/F (L/h)	Fexo	91 ± 47	108 ± 47	74 ± 26	75 ± 27	62 ± 17	55 ± 12	[73]
	Cl/F (L/h)	Fexo + St John’s Wort	158 ± 92 → ↑74%	195 ± 46	141 ± 58	112 ± 54 → ↑49%	86 ± 14 → ↑39%	98 ± 49 → ↑78%	

Fexo: Fexofenadine; N/N: Not Narrated; Refs: Reference; AUC_0–∞_: Area under the curve from time 0 to infinity; Cl/F: Oral Clearance; CL_R_: Renal clearance; C_max_: Maximum plasma concentration; t_1/2_: half-life; T_max_: Time to reach maximum plasma concentration; ^a^ Short dose administration; ^b^ Long dose administration; Relative decrease in a specific parameter; ↑ Relative increase in a specific parameter.

## Data Availability

All the data used for this publication are either presented in the main article or is available as Supplementary Information.

## Supplementary Materials

The following supporting information can be downloaded at https://www.mdpi.com/article/10.3390/pharmaceutics16121619/s1. Supplementary Table S1: PRISMA Checklist 2020. Supplementary Table S2: Screening of articles based on title, abstract, involvement of animals, and accessibility. Supplementary Table S3: JADAD Scoring. Supplementary Table S4: Critical Appraisal Skill Program (CASP). Supplementary Table S5: Critical Appraisal Clinical Pharmacokinetic (CACPK) Tool. Supplementary Table S6: Cochrane Collaboration Tool.

## References

[1] Dicpinigaitis P.V., Gayle Y.E. (2003). Effect of the second-generation antihistamine, fexofenadine, on cough reflex sensitivity and pul-monary function. Br. J. Clin. Pharmacol..

[2] Ciprandi G., Tosca M.A., Cosentino C., Riccio A.M., Passalacqua G., Canonica G.W. (2003). Effects of fexofenadine and other antihistamines on components of the allergic response: Adhesion molecules. J. Allergy Clin. Immunol..

[3] Handley D.A. (1999). Advancement of the Third Generation of Antihistamines. Pediatr. Asthma Allergy Immunol..

[4] Fischer J., Ganellin C.R. (2010). Analogue-based drug discovery. Chem. Int. Newsmag. IUPAC.

[5] Asha P.K., Raghu M.S., Devi V.S.A. (2020). Properties of Potassium Permanganate as Oxidant in the Determination of Fexofenadine in Pharmaceuticals. Sens. Lett..

[6] Barnett A.A. (1996). FDA approves safer form of terfenadine. Lancet.

[7] Yamada S., Yasui-Furukori N., Akamine Y., Kaneko S., Uno T. (2009). Effects of the P-glycoprotein inducer carbamazepine on fexofenadine phar-macokinetics. Ther. Drug Monit..

[8] Devillier P., Roche N., Faisy C. (2008). Clinical Pharmacokinetics and Pharmacodynamics of Desloratadine, Fexofenadine and Levocetirizine: A comparative review. Clin. Pharmacokinet..

[9] Axelrod D., Bielory L. (2008). Fexofenadine hydrochloride in the treatment of allergic disease: A review. J. Asthma Allergy.

[10] Team DSbHJ Fexofenadine. https://healthjade.net/fexofenadine/.

[11] Craun K.L., Schury M.P. (2024). Fexofenadine: StatPearls [Internet].

[12] Helmy S.A., El-Bedaiwy H.M., El-Masry S.M. (2020). Applying Biopharmaceutical Classification System Criteria to Predict the Potential Effect of Cremophor ^®^ RH 40 on Fexofenadine Bioavailability at Higher Doses. Ther. Deliv..

[13] Yamazaki A., Kumagai Y., Yamane N., Tozuka Z., Sugiyama Y., Fujita T., Yokota S., Maeda M. (2010). Microdose study of a P-glycoprotein substrate, fexofenadine, using a non-radioisotope-labelled drug and LC/MS/MS. J. Clin. Pharm. Ther..

[14] Simons F.E., Simons K.J. (1999). Clinical pharmacology of new histamine H1 receptor antagonists. Clin. Pharmacokinet..

[15] Molimard M., Diquet B., Benedetti M.S. (2004). Comparison of pharmacokinetics and metabolism of desloratadine, fexofenadine, levocetirizine and mizolastine in humans. Fundam. Clin. Pharmacol..

[16] Akamine Y., Miura M., Yasui-Furukori N., Kojima M., Uno T. (2012). Carbamazepine differentially affects the pharmacokinetics of fexofenadine enantiomers. Br. J. Clin. Pharmacol..

[17] Abilash K., Dinesh G., Janartanan S., Praveena J., Vanitha G., Gokul Manikandan P., Jeevanandham S. (2022). Formulation and evaluation of mouth dissolving films of fexofenadine hydrocloride by solvent casting method. World J. Pharm. Res..

[18] Liu S., Beringer P.M., Hidayat L., Rao A.P., Louie S., Burckart G.J., Shapiro B. (2008). Probenecid, but Not Cystic Fibrosis, Alters the Total and Renal Clearance of Fexofenadine. J. Clin. Pharmacol..

[19] Kusuhara H., Miura M., Yasui-Furukori N., Yoshida K., Akamine Y., Yokochi M., Fukizawa S., Ikejiri K., Kanamitsu K., Uno T. (2013). Effect of Coadministration of Single and Multiple Doses of Rifampicin on the Pharmacokinetics of Fexofenadine Enantiomers in Healthy Subjects. Drug Metab. Dispos..

[20] Valizadeh H., Leila B., Jalilian H., Islambulchilar Z., Zakeri-Milani P. (2009). Bioequivalence of Fexofenadine Tablet Formulations Assessed in Healthy Iranian Volunteers. Arzneimittelforschung.

[21] Lappin G., Shishikura Y., Jochemsen R., Weaver R.J., Gesson C., Houston B., Oosterhuis B., Bjerrum O.J., Rowland M., Garner C. (2010). Pharmacokinetics of fexofenadine: Evaluation of a microdose and assessment of absolute oral bioavailability. Eur. J. Pharm. Sci..

[22] Tannergren C., Petri N., Knutson L., Hedeland M., Bondesson U., Lennernäs H. (2003). Multiple transport mechanisms involved in the intestinal absorption and first-pass extraction of fexofenadine. Clin. Pharmacol. Ther..

[23] Kumar L., Alam M.S., Meena C.L., Jain R., Bansal A.K. (2009). Fexofenadine Hydrochloride. Profiles of Drug Substances, Excipients and Related Methodology.

[24] Pinto L., Moreira F.d.L., Nardotto G.H.B., Cavalli R.C., Moisés E.C.D., Duarte G., Lanchote V.L. (2020). Chiral Discrimination of P-glycoprotein in Parturient Women: Effect of Fluoxetine on Maternal-Fetal Fexofenadine Pharmacokinetics. Pharm. Res..

[25] Compalati E., Baena-Cagnani R., Penagos M., Badellino H., Braido F., Gómez R., Canonica G., Baena-Cagnani C. (2011). Systematic Review on the Efficacy of Fexofenadine in Seasonal Allergic Rhinitis: A Meta-Analysis of Randomized, Double-Blind, Placebo-Controlled Clinical Trials. Int. Arch. Allergy Immunol..

[26] Huang C.-Z., Jiang Z.-H., Wang J., Luo Y., Peng H. (2019). Antihistamine effects and safety of fexofenadine: A systematic review and Meta-analysis of randomized controlled trials. BMC Pharmacol. Toxicol..

[27] Mancano M.A. (2018). ISMP Adverse Drug Reactions: Propofol-Related Infusion Syndrome (PRIS) 1, 2; Ivermectin-Induced Ste-vens-Johnson Syndrome; Stevens-Johnson Syndrome/Toxic Epidermal Necrolysis From Fexofenadine; Memantine-Related Drug Eruption. Hosp. Pharm..

[28] Meltzer E.O., Rosario N.A., Van Bever H., Lucio L. (2021). Fexofenadine: Review of safety, efficacy and unmet needs in children with allergic rhinitis. Allergy, Asthma Clin. Immunol..

[29] Simpson K., Jarvis B. (2000). Fexofenadine: A review of its use in the management of seasonal allergic rhinitis and chronic idiopathic urticaria. Drugs.

[30] Smith S.M., Gums J.G. (2009). Fexofenadine: Biochemical, pharmacokinetic and pharmacodynamic properties and its unique role in allergic disorders. Expert. Opin. Drug Metab. Toxicol..

[31] Carnovale C., Battini V., Gringeri M., Volonté M., Uboldi M.C., Chiarenza A., Passalacqua G. (2022). Safety of fexofenadine and other second-generation oral antihistamines before and after the removal of the prescription requirement in Italy and other European countries: A real-world evidence study and systematic review. World Allergy Organ. J..

[32] Higgins J.P., Green S., Ben Van Den A. (2008). Cochrane Handbook for Systematic Reviews of Interventions.

[33] Moher D., Liberati A., Tetzlaff J., Altman D.G. (2010). Preferred reporting items for systematic reviews and meta-analyses: The PRISMA statement. Int. J. Surg..

[34] Clark H.D., Wells G.A., Huët C., McAlister F.A., Salmi L.R., Fergusson D., Laupacis A. (1999). Assessing the quality of randomized trials: Reliability of the Jadad scale. Control. Clin. Trials.

[35] Al-Dirini R.M.A., Thewlis D., Paul G. (2012). A Comprehensive Literature Review of the Pelvis and the Lower Extremity FE Human Models under Quasi-static Conditions. Work..

[36] Soliman A.B.E., Pawluk S.A., Wilby K.J., Rachid O. (2022). The use of a modified Delphi technique to develop a critical appraisal tool for clinical pharmacokinetic studies. Int. J. Clin. Pharm. Weekbl..

[37] Higgins J.P.T., Altman D.G., Gøtzsche P.C., Jüni P., Moher D., Oxman A.D., Savović J., Schulz K.F., Weeks L., Sterne J.A.C. (2011). The Cochrane Collaboration’s tool for assessing risk of bias in randomised trials. BMJ.

[38] Simons F.E.R., Bergman J.N., Watson W.T.A., Simons K. (1996). The clinical pharmacology of fexofenadine in children. J. Allergy Clin. Immunol..

[39] Russell T., Stoltz M., Weir S. (1998). Pharmacokinetics, pharmacodynamics, and tolerance of single- and multiple-dose fexofenadine hydrochloride in healthy male volunteers. Clin. Pharmacol. Ther..

[40] Robbins D.K., Castles M.A., Pack D.J., Bhargava V.O., Weir S.J. (1998). Dose proportionality and comparison of single and multiple dose pharmacokinetics of fexofenadine (MDL 16455) and its enantiomers in healthy male volunteers. Biopharm Drug Dispos.

[41] Drescher S., Schaeffeler E., Hitzl M., Hofmann U., Schwab M., Brinkmann U., Eichelbaum M., Fromm M.F. (2002). MDR1 gene polymorphisms and disposition of the P-glycoprotein substrate fexofena-dine. Br. J. Clin. Pharmacol..

[42] Hofmann U., Seiler M., Drescher S., Fromm M.F. (2002). Determination of fexofenadine in human plasma and urine by liquid chromatog-raphy-mass spectrometry. J. Chromatogr. B Anal. Technol. Biomed. Life Sci..

[43] Yi S.Y., Hong K.S., Lim H.S., Chung J.Y., Oh D.S., Kim J.R., Jung H.R., Cho J.Y., Yu K.S., Jang I.J. (2004). A variant 2677A allele of the MDR1 gene affects fexofenadine disposition. Clin Pharmacol Ther.

[44] Boyle J., Ridout F., Meadows R., Johnsen S., Hindmarch I. (2005). Suppression of the histamine-induced wheal and flare response by fexofenadine HCl 60 mg twice daily, loratadine 10 mg once daily and placebo in healthy Japanese volunteers. Curr. Med. Res. Opin..

[45] Mendoza L., Begany P., Dyrhonova M., Emritte N., Svobodova X. (2007). Bioequivalence of two fexofenadine formulations in healthy human volunteers after single oral administration. Biomed. Pap. Med. Fac. Univ. Palacky. Olomouc.

[46] Miura M., Uno T., Tateishi T., Suzuki T. (2007). Pharmacokinetics of fexofenadine enantiomers in healthy subjects. Chirality.

[47] Teng G., Teng L., Wu Y., Tang Y., Liu L., Gu J. (2007). Rapid and Sensitive LC-MS/MS Method for Quantification of Fexofenadine in Human Plas-ma—Application to a Bioequivalence Study in Chinese Volunteers. Chem. Res. Chin. Univ..

[48] Bharathi V.D., Radharani K., Jagadeesh B., Ramulu G., Bhushan I., Naidu A., Mullangi R. (2008). LC–MS–MS Assay for Simultaneous Quantification of Fexofenadine and Pseudoephedrine in Human Plasma. Chromatographia.

[49] Segall N., Grubbe R.E., Levy A.L., Maloney M.J., Nayak A.S., Kittner B., Quesada J.T. (2008). Pharmacokinetics, safety and tolerability of an oral suspension of fexofenadine for children with allergic rhinitis. Allergy Asthma Proc..

[50] Nolin T.D., Frye R.F., Le P., Sadr H., Naud J., Leblond F.A., Pichette V., Himmelfarb J. (2009). ESRD Impairs Nonrenal Clearance of Fexofenadine but not Midazolam. J. Am. Soc. Nephrol..

[51] Akamine Y., Miura M., Sunagawa S., Kagaya H., Yasui-Furukori N., Uno T. (2010). Influence of drug-transporter polymorphisms on the pharmacokinetics of fexofenadine enantiomers. Xenobiotica.

[52] Guo D., Zou J., Zhu Y., Lou S., Fan H., Qin Q. (2010). Measurement of fexofenadine concentration in micro-sample human plasma by a rapid and sensitive LC-MS/MS employing protein precipitation: Application to a clinical pharmacokinetic study. Biomed. Chromatogr..

[53] Muppavarapu R., Guttikar S., Rajappan M., Kamarajan K., Mullangi R. (2014). Sensitive LC-MS/MS-ESI method for simultaneous determination of montelukast and fexofenadine in human plasma: Application to a bioequivalence study. Biomed. Chromatogr..

[54] Chen Y.-A., Hsu K.-Y. (2014). Pharmacokinetics of fexofenadine in healthy Taiwanese volunteers. Pak. J. Pharm. Sci..

[55] Joy M.S., Frye R.F., Nolin T.D., Roberts B.V., La M.K., Wang J., Brouwer K.L., Dooley M.A., Falk R.J. (2014). In Vivo Alterations in Drug Metabolism and Transport Pathways in Patients with Chronic Kidney Diseases. Pharmacother. J. Hum. Pharmacol. Drug Ther..

[56] Yehia S.A., El-Ridi M.S., Tadros M.I., El-Sherif N.G. (2015). Phenylalanine-free taste-masked orodispersible tablets of fexofenadine hydrochloride: Development, in vitro evaluation and in vivo estimation of the drug pharmacokinetics in healthy human volunteers. Pharm. Dev. Technol..

[57] Thomson B.K., Nolin T.D., Velenosi T.J., Feere D.A., Knauer M.J., Asher L.J., House A.A., Urquhart B.L. (2015). Effect of CKD and Dialysis Modality on Exposure to Drugs Cleared by Nonrenal Mechanisms. Am. J. Kidney Dis..

[58] Helmy S.A., El Bedaiwy H.M. (2016). HPLC Determination of Fexofenadine in Human Plasma for Therapeutic Drug Monitoring and Pharmacokinetic Studies. Biomed. Chromatogr..

[59] Calvo E., Lee J., Kim S., Moreno V., Carpeno J.D., Weilert D., Laus G., Mann H., Vishwanathan K. (2019). Modulation of Fexofenadine Pharmacokinetics by Osimertinib in Patients with Advanced EGFR-Mutated Non–Small Cell Lung Cancer. J. Clin. Pharmacol..

[60] Cusinato D.A.C., Filgueira G.C.d.O., Rocha A., Cintra M.A.C., Lanchote V.L., Coelho E.B. (2019). LC-MS/MS analysis of the plasma concentrations of a cocktail of 5 cytochrome P450 and P-glycoprotein probe substrates and their metabolites using subtherapeutic doses. J. Pharm. Biomed. Anal..

[61] Pinto L.S.R., Vale G.T.D., Moreira F.d.L., Marques M.P., Coelho E.B., Cavalli R.C., Lanchote V.L. (2020). Direct chiral LC-MS/MS analysis of fexofenadine enantiomers in plasma and urine with application in a maternal-fetal pharmacokinetic study. J. Chromatogr. B.

[62] Egeland E.J., Witczak B.J., Zaré H.K., Christensen H., Åsberg A., Robertsen I. (2020). Chronic Inhibition of CYP3A is Temporarily Reduced by Each Hemodialysis Session in Patients with End-Stage Renal Disease. Clin. Pharmacol. Ther..

[63] Everardo P.G., Magdalena G.S., Elena G.P.M., Vanessa C.M., Gabriela S.C. (2021). Bioavailability assessment of fexofenadine and montelukast in a fixed-dose combination tablet versus the components administered simultaneously. Allergol. Immunopathol..

[64] Rauch C., Lucio L., De Fer B.B., Lheritier-Barrand M. (2023). Bioequivalence of 2 Pediatric Formulations of Fexofenadine Hydrochloride Oral Suspension. Clin. Pharmacol. Drug Dev..

[65] Chretien M.L., Bailey D.G., Asher L., Parfitt J., Driman D., Gregor J., Dresser G.K. (2023). Severity of coeliac disease and clinical management study when using a non-metabolised medication: A phase I pharmacokinetic study. BMJ Open.

[66] Gupta S., Banfield C., Kantesaria B., Marino M., Clement R., Affrime M., Batra V. (2001). Pharmacokinetic and safety profile of desloratadine and fexofenadine when coad-ministered with azithromycin: A randomized, placebo-controlled, parallel-group study. Clin. Ther..

[67] Hamman M.A., Bruce M.A., Haehner-Daniels B.D., Hall S.D. (2001). The effect of rifampin administration on the disposition of fexofenadine. Clin. Pharmacol. Ther..

[68] Wang Z., Hamman M.A., Huang S.M., Lesko L.J., Hall S.D. (2002). Effect of St John’s wort on the pharmacokinetics of fexofenadine. Clin. Pharmacol. Ther..

[69] Banfield C., Gupta S., Marino M., Lim J., Affrime M. (2002). Grapefruit Juice Reduces the Oral Bioavailability of Fexofenadine But Not Desloratadine. Clin. Pharmacokinet..

[70] Dresser G.K., Bailey D.G., Leake B.F., Schwarz U.I., Dawson P.A., Freeman D.J., Kim R.B. (2002). Fruit juices inhibit organic anion transporting polypeptide–mediated drug uptake to decrease the oral availability of fexofenadine. Clin. Pharmacol. Ther..

[71] Dresser G.K., Schwarz U.I., Wilkinson G.R., Kim R.B. (2003). Coordinate induction of both cytochrome P4503A and MDR1 by St John’s wort in healthy subjects. Clin. Pharmacol. Ther..

[72] Shon J.H., Yoon Y.R., Hongm W.S., Nguyen P.M., Lee S.S., Choi Y.G., Cha I.J., Shin J.G. (2005). Effect of itraconazole on the pharmacokinetics and pharmacodynamics of fexofenadine in relation to the MDR1 genetic polymorphism. Clin. Pharmacol. Ther..

[73] Xie R., Tan L.H., Polasek E.C., Hong C., Teillol-Foo M., Gordi T., Sharma A., Nickens D.J., Arakawa T., Knuth D.W. (2005). CYP3A and P-glycoprotein activity induction with St. John’s Wort in healthy volunteers from 6 ethnic populations. J. Clin. Pharmacol..

[74] Yasuifurukori N., Uno T., Sugawara K., Tateishi T. (2005). Different effects of three transporting inhibitors, verapamil, cimetidine, and probenecid, on fexofenadine pharmacokinetics. Clin. Pharmacol. Ther..

[75] Dresser G.K., Kim R.B., Bailey D.G. (2005). Effect of Grapefruit Juice Volume on the Reduction of Fexofenadine Bioavailability: Possible Role of Organic Anion Transporting Polypeptides*. Clin. Pharmacol. Ther..

[76] Rolf P.G., Heeswijk v., Bourbeau M., Campbell P., Seguin I., Chauhan B.M., Foster B.C., Cameron D.W. (2006). Time-Dependent Interaction Between Lopinavir/Ritonavir and Fexofenadine. J. Clin. Pharmacol..

[77] Lemma G.L., Wang Z., Hamman M.A., Zaheer N.A., Gorski J.C., Hall S.D. (2006). The effect of short- and long-term administration of verapamil on the disposition of cytochrome P450 3A and P-glycoprotein substrates. Clin. Pharmacol. Ther..

[78] Shimizu M., Uno T., Sugawara K., Tateishi T. (2006). Effects of itraconazole and diltiazem on the pharmacokinetics of fexofenadine, a substrate of P-glycoprotein. Br. J. Clin. Pharmacol..

[79] Uno T., Shimizu M., Sugawara K., Tateishi T. (2006). Lack of Dose-Dependent Effects of Itraconazole on the Pharmacokinetic Interaction with Fexofenadine. Drug Metab. Dispos..

[80] Shimizu M., Uno T., Sugawara K., Tateishi T. (2006). Effects of single and multiple doses of itraconazole on the pharmacokinetics of fexofen-adine, a substrate of P-glycoprotein. Br. J. Clin. Pharmacol..

[81] Bailey D.G., Dresser G.K., Leake B.F., Kim R.B. (2007). Naringin is a major and selective clinical inhibitor of organic anion-transporting poly-peptide 1A2 (OATP1A2) in grapefruit juice. Clin. Pharmacol. Ther..

[82] Robertson S.M., Davey R.T., Voell J., Formentini E., Alfaro R.M., Penzak S.R. (2008). Effect of Ginkgo biloba extract on lopinavir, midazolam and fexofenadine pharma-cokinetics in healthy subjects. Curr. Med. Res. Opin..

[83] Kharasch E.D., Bedynek P.S., Walker A., Whittington D., Hoffer C. (2008). Mechanism of ritonavir changes in methadone pharmacokinetics and pharmaco-dynamics: II. Ritonavir effects on CYP3A and P-glycoprotein activities. Clin. Pharmacol. Ther..

[84] Tateishi T., Miura M., Suzuki T., Uno T. (2008). The different effects of itraconazole on the pharmacokinetics of fexofenadine enantiomers. Br. J. Clin. Pharmacol..

[85] Kharasch E.D., Walker A., Whittington D., Hoffer C., Bedynek P.S. (2009). Methadone metabolism and clearance are induced by nelfinavir despite inhibition of cytochrome P4503A (CYP3A) activity. Drug Alcohol. Depend..

[86] Kim K.-A., Park P.-W., Park J.-Y. (2009). Short-term effect of quercetin on the pharmacokinetics of fexofenadine, a substrate of P-glycoprotein, in healthy volunteers. Eur. J. Clin. Pharmacol..

[87] Kharasch E.D., Hoffer C., Whittington D., Walker A., Bedynek P.S. (2009). Methadone pharmacokinetics are independent of cytochrome P4503A (CYP3A) activity and gastrointestinal drug transport: Insights from methadone interactions with ritonavir/indinavir. Anesthesiology.

[88] Sakugawa T., Miura M., Hokama N., Suzuki T., Tateishi T., Uno T. (2009). Enantioselective disposition of fexofenadine with the P-glycoprotein inhibitor vera-pamil. Br. J. Clin. Pharmacol..

[89] Kim K.A., Park J.Y. (2010). Effect of metronidazole on the pharmacokinetics of fexofenadine, a P-glycoprotein substrate, in healthy male volunteers. Eur. J. Clin. Pharmacol..

[90] Penzak S.R., Robertson S.M., Hunt J.D., Chairez C., Malati C.Y., Alfaro R.M., Stevenson J.M., Kovacs J.A. (2010). Echinacea purpurea significantly induces cytochrome P450 3A activity but does not alter lopinavir-ritonavir exposure in healthy subjects. Pharmacotherapy.

[91] Imanaga J., Kotegawa T., Imai H., Tsutsumi K., Yoshizato T., Ohyama T., Shirasaka Y., Tamai I., Tateishi T., Ohashi K. (2011). The effects of the SLCO2B1 c.1457C > T polymorphism and apple juice on the pharma-cokinetics of fexofenadine and midazolam in humans. Pharmacogenet Genom..

[92] Malati C.Y., Robertson S.M., Hunt J.D., Chairez C., Alfaro R.M., Kovacs J.A., Penzak S.R. (2012). Influence of *Panax ginseng* on Cytochrome P450 (CYP)3A and P-glycoprotein (P-gp) Activity in Healthy Participants. J. Clin. Pharmacol..

[93] Saruwatari J., Yasui-Furukori N., Niioka T., Akamine Y., Takashima A., Kaneko S., Uno T. (2012). Different Effects of the Selective Serotonin Reuptake Inhibitors Fluvoxamine, Paroxetine, and Sertraline on the Pharmacokinetics of Fexofenadine in Healthy Volunteers. J. Clin. Psychopharmacol..

[94] Kharasch E.D., Bedynek P.S., Hoffer C., Walker A., Whittington D. (2012). Lack of Indinavir Effects on Methadone Disposition Despite Inhibition of Hepatic and Intestinal Cytochrome P4503A (CYP3A). Anesthesiology.

[95] Croft M., Keely B., Morris I., Tann L., Lappin G. (2012). Predicting Drug Candidate Victims of Drug-Drug Interactions, using Microdosing. Clin. Pharmacokinet..

[96] Kharasch E.D., Stubbert K. (2013). *Cytochrome P*4503A Does Not Mediate the Interaction between Methadone and Ritonavir-Lopinavir. Drug Metab. Dispos..

[97] Won C.S., Lan T., VanderMolen K.M., Dawso P.A., Oberlies N.H., Widner W.W., Scarlett Y.V., Paine M.F. (2013). A Modified Grapefruit Juice Eliminates Two Compound Classes as Major Mediators of the Grapefruit Juice–Fexofenadine Interaction: An In Vitro–In Vivo “Connect”. J. Clin. Pharmacol..

[98] Zhou Q., Ye Z., Ruan Z., Zeng S. (2013). Investigation on modulation of human P-gp by multiple doses of Radix Astragali extract granules using fexofenadine as a phenotyping probe. J. Ethnopharmacol..

[99] Ieiri I., Tsunemitsu S., Maeda K., Ando Y., Izumi N., Kimura M., Yamane N., Okuzono T., Morishita M., Kotani N. (2013). Mechanisms of pharmacokinetic enhancement between ritonavir and saquinavir; mi-cro/small dosing tests using midazolam (CYP3A4), fexofenadine (p-glycoprotein), and pravastatin (OATP1B1) as probe drugs. J. Clin. Pharmacol..

[100] Bedada S.K., Yakkanti S.A., Neerati P. (2014). Resveratrol enhances the bioavailability of fexofenadine in healthy human male volunteers: Involvement of P-glycoprotein inhibition. J. Bioequiv Avail..

[101] Akamine Y., Miura M., Komori H., Saito S., Kusuhara H., Tamai I., Ieiri I., Uno T., Yasui-Furukori N. (2014). Effects of one-time apple juice ingestion on the pharmacokinetics of fexofenadine en-antiomers. Eur. J. Clin. Pharmacol..

[102] Qiu F., Zeng J., Liu S., He M., Zhu L., Ye Y., Miao P., Shen S., Jiang J. (2014). Effects of Danshen Ethanol Extract on the Pharmacokinetics of Fexofenadine in Healthy Volunteers. Evidence-Based Complement. Altern. Med..

[103] Tomaru A., Takeda-Morishita M., Maeda K., Banba H., Takayama K., Kumagai Y., Kusuhara H., Sugiyama Y. (2015). Effects of Cremophor EL on the absorption of orally administered saquinavir and fexofenadine in healthy subjects. Drug Metab. Pharmacokinet..

[104] Akamine Y., Miura M., Yasui-Furukori N., Ieiri I., Uno T. (2015). Effects of multiple-dose rifampicin 450 mg on the pharmacokinetics of fexof-enadine enantiomers in Japanese volunteers. J. Clin. Pharm. Ther..

[105] Akamine Y., Miura M., Komori H., Tamai I., Ieiri I., Yasui-Furukori N., Uno T. (2015). The change of pharmacokinetics of fexofenadine enantiomers through the single and simultaneous grapefruit juice ingestion. Drug Metab. Pharmacokinet..

[106] Kullak-Ublick G.A., Gubler C., Spanaus K., Ismair M.G., da Silva T.C., Jetter A. (2016). No major effects of vitamin D3 (1,25 dihydroxyvitamin D3) on absorption and pharmacokinetics of folic acid and fexofenadine in healthy volunteers. Eur. J. Clin. Pharmacol..

[107] Kim M.G., Kim Y., Jeon J.Y., Kim D.S. (2016). Effect of fermented red ginseng on cytochrome P450 and P-glycoprotein activity in healthy subjects, as evaluated using the cocktail approach. Br. J. Clin. Pharmacol..

[108] Luo J., Imai H., Ohyama T., Hashimoto S., Hasunuma T., Inoue Y., Kotegawa T., Ohashi K., Uemura N. (2016). The Pharmacokinetic Exposure to Fexofenadine is Volume-Dependently Reduced in Healthy Subjects Following Oral Administration With Apple Juice. Clin. Transl. Sci..

[109] Bedada S.K., Boga P.K., Kotakonda H.K. (2017). The effect of diosmin on the pharmacokinetics of fexofenadine in healthy human vol-unteers. Xenobiotica.

[110] Bedada S.K., Boga P.K. (2017). The influence of piperine on the pharmacokinetics of fexofenadine, a P-glycoprotein substrate, in healthy volunteers. Eur. J. Clin. Pharmacol..

[111] Cusinato D.A., Martinez E.Z., Cintra M.T., Filgueira G.C., Berretta A.A., Lanchote V.L., Coelho E.B. (2019). Evaluation of potential herbal-drug interactions of a standardized propolis extract (EPP-AF^®^) using an in vivo cocktail approach. J. Ethnopharmacol..

[112] Bosilkovska M., Magliocco G., Desmeules J., Samer C., Daali Y. (2019). Interaction between Fexofenadine and CYP Phenotyping Probe Drugs in Geneva Cocktail. J. Pers. Med..

[113] Zhao Y., Miao Z., Jiang M., Zhou X., Lai Y. (2021). Effects of breviscapine and C3435T MDR1 gene polymorphism on the pharmacokinetics of fexofenadine, a P-glycoprotein substrate, in healthy volunteers. Xenobiotica.

[114] Misaka S., Ono Y., Taudte R.V., Hoier E., Ogata H., Ono T., König J., Watanabe H., Fromm M.F., Shimomura K. (2022). Exposure of Fexofenadine, but Not Pseudoephedrine, Is Markedly Decreased by Green Tea Extract in Healthy Volunteers. Clin. Pharmacol. Ther..

