# Supplementary material for: Clinical Pharmacokinetics of Fexofenadine: A Systematic Review
Source: Pharmaceutics. 2024 Dec 20;16(12):1619. doi: 10.3390/pharmaceutics16121619 (PMC11677975; doi:10.3390/pharmaceutics16121619)
Supplement: Supplementary file 1 [file pharmaceutics-16-01619-s001.zip › pharmaceutics-3337002-supplementary/pharmaceutics-3337002-supplementary.pdf]

Clinical Pharmacokinetics of Fexofenadine: A Systematic Review

Maryam Batool[1], Ammara Zamir[1], Faleh Alqahtani[2*], Tanveer Ahmad[3], Hamid Saeed[4] & Muhammad Fawad Rasool[1*]

[1]Department of Pharmacy Practice, Faculty of Pharmacy, Bahauddin Zakariya University, 60800, Multan, Pakistan; maryambatool1818@gmail.com (M.B.); ammarazamir20@gmail.com (A.Z.); fawadrasool@bzu.edu.pk (M.F.R.)

[2]Department of Pharmacology and Toxicology, College of Pharmacy, King Saud University, Riyadh 11451, Saudi Arabia; afaleh@ksu.edu.sa (F.A.)

[3]Institute for Advanced Biosciences (IAB), CNRS UMR5309, INSERM U1209, Grenoble Alpes University, La Tronche, 38700, France; tanveer.ahmad@univ-grenoble-alpes.fr (T.A.)

[4]Section of Pharmaceutics, University College of Pharmacy, Allama Iqbal Campus, University of the Punjab, Lahore 54000, Pakistan; Section of Pharmaceutics, University College of Pharmacy, Allama Iqbal Campus, University of the Punjab, Lahore 54000, Pakistan; hamid.pharmacy@pu.edu.pk (H.S.)

*Corresponding author: fawadrasool@bzu.edu.pk (M.F.R.); afaleh@ksu.edu.sa (F.A.)

Telephone: +92 3008639046

Supplementary Table S1. PRISMA Checklist 2020

Section and Topic	Item #	Checklist item	Location where item is reported
**TITLE**			
Title	1	Identify the report as a systematic review.	1
**ABSTRACT**			
Abstract	2	See the PRISMA 2020 for Abstracts checklist.	1
**INTRODUCTION**			
Rationale	3	Describe the rationale for the review in the context of existing knowledge.	2
Objectives	4	Provide an explicit statement of the objective(s) or question(s) the review addresses.	2
**METHODS**			
Eligibility criteria	5	Specify the inclusion and exclusion criteria for the review and how studies were grouped for the syntheses.	3
Information sources	6	Specify all databases, registers, websites, organisations, reference lists and other sources searched or consulted to identify studies. Specify the date when each source was last searched or consulted.	3
Search strategy	7	Present the full search strategies for all databases, registers and websites, including any filters and limits used.	3
Selection process	8	Specify the methods used to decide whether a study met the inclusion criteria of the review, including how many reviewers screened each record and each report retrieved, whether they worked independently, and if applicable, details of automation tools used in the process.	4
Data collection process	9	Specify the methods used to collect data from reports, including how many reviewers collected data from each report, whether they worked independently, any processes for obtaining or confirming data from study investigators, and if applicable, details of automation tools used in the process.	4
Data items	10a	List and define all outcomes for which data were sought. Specify whether all results that were compatible with each outcome domain in each study were sought (e.g. for all measures, time points, analyses), and if not, the methods used to decide which results to collect.	4
	10b	List and define all other variables for which data were sought (e.g. participant and intervention characteristics, funding sources). Describe any assumptions made about any missing or unclear information.	4
Study risk of bias assessment	11	Specify the methods used to assess risk of bias in the included studies, including details of the tool(s) used, how many reviewers assessed each study and whether they worked independently, and if applicable, details of automation tools used in the process.	4
Effect	12	Specify for each outcome the effect measure(s) (e.g. risk ratio, mean difference) used in the synthesis or presentation of	4

Section and Topic	Item #	Checklist item	Location where item is reported
measures		results.	
Synthesis methods	13a	Describe the processes used to decide which studies were eligible for each synthesis (e.g. tabulating the study intervention characteristics and comparing against the planned groups for each synthesis (item #5)).	4
	13b	Describe any methods required to prepare the data for presentation or synthesis, such as handling of missing summary statistics, or data conversions.	4
	13c	Describe any methods used to tabulate or visually display results of individual studies and syntheses.	4
	13d	Describe any methods used to synthesize results and provide a rationale for the choice(s). If meta-analysis was performed, describe the model(s), method(s) to identify the presence and extent of statistical heterogeneity, and software package(s) used.	4
	13e	Describe any methods used to explore possible causes of heterogeneity among study results (e.g. subgroup analysis, meta-regression).	4
	13f	Describe any sensitivity analyses conducted to assess robustness of the synthesized results.	4
Reporting bias assessment	14	Describe any methods used to assess risk of bias due to missing results in a synthesis (arising from reporting biases).	4
Certainty assessment	15	Describe any methods used to assess certainty (or confidence) in the body of evidence for an outcome.	4
**RESULTS**			
Study selection	16a	Describe the results of the search and selection process, from the number of records identified in the search to the number of studies included in the review, ideally using a flow diagram.	5
	16b	Cite studies that might appear to meet the inclusion criteria, but which were excluded, and explain why they were excluded.	5
Study characteristics	17	Cite each included study and present its characteristics.	6,7,8,9
Risk of bias in studies	18	Present assessments of risk of bias for each included study.	6
Results of individual studies	19	For all outcomes, present, for each study: (a) summary statistics for each group (where appropriate) and (b) an effect estimates and it's precision (e.g. confidence/credible interval), ideally using structured tables or plots.	9,12,13,14,18, 19
Results of syntheses	20a	For each synthesis, briefly summarise the characteristics and risk of bias among contributing studies.	4,6

Section and Topic	Item #	Checklist item	Location where item is reported
	20b	Present results of all statistical syntheses conducted. If meta-analysis was done, present for each the summary estimate and its precision (e.g. confidence/credible interval) and measures of statistical heterogeneity. If comparing groups, describe the direction of the effect.	N/A
	20c	Present results of all investigations of possible causes of heterogeneity among study results.	N/A
	20d	Present results of all sensitivity analyses conducted to assess the robustness of the synthesized results.	N/A
Reporting biases	21	Present assessments of risk of bias due to missing results (arising from reporting biases) for each synthesis assessed.	6 (Supplementary Tables S3,S4,S5,S6)
Certainty of evidence	22	Present assessments of certainty (or confidence) in the body of evidence for each outcome assessed.	N/A
**DISCUSSION**			
Discussion	23a	Provide a general interpretation of the results in the context of other evidence.	21, 22, 23
	23b	Discuss any limitations of the evidence included in the review.	23
	23c	Discuss any limitations of the review processes used.	23
	23d	Discuss implications of the results for practice, policy, and future research.	23
**OTHER INFORMATION**			
Registration and protocol	24a	Provide registration information for the review, including register name and registration number, or state that the review was not registered.	N/A
	24b	Indicate where the review protocol can be accessed, or state that a protocol was not prepared.	N/A
	24c	Describe and explain any amendments to information provided at registration or in the protocol.	N/A
Support	25	Describe sources of financial or non-financial support for the review, and the role of the funders or sponsors in the review.	24
Competing interests	26	Declare any competing interests of review authors.	24
Availability of data, code and other materials	27	Report which of the following are publicly available and where they can be found: template data collection forms; data extracted from included studies; data used for all analyses; analytic code; any other materials used in the review.	24

Supplementary Table S2. Screening of articles based on title, abstract, involvement of animals, and accessibility

Sr #	Title of article	Exclusion basis
1	Allocco, F. T., V. Votypka, M. deTineo, R. M. Naclerio and F. M. Baroody (2002). "Effects of fexofenadine on the early response to nasal allergen challenge." Annals of allergy, asthma & immunology **89**(6): 578-584.	Abstract
2	Asahina, A., T. Etoh, A. Igarashi, S. Imafuku, H. Saeki, Y. Shibasaki, Y. Tomochika, S. Toyoizumi, M. Nagaoka and M. Ohtsuki (2016). "Oral tofacitinib efficacy, safety and tolerability in Japanese patients with moderate to severe plaque psoriasis and psoriatic arthritis: a randomized, double-blind, phase 3 study." Journal of dermatology. 43 (8) (pp 869-880), 2016. Date of publication: 01 aug 2016.	Title
3	Barrett-O'Keefe, Z., R. E. Kaplon and J. R. Halliwill (2013). "Sustained postexercise vasodilatation and histamine receptor activation following small muscle-mass exercise in humans." Experimental physiology **98**(1): 268-277.	Title
4	Bensch, G. W., H. S. Nelson and L. C. Borish (2002). "Evaluation of cytokines in nasal secretions after nasal antigen challenge: lack of influence of antihistamines." Annals of allergy, asthma & immunology **88**(5): 457-462.	Title
5	Blum, R. A., W. Kraft, J. A. Stewart, S. Meeves, G. Georges and S. J. Kovacs (2005). "Short-term safety, tolerability, and pharmacokinetics of desloratadine and fexofenadine in healthy adults identified as desloratadine slow metabolizers (DSMs)." Journal of allergy and clinical immunology **115**(2 Suppl): S127.	Full text
6	Bosilkovska, M., C. F. Samer, J. Deglon, A. Thomas, J. A. Desmeules and Y. Daali (2016). "Validation of the Geneva cocktail for cytochrome P450 phenotyping using innovative dried blood sampling method." Drug metabolism reviews **48**: 21.	Title
7	Botson, J., P. M. Peloso, K. Obermeyer, B. Lamoreaux, M. E. Weinblatt and J. Peterson (2020). "Pegloticase response improvement by co-treatment with methotrexate: results from the mirror open-label clinical trial in patients with uncontrolled gout." Annals of the rheumatic diseases **79**(SUPPL 1): 446.	Title
8	Bulan, K., M. Aydogan, R. Siraneci and C. Y. Aydogmus (2013). "The effect of montelukast on wheal reactions in skin prick tests: a double-blind-placebo-controlled randomized trial." International journal of pediatric otorhinolaryngology **77**(10): 1655-1658.	Title
9	Chi, C. T. (2012). "A randomised, placebo-controlled, two-period crossover pharmacokinetic study on modulation of human P-gp by multiple doses of Astragalus root extract granules using fexofenadine as a phenotyping probe." https://trialsearch.who.int/Trial2.aspx?TrialID=ChiCTR-TTRCC-12002288.	Title
10	Ellis, A. K., M. Murrieta-Aguttes, S. Furey and C. Carlsten (2020). "Efficacy of fexofenadine HCL 180 mg vs placebo in seasonal allergic rhinitis aggravated in presence of air pollutants: phase 3, sequential and parallel-group, double-blind, randomized study." Allergy **75**(SUPPL 109): 508.	Abstract

11	Ellis, A. K., M. Murrieta-Aguttes, S. Furey and C. Carlsten (2020). "Phase 3, single-center, sequential and parallel-group, double-blind, randomized study evaluating the efficacy and safety of Fexofenadine Hydrochloride 180 mg (Allegra®/Telfast®) versus placebo in subjects suffering from Allergic Rhinitis with symptoms aggravated in presence of pollutants: analysis of individual symptom scores." World Allergy Organization journal **13**(8).	Abstract
12	Ely, M. R., S. A. Romero, D. C. Sieck, J. E. Mangum, M. J. Luttrell and J. R. Halliwill (2017). "A single dose of histamine-receptor antagonists before downhill running alters markers of muscle damage and delayed-onset muscle soreness." Journal of applied physiology (Bethesda, Md. : 1985) **122**(3): 631-641.	Title
13	EU/EEA, E. O. (2017). "Safety, Efficacy and Pharmacokinetic Study of Allegra in Pediatric Patients With Atopic Dermatitis (AD)." https://trialsearch.who.int/Trial2.aspx?TrialID=EUCTR2017-000251-74-Outside-EU/EEA.	Not Accessible
14	EU/EEA, E. O. (2017). "Safety, Efficacy and Pharmacokinetic Study of Allegra in Pediatric Patients With Perennial Allergic Rhinitis (PAR)." https://trialsearch.who.int/Trial2.aspx?TrialID=EUCTR2017-000239-15-Outside-EU/EEA.	Not Accessible
15	Euctr, B. E. (2008). "CYP3A4/5 and PGP activity in renal transplantation. A study assessing in vivo hepatic and intestinal CYP3A4/5 and PGP activity in kidney transplant recipients and its relationship with genetic and non-genetic variables and the pharmacokinetics and metabolism of Tacrolimus." https://trialsearch.who.int/Trial2.aspx?TrialID=EUCTR2008-004158-33-BE.	Title
16	Glaeser, H., D. G. Bailey, G. K. Dresser, J. C. Gregor, U. I. Schwarz, J. S. McGrath, E. Jolicoeur, W. Lee, B. F. Leake, R. G. Tirona and et al. (2007). "Intestinal drug transporter expression and the impact of grapefruit juice in humans." Clinical pharmacology and therapeutics **81**(3): 362-370.	Title
17	Grant, J. A., L. Danielson, J. P. Rihoux and C. DeVos (1999). "A double-blind, single-dose, crossover comparison of cetirizine, ebastine, epinastine, fexofenadine, terfenadine, and loratadine versus placebo: suppression of histamine-induced wheal and flare response for 24 h in." Allergy **54**(7): 700-707.	Abstract
18	Grant, J. A., J. M. Riethuisen, B. Moulaert and C. DeVos (2002). "A double-blind, randomized, single-dose, crossover comparison of levocetirizine with ebastine, fexofenadine, loratadine, mizolastine, and placebo: suppression of histamine-induced wheal-and-flare response during 24 hours in healthy male subjects." Annals of allergy, asthma & immunology **88**(2): 190-197.	Abstract
19	Gupta, S., C. Banfield, J. Lim, M. Marino, R. P. Clement, M. B. Affrime and V. Batra (2001). "Unlike Fexofenadine, the pharmacokinetics of desloratadine are minimally altered by coadministration with azithromycin." Journal of allergy and clinical immunology **107**(2): S159.	Title
20	Hiraoka, K., M. Tashiro, T. Grobosch, M. Maurer, K. Oda, J. Toyohara, K. Ishii, K. Ishiwata and K. Yanai (2015). "Brain histamine H1 receptor occupancy measured by PET after oral administration of levocetirizine, a non-sedating antihistamine." Expert opinion on drug safety **14**(2): 199-206.	Title
21	Ieiri, I., Y. Doi, K. Maeda, T. Sasaki, M. Kimura, T. Hirota, T. Chiyoda, M. Miyagawa, S. Irie, K. Iwasaki and et al. (2012). "Microdosing clinical study: pharmacokinetic, pharmacogenomic (SLCO2B1), and interaction (grapefruit juice) profiles of celiprolol following the oral microdose and therapeutic dose." Journal of clinical pharmacology **52**(7): 1078-1089.	Title

22	Irct20180620040164N (2021). "bioequivalence study of Fexofenadine 180 mg tablets in 24 healthy male under fasting conditions." https://trialsearch.who.int/Trial2.aspx?TrialID=IRCT20180620040164N7.	Abstract
23	Irct20180620040164N (2023). "Comparative in vivo evaluation of 2 Fexofenadin 30 mg/5 ml Suspension formulations." https://trialsearch.who.int/Trial2.aspx?TrialID=IRCT20180620040164N42.	Abstract
24	Irct20200513047423N (2022). "Bioequivalance study of Fexofenadine 180 mg Tablets." https://trialsearch.who.int/Trial2.aspx?TrialID=IRCT20200513047423N6.	Abstract
25	JapicCti (2019). "A Study to Assess the Effect of S-600918 on the Pharmacokinetics of Fexofenadine, Rosuvastatin, and Atorvastatin in Healthy Adult Subjects." https://trialsearch.who.int/Trial2.aspx?TrialID=JPRN-JapicCTI-194757.	Not Accessible
26	Kaliner, M. A., M. V. White, A. Economides, T. Crisalida, M. Hale, Y. Liao, C. D. Christian, G. C. Georges, T. H. Woodworth and S. G. Meeves (2003). "Relative potency of fexofenadine HCl 180 mg, loratadine 10 mg, and placebo using a skin test model of wheal-and-flare suppression." Annals of allergy, asthma & immunology **90**(6): 629-634.	Abstract
27	Kaur, G., R. Dhingra and M. Singh (2017). "Assessment of clinical effectiveness of various drugs in the patients of allergic rhinitis visiting tertiary care hospital of Punjab, India." Clinical rhinology **10**(3): 132-136.	Title
28	Kharasch, E. D., A. Walker, C. Hoffer and P. Sheffels (2005). "Evaluation of first-pass cytochrome P4503A (CYP3A) and P-glycoprotein activities using alfentanil and fexofenadine in combination." Journal of clinical pharmacology **45**(1): 79-88.	Full text
29	Lennox, R. D. and M. J. Leahy (2004). "Validation of the Dermatology Life Quality Index as an outcome measure for urticaria-related quality of life." Annals of allergy, asthma & immunology **93**(2): 142-146.	Title
30	Magen, E., C. Yosefy, R. J. Viskoper and J. Mishal (2006). "Treatment of allergic rhinitis can improve blood pressure control." Journal of human hypertension **20**(11): 888-893.	Title
31	Mahatme, M. S., G. N. Dakhale, K. Tadke, S. K. Hiware, S. D. Dudhgaonkar and S. Wankhede (2016). "Comparison of efficacy, safety, and cost-effectiveness of montelukast-levocetirizine and montelukast-fexofenadine in patients of allergic rhinitis: a randomized, double-blind clinical trial." Indian journal of pharmacology **48**(6): 649-653.	Not Accessible
32	Mann, R. D. (2002). "Executive summary fexofenadine." Journal of drug evaluation: respiratory medicine **1**(1): 9-11.	Title

33	Mody, D., W. Yu, J. Lin and P. Ibrahim (2022). "Clinical Evaluation of Cudetaxestat for Safety,Tolerability, Pharmacokinetics, Pharmacodynamics, and Potential Drug-Drug Interactions." American journal of respiratory and critical care medicine **205**(1).	Title
34	Nct (2002). "Effects of Ginseng and Ginkgo on Drug Disposition in Man." https://clinicaltrials.gov/show/NCT00029692.	Title
35	Nct (2008). "Fexofenadine (Allegra®) in Healthy Adults Who Have Been Identified as Slow Metabolizers for Desloratadine." https://clinicaltrials.gov/show/NCT00636870.	Abstract
36	Nct (2012). "Safety Study of Levocetirizine and Fexofenadine." https://clinicaltrials.gov/show/NCT01586091.	Not Accessible
37	Nct (2013). "Dose Proportionality of Fexofenadine in Healthy Human Egyptian Volunteers." https://clinicaltrials.gov/show/NCT01767272.	Not Accessible
38	Nct (2014). "Clinical Trials on Evaluate the Red Ginseng and Fermented-Red Ginseng Affect to Drug Metabolizing Enzyme and Transporter in Healthy Volunteers." https://clinicaltrials.gov/show/NCT02056743.	Not Accessible
39	Nct (2015). "Evaluation of the Potential Pharmacokinetic Interactions Between Probe Drugs in the Geneva Phenotyping Cocktail." https://clinicaltrials.gov/show/NCT02391688.	Not Accessible
40	Nct (2017). "The Effect Dialysis on the Pharmacokinetics of Fexofenadine." https://clinicaltrials.gov/show/NCT03078777.	Abstract
41	Nct (2017). "Study to Evaluate the Pharmacokinetics and Pharmacodynamics of JMI-001." https://clinicaltrials.gov/show/NCT03183297.	Title
42	Nct (2017). "Oral Pharmacokinetics of Sulfasalazine, Paracetamol, Fexofenadine and Valsartan Using Different Administration Mediums." https://clinicaltrials.gov/show/NCT03012763.	Not Accessible
43	Nct (2020). "Excipient Effect on Drug Absorption in Humans." https://clinicaltrials.gov/show/NCT04534153.	Title
44	North, M. L., T. J. Walker, L. M. Steacy, B. G. Hobsbawn, R. J. Allan, F. Hackman, X. Sun, A. G. Day and A. K. Ellis (2014). "Add-on histamine receptor-3 antagonist for allergic rhinitis: a double blind randomized crossover trial using the environmental exposure unit." Allergy, asthma and clinical immunology **10**(1).	Abstract

45	Okubo, K., M. Gotoh, M. Asako, Y. Nomura, M. Togawa, A. Saito, T. Honda and Y. Ohashi (2017). "Efficacy and safety of bilastine in Japanese patients with perennial allergic rhinitis: a multicenter, randomized, double-blind, placebo-controlled, parallel-group phase III study." Allergology international **66**(1): 97-105.	Title
46	Okubo, K., M. Togawa, T. Honda and K. Hashiguchi (2016). "Efficacy of bilastine in Japanese cedar pollinosis: results of a randomized, double-blind, 4-way crossover, placebocontrolled, phase II study using an artificial exposure chamber (OHIO Chamber)." Allergy **71**: 384.	Title
47	Otsuka, H., K. Otsuka, S. Matsune and K. Okubo (2019). "Nasal symptoms reduction and decreased neutrophilia in japanese cedar pollinosis with prophylactic treatment with a combination of montelukast, fexofenadine, and fluticasone nasal spray." American journal of rhinology & allergy **33**(4): 369-377.	Abstract
48	Pawlowicz, R., K. Wytrychowski and B. Panaszek (2018). "Eradication of helicobacter pylori, as add-on therapy, has a significant, but temporary influence on recovery in chronic idiopathic urticaria: a placebo-controlled, double blind trial in the polish population." Postepy dermatologii i alergologii **35**(2): 151-155.	Abstract
49	Pellinger, T. K., B. R. Dumke and J. R. Halliwill (2013). "Effect of H1- and H2-histamine receptor blockade on postexercise insulin sensitivity." Physiological reports **1**(2).	Abstract
50	Sanchez, J., J. Zakzuk and R. Cardona (2016). "Clinical impact in quality of life of EAACI/GA2LEN/EDF/WAO guidelines for chronic spontaneous urticaria: evaluation step by step." Allergy **71**: 39.	Title
51	Sanofi, A. (2008). "A randomized, double-blind, repeat-dose, crossover study to evaluate the pharmacokinetics, safety, and tolerability of desloratadine (clarinex) compared to fexofenadine (allegra) in healthy adults who have been identified as slow metabolizers for desloratadine." Clinicaltrials.gov [accessed 31 jul 2008]: ClinicalTrials.gov ID: NCT00636870.	Not Accessible
52	Setiawati, A., M. S. S. Wiria, F. D. Suyatna, J. T. Sion and M. Hamadian (2005). "Interaction of erythromycin and clarithromycin with orange juice." Medical journal of indonesia **14**(2): 78-86.	Title
53	Sieck, D. C., J. E. Mangum, M. R. Ely, M. E. Francisco, E. A. Larson, B. W. Kaiser, C. T. Minson and J. R. Halliwill (2020). "Effect of Histamine-receptor Antagonism on VO2Peak Improvements to Exercise Training." FASEB journal **34**(SUPPL 1).	Abstract
54	Simons, F. E., N. A. Silver, X. Gu and K. J. Simons (2001). "Skin concentrations of H1-receptor antagonists." Journal of allergy and clinical immunology **107**(3): 526-530.	Abstract
55	Snidvongs, K., C. Rotjanasiriphong, C. Phannaso, S. Chusakul and S. Aeumjaturapat (2015). "Fexofenadine and levocetirizine have equivalent effectiveness for persistent allergic rhinitis." Asian biomedicine **9**(3): 387-395.	Short Communication

56	Takahashi, H., Y. Zhang and E. Morita (2008). "Evaluation of the antihistamine effects of olopatadine, cetirizine and fexofenadine during a 24 h period: a double-blind, randomized, crossover, placebo-controlled comparison in skin responses induced by histamine iontophoresis." Archives of dermatological research **300**(6): 291-295.	Abstract
57	Tanizaki, H., A. Ikoma, M. Fukuoka, Y. Miyachi and K. Kabashima (2012). "Effects of bepotastine and fexofenadine on histamine-induced flare, wheal and itch." International archives of allergy and immunology **158**(2): 191-195.	Short Communication
58	Tashiro, M., H. Mochizuki, K. Iwabuchi, Y. Sakurada, M. Itoh, T. Watanabe and K. Yanai (2002). "Roles of histamine in regulation of arousal and cognition: functional neuroimaging of histamine H1 receptors in human brain." Life sciences **72**(4-5): 409-414.	Abstract
59	Tctr (2018). "A Single Dose, Randomized, Open-label, Two-way Crossover Bioequivalence Study of Generic Fexofenadine Hydrochloride 180 mg Film-coated Tablets and Reference Product (TelfastÂ® 180mg) in Healthy Thai Volunteers under Fasting Conditions." https://trialsearch.who.int/Trial2.aspx?TrialID=TCTR20180517002.	Not Accessible
60	Tctr (2023). "A Bioequivalence study of a randomized, open-label, single dose, full replicate crossover design with four-period, two-treatment, and two-sequence of Fexofenadine 180 mg tablets relative to Telfast 180 mg tablets in healthy Thai volunteers under fasting condition." https://trialsearch.who.int/Trial2.aspx?TrialID=TCTR20230915004.	Not Accessible
61	Umin (2010). "Clinical research to evaluate the contribution of drug transporters and drug metabolizing enzymes for PI boosting by ritonavir." https://trialsearch.who.int/Trial2.aspx?TrialID=JPRN-UMIN000004370.	Title
62	Umin (2010). "Exploratory trials evaluating the pharmacokinetics of Quinidine and Verapamil after oral administration in healthy male subjects." https://trialsearch.who.int/Trial2.aspx?TrialID=JPRN-UMIN000004094.	Title
63	Umin (2013). "Effect of timing of apple juice intake on the pharmacokinetics of fexofenadine: an open-label, randomized study in healthy volunteers." https://trialsearch.who.int/Trial2.aspx?TrialID=JPRN-UMIN000011034.	Not Accessible
64	Umin (2014). "The effect of apple juice on the pharmacokinetics of fexofenadine -evaluation of dose-effect relationship." https://trialsearch.who.int/Trial2.aspx?TrialID=JPRN-UMIN000014773.	Not Accessible
65	Umin (2018). "Influence of green tea on pharmacokinetics of fexofenadine in healthy volunteers." https://trialsearch.who.int/Trial2.aspx?TrialID=JPRN-UMIN000032828.	Not Accessible
66	Wetter, T. J., Z. Xiang, D. A. Sonetti, H. C. Haverkamp, A. J. Rice, A. A. Abbasi, K. C. Meyer and J. A. Dempsey (2002). "Role of lung inflammatory mediators as a cause of exercise-induced arterial hypoxemia in young athletes." Journal of applied physiology (Bethesda, Md. : 1985) **93**(1): 116-126.	Title

67	Yakusheva, E. N., I. V. Chemykh, A. V. Shchulkin, M. V. Gatsanoga and A. A. Nikiforov (2019). "Influence of afobazole on the abcb1 protein functional activity in volunteers with low anxiety level." Eksperimental'naya i klinicheskaya farmakologiya **82**(3): 17-21.	Title
68	Yan, H., M. A. Behun, M. D. Cook, S. M. Ranadive, A. D. Lane-Cordova, R. M. Kappus, J. A. Woods, K. R. Wilund, T. Baynard, J. R. Halliwill and et al. (2016). "Differential post-exercise blood pressure responses between blacks and caucasians." Plos ONE. 11 (4) (no pagination), 2016. Article number: e0153445. Date of publication: april 2016.	Title
69	zmfs, R. B. R. (2013). "Evaluation of Green Propolis as a oral Anti-inflammatory Drug." https://trialsearch.who.int/Trial2.aspx?TrialID=RBR-9zmfs9.	Title
70	Abdelaziz, M. M., J. L. Devalia, O. A. Khair, H. Bayram, A. J. Prior and R. J. Davies (1998). "Effect of fexofenadine on eosinophil-induced changes in epithelial permeability and cytokine release from nasal epithelial cells of patients with seasonal allergic rhinitis." Journal of allergy and clinical immunology **101**(3): 410-420.	Abstract
71	Acee, A. M. and L. Sharoff (2012). "Herbal Remedies, Mood, and Cognition." Holistic Nursing Practice **26**(1): 38-51.	Title
72	ACUÑA, M., L. EATON and L. CIFUENTES (2006). "ALVES, VMN see SIMOES, JA ANDERSON, BJ see ALLEGAERT, K. ANTIKAINEN, M. see HEDMAN, M. ARONSON, JK A prescription for better prescribing: 487." Br J Clin Pharmacol **61**(6): 791-796.	Title
73	Agarwal, S. K., B. Tong, O. F. Bueno, R. M. Menon and A. H. Salem (2018). "Effect of azithromycin on venetoclax pharmacokinetics in healthy volunteers: implications for dosing venetoclax with P-gp inhibitors." Advances in Therapy **35**: 2015-2023.	Abstract
74	Ahmed, S., M. Nazmi, I. Hasan, S. Sultana, S. Haldar and M. S. Reza (2013). "Fexofenadine HCl immediate release tablets: in vitro characterization and evaluation of excipients." Bangladesh pharmaceutical journal **16**(1): 1-9.	Abstract
75	Akamine, Y. (2015). "Determinants of the stereoselective pharmacokinetics of fexofenadine." Yakugaku Zasshi: Journal of the Pharmaceutical Society of Japan **135**(3): 473-481.	Other language
76	Akamine, Y. and M. Miura (2018). "An update on the clinical pharmacokinetics of fexofenadine enantiomers." Expert Opinion on Drug Metabolism & Toxicology **14**(4): 429-434.	Title
77	Al-Jenoobi, F. I. (2010). "Effect of itraconazole on the pharmacokinetics of diclofenac in Beagle dogs." Scientia pharmaceutica **78**(3): 465-472.	Animal

78	Aleksi, T., M. Heli and M. Heli "Contact information, Principal Investigator."	Title
79	Allegra, A. O. "Search Knowledgebase Topic Contents."	Title
80	Allegra, A. O. "Section Title References."	Title
81	Alsaif, N. A., T. A. Wani, A. H. Bakheit and S. Zargar (2020). "Multi-spectroscopic investigation, molecular docking and molecular dynamic simulation of competitive interactions between flavonoids (quercetin and rutin) and sorafenib for binding to human serum albumin." International Journal of Biological Macromolecules **165**: 2451-2461.	Title
82	Amara, S. B., M. A. Ami, T. Koslowski and A. Zaidi (2023). "Quantum chemistry of cocaine and its isomers II: Spectroscopy." Computational and Theoretical Chemistry **1219**: 113952.	Title
83	Amer, A. A. E.-M. (2023). "A narrative review on clinical trials showing contraindicated drugs with grapefruit juice." Phytochemistry **2**(2): 48-69.	Title
84	ANDREWS, E., B. DAMLE, A. FANG, G. FOSTER and P. CROWNOVER (2008). "BÜRKI, F., BADIE, K., BARTOLI, P., BERNARD, P., MONTASTRUC, JL & BAGHERI, H. Reversible posterior leukoencephalopathy syndrome associated with."	Title
85	Arai, M., H. Komori, D. Fujita and I. Tamai (2021). "Uptake pathway of apple-derived nanoparticle by intestinal cells to deliver its cargo." Pharmaceutical Research **38**: 523-530.	Title
86	Armstrong, S. C. and K. L. Cozza (2003). "Antihistamines." Psychosomatics **44**(5): 430-434.	Title
87	Arold, G., F. Donath, A. Maurer, K. Diefenbach, S. Bauer, H.-H. Henneicke-von Zepelin, M. Friede and I. Roots (2005). "No relevant interaction with alprazolam, caffeine, tolbutamide, and digoxin by treatment with a low-hyperforin St John’s wort extract." Planta medica **71**(04): 331-337.	Title
88	Artero, A. P. and A. J. F. Villacampa "INTERACCIONES FÁRMACO/ALIMENTO DRUG/FOOD INTERACTIONS."	Other language
89	Asano, K., K.-i. Kanai and H. Suzaki (2004). "Suppressive activity of fexofenadine hydrochloride on thymus-and activation-regulated chemokine production from human peripheral blood leukocytes in response to antigenic stimulation in vitro." International archives of allergy and immunology **133**(3): 267-275.	Abstract

90	BACKMAN, J. and M. KARJALAINEN (2006). "BURGGRAAF, J. see RAD, M. BURGGRAAF, K. see VAN DOORN, M. BURTIN, B. see LARBIG, M." Br J Clin Pharmacol **62**(6): 741.	Title
91	Bailey, D. (2019). "Drug administration and grapefruit." Compendium of pharmaceuticals and specialties. Ottawa, Ontario, Canada: Canadian Pharmacists Association: L11-L46.	Title
92	Barrett, J. S. and A. F. Zuppa (2012). "Basic Principles of Clinical Pharmacology Relevant to Pharmacoepidemiologic Studies." Pharmacoepidemiology: 23-37.	Title
93	Bell, E. C., W. R. Ravis, K. B. Lloyd and T. J. Stokes (2007). "Effects of St. John's wort supplementation on ibuprofen pharmacokinetics." Annals of Pharmacotherapy **41**(2): 229-234.	Title
94	Bhadraiah, A., G. D. Reddy, K. Bharavi, V. R. Devi and P. R. Kumar (2023). "Albendazole encapsulation along with quercetin in chitosan-alginate microspheres enhances bioavailability and sustained release in broilers."	Animal
95	Bhoopathy, S., C. Bode, V. Naageshwaran, E. Weiskircher-Hildebrandt, V. Mukkavilli and I. J. Hidalgo (2021). "Case Study 7: Transporters Case Studies—In Vitro Solutions for Translatable Outcomes." Enzyme Kinetics in Drug Metabolism: Fundamentals and Applications: 709-735.	Title
96	Blaiss, M. S. (2005). Antihistamines: treatment selection criteria for pediatric seasonal allergic rhinitis. Allergy and asthma proceedings, OceanSide Publications.	Title
97	Boaz Amichai, M. "Fexofenadine Hydrochloride±A New Anti-Histaminic Drug."	Abstract
98	Boike, S., M. Pue and M. Freed (1994). "Famciclovir–Selected References." Clin Pharmacol Ther **55**: 18-26.	Title
99	Borrelli, F. and A. A. Izzo (2009). "Herb–drug interactions with St John’s wort (Hypericum perforatum): an update on clinical observations." The AAPS journal **11**: 710-727.	Title
100	Bosgra, S., M. L. Vlaming and W. H. Vaes (2016). "To apply microdosing or not? Recommendations to single out compounds with non-linear pharmacokinetics." Clinical pharmacokinetics **55**: 1-15.	Title

101	Bosilkovska, M., J. Déglon, C. Samer, B. Walder, J. Desmeules, C. Staub and Y. Daali (2014). "Simultaneous LC–MS/MS quantification of P-glycoprotein and cytochrome P450 probe substrates and their metabolites in DBS and plasma." Bioanalysis **6**(2): 151-164.	Abstract
102	Calderon-Ospina, C. A., M. Hernández-Sómerson, A. M. García, A. Mejia, C. Tamayo-Agudelo, P. Laissue and D. J. Fonseca Mendoza (2020). "A pharmacogenomic dissection of a rosuvastatin-induced rhabdomyolysis case evokes the polygenic nature of adverse drug reactions." Pharmacogenomics and Personalized Medicine: 59-70.	Title
103	Campos, M., G. Costa and O. Pelkonen (2010). "A huge number of mixtures and substances: How to deal with them?" Toxicology Letters **196**: S16-S17.	Title
104	Chae, K. M. and M. D. Tharp (2000). "Use and safety of antihistamines in children." Dermatologic Therapy **13**(4): 374-383.	Title
105	Chan, W.-J. J., J. Adiwidjaja, A. J. McLachlan, A. V. Boddy and J. E. Harnett (2023). "Interactions between natural products and cancer treatments: Underlying mechanisms and clinical importance." Cancer Chemotherapy and Pharmacology **91**(2): 103-119.	Title
106	Chaudhary, H., S. Saini and G. Singh (2012). "Formulation and evaluation of fexofenadine hydrochloride transdermal patch." Journal of Drug Delivery and Therapeutics **2**(5).	Abstract
107	Chen, J., Q. Su, J. Qin, Y. Zhou, H. Ruan, Z. Chen, Z. Chen, H. Li, Y. Zhou and S. Zhou (2019). "Correlation of MCT1 and ABCC2 gene polymorphisms with valproic acid resistance in patients with epilepsy on valproic acid monotherapy." Drug Metabolism and Pharmacokinetics **34**(3): 165-171.	Title
108	Chen, M., S. Hu, Y. Li, A. A. Gibson, Q. Fu, S. D. Baker and A. Sparreboom (2020). "Role of Oatp2b1 in drug absorption and drug-drug interactions." Drug Metabolism and Disposition **48**(5): 419-425.	Title
109	Chen, M., S.-y. Zhou, E. Fabriaga, P.-h. Zhang and Q. Zhou (2018). "Food-drug interactions precipitated by fruit juices other than grapefruit juice: An update review." Journal of food and drug analysis **26**(2): S61-S71.	Title
110	Choi, P. M., J. W. O'Brien, J. Li, G. Jiang, K. V. Thomas and J. F. Mueller (2018). "Population histamine burden assessed using wastewater-based epidemiology: The association of 1, 4-methylimidazole acetic acid and fexofenadine." Environment international **120**: 172-180.	Abstract
111	Chrubasik-Hausmann, S., J. Vlachojannis and A. J. McLachlan (2019). "Understanding drug interactions with St John's wort (Hypericum perforatum L.): impact of hyperforin content." Journal of Pharmacy and Pharmacology **71**(1): 129-138.	Title

112	Church, D. S. and M. K. Church (2011). "Pharmacology of antihistamines." World Allergy Organization Journal **4**(3): S22-S27.	Title
113	Church, M. K. (2021). "Antihistamines." Urticaria and Angioedema: 153-165.	Title
114	Conen, S., E. L. Theunissen, A. Vermeeren, P. van Ruitenbeek, P. Stiers, M. A. Mehta, S. W. Toennes and J. G. Ramaekers (2013). "The role of P-glycoprotein in CNS antihistamine effects." Psychopharmacology **229**: 9-19.	Title
115	Cooke, C. E., G. E. Sklar and J. M. Nappi (1996). "Possible pharmacokinetic interaction with quinidine: ciprofloxacin or metronidazole?" Annals of Pharmacotherapy **30**(4): 364-366.	Title
116	Cortés, M., R. Díaz, S. Verdugo, S. Cabrera and P. Torres-Vergara (2021). "Jugos de fruta y productos herbáceos como perpetradores de interacciones farmacocinéticas mediadas por enzimas metabolizadoras y transportadores de membrana. Relevancia en clínica." Revista médica de Chile **149**(7): 1036-1046.	Other language
117	Craun, K. L. and M. P. Schury "Continuing Education Activity."	Title
118	Craun, K. L. and M. P. Schury. (December 5, 2022). "Fexofenadine." StatPearls [Internet] Retrieved 23 April; 2024, 2024, from https://www.ncbi.nlm.nih.gov/books/NBK556104/#_article-21720_s11_.	Title
119	Crowe, A. (2015). "Case Studies of Non-CNS Drugs to Minimize Brain Penetration—Nonsedative Antihistamines." Blood-Brain Barrier in Drug Discovery: Optimizing Brain Exposure of CNS Drugs and Minimizing Brain Side Effects for Peripheral Drugs: 463-481.	Title
120	Cunha, S. V. P. d. (2019). Characterization of the interaction and permeation of drug-like molecules through membrane models including P-glycoprotein.	Title
121	Cusinato, D. A. C. (2017). Influência do EPP-AF® na atividade da glicoproteína P e do citocromo P450 em voluntários sadios usando coquetel de marcadores, Universidade de São Paulo.	Other language
122	Danielova Tsankova, A. "Interacciones del hipérico (Hypericum perforatum)."	Other language
123	Dasgupta, A. (2008). "Drug–Herb and Drug–Food Interactions: Impact on Therapeutic Drug Monitoring." Handbook of Drug Monitoring Methods: Therapeutics and Drugs of Abuse: 235-261.	Title

124	Daud, A. N., J. E. Bergman, M. P. Oktora, W. S. Kerstjens-Frederikse, H. Groen, J. H. Bos, E. Hak and B. Wilffert (2017). "Maternal use of drug substrates of placental transporters and the effect of transporter-mediated drug interactions on the risk of congenital anomalies." PloS one **12**(3): e0173530.	Title
125	Dávila, I., A. Del Cuvillo, J. Mullol, I. Jáuregui, J. Bartra, M. Ferrer, J. Montoro, J. Sastre and A. Valero (2013). "Use of Second Generation H." J Investig Allergol Clin Immunol **23**(1): 1-16.	Title
126	Di Gioia, S., M. N. Hossain and M. Conese (2020). "Biological properties and therapeutic effects of plant-derived nanovesicles." Open Medicine **15**(1): 1096-1122.	Title
127	Di, Y. M., C. G. Li, C. C. Xue and S.-F. Zhou (2008). "Clinical Drugs that Interact with St. John's Wort and mplication in Drug Development." Current pharmaceutical design **14**(17): 1723-1742.	Title
128	do Nascimento, S. B., M. de Lima Nascimento, L. L. de Araújo, F. M. de Oliveira, M. do Carmo Vieira, J. M. Duarte-Almeida, J. M. Siqueira, I. da Costa César, H. Derendorf and W. V. de Castro (2020). "Evaluation of the effects of Maytenus ilicifolia on the activities of cytochrome P450 3A and P-glycoprotein." Current Drug Metabolism **21**(4): 281-290.	Title
129	Dostalek, M. and A. K. Stark (2012). "St John's Wort (Hypericum Perforatum L.)." Metabolism of Drugs and Other Xenobiotics: 583-610.	Title
130	DuBuske, L. and K. Kowal (2009). "Update on prescription and over-the-counter histamine inverse agonists in rhinitis therapy." Current allergy and asthma reports **9**(2): 140-148.	Title
131	Elmghari, M., H. Benjelloun, N. Zaghba, A. Bakhatar, N. Yassine and A. Bahlaoui (2015). "Les atteintes respiratoires au cours de la polyarthrite rhumatoïde." Revue des Maladies Respiratoires **32**: A186-A187.	Other language
132	Erhunse, N., E. S. Omoregie and D. Sahal (2023). "Antiplasmodial and antimalarial evaluation of a Nigerian hepta-herbal Agbo-iba decoction: Identification of magic bullets and possible facilitators of drug action." Journal of Ethnopharmacology **301**: 115807.	Title
133	Filer, C., G. Allen, G. Brown and T. Brown (2006). "Famciclovir-Selected References." Drugs **66**: 2397-2416.	Title
134	Flammiger, A. and H. Maibach (2006). "Drug dosage in the elderly: dermatological drugs." Drugs & aging **23**: 203-215.	Title

135	Fleisher, B., J. Unum, J. Shao and G. An (2015). "Ingredients in fruit juices interact with dasatinib through inhibition of BCRP: a new mechanism of beverage-drug interaction." Journal of Pharmaceutical Sciences **104**(1): 266-275.	Title
136	Flynn, C. A. (2011). FEXOFENADINE AND ORGANIC ANION TRANSPORTING POLYPEPTIDES (OATPs): TRANSPORT AND DRUG-DRUG INTERACTIONS, University of Kansas.	Title
137	Flynn, C. A., Y. Alnouti and G. A. Reed (2011). "Quantification of the transporter substrate fexofenadine in cell lysates by liquid chromatography/tandem mass spectrometry." Rapid communications in mass spectrometry **25**(16): 2361-2366.	Abstract
138	Fuentes Noriega, I. (2014). "¿ Qué sabe usted acerca de... la fexofenadina como antihistamínico?" Revista mexicana de ciencias farmacéuticas **45**(4): 1-3.	Other language
139	Fujita, D., Y. Saito, T. Nakanishi and I. Tamai (2016). "Organic anion transporting polypeptide (OATP) 2B1 contributes to gastrointestinal toxicity of anticancer drug SN-38, active metabolite of irinotecan hydrochloride." Drug Metabolism and Disposition **44**(1): 1-7.	Title
140	Furihata, T., Z. Fu, Y. Suzuki, S. Matsumoto, H. Morio, A. Tsubota, S. Matsumoto and K. Chiba (2015). "Differential inhibition features of direct-acting anti-hepatitis C virus agents against human organic anion transporting polypeptide 2B1." International journal of antimicrobial agents **46**(4): 381-388.	Title
141	Gillard, M., M. S. Benedetti, P. Chatelain and E. Baltes (2005). "Histamine H 1 receptor occupancy and pharmacodynamics of second generation H 1-antihistamines." Inflammation research **54**: 367-369.	Title
142	Glaeser, H. (2011). "Importance of P-glycoprotein for drug–drug interactions." Drug transporters: 285-297.	Title
143	Graft, D. F., D. I. Bernstein, A. Goldsobel, E. O. Meltzer, J. Portnoy and J. Long (2001). "Safety of fexofenadine in children treated for seasonal allergic rhinitis." Annals of Allergy, Asthma & Immunology **87**(1): 22-26.	Abstract
144	Granung, J. (2017). Optimering av rensningsprocessen för hel svartpeppar och timjan.	Other language
145	Grimstein, M. and S.-M. Huang (2018). "A regulatory science viewpoint on botanical–drug interactions." journal of food and drug analysis **26**(2): S12-S25.	Title

146	Grzelewska-Rzymowska, I. and M. Górska "Leki przeciwhistaminowe drugiej generacji-farmakokinetyka i farmakodynamika." Przewodnik Lekarza/Guide for GPs **4**(3): 132-139.	Other language
147	Gundogdu, E., I. G. Alvarez, M. B. Sanz and E. Karasulu (2011). "Assessment of fexofenadine hydrochloride permeability and dissolution with an anionic surfactant using Caco-2 cells." Die Pharmazie-An International Journal of Pharmaceutical Sciences **66**(10): 747-753.	Full text
148	Guo, A., W. Zhu, C. Zhang, S. Wen, X. Chen, M. Chen, J. Zhang, J. Su, W. Chen and Y. Zhao (2015). "Association of FCER1A genetic polymorphisms with risk for chronic spontaneous urticaria and efficacy of nonsedating H1-antihistamines in Chinese patients." Archives of dermatological research **307**: 183-190.	Title
149	Gurley, B. J., A. Swain, D. K. Williams, G. Barone and S. K. Battu (2008). "Gauging the clinical significance of P-glycoprotein-mediated herb-drug interactions: comparative effects of St. John's wort, Echinacea, clarithromycin, and rifampin on digoxin pharmacokinetics." Molecular nutrition & food research **52**(7): 772-779.	Title
150	Halls, G. "Scientific Poster Presentations, I."	Title
151	Hameed, A., N. Sharif, M. Ashraf, E. Khan, S. Gul, Z. Khan and N. Hayat (2018). "Pharmaceutical Equivalent Analysis Work for Fexofenadine Preparations Accessible in Pakistan." SAJ Pharma Pharmacol **5**: 203.	Title
152	Hasan, I., S. Halder and M. S. Reza "Fexofenadine HCl Immediate Release Tablets: In vitro Characterization and Evaluation of Excipients."	Abstract
153	He, S.-M., A.-K. Yang, X.-T. Li, Y.-M. Du and S.-F. Zhou (2010). "Effects of herbal products on the metabolism and transport of anticancer agents." Expert Opinion on Drug Metabolism & Toxicology **6**(10): 1195-1213.	Title
154	Hovancakova, J., G. Schoretsanitis, M. Grözinger, G. Gründer and M. Paulzen (2016). "Interaction between risperidone, venlafaxine, and metronidazole: an unknown thread." Journal of Clinical Psychopharmacology **36**(6): 730-733.	Title
155	Howarth, P. H., M. A. Stern, L. Roi, R. Reynolds and J. Bousquet (1999). "Double-blind, placebo-controlled study comparing the efficacy and safety of fexofenadine hydrochloride (120 and 180 mg once daily) and cetirizine in seasonal allergic rhinitis." Journal of allergy and clinical immunology **104**(5): 927-933.	Abstract
156	Hsin, C.-h., M. S. Stoffel, M. Gazzaz, E. Schaeffeler, M. Schwab, U. Fuhr and M. Taubert (2020). "Combinations of common SNPs of the transporter gene ABCB1 influence apparent bioavailability, but not renal elimination of oral digoxin." Scientific Reports **10**(1): 12457.	Title

157	Hussaarts, K. G., D. P. Hurkmans, E. Oomen-de Hoop, L. J. van Harten, S. Berghuis, R. J. van Alphen, L. E. Spierings, Q. C. van Rossum-Schornagel, M. B. Vastbinder and R. H. van Schaik (2019). "Impact of curcumin (with or without piperine) on the pharmacokinetics of tamoxifen." Cancers **11**(3): 403.	Title
158	Ieiri, I., Y. Doi, K. Maeda, T. Sasaki, M. Kimura, T. Hirota, T. Chiyoda, M. Miyagawa, S. Irie and K. Iwasaki (2012). "Microdosing clinical study: pharmacokinetic, pharmacogenomic (SLCO2B1), and interaction (grapefruit juice) profiles of celiprolol following the oral microdose and therapeutic dose." The Journal of Clinical Pharmacology **52**(7): 1078-1089.	Abstract
159	Ieiri, I., C. Nishimura, K. Maeda, T. Sasaki, M. Kimura, T. Chiyoda, T. Hirota, S. Irie, H. Shimizu and T. Noguchi (2011). "Pharmacokinetic and pharmacogenomic profiles of telmisartan after the oral microdose and therapeutic dose." Pharmacogenetics and genomics **21**(8): 495-505.	Title
160	Iijima, R., T. Watanabe, K. i. Ishiuchi, T. Matsumoto, J. Watanabe and T. Makino (2018). "Interactions between crude drug extracts used in Japanese traditional Kampo medicines and organic anion-transporting polypeptide 2B1." Journal of Ethnopharmacology **214**: 153-159.	Title
161	Izzo, A. A. and E. Ernst (2009). "Interactions between herbal medicines and prescribed drugs: an updated systematic review." Drugs **69**: 1777-1798.	Title
162	Jaisue, S. (2010). Influence of inflammation on the pharmacokinetics of fexofenadine, a P-gp and Oatp substrate.	Title
163	Jang, J.-H., S.-H. Jeong and Y.-B. Lee (2023). "Quantitative assessment of the relevance of organic-anion-transporting-polypeptide 1B1 and 2B1 polymorphisms in fexofenadine pharmacokinetic variants via pharmacometrics." Journal of Pharmaceutical Analysis.	Full text
164	Jang, J.-H., S.-H. Jeong and Y.-B. Lee (2023). "Establishment of a fexofenadine population pharmacokinetic (PK)–pharmacodynamic (PD) model and exploration of dosing regimens through simulation." Journal of Pharmaceutical Investigation **53**(3): 427-441.	Title
165	Jáuregui Presa, I., B. Navajas Rodríguez, B. Ramos Bareño, P. M. Gamboa Setién, I. Urrutia Etxebarria and I. Antépara Ercoreca (2016). "Chronic urticaria in special populations: children, pregnancy, lactation and elderly people." Current Treatment Options in Allergy **3**: 423-438.	Title
166	Jiang, H., C. Titsch, J. Zeng, B. Jones, P. Joyce, Y. Gandhi, W. Turley, R. Burrell, A. F. Aubry and M. E. Arnold (2017). "Overcoming interference with the detection of a stable isotopically labeled microtracer in the evaluation of beclabuvir absolute bioavailability using a concomitant microtracer approach." Journal of Pharmaceutical and Biomedical Analysis **143**: 9-16.	Title
167	Jin, M. J. and H. K. Han (2010). "Effect of piperine, a major component of black pepper, on the intestinal absorption of fexofenadine and its implication on food–drug interaction." Journal of food science **75**(3): H93-H96.	Animal

168	Johne, A., G. Arold, S. Bauer, S. Krusekopf, I. Mai and I. Roots (2003). "Interactions between herbal medicines and other drugs. The case of St. John's wort: Das Beispiel Johanniskraut." Bundesgesundheitsblatt-Gesundheitsforschung-Gesundheitsschutz **46**: 1061-1067.	Title
169	Johnson, E. J., C. S. Won, K. Köck and M. F. Paine (2017). "Prioritizing pharmacokinetic drug interaction precipitants in natural products: application to OATP inhibitors in grapefruit juice." Biopharmaceutics & drug disposition **38**(3): 251-259.	Title
170	Judson, P. L., R. Abdallah, Y. Xiong, J. Ebbert and J. M. Lancaster (2017). "Complementary and alternative medicine use in individuals presenting for care at a comprehensive cancer center." Integrative cancer therapies **16**(1): 96-103.	Title
171	KAKADE, T. C., A. S. RATHORE, S. LOHIDASAN and K. R. MAHADIK "SEPARATION AND DETERMINATION OF FEXOFENADINE HYDROCHLORIDE AND MONTELUKAST SODIUM IN TABLET DOSAGE FORM USING RP-HPLC."	Abstract
172	Kamei, H., A. Isaji, Y. Noda, K. Ishikawa, K. Senzaki, K. Yamada, K. Sugiura, Y. Tomita and T. Nabeshima (2012). "Effects of single therapeutic doses of promethazine, fexofenadine and olopatadine on psychomotor function and histamine-induced wheal-and flare-responses: a randomized double-blind, placebo-controlled study in healthy volunteers." Archives of dermatological research **304**: 263-272.	Abstract
173	Kato, T., T. Mikkaichi, Y. Yoshigae, N. Okudaira, T. Shimizu, T. Izumi, S. Ando and Y. Matsumoto (2021). "Quantitative analysis of an impact of P-glycoprotein on edoxaban's disposition using a human physiologically based pharmacokinetic (PBPK) model." International Journal of Pharmaceutics **597**: 120349.	Title
174	Katz, D. A., D. R. Grimm, S. C. Cassar, M. C. Gentile, X. Ye, M. J. Rieser, E. F. Gordon, J. E. Polzin, L. E. Gustavson and R. M. Driscoll (2004). "CYP3A5 genotype has a dose-dependent effect on ABT-773 plasma levels." Clinical Pharmacology & Therapeutics **75**(6): 516-528.	Title
175	Kaur, S., S. K. Samal, S. Roy and A. T. Sangamwar (2020). "Successful oral delivery of fexofenadine hydrochloride by improving permeability via phospholipid complexation." European Journal of Pharmaceutical Sciences **149**: 105338.	Abstract
176	Kaviani, N., M. Shahtusi, M. H. N. Tehrani and S. Nazari (2014). "Effect of oral midazolam premedication on children's co-operation before general anesthesia in pediatric dentistry." Journal of Dentistry **15**(3): 123.	Title
177	Keskitalo, J. E., K. J. Kurkinen, P. J. Neuvonen and M. Niemi (2008). "ABCB1 haplotypes differentially affect the pharmacokinetics of the acid and lactone forms of simvastatin and atorvastatin." Clinical Pharmacology & Therapeutics **84**(4): 457-461.	Title

178	Khuda, F., M. Ovais, A. Khan, G. Ali, S. Ullah, W. A. Shah and N. A. Qadar (2019). "Drug-food interactions of commonly available juices of Pakistan." Pakistan Journal of Pharmaceutical Sciences **32**(5).	Title
179	Kim, S. W., J. H. Lee, S. H. Lee, H. J. Hong, M. G. Lee and K.-H. Yook (2013). "ABCB1 c. 2677G> T variation is associated with adverse reactions of OROS-methylphenidate in children and adolescents with ADHD." Journal of clinical psychopharmacology **33**(4): 491-498.	Title
180	Kirby, B., E. D. Kharasch, K. T. Thummel, V. S. Narang, C. J. Hoffer and J. D. Unadkat (2006). "Simultaneous measurement of in vivo P-glycoprotein and cytochrome P450 3A activities." The Journal of Clinical Pharmacology **46**(11): 1313-1319.	Title
181	Kitamura, Y., H. Koto, S. Matsuura, T. Kawabata, H. Tsuchiya, H. Kusuhara, H. Tsujimoto and Y. Sugiyama (2008). "Modest effect of impaired P-glycoprotein on the plasma concentrations of fexofenadine, quinidine, and loperamide following oral administration in collies." Drug Metabolism and Disposition **36**(5): 807-810.	Animal
182	Klotz, U., W. Beil, C. Gleiter, B. Drewelow, E. Garbe, A. Gillessen and E. Mutschler (2003). "Arzneimittelinteraktionen: Mechanismen und klinische Bedeutung." Der Internist **44**: 1444-1449.	Other language
183	Komori, H., D. Fujita, Y. Shirasaki, Q. Zhu, Y. Iwamoto, T. Nakanishi, M. Nakajima and I. Tamai (2021). "MicroRNAs in Apple-Derived Nanoparticles Modulate Intestinal Expression of Organic Anion–Transporting Peptide 2B1/SLCO2B1 in Caco-2 Cells." Drug Metabolism and Disposition **49**(9): 803-809.	Title
184	Krishnaswami, S., B. Kittner, A. Sankoh and J. Barrett (2003). Population pharmacokinetics of fexofenadine in pediatric patients. JOURNAL OF CLINICAL PHARMACOLOGY, SAGE PUBLICATIONS INC 2455 TELLER RD, THOUSAND OAKS, CA 91320 USA.	Title
185	Kruszewski, J. (2009). Leki przeciwhistaminowe w leczeniu alergicznego nieżytu nosa–uaktualnienie 2008/2009, Elsevier. **63:** 5-10.	Other language
186	Kumar, L., M. S. Alam, C. L. Meena, R. Jain and A. K. Bansal (2009). Fexofenadine hydrochloride. Profiles of drug substances, excipients and related methodology, Elsevier. **34:** 153-192.	Title
187	Kuratli, J., C. A. Leonard, R. Schoborg and N. Borel (2023). "Anti-chlamydial effects of azelastine hydrochloride and the impact of the histamine H1 receptor on chlamydial development." Journal of medical microbiology **72**(5): 001691.	Title
188	Lal, J., A. Sharma and S. Jaiswal (2013). "Carbamazepine Pharmacokinetic and Drug Interactions in Poly-Treated Patients." MEDICAL USES, PHARMACOKINETICS AND ADVERSE EFFECTS **11**: 0.	Title

189	Lam, M. P., B. M. Cheung, K. Chopra and V. Tiwari (2012). "Translation Section." Br J Clin Pharmacol **73**(3/492): 499.	Title
190	Lappin, G., M. Seymour, J. Hague, D. Higton and P. Dickinson (2011). A Resource and Data Quality Comparison: Absolute Bioavailability Data from an Oral/IV Crossover and an IV Isotopic Tracer Clinical Design, AAPS Washington, DC;.	Title
191	Lappin, G., M. Seymour, G. Young, D. Higton and H. M. Hill (2011). "AMS method validation for quantitation in pharmacokinetic studies with concomitant extravascular and intravenous administration." Bioanalysis **3**(4): 393-405.	Title
192	Lappin, G., M. Seymour, G. Young, D. Higton and H. M. Hill (2011). "An AMS method to determine analyte recovery from pharmacokinetic studies with concomitant extravascular and intravenous administration." Bioanalysis **3**(4): 407-410.	Title
193	Laux, J., M. Martorelli, N. Späth, F. Maier, M. Burnet and S. A. Laufer (2023). "Selective Inhibitors of Janus Kinase 3 Modify Responses to Lipopolysaccharides by Increasing the Interleukin-10-to-Tumor Necrosis Factor α Ratio." ACS Pharmacology & Translational Science.	Title
194	Lee, H.-W., H. W. Lee, D. J. Park, S. O. Moon, J. H. Ahn, M. J. Kim, S.-D. Kim, J.-E. Kim and Y.-R. Yoon (2008). "The effects of fexofenadine at steady-state on sleep architecture: a study using polysomnography in healthy Korean volunteers." Expert Opinion on Pharmacotherapy **9**(10): 1655-1665.	Abstract
195	Lee Page, R., P. M. Klem and C. Rogers (2005). "Potential elevation of tacrolimus trough concentrations with concomitant metronidazole therapy." Annals of Pharmacotherapy **39**(6): 1109-1113.	Title
196	Lee, S. W., J. Oh, A. H. Kim, S. C. Ji, S.-I. Park, S. H. Yoon, J.-Y. Chung, K.-S. Yu, I.-J. Jang and S. Lee (2020). "Oral absorption of voriconazole is affected by SLCO2B1 c.* 396T> C genetic polymorphism in CYP2C19 poor metabolizers." The Pharmacogenomics Journal **20**(6): 792-800.	Title
197	Lehtisalo, M., S. Taskinen, E. K. Tarkiainen, M. Neuvonen, J. Viinamäki, M. Paile-Hyvärinen, T. O. Lilius, T. Tapaninen, J. T. Backman and A. Tornio (2023). "A comprehensive pharmacogenomic study indicates roles for SLCO1B1, ABCG2 and SLCO2B1 in rosuvastatin pharmacokinetics." British Journal of Clinical Pharmacology **89**(1): 242-252.	Title
198	Leuschner, M. and A. D. Cromarty (2023). "Critical Assessment of Phenotyping Cocktails for Clinical Use in an African Context." Journal of Personalized Medicine **13**(7): 1098.	Title
199	Li, C. G., L. Yang and S.-F. Zhou (2007). "Interactions between Chinese herbal medicines and drugs." Australian journal of acupuncture and Chinese medicine **2**(1): 17-24.	Title

200	Li, L., R. Liu, C. Peng, X. Chen and J. Li (2022). "Pharmacogenomics for the efficacy and side effects of antihistamines." Experimental Dermatology **31**(7): 993-1004.	Title
201	Li, R. (2019). "Estimating in vivo fractional contribution of OATP1B1 to human hepatic active uptake by mechanistically modeling pharmacogenetic data." The AAPS Journal **21**(4): 69.	Title
202	Lippi, G., E. J. Favaloro and C. Mattiuzzi (2014). Combined administration of antibiotics and direct oral anticoagulants: a renewed indication for laboratory monitoring? Seminars in thrombosis and hemostasis, Thieme Medical Publishers.	Title
203	Liu, M.-Z., Y.-L. Zhang, M.-Z. Zeng, F.-Z. He, Z.-Y. Luo, J.-Q. Luo, J.-G. Wen, X.-P. Chen, H.-H. Zhou and W. Zhang (2015). "Pharmacogenomics and herb-drug interactions: merge of future and tradition." Evidence-Based Complementary and Alternative Medicine **2015**.	Title
204	LIU, Q., C. LIU, H.-w. SHANG, L. YANG and L. WANG (2007). "Study on relative bioavailability and pharmacokinetics of fexofenadine hydrochloride capsules in healthy volunteers." Chinese Journal of Pharmaceutical Analysis **27**(11): 1707-1710.	Not Accessible
205	Liyanage, M., C. L. Blaquera, J. Piscitelli, S. R. Penzak, T. D. Nolin, M. F. Paine and J. D. Ma (2023). "Limitations of fexofenadine limited sampling strategy using plasma concentrations and partial area under the concentration-time curve to estimate transporter activity in healthy adults." International Journal of Clinical Pharmacology and Therapeutics **61**(6): 262.	Not Accessible
206	Lohse, M. J., A. Lorenzen and B. Müller-Oerlinghausen (2004). "Psychopharmaka." Arzneiverordnungs-Report 2003: Aktuelle Daten, Kosten, Trends und Kommentare: 704-749.	Other language
207	Lopes, M., M. A. Coimbra, M. d. C. Costa and F. Ramos (2023). "Food supplement vitamins, minerals, amino-acids, fatty acids, phenolic and alkaloid-based substances: An overview of their interaction with drugs." Critical Reviews in Food Science and Nutrition **63**(19): 4106-4140.	Title
208	Lund, K. C. and T. Pantuso (2014). "Combination effects of quercetin, resveratrol and curcumin on in vitro intestinal absorption." Journal of Restorative Medicine **3**(1): 112-120.	Title
209	Madrigal-Bujaidar, E., E. Pérez-Montoya, S. García-Medina, J. M. Cristóbal-Luna, J. A. Morales-González, E. O. Madrigal-Santillán, R. Paniagua-Pérez and I. Álvarez-González (2019). "Pharmacokinetic parameters of ifosfamide in mouse pre-administered with grapefruit juice or naringin." Scientific Reports **9**(1): 16621.	Animal
210	Maeda, K., I. Ieiri, K. Yasuda, A. Fujino, H. Fujiwara, K. Otsubo, M. Hirano, T. Watanabe, Y. Kitamura and H. Kusuhara (2006). "Effects of organic anion transporting polypeptide 1B1 haplotype on pharmacokinetics of pravastatin, valsartan, and temocapril." Clinical Pharmacology & Therapeutics **79**(5): 427-439.	Title

211	Maeda, K. and Y. Sugiyama (2011). "Novel strategies for microdose studies using non-radiolabeled compounds." Advanced drug delivery reviews **63**(7): 532-538.	Title
212	Maeda, K., J. Takano, Y. Ikeda, T. Fujita, Y. Oyama, K. Nozawa, Y. Kumagai and Y. Sugiyama (2011). "Nonlinear pharmacokinetics of oral quinidine and verapamil in healthy subjects: a clinical microdosing study." Clinical Pharmacology & Therapeutics **90**(2): 263-270.	Title
213	Majstorović, T. (2021). Procjena informiranosti magistara farmacije o interakcijama hrane i lijekova u Hrvatskoj, Josip Juraj Strossmayer University of Osijek. FACULTY OF FOOD TECHNOLOGY ….	Other language
214	Mannel, M. (2004). "Drug interactions with St John's wort: mechanisms and clinical implications." Drug safety **27**: 773-797.	Title
215	Mansfield, L. E. (2006). "Once-daily immediate-release fexofenadine and sustained-release pseudoephedrine combination: a new treatment option for allergic rhinitis." Expert Opinion on Pharmacotherapy **7**(7): 941-951.	Expert Opinion
216	Mansfield, L. E. (2008). "Fexofenadine in pediatrics: oral tablet and suspension formulations." Expert Opinion on Pharmacotherapy **9**(2): 329-337.	Abstract
217	Mansoor, N., R. Nadig, R. Munoth, V. Reddy and Y. Kamdi (2021). "COMPARATIVE ANALYSIS OF MONTELUKAST, LEVOCETIRIZINE, CETIRIZINE & FEXOFENADINE."	Full text
218	Marie, S., I. Hernández-Lozano, L. Breuil, C. Truillet, S. Hu, A. Sparreboom, N. Tournier and O. Langer (2021). "Imaging-Based Characterization of a Slco2b1 (-/-) Mouse Model Using [11C] Erlotinib and [99mTc] Mebrofenin as Probe Substrates." Pharmaceutics **13**(6): 918.	Animal
219	Markham, A. and A. J. Wagstaff (1998). "Fexofenadine." Drugs **55**(2).	Title
220	Markowitz, J. S., J. L. Donovan, C. L. DeVane, R. M. Taylor, Y. Ruan, J.-S. Wang and K. D. Chavin (2003). "Effect of St John's wort on drug metabolism by induction of cytochrome P450 3A4 enzyme." Jama **290**(11): 1500-1504.	Abstract
221	Martinez, J.-M., S. Khier, S. Morita, C. Rauch and D. Fabre (2014). "Population pharmacokinetic analysis of fexofenadine in Japanese pediatric patients." Journal of pharmacokinetics and pharmacodynamics **41**: 187-195.	Abstract
222	Mbah, C. (2008). "Studies on the Effects of Electrolytes and pH Control on the Lipophilicity of Fexofenadine Hydrochloride." Bio-Research **6**(1): 301-302.	Title

223	McCrea, C. E. and M. E. Pritchard (2011). "Concurrent herb-prescription medication use and health care provider disclosure among university students." Complementary Therapies in Medicine **19**(1): 32-36.	Title
224	McLean, C., A. Wilson and R. B. Kim (2016). "Impact of transporter polymorphisms on drug development: is it clinically significant?" The Journal of Clinical Pharmacology **56**: S40-S58.	Title
225	Meltzer, E. O., N. A. Rosario, H. Van Bever and L. Lucio (2021). "Fexofenadine: review of safety, efficacy and unmet needs in children with allergic rhinitis." Allergy, Asthma & Clinical Immunology **17**: 1-11.	Title
226	Mendes, R. A. (2022). "Farmácia Maia, Porto."	Other language
227	Metta, G. M. and A. R. M. de Oliveira (2012). "CM004 Evaluation of chiral stationary phases in the separation of the enantiomers of fexofenadine." Revista Eletrônica de Farmácia **9**(1): 1-1.	Not Accessible
228	MIAO, C.-y., Y. CHEN and J.-f. CHEN "Progress and research in the effect of itraconazole on pharmacokinetic drug-drug interactions." Chinese Journal of Clinical Pharmacology and Therapeutics **14**(10): 1183.	Title
229	Mills, E., V. M. Montori, P. Wu, K. Gallicano, M. Clarke and G. Guyatt (2004). "Interaction of St John's wort with conventional drugs: systematic review of clinical trials." Bmj **329**(7456): 27-30.	Title
230	Mischoulon, D. and M. H. Rapaport (2019). "Current role of herbal and natural preparations." Antidepressants: From Biogenic Amines to New Mechanisms of Action: 225-252.	Title
231	Mitra, A., D. Pal, M. Minocha and D. Kwatra (2010). "Compatibility risks between drugs and herbal medicines or botanical supplements." Toxicology Letters **196**: S17.	Title
232	Miura, M. and T. Uno (2010). "Clinical pharmacokinetics of fexofenadine enantiomers." Expert Opinion on Drug Metabolism & Toxicology **6**(1): 69-74.	Expert Opinion
233	Miura, M., T. Uno, T. Tateishi and T. Suzuki (2007). "Determination of fexofenadine enantiomers in human plasma with high-performance liquid chromatography." Journal of pharmaceutical and biomedical analysis **43**(2): 741-745.	Short Communication

234	Mohos, V., E. Fliszár-Nyúl, O. Ungvári, K. Kuffa, P. W. Needs, P. A. Kroon, Á. Telbisz, C. Özvegy-Laczka and M. Poór (2020). "Inhibitory effects of quercetin and its main methyl, sulfate, and glucuronic acid conjugates on cytochrome P450 enzymes, and on OATP, BCRP and MRP2 transporters." Nutrients **12**(8): 2306.	Abstract
235	Mörike, K. and C. Gleiter (2003). "Pflanzliche Arzneimittel: Eine mögliche Quelle von Wechselwirkungen." Der Internist **44**: 748-752.	Other language
236	Morishita, H., K. Okawa, M. Ishii, K. Mizoi, M.-a. Ito, H. Arakawa, K. Yano and T. Ogihara (2020). "Gastrointestinal absorption of pimozide is enhanced by inhibition of P-glycoprotein." Plos one **15**(10): e0232438.	Title
237	Morita, T., T. Akiyoshi, T. Tsuchitani, H. Kataoka, N. Araki, K. Yajima, K. Katayama, A. Imaoka and H. Ohtani (2022). "Inhibitory Effects of Cranberry Juice and Its Components on Intestinal OATP1A2 and OATP2B1: Identification of Avicularin as a Novel Inhibitor." Journal of agricultural and food chemistry **70**(10): 3310-3320.	Title
238	Myers, M. J., M. Martinez, F. Li, K. Howard, H. F. Yancy, L. Troutman and M. Sharkey (2018). "Impact of ABCB 1 genotype in Collies on the pharmacokinetics of R-and S-fexofenadine." Journal of veterinary pharmacology and therapeutics **41**(6): 805-814.	Animal
239	Nadkar, M. Y., M. Tiwaskar, S. Kalra, S. N. Shah, B. Bansode, A. Dutta, S. Bajaj, S. Aggarwal, Y. P. S. Balhara and A. Das10 (2017). "Association of Physicians of India: position statement on role of chirally pure molecules in clinical practice." J Assoc Physicians India **65**(12): 49-60.	Title
240	Namgoong, S., H. S. Cheong, J. O. Kim, L. H. Kim, H. S. Na, I. S. Koh, M. W. Chung and H. D. Shin (2015). "Comparison of genetic variations of the SLCO1B1, SLCO1B3, and SLCO2B1 genes among five ethnic groups." Environmental Toxicology and Pharmacology **40**(3): 692-697.	Title
241	Nathan, R. A., J. Mason, D. I. Bernstein, W. C. Howland, H. B. Kaiser, E. O. Meltzer and N. Segall (1999). "Long-term tolerability of fexofenadine in healthy volunteers." Clinical drug investigation **18**: 317-328.	Abstract
242	Naveed, S., F. Qamar, S. Z. G. Sarwar and M. T. Alam "ASSAY OF FEXOFENADINE BRANDS USING UV SPECTROPHOTOMETER."	Abstract
243	Naveed, S., F. Qamar, S. Zainab, G. Sarwar, K. Usmanghani and T. Alam (2015). "EFFECT OF ACIDIC AND ALKALINE MEDIUM ON FEXOFENADINE BRANDS USING UV SPECTROPHOTOMETER." Can J App Sci **5**(01): 21-25.	Abstract
244	Neustadt, J. (2006). "Herb-drug interactions: What clinicians need to know." Integrative Med **5**: 16-26.	Title

245	Nirogi, R. V., V. N. Kandikere, M. Shukla, K. Mudigonda, S. Maurya and P. Komarneni (2007). "Quantification of fexofenadine in human plasma by liquid chromatography coupled to electrospray tandem mass spectrometry using mosapride as internal standard." Biomedical Chromatography **21**(2): 209-216.	Full text
246	Ogura, J., H. Yamaguchi and N. Mano (2020). "Stimulatory effect on the transport mediated by organic anion transporting polypeptide 2B1." Asian Journal of Pharmaceutical Sciences **15**(2): 181-191.	Title
247	Oliveira, D. C. d. (2006). "Desenvolvimento e validação de métodos para avaliação de cloridrato de fexofenadina."	Other language
248	Olivier, C. E. (2022). "Considering Intestinal Hyperpermeability and Immune-Inflammatory Metabolism in the Treatment of Food Allergy." European Journal of Clinical Medicine **3**(3): 13-18.	Title
249	Pandya, S. J., T. Pasha, A. Bhandari, J. Patel, T. Naitik and T. Upama (2011). "Physical Characterization and Enhance the Solubility of Fexofenadine-[Beta]-Cyclodextrin Inclusion Complexes." Journal of Current Pharma Research **1**(3): 260.	Not Accessible
250	Park, J., C. Choi, K. Jeon, J. Bae, C. Jang and S. Lee (2011). "ASSOCIATION BETWEEN ABCC2-24C> T POLYMORPHISM AND THE PHARMACOKINETICS OF FEXOFENADINE." BASIC & CLINICAL PHARMACOLOGY & TOXICOLOGY **109**: 64-64.	Not Accessible
251	Park, J.-I., B.-S. Kang and C.-I. Choi (2012). "Lack of association between MRP2 C-24T variant and the pharmacokinetics of fexofenadine." PHARMACOTHERAPY **32**(10): E301-E301.	Not Accessible
252	Paśko, P., T. Rodacki, R. Domagała-Rodacka, K. Palimonka, M. Marcinkowska and D. Owczarek (2017). "Second generation H1-antihistamines interaction with food and alcohol—a systematic review." Biomedicine & Pharmacotherapy **93**: 27-39.	Title
253	Patle, D. and S. Nagar (2017). "UV-visible spectrophotometric estimation of montelukast and fexofenadine by simultaneous equation method in bulk & combined tablet dosage form." Current Trends in Biotechnology and Pharmacy **11**(4): 382-388.	Abstract
254	Paul, E., J. Berth-Jones, J. Ortonne and M. Stern (1998). "Fexofenadine hydrochloride in the treatment of chronic idiopathic urticaria: a placebo-controlled, parallel-group, dose-ranging study." Journal of dermatological treatment **9**(3): 143-149.	Abstract
255	Pereverzev, A. and O. Ostroumova (2021). "Food-drug interaction as a risk factor of drug-induced diseases: epidemiology, risk factors, potential mechanisms of interaction." Kachestvennaya Klinicheskaya Praktika= Good Clinical Practice(3): 31-38.	Title

256	Perrela Artero, A. and A. J. Fanlo Villacampa "Interacciones fármaco/alimento."	Other language
257	Persky, A. M. (2015). "9 Drug, Nutrient, and Exercise Interactions." Nutrition for Elite Athletes: 205.	Title
258	Petri, N. (2005). Involvement of Membrane Transport Proteins in Intestinal Absorption and Hepatic Disposition of Drugs Using Fexofenadine as a Model Drug, Acta Universitatis Upsaliensis.	Abstract
259	Petri, N., O. Borgå, L. Nyberg, M. Hedeland, U. Bondesson and H. Lennernäs (2005). "Transport mechanisms of fexofenadine in jejunum, ileum and colon: experiences from a novel in vivo intubation approach."	Not Accessible
260	Petri, N., E. Lundgren, P. Forsell, M. Hedeland, U. Bondesson, L. Knutson and H. Lennernäs (2005). "First-pass effects of verapamil on the absorption and disposition of fexofenadine in the porcine model."	Title
261	Philpot, E. E. (2000). Safety of second generation antihistamines. Allergy and asthma proceedings, OceanSide Publications.	Title
262	Pinto, L., P. Bapat, F. de Lima Moreira, A. Lubetsky, R. de Carvalho Cavalli, H. Berger, V. L. Lanchote and G. Koren (2021). "Chiral Transplacental Pharmacokinetics of Fexofenadine: Impact of P-Glycoprotein Inhibitor Fluoxetine Using the Human Placental Perfusion Model." Pharmaceutical Research **38**: 647-655.	Title
263	Pippa, L. F., C. P. Vieira, J. A. Caris, A. Rocha, M. P. Marques, C. P. Garcia, R. E. F. Rezende and V. L. Lanchote (2023). "Effect of chronic hepatitis C on the activity of the membrane transporters P-gp and OATP1B1/BCRP on patients with different stages of hepatic fibrosis." Clinical Pharmacology & Therapeutics.	Abstract
264	PLA, T. and A. TRANSPOR (2017). "Cases sampling Cases were selected from EUROCAT Northern Netherlands (NNL), a population-based registry for children with congenital anomalies born in the Northern provinces of the Netherlands. EUROCAT NNL registers fetuses or children with major congenital anomalies diagnosed before or after birth, and up to 10 years old." Paving ways for personalizing drug therapy during pregnancy: 51.	Not Accessible
265	Pohl, O., R. H. Zobrist and J.-P. Gotteland (2015). "The clinical pharmacology and pharmacokinetics of ulipristal acetate for the treatment of uterine fibroids." Reproductive Sciences **22**(4): 476-483.	Title
266	Polaka, S., K. Rajpoot, M. Tekade, M. C. Sharma and R. K. Tekade (2022). Food–drug interactions and their implications on oral drug bioavailability. Pharmacokinetics and Toxicokinetic Considerations, Elsevier**:** 263-289.	Title

267	Pradeepa, B., T. Vijayakumar, K. Manikandan and A. K. Kammala (2023). "Cytochrome P450-mediated alterations in clinical pharmacokinetic parameters of conventional drugs coadministered with piperine: a systematic review and meta-analysis." Journal of Herbal Medicine **41**: 100713.	Title
268	Prandota, J. (2004). "Limitations in the clinical usefulness of single-dose pharmacokinetic studies of drugs and a bayesian approach for the estimation of kinetic parameters." American journal of therapeutics **11**(4): 295-301.	Title
269	Rapiejko, P., D. Jurkiewicz, W. Pietruszewska, B. Zielnik-Jurkiewicz, J. Woroń and A. Lipiec (2018). "Treatment strategy of allergic rhinitis in the face of modern world threats." Otolaryngologia Polska **72**(2).	Title
270	Reddy, M. R., S. K. Jha and K. Parameshwar (2018). "ROLE OF VARIOUS NATURAL AND SYNTHETIC POLYMERS IN DEVELOPMENT AND IN-VITRO CHARACTERIZATION OF ORAL CONTROLLED DRUG DELIVERY SYSTEM OF FEXOFENADINE." Pharmaceutical Research **8**(03).	Title
271	Roedler, R., M. M. Neuhauser and S. R. Penzak (2007). "Does metronidazole interact with CYP3A substrates by inhibiting their metabolism through this metabolic pathway? Or should other mechanisms be considered?" Annals of Pharmacotherapy **41**(4): 653-658.	Title
272	Rosskopf, D., H. Kroemer and W. Siegmund (2009). "Pharmakokinetische Probleme in der Praxis." DMW-Deutsche Medizinische Wochenschrift **134**(08): 345-356.	Title
273	Rowland, M. (2012). "Microdosing: a critical assessment of human data." Journal of pharmaceutical sciences **101**(11): 4067-4074.	Title
274	RUSSELL, T. and P. RPH (2005). "PETER L. BONATE, PHD FCP." Pharmacokinetics in Drug Development: Clinical Study Design and Analysis **1**: 149.	Title
275	Sane, R. S., K. W. K. Cheung, E. Cho, B. M. Liederer, J. Hanover, V. Malhi, E. Plise, S. Wong and L. Musib (2021). "Evaluation of ipatasertib interactions with itraconazole and coproporphyrin I and III in a single drug interaction study in healthy subjects." Journal of Pharmacology and Experimental Therapeutics **378**(2): 87-95.	Title
276	Santamaría, E., J. A. Estévez, J. Riba, I. Izquierdo and M. Valle (2017). "Population pharmacokinetic modelling of rupatadine solution in 6–11 year olds and optimisation of the experimental design in younger children." PLoS One **12**(4): e0176091.	Title
277	Santamaría Masdeu, E. (2020). "Estrategia de desarrollo de rupatadina solución oral en niños de 2 a 5 años con rinitis alérgica: la farmacocinética poblacional como herramienta para la optimización del diseño de ensayos clínicos."	Other language

278	Schepers, J. M., P. Potter and C. Van Niekerk (2003). "The effect of Telfast (R) 180 on driver behaviour, decision-making and reaction time." SA Journal of Industrial Psychology **29**(3): 76-86.	Abstract
279	Scherf-Clavel, O. (2022). "Drug–Drug Interactions With Over-The-Counter Medicines: Mind the Unprescribed." Therapeutic Drug Monitoring **44**(2): 253-274.	Title
280	Schoepke, N., M. K. Church and M. Maurer (2013). "The inhibition by levocetirizine and fexofenadine of the histamine-induced wheal and flare response in healthy Caucasian and Japanese volunteers." Acta Dermato-Venereologica **93**(3): 286-293.	Investigative Report
281	Schulkin, A., E. Yakusheva and I. Chernykh (2015). "PHARMACOKINETICS OF FEXOFENADINE IN THE SETTING OF DYSFUNCTION OF THYROID GLAND." Pharmacy & Pharmacology **3**(5s): 122-123.	Animal
282	Schulze, J., M. Johansson, A. Rane and L. Ekstrom (2012). "Genetic variation in SLCO2B1 is associated with serum levels of testosterone and its metabolites prior to and two days after testosterone administration." Current Pharmacogenomics and Personalized Medicine (Formerly Current Pharmacogenomics) **10**(3): 226-230.	Abstract
283	Schwab, D., A. Portron, Z. Backholer, B. Lausecker and K. Kawashima (2013). "A novel double-tracer technique to characterize absorption, distribution, metabolism and excretion (ADME) of [14 C] tofogliflozin after oral administration and concomitant intravenous microdose administration of [13 C] tofogliflozin in humans." Clinical pharmacokinetics **52**: 463-473.	Title
284	Seymour, M. A. (2011). "Accelerator MS: its role as a frontline bioanalytical technique." Bioanalysis **3**(24): 2817-2823.	Title
285	Sheu, M.-J., Y.-N. Teng, Y.-Y. Chen and C.-C. Hung (2014). "The functional influences of common ABCB1 genetic variants on the inhibition of P-glycoprotein by Antrodia cinnamomea extracts." PLoS One **9**(2): e89622.	Abstract
286	Shruthi, K. (2017). Comparison of Efficacy and Safety of Fexofenadine and Desloratadine in Treatment of Chronic Spontaneous Urticaria at a Tertiary Care Hospital, Rajiv Gandhi University of Health Sciences (India).	Abstract
287	Simons, F. E. R. and K. J. Simons (1999). "Clinical pharmacology of new histamine H 1 receptor antagonists." Clinical pharmacokinetics **36**: 329-352.	Abstract
288	Simpson, K. and B. Jarvis (2000). "Fexofenadine: a review of its use in the management of seasonal allergic rhinitis and chronic idiopathic urticaria." Drugs **59**: 301-321.	Title
289	Singman, E. L., B. Hocum, J. Yohannan and V. Pearson (2015). "Fluvoxamine-associated oscillopsia and a role for personalized medication dosing." Drug Metabolism and Personalized Therapy **30**(4): 271-275.	Title

290	Sjögren, E., H. Thorn and C. Tannergren (2016). "In silico modeling of gastrointestinal drug absorption: predictive performance of three physiologically based absorption models." Molecular pharmaceutics **13**(6): 1763-1778.	Title
291	Sjögren, E., H. Thörn and C. Tannergren (2017). "Reply to "Comment on 'In silico modeling of gastrointestinal drug absorption: predictive performance of three physiologically based absorption models'"." Molecular Pharmaceutics **14**(1): 340-343.	Title
292	Skowronski, G. A., M. S. Abdel-Rahman and R. M. Turkall (2002). "Application of Kinetic and Dynamic Data for Antihistamines to Risk Characterization in Sensitive Populations." Human and Ecological Risk Assessment **8**(4): 859-876.	Title
293	Smith, P., J. M. Bullock, B. M. Booker, C. E. Haas, C. S. Berenson and W. J. Jusko (2004). "The influence of St. John's wort on the pharmacokinetics and protein binding of imatinib mesylate." Pharmacotherapy: The Journal of Human Pharmacology and Drug Therapy **24**(11): 1508-1514.	Title
294	So, M., P. Bozzo, M. Inoue and A. Einarson (2010). "Safety of antihistamines during pregnancy and lactation." Canadian Family Physician **56**(5): 427-429.	Title
295	Spanakis, M., E. Patelarou and A. Patelarou (2022). "Drug-Food Interactions with a Focus on Mediterranean Diet." Applied Sciences **12**(20): 10207.	Title
296	Spina, E., M. A. Barbieri, G. Cicala, A. Bruno and J. de Leon (2020). "Clinically relevant drug interactions between newer antidepressants and oral anticoagulants." Expert opinion on drug metabolism & toxicology **16**(1): 31-44.	Title
297	Spina, S. P. and E. C. Dillon Jr (2003). "Effect of chronic probenecid therapy on cefazolin serum concentrations." Annals of Pharmacotherapy **37**(5): 621-624.	Title
298	Srinivas, N. R. (2016). "Prediction of area under the curve for a p-glycoprotein, a CYP3A4 and a CYP2C9 substrate using a single time point strategy: assessment using fexofenadine, itraconazole and losartan and metabolites." Drug development and industrial pharmacy **42**(6): 945-957.	Abstract
299	Stein, C. M. (2002). "Are herbal products dietary supplements or drugs? An important question for public safety." Clinical Pharmacology & Therapeutics **71**(6): 411-413.	Title
300	Stern, R. H., J. A. Smithers and S. C. Olson (1998). "Atorvastatin does not produce a clinically significant effect on the pharmacokinetics of terfenadine." The Journal of Clinical Pharmacology **38**(8): 753-757.	Title

301	Sussman, G. L., J. Mason, D. Compton, J. Stewart and N. Ricard (1999). "The efficacy and safety of fexofenadine HCl and pseudoephedrine, alone and in combination, in seasonal allergic rhinitis." Journal of allergy and clinical immunology **104**(1): 100-106.	Abstract
302	Svendsen, P., T. C. El-Galaly, K. Dybkær, M. Bøgsted, M. B. Laursen, A. Schmitz, P. Jensen and H. E. Johnsen (2016). "The application of human phase 0 microdosing trials: a systematic review and perspectives." Leukemia & lymphoma **57**(6): 1281-1290.	Title
303	Sybilski, A. J. (2017). "Feksofenadyna–skuteczny i bezpieczny lek w alergicznym nieżycie nosa oraz pokrzywce." Medycyna Faktów **10**(3 (36)): 267-269.	Other language
304	Sybilski, A. J. (2019). "Feksofenadyna–fakty mówią same za siebie." Medycyna Faktów **12**(3 (44)): 238-241.	Other language
305	Szeleszczuk, Ł., D. M. Pisklak, M. Zielińska-Pisklak and I. Wawer (2017). "Spectroscopic and structural studies of the diosmin monohydrate and anhydrous diosmin." International Journal of Pharmaceutics **529**(1-2): 193-199.	Title
306	Tachibana, T., M. Kato, T. Watanabe, T. Mitsui and Y. Sugiyama (2009). "Method for predicting the risk of drug–drug interactions involving inhibition of intestinal CYP3A4 and P-glycoprotein." Xenobiotica **39**(6): 430-443.	Title
307	Tamai, I. (2013). "How should we handle decreased efficacy caused by DDI on transporters for drug absorption and target-tissue distribution, but not associated with toxicity?" Drug Metabolism and Pharmacokinetics **28**(6): 451-452.	Title
308	Tamai, I. and T. Nakanishi (2013). "OATP transporter-mediated drug absorption and interaction." Current opinion in pharmacology **13**(6): 859-863.	Abstract
309	Tandulwadkar, S. S., S. J. More, A. S. Rathore, A. R. Nikam, L. Sathiyanarayanan and K. R. Mahadik (2012). "Method development and validation for the simultaneous determination of fexofenadine hydrochloride and montelukast sodium in drug formulation using normal phase high-performance thin-layer chromatography." International Scholarly Research Notices **2012**.	Abstract
310	Tannergren, C., T. Knutson, L. Knutson and H. Lennernäs (2003). "The effect of ketoconazole on the in vivo intestinal permeability of fexofenadine using a regional perfusion technique." British journal of clinical pharmacology **55**(2): 182-190.	Abstract
311	Tapaninen, T., T. Karonen, J. T. Backman, P. J. Neuvonen and M. Niemi (2013). "SLCO2B1 c. 935G> A single nucleotide polymorphism has no effect on the pharmacokinetics of montelukast and aliskiren." Pharmacogenetics and genomics **23**(1): 19-24.	Title

312	Tascilar, M., F. A. de Jong, J. Verweij and R. H. Mathijssen (2006). "Complementary and alternative medicine during cancer treatment: beyond innocence." The oncologist **11**(7): 732-741.	Title
313	Tian, R., N. Koyabu, S. Morimoto, Y. Shoyama, H. Ohtani and Y. Sawada (2005). "Functional induction and de-induction of P-glycoprotein by St. John's wort and its ingredients in a human colon adenocarcinoma cell line." Drug metabolism and disposition **33**(4): 547-554.	Title
314	Tikkanen, A., E. Pierrot, F. Deng, V. B. Sánchez, M. Hagström, J. B. Koenderink and H. Kidron (2020). "Food additives as inhibitors of intestinal drug transporter OATP2B1." Molecular Pharmaceutics **17**(10): 3748-3758.	Title
315	Tod, M., S. Goutelle, N. Bleyzac and L. Bourguignon (2019). "A generic model for quantitative prediction of interactions mediated by efflux transporters and cytochromes: application to P-glycoprotein and cytochrome 3A4." Clinical Pharmacokinetics **58**: 503-523.	Title
316	Trivedi, K., P. V. Patel and Z. Pujara (2013). "Development and characterization of liquid and solid self-emulsifying drug delivery system of fexofenadine." Journal of Pharmaceutical Investigation **43**(5): 385-394.	Title
317	Tsuji, D., Y.-I. Kim, H. Nakamichi, T. Daimon, K. Suwa, Y. Iwabe, H. Hayashi, K. Inoue, M. Yoshida and K. Itoh (2013). "Association of ABCB1 polymorphisms with the antiemetic efficacy of granisetron plus dexamethasone in breast cancer patients." Drug Metabolism and Pharmacokinetics **28**(4): 299-304.	Title
318	Türkmen, Ö., Z. A. Şenyiğit and E. Baloğlu (2018). "Formulation and evaluation of fexofenadine hydrochloride orally disintegrating tablets for pediatric use." Journal of Drug Delivery Science and Technology **43**: 201-210.	Abstract
319	Turner, D. B., B. Liu, N. Patel, S. M. Pathak, S. Polak, M. Jamei, J. Dressman and A. Rostami-Hodjegan (2017). "Comment on “In Silico Modeling of Gastrointestinal Drug Absorption: Predictive Performance of Three Physiologically-Based Absorption Models”." Molecular Pharmaceutics **14**(1): 336-339.	Title
320	Umeyama, Y., Y. Fujioka and T. Okuda (2014). "Clarification of P-glycoprotein inhibition-related drug–drug interaction risks based on a literature search of the clinical information." Xenobiotica **44**(12): 1135-1144.	Title
321	Uno, T., Y. Akamine, N. Yasui-Furukori and T. Hirano (2017). "Effects of selective serotonin reuptake inhibitors on the pharmacokinetics of proton pump inhibitors." Neuropsychiatry **7**(4).	Title
322	Vermeer, L. M., C. D. Isringhausen, B. W. Ogilvie and D. B. Buckley (2016). "Evaluation of ketoconazole and its alternative clinical CYP3A4/5 inhibitors as inhibitors of drug transporters: the in vitro effects of ketoconazole, ritonavir, clarithromycin, and itraconazole on 13 clinically-relevant drug transporters." Drug metabolism and disposition **44**(3): 453-459.	Title

323	Walsh, G. M. (2004). Antihistamines (H1 receptor antagonists). Side Effects of Drugs Annual, Elsevier. **27:** 165-171.	Title
324	Wang, P., S. M. Henning, D. Heber and J. V. Vadgama (2015). "Sensitization to docetaxel in prostate cancer cells by green tea and quercetin." The Journal of nutritional biochemistry **26**(4): 408-415.	Title
325	Wang, P., J. V. Vadgama, J. W. Said, C. E. Magyar, N. Doan, D. Heber and S. M. Henning (2014). "Enhanced inhibition of prostate cancer xenograft tumor growth by combining quercetin and green tea." The Journal of nutritional biochemistry **25**(1): 73-80.	Title
326	Wang, Z. (2002). "Hypericum's effect on the pharmacokinetics of fexofenadine." Reactions **921**: 28.	Abstract
327	Wani, T. A., A. H. Bakheit, S. Zargar, Z. S. Alanazi and A. A. Al-Majed (2021). "Influence of antioxidant flavonoids quercetin and rutin on the in-vitro binding of neratinib to human serum albumin." Spectrochimica Acta Part A: Molecular and Biomolecular Spectroscopy **246**: 118977.	Title
328	Whomsley, R. and F.-X. Mathy (2007). "Stereoslective renal tubular secretion of levocetirizine and dextrocetirizine, the two enantiomers of the H1-antihistamine cetirizine."	Title
329	Wong, G. A. and N. H. Shear (2005). "Adverse drug interactions and reactions in dermatology: current issues of clinical relevance." Dermatologic clinics **23**(2): 335-342.	Title
330	Wudel, S. (2007). Einfluss von Haplotypen des MDR1-Gens (Multidrug Resistance Gene) auf die Pharmakokinetik von Fexofenadin und Digoxin bei gesunden Probanden.	Other language
331	Yabut, J., R. Houle, S. Wang, A. Liaw, R. Katwaru, H. Collier, L. Hittle and X. Chu (2022). "Selection of an Optimal In Vitro Model to Assess P-gp Inhibition: Comparison of Vesicular and Bidirectional Transcellular Transport Inhibition Assays." Drug Metabolism and Disposition **50**(7): 909-922.	Title
332	Yakusheva, E., I. Chernykh, A. Shulkin and M. Gatsanoga (2015). "Methods of identification of drugs as P-glycoprotein substrates." IP Pavlov Russian Medical Biological Herald **23**(3): 49-53.	Title
333	Yanai, K., T. Yoshikawa and M. K. Church (2021). "Efficacy and Safety of Non-brain Penetrating H1-Antihistamines for the Treatment of Allergic Diseases." The Functional Roles of Histamine Receptors: 193-214.	Title

334	Yao, Q., B. Qu, Q. Zhou and S. Zeng (2014). "Establishment of reverse-phase high-performance liquid chromatography with chiral reagent derivatization for separation of fexofenadine enantiomers." Zhejiang da xue xue bao. Yi xue ban= Journal of Zhejiang University. Medical Sciences **43**(2): 155-159.	Not Accessible
335	Yasui-Furukori, N., S. Tsuchimine, K. Kubo, M. Ishioka, K. Nakamura and Y. Inoue (2016). "The effects of fluvoxamine on the steady-state plasma concentrations of escitalopram and desmethylescitalopram in depressed Japanese patients." Therapeutic drug monitoring **38**(4): 483-486.	Title
336	Ye, Z. (2010). "Standardized evaluation of TCM drug safety and its safe use in China." Toxicology Letters **196**: S17.	Title
337	Yehia, S. A., M. S. El-Ridi, M. I. Tadros and N. G. El-Sherif (2015). "Enhancement of the oral bioavailability of fexofenadine hydrochloride via Cremophor® El-Based liquisolid tablets." Advanced Pharmaceutical Bulletin **5**(4): 569.	Full text
338	Yiannakopoulou, E. C. (2013). "Pharmacogenomics of phase II metabolizing enzymes and drug transporters: clinical implications." The pharmacogenomics journal **13**(2): 105-109.	Title
339	Zhang, W., Q. Zhang, H. Liang, S. Wang, J. Pan, S. Pan, B. Zhu, Z. Ding, W. Zhao and Y. Ni (2022). "Association of MDR1 C1236T Polymorphisms and B Cell Non-Hodgkin Lymphoma." Journal of Cancer **13**(6): 1768.	Title
340	Zhou, F., L. Zhu, K. Wang and M. Murray (2017). "Recent advance in the pharmacogenomics of human Solute Carrier Transporters (SLCs) in drug disposition." Advanced drug delivery reviews **116**: 21-36.	Title
341	Zhou, S.-F., Z.-W. Zhou, C.-G. Li, X. Chen, X. Yu, C. C. Xue and A. Herington (2007). "Identification of drugs that interact with herbs in drug development." Drug discovery today **12**(15-16): 664-673.	Title
342	Zou, L., P. Spanogiannopoulos, L. M. Pieper, H.-C. Chien, W. Cai, N. Khuri, J. Pottel, B. Vora, Z. Ni and E. Tsakalozou (2020). "Bacterial metabolism rescues the inhibition of intestinal drug absorption by food and drug additives." Proceedings of the National Academy of Sciences **117**(27): 16009-16018.	Title
343	Кирюхина, Л., В. Кузнецова, Е. Аганезова, Н. Яковлева and М. Каменева (2000). "Метод импульсной осциллометрии в диагностике нарушений механики дыхания." Пульмонология(2): 31-36.	Other language
344	Переверзев, А. П. and О. Д. Остроумова (2021). "Взаимодействие пищевых продуктов и лекарственных средств как фактор риска развития лекарственно индуцированных заболеваний: эпидемиология, факторы риска, потенциальные механизмы развития взаимодействий." Качественная клиническая практика(3): 31-38.	Other language

345	Шойхет, Я. Н., В. Б. Колядо, С. И. Трибунский and Т. И. Мартыненко (2002). "Потери здоровья населения Алтайского края от болезней органов дыхания." Пульмонология(3): 27-35.	Other language
346	Щулькин, А., Е. Якушева and И. Черных (2015). "Фармакокинетика фексофенадина при дисфункции щитовидной железы." Фармация и фармакология **3**(Приложение 1): 122-123.	Other language
347	Щулькин, А. В., Е. Н. Якушева, И. В. Черных, А. А. Никифоров and Н. М. Попова (2017). "Влияние тестостерона на функциональную активность гликопротеина-Р." Химико-фармацевтический журнал **51**(9): 3-7.	Other language
348	大橋快, 角本幹夫, 岡野友信, 守屋友加 and 藤田卓也 (2010). マウス大脳皮質初代培養神経細胞における carnitine の輸送機構. 日本薬物動態学会年会講演要旨集 第 25 回日本薬物動態学会年会, 一般社団法人 日本薬物動態学会.	Other language
349	家入一郎 (2012). "5. PK/PD/PGx 臨床研究による最新の知見と展望." 臨床薬理 **43**(4): 281-287.	Other language
350	嵯峨知生, 植木重治, 技師長, 小林則子, 副技師長, 鎌田由美子, 富谷陽子, 主任技官, 平澤裕之 and 戸島洋子 "中 央 検 査 部." Biol. Blood Marrow Transplant **24**: 1180-1186.	Other language
351	猿渡淳二, 古郡規雄, 新岡丈典, 赤嶺由美子, 高嶋綾佳, 兼子直 and 宇野司 (2012). "選択的セロトニン取り込み阻害薬が P-糖タンパクの基質薬物フェキソフェナジンの体内動態に与える影響." Journal of Clinical Psychopharmacology **32**(2): 195-199.	Other language
352	簗本美喜子 (2015). "小腸 P-糖蛋白質を介する薬物相互作用の評価における aliskiren の有用性に関する研究." 静岡県立大学学位論文: 0001-0054.	Other language
353	赤嶺由美子. "Various Drug-Transporters Related to the Fexofenadine Enantiomers Pharmacokinetics." 九州大学, 2014	Other language
354	苗彩云, 陈漪 and 陈江飞 (2009). "伊曲康唑影响的药代动力学相互作用研究进展." 中国临床药理学与治疗学 **14**(10): 1183.	Other language
355	赤嶺由美子 (2015). "フェキソフェナジンの立体選択的体内動態とその規定因子の解析." YAKUGAKU ZASSHI **135**(3): 473-481.	Other language
356	赤嶺由美子, 三浦昌朋, 古郡規雄 and 宇野司 (2010). フェキソフェナジン光学異性体 PK にリファンピシンが及ぼす OATPs 阻害効果. 日本薬物動態学会年会講演要旨集 第 25 回日本薬物動態学会年会, 一般社団法人 日本薬物動態学会.	Other language

357	近藤安佑子. "紅茶抽出物及び含有ポリフェノール成分における Oatp2b1 を介した食物-薬物間相互作用に関する検討." 北海道大学 , 2020	Other language
358	齋藤義正 (2018). 小児がん患者の QOL 向上を目指した支持療法の最適化に関する研究, 金沢大学.	Other language
359	(1997). "[Drug side effects and their prevention]." Hautarzt **48**(8 Suppl): 3-4.	Title
360	(2011). "In brief: fexofenadine (Allegra) and fruit juice." Med Lett Drugs Ther **53**(1365): 41.	Title
361	Amadi, C. N. and A. A. Mgbahurike (2018). "Selected Food/Herb-Drug Interactions: Mechanisms and Clinical Relevance." Am J Ther **25**(4): e423-e433.	Title
362	Amchin, J., W. Zarycranski, K. P. Taylor, D. Albano and P. M. Klockowski (1998). "Effect of venlafaxine on the pharmacokinetics of terfenadine." Psychopharmacol Bull **34**(3): 383-389.	Abstract
363	Baena-Cagnani, C. E. (2004). "Safety and tolerability of treatments for allergic rhinitis in children." Drug Saf **27**(12): 883-898.	Title
364	Bailey, D. G. (2010). "Fruit juice inhibition of uptake transport: a new type of food-drug interaction." Br J Clin Pharmacol **70**(5): 645-655.	Abstract
365	Bednarczyk, D. and M. V. Sanghvi (2020). "Organic anion transporting polypeptide 2B1 (OATP2B1), an expanded substrate profile, does it align with OATP2B1's hypothesized function?" Xenobiotica **50**(9): 1128-1137.	Title
366	Bosilkovska, M., C. Samer, J. Déglon, A. Thomas, B. Walder, J. Desmeules and Y. Daali (2016). "Evaluation of Mutual Drug-Drug Interaction within Geneva Cocktail for Cytochrome P450 Phenotyping using Innovative Dried Blood Sampling Method." Basic Clin Pharmacol Toxicol **119**(3): 284-290.	Abstract
367	Bosilkovska, M., C. F. Samer, J. Déglon, M. Rebsamen, C. Staub, P. Dayer, B. Walder, J. A. Desmeules and Y. Daali (2014). "Geneva cocktail for cytochrome p450 and P-glycoprotein activity assessment using dried blood spots." Clin Pharmacol Ther **96**(3): 349-359.	Abstract
368	Caballero, E., I. Ocaña, J. R. Azanza and B. Sádaba (1999). "[Fexofenadine: a antihistaminic. Review of its practical characteristics]." Rev Med Univ Navarra **43**(2): 93-97.	Title
369	Camelo-Nunes, I. C. (2006). "New antihistamines: a critical view." J Pediatr (Rio J) **82**(5 Suppl): S173-180.	Title

370	Chiou, W. L., S. M. Chung, T. C. Wu and C. Ma (2001). "A comprehensive account on the role of efflux transporters in the gastrointestinal absorption of 13 commonly used substrate drugs in humans." Int J Clin Pharmacol Ther **39**(3): 93-101.	Title
371	Church, M. K. and M. Maurer (2012). "H(1)-antihistamines and urticaria: how can we predict the best drug for our patient?" Clin Exp Allergy **42**(10): 1423-1429.	Title
372	Crowe, A. and C. Wright (2012). "The impact of P-glycoprotein mediated efflux on absorption of 11 sedating and less-sedating antihistamines using Caco-2 monolayers." Xenobiotica **42**(6): 538-549.	Title
373	Darnaud, L., C. Delage, Y. Daali, A. P. Trouvin, S. Perrot, N. Khoudour, N. Merise, L. Labat, B. Etain, F. Bellivier, C. Lloret-Linares, V. Bloch, E. Curis and X. Declèves (2023). "Phenotyping Indices of CYP450 and P-Glycoprotein in Human Volunteers and in Patients Treated with Painkillers or Psychotropic Drugs." Pharmaceutics **15**(3).	Title
374	De Vos, C., K. Mitchev, M. E. Pinelli, M. P. Derde and R. Boev (2008). "Non-interventional study comparing treatment satisfaction in patients treated with antihistamines." Clin Drug Investig **28**(4): 221-230.	Title
375	Devillier, P., N. Roche and C. Faisy (2008). "Clinical pharmacokinetics and pharmacodynamics of desloratadine, fexofenadine and levocetirizine : a comparative review." Clin Pharmacokinet **47**(4): 217-230.	Title
376	Di Giacomo, B., D. Coletta, B. Natalini, M. H. Ni and R. Pellicciari (1999). "A new synthesis of carboxyterfenadine (fexofenadine) and its bioisosteric tetrazole analogs." Farmaco **54**(9): 600-610.	Abstract
377	Dresser, G. K. and D. G. Bailey (2003). "The effects of fruit juices on drug disposition: a new model for drug interactions." Eur J Clin Invest **33 Suppl 2**: 10-16.	Title
378	DuBuske, L. (2007). "Desloratadine for chronic idiopathic urticaria: a review of clinical efficacy." Am J Clin Dermatol **8**(5): 271-283.	Title
379	Duran, I., J. Carles, I. Bulat, P. Hellemans, A. Mitselos, P. Ward, J. Jiao, D. Armas and C. Chien (2020). "Pharmacokinetic Drug-Drug Interaction of Apalutamide, Part 1: Clinical Studies in Healthy Men and Patients with Castration-Resistant Prostate Cancer." Clin Pharmacokinet **59**(9): 1135-1148.	Abstract
380	Eseverri, J. (2000). "[Projection of new antihistamines]." Allergol Immunopathol (Madr) **28**(3): 143-152.	Title
381	Estes, K. S. and M. A. González (1999). "Pharmacokinetic aspects of second-generation H1 antihistamines: a reply to Philpot." Int J Clin Pharmacol Ther **37**(6): 310-312.	Title

382	Ferrer, M. (2011). "Pharmacokinetic evaluation of levocetirizine." Expert Opin Drug Metab Toxicol **7**(8): 1035-1047.	Title
383	Fukumori, S., M. Masago, K. Ishida, Y. Kayano, M. Taguchi and Y. Hashimoto (2009). "Temperature-dependent specific transport of levofloxacin in human intestinal epithelial LS180 cells." Biopharm Drug Dispos **30**(8): 448-456.	Title
384	Fukumori, S., T. Murata, M. Takaai, K. Tahara, M. Taguchi and Y. Hashimoto (2008). "The apical uptake transporter of levofloxacin is distinct from the peptide transporter in human intestinal epithelial Caco-2 cells." Drug Metab Pharmacokinet **23**(5): 373-378.	Title
385	Gillman, S., M. Gillard and M. Strolin Benedetti (2009). "The concept of receptor occupancy to predict clinical efficacy: a comparison of second generation H1 antihistamines." Allergy Asthma Proc **30**(4): 366-376.	Title
386	Gómez-Garduño, J., R. León-Rodríguez, R. Alemón-Medina, B. E. Pérez-Guillé, R. E. Soriano-Rosales, A. González-Ortiz, J. L. Chávez-Pacheco, E. Solorio-López, P. Fernandez-Pérez and L. Rivera-Espinosa (2022). "Phytochemicals That Interfere With Drug Metabolism and Transport, Modifying Plasma Concentration in Humans and Animals." Dose Response **20**(3): 15593258221120485.	Title
387	González, M. A. and K. S. Estes (1998). "Pharmacokinetic overview of oral second-generation H1 antihistamines." Int J Clin Pharmacol Ther **36**(5): 292-300.	Title
388	Greenblatt, D. J. (2009). "Analysis of drug interactions involving fruit beverages and organic anion-transporting polypeptides." J Clin Pharmacol **49**(12): 1403-1407.	Title
389	Greisner, W. A., 3rd (2004). "Onset of action for the relief of allergic rhinitis symptoms with second-generation antihistamines." Allergy Asthma Proc **25**(2): 81-83.	Title
390	Hay, J. W. and M. A. Kaliner (2009). "Costs of second-generation antihistamines in the treatment of allergic rhinitis: US perspective." Curr Med Res Opin **25**(6): 1421-1431.	Title
391	Holgate, S., R. Powell, M. Jenkins and O. Ali (2005). "A treatment for allergic rhinitis: a view on the role of levocetirizine." Curr Med Res Opin **21**(7): 1099-1106.	Title
392	Howard, D. R., R. Haribhakti, B. Kittner and P. Agrawala (2005). "Single-dose and steady-state bioequivalence of fexofenadine and pseudoephedrine combination tablets compared with individual formulations in healthy adults." Curr Med Res Opin **21**(5): 769-776.	Full text
393	Ieiri, I., H. Takane and K. Otsubo (2004). "The MDR1 (ABCB1) gene polymorphism and its clinical implications." Clin Pharmacokinet **43**(9): 553-576.	Title

394	Izumi, S., Y. Nozaki, H. Kusuhara, K. Hotta, T. Mochizuki, T. Komori, K. Maeda and Y. Sugiyama (2018). "Relative Activity Factor (RAF)-Based Scaling of Uptake Clearance Mediated by Organic Anion Transporting Polypeptide (OATP) 1B1 and OATP1B3 in Human Hepatocytes." Mol Pharm **15**(6): 2277-2288.	Title
395	Izzo, A. A. (2004). "Drug interactions with St. John's Wort (Hypericum perforatum): a review of the clinical evidence." Int J Clin Pharmacol Ther **42**(3): 139-148.	Title
396	Jang, S. Y., M. A. Dooley and M. S. Joy (2013). "Impact of severe hypothyroidism on cyclophosphamide disposition and routes of metabolism and transport in a patient with treatment-resistant lupus nephritis." Ann Pharmacother **47**(7-8): e35.	Title
397	Júnior, M. A., A. C. de Faria, S. Velozo Eda, T. Dalla Costa, F. P. de Andrade and W. V. de Castro (2015). "Determination of fexofenadine in Hank's balanced salt solution by high-performance liquid chromatography with ultraviolet detection: application to Caco-2 cell permeability studies." Biomed Chromatogr **29**(4): 537-544.	Abstract
398	Kalliokoski, A. and M. Niemi (2009). "Impact of OATP transporters on pharmacokinetics." Br J Pharmacol **158**(3): 693-705.	Title
399	Kataoka, M., T. Yokoyama, Y. Masaoka, S. Sakuma and S. Yamashita (2011). "Estimation of P-glycoprotein-mediated efflux in the oral absorption of P-gp substrate drugs from simultaneous analysis of drug dissolution and permeation." Eur J Pharm Sci **44**(4): 544-551.	Title
400	Kharasch, E. D., C. Hoffer, D. Whittington, A. Walker and P. S. Bedynek (2009). "Methadone pharmacokinetics are independent of cytochrome P4503A (CYP3A) activity and gastrointestinal drug transport: insights from methadone interactions with ritonavir/indinavir." Anesthesiology **110**(3): 660-672.	Abstract
401	Kharasch, E. D., D. Whittington, D. Ensign, C. Hoffer, P. S. Bedynek, S. Campbell, K. Stubbert, A. Crafford, A. London and T. Kim (2012). "Mechanism of efavirenz influence on methadone pharmacokinetics and pharmacodynamics." Clin Pharmacol Ther **91**(4): 673-684.	Title
402	Kim, R. B., B. F. Leake, E. F. Choo, G. K. Dresser, S. V. Kubba, U. I. Schwarz, A. Taylor, H. G. Xie, J. McKinsey, S. Zhou, L. B. Lan, J. D. Schuetz, E. G. Schuetz and G. R. Wilkinson (2001). "Identification of functionally variant MDR1 alleles among European Americans and African Americans." Clin Pharmacol Ther **70**(2): 189-199.	Title
403	Knutson, T., P. Fridblom, H. Ahlström, A. Magnusson, C. Tannergren and H. Lennernäs (2009). "Increased understanding of intestinal drug permeability determined by the LOC-I-GUT approach using multislice computed tomography." Mol Pharm **6**(1): 2-10.	Title
404	Krishna, R., S. Krishnaswami, B. Kittner, A. J. Sankoh and B. K. Jensen (2004). "The utility of mixed-effects covariate analysis in rapid selection of doses in pediatric subjects: a case study with fexofenadine hydrochloride." Biopharm Drug Dispos **25**(9): 373-387.	Full text

405	Kuwayama, K., H. Miyaguchi, T. Kanamori, K. Tsujikawa, T. Yamamuro, H. Segawa, Y. Okada and Y. T. Iwata (2021). "Development of an improved method to estimate the days of continuous drug ingestion, based on the micro-segmental hair analysis." Drug Test Anal **13**(7): 1295-1304.	Title
406	Lin, H., M. Gebhardt, S. Bian, K. A. Kwon, C. K. Shim, S. J. Chung and D. D. Kim (2007). "Enhancing effect of surfactants on fexofenadine.HCl transport across the human nasal epithelial cell monolayer." Int J Pharm **330**(1-2): 23-31.	Abstract
407	Lloret-Linares, C., Y. Daali, S. Chevret, I. Nieto, F. Molière, P. Courtet, F. Galtier, R. M. Richieri, S. Morange, P. M. Llorca, W. El-Hage, T. Desmidt, F. Haesebaert, P. Vignaud, J. Holtzmann, J. L. Cracowski, M. Leboyer, A. Yrondi, F. Calvas, L. Yon, P. Le Corvoisier, O. Doumy, K. Heron, D. Montange, S. Davani, J. Déglon, M. Besson, J. Desmeules, E. Haffen and F. Bellivier (2017). "Exploring venlafaxine pharmacokinetic variability with a phenotyping approach, a multicentric french-swiss study (MARVEL study)." BMC Pharmacol Toxicol **18**(1): 70.	Title
408	Loi, C. M., M. Young, E. Randinitis, A. Vassos and J. R. Koup (1999). "Clinical pharmacokinetics of troglitazone." Clin Pharmacokinet **37**(2): 91-104.	Title
409	Ma, J. D., S. M. Tsunoda, J. S. Bertino, Jr., M. Trivedi, K. K. Beale and A. N. Nafziger (2010). "Evaluation of in vivo P-glycoprotein phenotyping probes: a need for validation." Clin Pharmacokinet **49**(4): 223-237.	Title
410	Mason, J., R. Reynolds and N. Rao (1999). "The systemic safety of fexofenadine HCl." Clin Exp Allergy **29 Suppl 3**: 163-170; discussion 171-163.	Abstract
411	Matsushima, S., K. Maeda, K. Inoue, K. Y. Ohta, H. Yuasa, T. Kondo, H. Nakayama, S. Horita, H. Kusuhara and Y. Sugiyama (2009). "The inhibition of human multidrug and toxin extrusion 1 is involved in the drug-drug interaction caused by cimetidine." Drug Metab Dispos **37**(3): 555-559.	Title
412	Mattila, M. J. and I. Paakkari (1999). "Variations among non-sedating antihistamines: are there real differences?" Eur J Clin Pharmacol **55**(2): 85-93.	Title
413	McAleer, C. W., A. Pointon, C. J. Long, R. L. Brighton, B. D. Wilkin, L. R. Bridges, N. Narasimhan Sriram, K. Fabre, R. McDougall, V. P. Muse, J. T. Mettetal, A. Srivastava, D. Williams, M. T. Schnepper, J. L. Roles, M. L. Shuler, J. J. Hickman and L. Ewart (2019). "On the potential of in vitro organ-chip models to define temporal pharmacokinetic-pharmacodynamic relationships." Sci Rep **9**(1): 9619.	Title
414	Meltzer, E. O. and S. A. Gillman (2007). "Efficacy of fexofenadine versus desloratadine in suppressing histamine-induced wheal and flare." Allergy Asthma Proc **28**(1): 67-73.	Abstract
415	Moreira, F. L., P. Melli, M. P. Marques, A. Rocha, G. H. B. Nardotto, G. Duarte and V. L. Lanchote (2023). "P-Glycoprotein and Organic Anion Transporter Polypeptide 1B/Breast Cancer Resistance Protein Drug Transporter Activity in Pregnant Women Living With HIV." J Clin Pharmacol **63**(2): 219-227.	Not Accessible

416	Nelson, H. S., R. Reynolds and J. Mason (2000). "Fexofenadine HCl is safe and effective for treatment of chronic idiopathic urticaria." Ann Allergy Asthma Immunol **84**(5): 517-522.	Abstract
417	Niemi, M., K. T. Kivistö, U. Hofmann, M. Schwab, M. Eichelbaum and M. F. Fromm (2005). "Fexofenadine pharmacokinetics are associated with a polymorphism of the SLCO1B1 gene (encoding OATP1B1)." Br J Clin Pharmacol **59**(5): 602-604.	Letter to Editor
418	Nirogi, R. V., V. N. Kandikere, M. Shukla, K. Mudigonda, S. Maurya and P. Komarneni (2006). "Simultaneous quantification of fexofenadine and pseudoephedrine in human plasma by liquid chromatography/tandem mass spectrometry with electrospray ionization: method development, validation and application to a clinical study." Rapid Commun Mass Spectrom **20**(20): 3030-3038.	Full text
419	Nozawa, T., K. Imai, J. Nezu, A. Tsuji and I. Tamai (2004). "Functional characterization of pH-sensitive organic anion transporting polypeptide OATP-B in human." J Pharmacol Exp Ther **308**(2): 438-445.	Abstract
420	Ohura, K. (2020). "[Evaluation of the Oral Absorption of Ester-type Prodrugs]." Yakugaku Zasshi **140**(3): 369-376.	Title
421	Ohura, K., H. Sakamoto, S. Ninomiya and T. Imai (2010). "Development of a novel system for estimating human intestinal absorption using Caco-2 cells in the absence of esterase activity." Drug Metab Dispos **38**(2): 323-331.	Abstract
422	Oleaga, C., A. Riu, S. Rothemund, A. Lavado, C. W. McAleer, C. J. Long, K. Persaud, N. S. Narasimhan, M. Tran, J. Roles, C. A. Carmona-Moran, T. Sasserath, D. H. Elbrecht, L. Kumanchik, L. R. Bridges, C. Martin, M. T. Schnepper, G. Ekman, M. Jackson, Y. I. Wang, R. Note, J. Langer, S. Teissier and J. J. Hickman (2018). "Investigation of the effect of hepatic metabolism on off-target cardiotoxicity in a multi-organ human-on-a-chip system." Biomaterials **182**: 176-190.	Title
423	Petri, N., O. Borga, L. Nyberg, M. Hedeland, U. Bondesson and H. Lennernas (2006). "Effect of erythromycin on the absorption of fexofenadine in the jejunum, ileum and colon determined using local intubation in healthy volunteers." Int J Clin Pharmacol Ther **44**(2): 71-79.	Abstract
424	Petri, N., C. Tannergren, D. Rungstad and H. Lennernäs (2004). "Transport characteristics of fexofenadine in the Caco-2 cell model." Pharm Res **21**(8): 1398-1404.	Title
425	Philpot, E. (1999). "Reply to Gonzalez and Estes." Int J Clin Pharmacol Ther **37**(4): 207-208.	Title
426	Pilch, N. A., M. L. Sell, W. McGhee and R. Venkataramanan (2021). "Important considerations for drugs, nutritional, and herbal supplements in pediatric solid organ transplant recipients." Pediatr Transplant **25**(1): e13881.	Title

427	Pinto, L., F. L. Moreira, G. H. B. Nardotto, R. C. Cavalli, E. C. D. Moisés, G. Duarte and V. L. Lanchote (2020). "Chiral Discrimination of P-glycoprotein in Parturient Women: Effect of Fluoxetine on Maternal-Fetal Fexofenadine Pharmacokinetics." Pharm Res **37**(7): 131.	Full text
428	Rebello, S., S. Zhao, S. Hariry, M. Dahlke, N. Alexander, A. Vapurcuyan, I. Hanna and V. Jarugula (2012). "Intestinal OATP1A2 inhibition as a potential mechanism for the effect of grapefruit juice on aliskiren pharmacokinetics in healthy subjects." Eur J Clin Pharmacol **68**(5): 697-708.	Abstract
429	Reist, C., J. C. Wu, Y. Lilja, J. Mukherjee, D. Gripeos, C. Constantinescu, M. A. Raggi, L. Mercolini and V. Ozdemir (2012). "Ketoconazole-associated preferential increase in dopamine D2 receptor occupancy in striatum compared to pituitary in vivo: role for drug transporters?" J Clin Psychopharmacol **32**(1): 110-113.	Title
430	Ridout, F. and I. Hindmarch (2003). "The effects of acute doses of fexofenadine, promethazine, and placebo on cognitive and psychomotor function in healthy Japanese volunteers." Ann Allergy Asthma Immunol **90**(4): 404-410.	Abstract
431	Roongapinun, S., S. Wajajamreon and S. Fooanant (2004). "Comparative efficacy of wheal-and-flare suppression among various non-sedating antihistamines and the pharmacologic insights to their efficacy." J Med Assoc Thai **87**(5): 551-556.	Title
432	Scholz, I., E. Liakoni, F. Hammann, K. E. Grafinger, U. Duthaler, M. Nagler, S. Krähenbühl and M. Haschke (2021). "Effects of Hypericum perforatum (St John's wort) on the pharmacokinetics and pharmacodynamics of rivaroxaban in humans." Br J Clin Pharmacol **87**(3): 1466-1474.	Title
433	Segall, N., R. E. Grubbe, A. L. Levy, M. J. Maloney, A. S. Nayak, B. Kittner and J. T. Quesada (2008). "Pharmacokinetics, safety and tolerability of an oral suspension of fexofenadine for children with allergic rhinitis." Allergy Asthma Proc **29**(4): 380-385.	Abstract
434	Shimizu, M., K. Fuse, K. Okudaira, R. Nishigaki, K. Maeda, H. Kusuhara and Y. Sugiyama (2005). "Contribution of OATP (organic anion-transporting polypeptide) family transporters to the hepatic uptake of fexofenadine in humans." Drug Metab Dispos **33**(10): 1477-1481.	Abstract
435	Simons, F. E. (2002). "H1-antihistamines in children." Clin Allergy Immunol **17**: 437-464.	Title
436	Simons, F. E. (2002). "Comparative pharmacology of H1 antihistamines: clinical relevance." Am J Med **113 Suppl 9A**: 38s-46s.	Title
437	Simons, F. E., N. A. Silver, X. Gu and K. J. Simons (2002). "Clinical pharmacology of H1-antihistamines in the skin." J Allergy Clin Immunol **110**(5): 777-783.	Abstract

438	Sjögren, E., D. Dahlgren, C. Roos and H. Lennernäs (2015). "Human in vivo regional intestinal permeability: quantitation using site-specific drug absorption data." Mol Pharm **12**(6): 2026-2039.	Title
439	Slater, J. W., A. D. Zechnich and D. G. Haxby (1999). "Second-generation antihistamines: a comparative review." Drugs **57**(1): 31-47.	Title
440	Smith, M. K., R. Bikmetov, S. B. Al Rihani, M. Deodhar, M. Hafermann, P. Dow, J. Turgeon and V. Michaud (2021). "Adverse drug event risk assessment by the virtual addition of COVID-19 repurposed drugs to Medicare and commercially insured patients' drug regimens: A drug safety simulation study." Clin Transl Sci **14**(5): 1799-1809.	Title
441	Spangler, D. L., M. B. Abelson, A. Ober and P. J. Gotnes (2003). "Randomized, double-masked comparison of olopatadine ophthalmic solution, mometasone furoate monohydrate nasal spray, and fexofenadine hydrochloride tablets using the conjunctival and nasal allergen challenge models." Clin Ther **25**(8): 2245-2267.	Abstract
442	Srinivas, N. R. (2016). "The Interesting Case of Acyclovir Delivered Using Chitosan in Humans: Is it a Drug Issue or Formulation Issue?" Pharm Res **33**(3): 543-547.	Title
443	Stanton, M. L., M. S. Joy and R. F. Frye (2010). "Validation and application of a liquid chromatography-tandem mass spectrometric method for quantification of the drug transport probe fexofenadine in human plasma using 96-well filter plates." J Chromatogr B Analyt Technol Biomed Life Sci **878**(3-4): 497-501.	Short Communication
444	Stoltz, M., T. Arumugham, C. Lippert, D. Yu, V. Bhargava, M. Eller and S. Weir (1997). "Effect of food on the bioavailability of fexofenadine hydrochloride (MDL 16455A)." Biopharm Drug Dispos **18**(7): 645-648.	Short Communication
445	Takahata, T., N. Yasui-Furukori, G. Yoshiya, T. Uno, K. Sugawara and T. Tateishi (2004). "Fexofenadine does not affect omeprazole pharmacokinetics: both are putative P-glycoprotein substrates." Basic Clin Pharmacol Toxicol **94**(5): 252-256.	Full text
446	Tashiro, M., Y. Sakurada, K. Iwabuchi, H. Mochizuki, M. Kato, M. Aoki, Y. Funaki, M. Itoh, R. Iwata, D. F. Wong and K. Yanai (2004). "Central effects of fexofenadine and cetirizine: measurement of psychomotor performance, subjective sleepiness, and brain histamine H1-receptor occupancy using 11C-doxepin positron emission tomography." J Clin Pharmacol **44**(8): 890-900.	Abstract
447	Togami, K., Y. Tosaki, S. Chono, K. Morimoto, M. Hayasaka and H. Tada (2013). "Enantioselective uptake of fexofenadine by Caco-2 cells as model intestinal epithelial cells." J Pharm Pharmacol **65**(1): 22-29.	Abstract
448	Uno, T., N. Yasui-Furukori, T. Takahata, K. Sugawara and T. Tateishi (2004). "Liquid chromatographic determination of fexofenadine in human plasma with fluorescence detection." J Pharm Biomed Anal **35**(4): 937-942.	Abstract

449	Vanhove, T., T. Bouillon, H. de Loor, P. Annaert and D. Kuypers (2017). "Fexofenadine, a Putative In Vivo P-glycoprotein Probe, Fails to Predict Clearance of the Substrate Tacrolimus in Renal Recipients." Clin Pharmacol Ther **102**(6): 989-996.	Full text
450	Vyas, F. I., S. Prakash and A. J. Singh (2010). "QTc interval prolongation by fexofenadine in healthy human volunteers and its correlation with plasma levels of fexofenadine: A demonstration of anticlockwise hysteresis." Indian J Pharmacol **42**(6): 366-369.	Abstract
451	Wang, X. Y., M. Lim-Jurado, N. Prepageran, P. Tantilipikorn and Y. Wang de (2016). "Treatment of allergic rhinitis and urticaria: a review of the newest antihistamine drug bilastine." Ther Clin Risk Manag **12**: 585-597.	Title
452	Weinberger, M. and L. Hendeles (2018). "Nonprescription medications for respiratory symptoms: Facts and marketing fictions." Allergy Asthma Proc **39**(3): 169-176.	Title
453	Wilson, A., R. G. Tirona and R. B. Kim (2017). "CYP3A4 Activity is Markedly Lower in Patients with Crohn's Disease." Inflamm Bowel Dis **23**(5): 804-813.	Title
454	Yamane, N., Z. Tozuka, Y. Sugiyama, T. Tanimoto, A. Yamazaki and Y. Kumagai (2007). "Microdose clinical trial: quantitative determination of fexofenadine in human plasma using liquid chromatography/electrospray ionization tandem mass spectrometry." J Chromatogr B Analyt Technol Biomed Life Sci **858**(1-2): 118-128.	Abstract
455	Yoshitomo, A., S. Asano, S. Hozuki, Y. Tamemoto, Y. Shibata, N. Hashimoto, K. Takahashi, Y. Sasaki, N. Ozawa, M. Kageyama, T. Iijima, Y. Kazuki, H. Sato and A. Hisaka (2023). "Significance of Basal Membrane Permeability of Epithelial Cells in Predicting Intestinal Drug Absorption." Drug Metab Dispos **51**(3): 318-328.	Title
456	Yu, J., Z. Zhou, J. Tay-Sontheimer, R. H. Levy and I. Ragueneau-Majlessi (2017). "Intestinal Drug Interactions Mediated by OATPs: A Systematic Review of Preclinical and Clinical Findings." J Pharm Sci **106**(9): 2312-2325.	Title
457	Chen, C. (2007). "Some pharmacokinetic aspects of the lipophilic terfenadine and zwitterionic fexofenadine in humans." Drugs R D **8**(5): 301-314.	Abstract
458	Cvetkovic, M., B. Leake, M. F. Fromm, G. R. Wilkinson and R. B. Kim (1999). "OATP and P-glycoprotein transporters mediate the cellular uptake and excretion of fexofenadine." Drug Metab Dispos **27**(8): 866-871.	Abstract
459	Gotoh, Y., N. Kamada and D. Momose (2005). "The advantages of the Ussing chamber in drug absorption studies." J Biomol Screen **10**(5): 517-523.	Title
460	Grzelewska-Rzymowska, I., M. Pietrzkowicz and M. Górska (2001). "[The effect of second generation histamine antagonists on the heart]." Pneumonol Alergol Pol **69**(3-4): 217-226.	Title

461	Gundogdu, E., I. G. Alvarez and E. Karasulu (2011). "Improvement of effect of water-in-oil microemulsion as an oral delivery system for fexofenadine: in vitro and in vivo studies." Int J Nanomedicine **6**: 1631-1640.	Full text
462	Gundogdu, E., V. Mangas-Sanjuan, I. Gonzalez-Alvarez, M. Bermejo and E. Karasulu (2012). "In vitro-in situ permeability and dissolution of fexofenadine with kinetic modeling in the presence of sodium dodecyl sulfate." Eur J Drug Metab Pharmacokinet **37**(1): 65-75.	Animal
463	Huh, Y., H. J. Cho, I. S. Yoon, M. K. Choi, J. S. Kim, E. Oh, S. J. Chung, C. K. Shim and D. D. Kim (2010). "Preparation and evaluation of spray-dried hyaluronic acid microspheres for intranasal delivery of fexofenadine hydrochloride." Eur J Pharm Sci **40**(1): 9-15.	Abstract
464	Imai, T., S. Ariyoshi, K. Ohura, T. Sawada and Y. Nakada (2016). "Expression of Carboxylesterase Isozymes and Their Role in the Behavior of a Fexofenadine Prodrug in Rat Skin." J Pharm Sci **105**(2): 714-721.	Animal
465	Jiang, S., W. Zhao, Y. Chen, Z. Zhong, M. Zhang, F. Li, P. Xu, K. Zhao, Y. Li, L. Liu and X. Liu (2015). "Paroxetine decreased plasma exposure of glyburide partly via inhibiting intestinal absorption in rats." Drug Metab Pharmacokinet **30**(3): 240-246.	Animal
466	Karibe, T., T. Imaoka, K. Abe and O. Ando (2018). "Curcumin as an In Vivo Selective Intestinal Breast Cancer Resistance Protein Inhibitor in Cynomolgus Monkeys." Drug Metab Dispos **46**(5): 667-679.	Animal
467	MacLean, C., U. Moenning, A. Reichel and G. Fricker (2010). "Regional absorption of fexofenadine in rat intestine." Eur J Pharm Sci **41**(5): 670-674.	Animal
468	Medwid, S., M. M. J. Li, M. J. Knauer, K. Lin, S. E. Mansell, C. L. Schmerk, C. Zhu, K. E. Griffin, M. D. Yousif, G. K. Dresser, U. I. Schwarz, R. B. Kim and R. G. Tirona (2019). "Fexofenadine and Rosuvastatin Pharmacokinetics in Mice with Targeted Disruption of Organic Anion Transporting Polypeptide 2B1." Drug Metab Dispos **47**(8): 832-842.	Animal
469	Ming, X., B. M. Knight and D. R. Thakker (2011). "Vectorial transport of fexofenadine across Caco-2 cells: involvement of apical uptake and basolateral efflux transporters." Mol Pharm **8**(5): 1677-1686.	Abstract
470	Molimard, M., B. Diquet and M. S. Benedetti (2004). "Comparison of pharmacokinetics and metabolism of desloratadine, fexofenadine, levocetirizine and mizolastine in humans." Fundam Clin Pharmacol **18**(4): 399-411.	Review
471	Morimoto, K., M. Sasaki, E. Oikawa, M. Abe, T. Kikuchi, M. Ishii, T. Ogihara and M. Tomita (2021). "Intestinal Absorption of Alogliptin Is Mediated by a Fruit-Juice-Sensitive Transporter." Biol Pharm Bull **44**(5): 653-658.	Title

472	Nakayama, K., H. Kamimura, H. Suemizu, N. Yoneda, M. Nishiwaki, K. Iwamoto, M. Mizunaga, T. Negoro, S. Ito, H. Yamazaki and Y. Nomura (2020). "Predicted values for human total clearance of a variety of typical compounds with differently humanized-liver mouse plasma data." Drug Metab Pharmacokinet **35**(4): 389-396.	Animal
473	Ohura, K., Y. Nakada, S. Kotani and T. Imai (2015). "Design of Fexofenadine Prodrugs Based on Tissue-Specific Esterase Activity and Their Dissimilar Recognition by P-Glycoprotein." J Pharm Sci **104**(9): 3076-3083.	Abstract
474	Ono, A. and K. Sugano (2014). "Application of the BCS biowaiver approach to assessing bioequivalence of orally disintegrating tablets with immediate release formulations." Eur J Pharm Sci **64**: 37-43.	Title
475	Tomono, T., K. Otsuka, K. Yano, M. Kanagawa, H. Arakawa and T. Ogihara (2015). "Recommended Daily Dose of Sesame Lignans Has No Influence on Oral Absorption of P-Glycoprotein Substrates in Rats." Biol Pharm Bull **38**(12): 1960-1963.	Animal
476	(2003). Chapter 1 Role of bioanalysis in pharmaceutical drug development. Progress in Pharmaceutical and Biomedical Analysis. D. A. Wells, Elsevier. **5:** 1-40.	Title
477	Abdelhameed, A. H., W. A. Abdelhafez, k. Saleh and M. S. Mohamed (2022). "Formulation, optimization, and in-vivo evaluation of nanostructured lipid carriers loaded with Fexofenadine HCL for oral delivery." Journal of Drug Delivery Science and Technology **74**: 103607.	Not Accessible
478	Abdelhameed, A. H., W. A. Abdelhafez, k. I. Saleh, A. A. Hamad and M. S. Mohamed (2023). "Formulation and optimization of oral fast dissolving films loaded with nanosuspension to enhance the oral bioavailability of Fexofenadine HCL." Journal of Drug Delivery Science and Technology **85**: 104578.	Abstract
479	Abdelkawy, K. S., S. M. El-Haggar, D. H. Ziada, N. F. Ebaid, M. A. El-Magd and F. A. Elbarbry (2020). "The effect of genetic variations on ribavirin pharmacokinetics and treatment response in HCV-4 Egyptian patients receiving sofosbuvir/daclatasvir and ribavirin." Biomedicine & Pharmacotherapy **121**: 109657.	Title
480	Abelson, M. B., L. Smith and M. Chapin (2003). "Ocular Allergic Disease: Mechanisms, Disease Sub-types, Treatment." The Ocular Surface **1**(3): 127-149.	Title
481	Ahmed, M. S., A. B. Farag, I. N. Boys, P. Wang, I. Menendez-Montes, N. U. N. Nguyen, J. L. Eitson, M. B. Ohlson, W. Fan, M. B. McDougal, K. Mar, S. Thet, F. Ortiz, S. Y. Kim, A. Solmonson, N. S. Williams, A. Lemoff, R. J. DeBerardinis, J. W. Schoggins and H. A. Sadek (2023). "FDA approved drugs with antiviral activity against SARS-CoV-2: From structure-based repurposing to host-specific mechanisms." Biomedicine & Pharmacotherapy **162**: 114614.	Title
482	Al Lawati, H. A. J., A. M. Kadavilpparampu and F. O. Suliman (2014). "Combination of capillary micellar liquid chromatography with on-chip microfluidic chemiluminescence detection for direct analysis of buspirone in human plasma." Talanta **127**: 230-238.	Title

483	Alderman, J. (2005). "Coadministration of scrtraline with cisapride or pimozide: an open-label, nonrandomized examination of pharmacokinetics and corrected qt intervals in healthy adult volunteers." Clinical Therapeutics **27**(7): 1050-1063.	Title
484	Ali Reza, A. S. M., M. Mamunur Rashid, M. Shahidul Islam, M. Amjad Hossen, A. M. Abu Ahmed, M. Anwarul Haque, M. Samima Nasrin, M. Nazim Uddin, J. Khan and M. Atiar Rahman (2023). "Polyphenolics of purple devil fruits potentiate anti-inflammatory action by regulating the expression of inflammatory cytokines and apoptotic signaling molecules evident in extensive and combined experimental models." Journal of Functional Foods **106**: 105610.	Title
485	Alothman, Z. A., N. Bukhari, S. Haider, S. M. Wabaidur and A. A. Alwarthan (2010). "Spectrofluorimetric determination of fexofenadine hydrochloride in pharmaceutical preparation using silver nanoparticles." Arabian Journal of Chemistry **3**(4): 251-255.	Abstract
486	Alvinerie, M., J. Dupuy, S. Kiki-Mvouaka, J.-F. Sutra and A. Lespine (2008). "Ketoconazole increases the plasma levels of ivermectin in sheep." Veterinary Parasitology **157**(1): 117-122.	Animal
487	Ando, H., H. Hatakeyama, H. Sato, A. Hisaka and H. Suzuki (2017). "Determinants of Intestinal Availability for P-glycoprotein Substrate Drugs Estimated by Extensive Simulation With Mathematical Absorption Models." Journal of Pharmaceutical Sciences **106**(9): 2771-2779.	Title
488	Andreas, C. J., X. Pepin, C. Markopoulos, M. Vertzoni, C. Reppas and J. B. Dressman (2017). "Mechanistic investigation of the negative food effect of modified release zolpidem." European Journal of Pharmaceutical Sciences **102**: 284-298.	Title
489	Angst, M. S., L. F. Chu, M. S. Tingle, S. L. Shafer, J. D. Clark and D. R. Drover (2009). "No evidence for the development of acute tolerance to analgesic, respiratory depressant and sedative opioid effects in humans." Pain **142**(1): 17-26.	Title
490	Annaert, P., J. Brouwers, A. Bijnens, F. Lammert, J. Tack and P. Augustijns (2010). "Ex vivo permeability experiments in excised rat intestinal tissue and in vitro solubility measurements in aspirated human intestinal fluids support age-dependent oral drug absorption." European Journal of Pharmaceutical Sciences **39**(1): 15-22.	Title
491	Aoyagi, H., D. Kajiwara, K. Tsunekuni, K. Tanaka, K. Miyoshi and N. Hirasawa (2020). "Potential synergistic effects of novel hematopoietic prostaglandin D synthase inhibitor TAS-205 and different types of anti-allergic medicine on nasal obstruction in a Guinea pig model of experimental allergic rhinitis." European Journal of Pharmacology **875**: 173030.	Title
492	Azzolini, M., A. Mattarei, M. La Spina, M. Fanin, G. Chiodarelli, M. Romio, M. Zoratti, C. Paradisi and L. Biasutto (2017). "New natural amino acid-bearing prodrugs boost pterostilbene’s oral pharmacokinetic and distribution profile." European Journal of Pharmaceutics and Biopharmaceutics **115**: 149-158.	Title

493	Badolo, L., L. M. Rasmussen, H. R. Hansen and C. Sveigaard (2010). "Screening of OATP1B1/3 and OCT1 inhibitors in cryopreserved hepatocytes in suspension." European Journal of Pharmaceutical Sciences **40**(4): 282-288.	Title
494	Bajraktari, G. and J. Weiss (2020). "The aglycone diosmetin has the higher perpetrator drug-drug interaction potential compared to the parent flavone diosmin." Journal of Functional Foods **67**: 103842.	Title
495	Başaran, N., D. Paslı and A. A. Başaran (2022). "Unpredictable adverse effects of herbal products." Food and Chemical Toxicology **159**: 112762.	Title
496	Bedada, S. K., N. R. Yellu and P. Neerati (2016). "Effect of resveratrol on the pharmacokinetics of fexofenadine in rats: Involvement of P-glycoprotein inhibition." Pharmacological Reports **68**(2): 338-343.	Animal
497	Berger, W. E., E. J. Schenkel and L. E. Mansfield (2002). "Safety and efficacy of desloratadine 5 mg in asthma patients with seasonal allergic rhinitis and nasal congestion." Annals of Allergy, Asthma & Immunology **89**(5): 485-491.	Title
498	Berginc, K., J. Trontelj and A. Kristl (2012). "Bio-relevant media to assess drug permeability: Sodium taurocholate and lecithin combination or crude bile?" International Journal of Pharmaceutics **429**(1): 22-30.	Title
499	Bernstein, D. I., W. F. Schoenwetter, R. A. Nathan, W. Storms, R. Ahlbrandt and J. Mason (1997). "Efficacy and Safety of Fexofenadine Hydrochloride for Treatment of Seasonal Allergic Rhinitis." Annals of Allergy, Asthma & Immunology **79**(5): 443-448.	Abstract
500	Bindslev-Jensen, C. (2000). "Do sedating H1-antihistamines still have a role to play in the management of chronic idiopathic urticaria?" Revue Française d'Allergologie et d'Immunologie Clinique **40**(1): 78-81.	Title
501	Bircsak, K. M., R. DeBiasio, M. Miedel, A. Alsebahi, R. Reddinger, A. Saleh, T. Shun, L. A. Vernetti and A. Gough (2021). "A 3D microfluidic liver model for high throughput compound toxicity screening in the OrganoPlate®." Toxicology **450**: 152667.	Title
502	Bosma, R., J. van den Bor, H. F. Vischer, L. Labeaga and R. Leurs (2018). "The long duration of action of the second generation antihistamine bilastine coincides with its long residence time at the histamine H1 receptor." European Journal of Pharmacology **838**: 107-111.	Title
503	Bottoni, P. and S. Caroli (2018). "Presence of residues and metabolites of pharmaceuticals in environmental compartments, food commodities and workplaces: A review spanning the three-year period 2014–2016." Microchemical Journal **136**: 2-24.	Title

504	Bousquet, J. and P. Demoly (2002). Antihistamines (H1 receptor antagonists). Side Effects of Drugs Annual. J. K. Aronson, Elsevier. **25:** 183-188.	Title
505	Bousquet, J., P. van Cauwenberge and N. Khaltaev (2001). "Allergic Rhinitis and Its Impact on Asthma." Journal of Allergy and Clinical Immunology **108**(5, Supplement): S147-S334.	Title
506	Brand, W., M. E. Schutte, G. Williamson, J. J. van Zanden, N. H. P. Cnubben, J. P. Groten, P. J. van Bladeren and I. M. C. M. Rietjens (2006). "Flavonoid-mediated inhibition of intestinal ABC transporters may affect the oral bioavailability of drugs, food-borne toxic compounds and bioactive ingredients." Biomedicine & Pharmacotherapy **60**(9): 508-519.	Title
507	Brant, S. R., C. I. M. Panhuysen, D. Nicolae, D. M. Reddy, D. K. Bonen, R. Karaliukas, L. Zhang, E. Swanson, L. W. Datta, T. Moran, G. Ravenhill, R. H. Duerr, J.-P. Achkar, A. S. Karban and J. H. Cho (2003). "MDR1 Ala893 Polymorphism Is Associated with Inflammatory Bowel Disease." The American Journal of Human Genetics **73**(6): 1282-1292.	Title
508	Bressler, R. and J. J. Bahl (2003). "Principles of Drug Therapy for the Elderly Patient." Mayo Clinic Proceedings **78**(12): 1564-1577.	Title
509	Brudeli, B., L. R. Moltzau, C. H. T. Nguyen, K. W. Andressen, N. O. Nilsen, F. O. Levy and J. Klaveness (2013). "Synthesis and pharmacological properties of a new hydrophilic and orally bioavailable 5-HT4 antagonist." European Journal of Medicinal Chemistry **64**: 629-637.	Title
510	Butler, J., J. A. Ezekowitz, S. P. Collins, M. M. Givertz, J. R. Teerlink, M. N. Walsh, N. M. Albert, C. A. Westlake Canary, P. E. Carson, M. Colvin-Adams, J. C. Fang, A. F. Hernandez, R. E. Hershberger, S. D. Katz, J. G. Rogers, J. A. Spertus, W. G. Stevenson, N. K. Sweitzer, W. H. Wilson Tang, W. G. Stough and R. C. Starling (2012). "Update on Aldosterone Antagonists Use in Heart Failure With Reduced Left Ventricular Ejection Fraction Heart Failure Society of America Guidelines Committee." Journal of Cardiac Failure **18**(4): 265-281.	Title
511	Cacace, F., M. Menci, M. Papi and V. Piemonte (2021). "In-Silico Prediction of Oral Drug Bioavailability: A multi-boluses approach." Medical Engineering & Physics **98**: 140-150.	Title
512	Cai, J., A. Auster, S. Cho and Z. Lai (2023). "Dissecting the human gut microbiome to better decipher drug liability: A once-forgotten organ takes center stage." Journal of Advanced Research **52**: 171-201.	Title
513	Camacho-Muñoz, D., B. Kasprzyk-Hordern and K. V. Thomas (2016). "Enantioselective simultaneous analysis of selected pharmaceuticals in environmental samples by ultrahigh performance supercritical fluid based chromatography tandem mass spectrometry." Analytica Chimica Acta **934**: 239-251.	Title
514	Camacho-Muñoz, D., B. Petrie, L. Lopardo, K. Proctor, J. Rice, J. Youdan, R. Barden and B. Kasprzyk-Hordern (2019). "Stereoisomeric profiling of chiral pharmaceutically active compounds in wastewaters and the receiving environment – A catchment-scale and a laboratory study." Environment International **127**: 558-572.	Title

515	Cantrill, C. and J. B. Houston (2017). "Understanding the Interplay Between Uptake and Efflux Transporters Within In Vitro Systems in Defining Hepatocellular Drug Concentrations." Journal of Pharmaceutical Sciences **106**(9): 2815-2825.	Title
516	Cardelús, I., F. Antón, J. Beleta and J. M. Palacios (1999). "Anticholinergic effects of desloratadine, the major metabolite of loratadine, in rabbit and guinea-pig iris smooth muscle." European Journal of Pharmacology **374**(2): 249-254.	Animal
517	Casale, T. B., M. S. Blaiss, E. Gelfand, T. Gilmore, P. D. Harvey, I. Hindmarch, F. E. R. Simons, D. L. Spangler, S. J. Szefler, T. E. Terndrup, S. A. Waldman, J. Weiler and D. F. Wong (2003). "First do no harm: Managing antihistamine impairment in patients with allergic rhinitis." Journal of Allergy and Clinical Immunology **111**(5): S835-S842.	Title
518	Ceolotto, N., P. Dollamore, A. Hold, B. Balne, K. K. Jagadeesan, R. Standerwick, M. Robertson, R. Barden and B. Kasprzyk-Hordern (2024). "A new Wastewater-Based Epidemiology workflow to estimate community wide non-communicable disease prevalence using pharmaceutical proxy data." Journal of Hazardous Materials **461**: 132645.	Abstract
519	Chang, H.-Y., C.-J. Chen, W.-C. Ma, W.-K. Cheng, Y.-N. Lin, Y.-R. Lee, J.-J. Chen and Y.-P. Lim (2017). "Modulation of pregnane X receptor (PXR) and constitutive androstane receptor (CAR) activation by ursolic acid (UA) attenuates rifampin-isoniazid cytotoxicity." Phytomedicine **36**: 37-49.	Title
520	Chen, J., Q. Su, J. Qin, Y. Zhou, H. Ruan, Z. Chen, Z. Chen, H. Li, Y. Zhou, S. Zhou, X. Wang, L. Zhou and M. Huang (2019). "Correlation of MCT1 and ABCC2 gene polymorphisms with valproic acid resistance in patients with epilepsy on valproic acid monotherapy." Drug Metabolism and Pharmacokinetics **34**(3): 165-171.	Title
521	Chen, X., H. Zhao, P. Hatsis and J. Amin (2012). "Investigation of dried blood spot card-induced interferences in liquid chromatography/mass spectrometry." Journal of Pharmaceutical and Biomedical Analysis **61**: 30-37.	Title
522	Chiang, P.-C., J. Liu, P. Fan and H. Wong (2018). "Exploring a Kinetic Model Approach in Biopharmaceutics: Estimating the Fraction Absorbed of Orally Administered Drugs in Humans." Journal of Pharmaceutical Sciences **107**(7): 1798-1805.	Title
523	Cho, H.-J., P. Balakrishnan, E.-K. Park, K.-W. Song, S.-S. Hong, T.-Y. Jang, K.-S. Kim, S.-J. Chung, C.-K. Shim and D.-D. Kim (2011). "Poloxamer/Cyclodextrin/Chitosan-Based Thermoreversible Gel for Intranasal Delivery of Fexofenadine Hydrochloride." Journal of Pharmaceutical Sciences **100**(2): 681-691.	Animal
524	Cornet-Masana, J. M., A. Banús-Mulet, J. M. Carbó, M. Á. Torrente, F. Guijarro, L. Cuesta-Casanovas, J. Esteve and R. M. Risueño (2019). "Dual lysosomal-mitochondrial targeting by antihistamines to eradicate leukaemic cells." EBioMedicine **47**: 221-234.	Other language
525	Cox, L., H. Nelson, R. Lockey, C. Calabria, T. Chacko, I. Finegold, M. Nelson, R. Weber, D. I. Bernstein, J. Blessing-Moore, D. A. Khan, D. M. Lang, R. A. Nicklas, J. Oppenheimer, J. M. Portnoy, C. Randolph, D. E. Schuller, S. L. Spector, S. Tilles and D. Wallace (2011). "Allergen immunotherapy: A practice parameter third update." Journal of Allergy and Clinical Immunology **127**(1, Supplement): S1-S55.	Title

526	Crampton, H. J. (2003). "Comparison of ketotifen fumarate ophthalmic solution alone, desloratadine alone, and their combination for inhibition of the signs and symptoms of seasonal allergic rhinoconjunctivitis in the conjunctival allergen challenge model: a double-masked, placebo- and active-controlled trial." Clinical Therapeutics **25**(7): 1975-1987.	Title
527	Criscione, J., Z. Rezaei, C. M. Hernandez Cantu, S. Murphy, S. R. Shin and D.-H. Kim (2023). "Heart-on-a-chip platforms and biosensor integration for disease modeling and phenotypic drug screening." Biosensors and Bioelectronics **220**: 114840.	Title
528	Cruz, A. C., A. H. S. Pinto, C. D. D. Costa, L. P. Oliveira, J. R. Oliveira-Neto and L. C. Cunha (2021). "Food-Effect on (−) – Hydroxycitric Acid Absorption After Oral Administration of Garcinia cambogia Extract Formulation: a Phase I, Randomized, Cross-Over Study." Journal of Pharmaceutical Sciences **110**(2): 693-697.	Title
529	Dagenais, C., A. Avdeef, O. Tsinman, A. Dudley and R. Beliveau (2009). "P-glycoprotein deficient mouse in situ blood–brain barrier permeability and its prediction using an in combo PAMPA model." European Journal of Pharmaceutical Sciences **38**(2): 121-137.	Title
530	Dahan, A., B. T. West and G. L. Amidon (2009). "Segmental-dependent membrane permeability along the intestine following oral drug administration: Evaluation of a triple single-pass intestinal perfusion (TSPIP) approach in the rat." European Journal of Pharmaceutical Sciences **36**(2): 320-329.	Animal
531	Darney, K., L. Turco, F. M. Buratti, E. Di Consiglio, S. Vichi, A. C. Roudot, C. Béchaux, E. Testai, J. L. C. M. Dorne and L. S. Lautz (2020). "Human variability in influx and efflux transporters in relation to uncertainty factors for chemical risk assessment." Food and Chemical Toxicology **140**: 111305.	Title
532	Das, A. K., S. Yoshimura, R. Mishima, K. Fujimoto, H. Mizuguchi, S. Dev, Y. Wakayama, Y. Kitamura, S. Horio, N. Takeda and H. Fukui (2007). "Stimulation of Histamine H1 Receptor Up-Regulates Histamine H1 Receptor Itself Through Activation of Receptor Gene Transcription." Journal of Pharmacological Sciences **103**(4): 374-382.	Title
533	Dave, R. A. and M. E. Morris (2016). "Novel high/low solubility classification methods for new molecular entities." International Journal of Pharmaceutics **511**(1): 111-126.	Title
534	Day, J. (1999). "Pros and cons of the use of antihistamines in managing allergic rhinitis." Journal of Allergy and Clinical Immunology **103**(3, Supplement): S395-S399.	Title
535	de Leon, J. (2015). "The effects of antiepileptic inducers in neuropsychopharmacology, a neglected issue. Part II: Pharmacological issues and further understanding." Revista de Psiquiatría y Salud Mental (English Edition) **8**(3): 167-188.	Title
536	De Vocht, T., C. Buyck, N. Deferm, B. Qi, P. Van Brantegem, H. van Vlijmen, J. Snoeys, E. Hoeben, A. Vermeulen and P. Annaert (2021). "Identification of novel inhibitors of rat Mrp3." European Journal of Pharmaceutical Sciences **162**: 105813.	Animal

537	Deferme, S., R. Mols, W. V. Driessche and P. Augustijns (2002). "Apricot Extract Inhibits the P-gp–Mediated Efflux of Talinolol." Journal of Pharmaceutical Sciences **91**(12): 2539-2548.	Title
538	Demeester, C., D. Robins, A. E. Edwina, J. Tournoy, P. Augustijns, I. Ince, A. Lehmann, M. Vertzoni and J. F. Schlender (2023). "Physiologically based pharmacokinetic (PBPK) modelling of oral drug absorption in older adults – an AGePOP review." European Journal of Pharmaceutical Sciences **188**: 106496.	Title
539	Doshi, U. and A. P. Li (2011). "Luciferin IPA–Based Higher Throughput Human Hepatocyte Screening Assays for CYP3A4 Inhibition and Induction." SLAS Discovery **16**(8): 903-909.	Title
540	Dubbelboer, I. R., D. Dahlgren, E. Sjögren and H. Lennernäs (2019). "Rat intestinal drug permeability: A status report and summary of repeated determinations." European Journal of Pharmaceutics and Biopharmaceutics **142**: 364-376.	Animal
541	DuBuske, L. M. (1999). "Second-generation antihistamines: The risk of ventricular arrhythmias." Clinical Therapeutics **21**(2): 281-295.	Title
542	Durmus, S., S. van Hoppe and A. H. Schinkel (2016). "The impact of Organic Anion-Transporting Polypeptides (OATPs) on disposition and toxicity of antitumor drugs: Insights from knockout and humanized mice." Drug Resistance Updates **27**: 72-88.	Animal
543	Dykewicz, M. S., S. Fineman, D. P. Skoner, R. Nicklas, R. Lee, J. Blessing-Moore, J. T. Li, I. L. Bernstein, W. Berger, S. Spector and D. Schuller (1998). "Diagnosis and Management of Rhinitis: Complete Guidelines of the Joint Task Force on Practice Parameters in Allergy, Asthma and Immunology." Annals of Allergy, Asthma & Immunology **81**(5): 478-518.	Title
544	Eagles, S. K., X. Wang, A. S. Gross and A. J. McLachlan (2020). "An improved cytochrome P450 phenotyping cocktail with a simplified and highly sensitive UHPLC-MS/MS assay in human plasma." Journal of Chromatography B **1140**: 122013.	Title
545	Edafiogho, I. O., S. B. Kombian, K. V. V. Ananthalakshmi, N. N. Salama, N. D. Eddington, T. L. Wilson, M. S. Alexander, P. L. Jackson, C. D. Hanson and K. R. Scott (2007). "Enaminones: Exploring Additional Therapeutic Activities." Journal of Pharmaceutical Sciences **96**(10): 2509-2531.	Title
546	Eğilmez, O. K., N. Kökten, A. I. D. Ekici, M. T. Kalcıoğlu, E. Yesilada and M. Tekin (2015). "The effect of Hypericum perforatum L. (St. John's Wort) on prevention of myringosclerosis after myringotomy in a rat model." International Journal of Pediatric Otorhinolaryngology **79**(7): 1128-1134.	Animal
547	Estelle, F. and R. Simons (1999). "Clinical pharmacology of new H1-receptor antagonists, and why it matters." Revue Française d'Allergologie et d'Immunologie Clinique **39**(1): 11-14.	Title

548	Fagerberg, J. H., E. Sjögren and C. A. S. Bergström (2015). "Concomitant intake of alcohol may increase the absorption of poorly soluble drugs." European Journal of Pharmaceutical Sciences **67**: 12-20.	Title
549	Fan, X., J. Bai, M. Hu, Y. Xu, S. Zhao, Y. Sun, B. Wang, J. Hu and Y. Li (2020). "Drug interaction study of flavonoids toward OATP1B1 and their 3D structure activity relationship analysis for predicting hepatoprotective effects." Toxicology **437**: 152445.	Title
550	Faulx, M. D. and G. S. Francis (2008). "Adverse Drug Reactions in Patients with Cardiovascular Disease." Current Problems in Cardiology **33**(12): 703-768.	Title
551	Fearn, R. A. and B. H. Hirst (2006). "Predicting oral drug absorption and hepatobiliary clearance: Human intestinal and hepatic in vitro cell models." Environmental Toxicology and Pharmacology **21**(2): 168-178.	Title
552	Fellay, J., C. Marzolini, E. R. Meaden, D. J. Back, T. Buclin, J.-P. Chave, L. A. Decosterd, H. Furrer, M. Opravil, G. Pantaleo, D. Retelska, L. Ruiz, A. H. Schinkel, P. Vernazza, C. B. Eap and A. Telenti (2002). "Response to antiretroviral treatment in HIV-1-infected individuals with allelic variants of the multidrug resistance transporter 1: a pharmacogenetics study." The Lancet **359**(9300): 30-36.	Title
553	Fick, J., R. H. Lindberg, M. Tysklind and D. G. J. Larsson (2010). "Predicted critical environmental concentrations for 500 pharmaceuticals." Regulatory Toxicology and Pharmacology **58**(3): 516-523.	Title
554	Fromm, M. F. (2002). "The influence of MDR1 polymorphisms on P-glycoprotein expression and function in humans." Advanced Drug Delivery Reviews **54**(10): 1295-1310.	Title
555	Fu, I., E. J. Woolf and B. K. Matuszewski (2004). "Determination of fexofenadine in human plasma using 96-well solid phase extraction and HPLC with tandem mass spectrometric detection." Journal of Pharmaceutical and Biomedical Analysis **35**(4): 837-846.	Abstract
556	Galatsis, P. (1997). Chapter 31. To Market, To Market - 1996. Annual Reports in Medicinal Chemistry. J. A. Bristol, Academic Press. **32:** 305-326.	Title
557	Garrison, K. L., S. Sahin and L. Z. Benet (2015). "Few Drugs Display Flip-Flop Pharmacokinetics and These Are Primarily Associated with Classes 3 and 4 of the BDDCS." Journal of Pharmaceutical Sciences **104**(9): 3229-3235.	Title
558	Gavhane, Y. N. and A. V. Yadav (2012). "Loss of orally administered drugs in GI tract." Saudi Pharmaceutical Journal **20**(4): 331-344.	Title
559	Geha, R. S. and E. O. Meltzer (2001). "Desloratadine: A new, nonsedating, oral antihistamine." Journal of Allergy and Clinical Immunology **107**(4): 751-762.	Title

560	Genovese, I., A. Ilari, Y. G. Assaraf, F. Fazi and G. Colotti (2017). "Not only P-glycoprotein: Amplification of the ABCB1-containing chromosome region 7q21 confers multidrug resistance upon cancer cells by coordinated overexpression of an assortment of resistance-related proteins." Drug Resistance Updates **32**: 23-46.	Title
561	Gentile, D. A., G. A. Friday and D. P. Skoner (2000). "MANAGEMENT OF ALLERGIC RHINITIS: Antihistamines and Decongestants." Immunology and Allergy Clinics of North America **20**(2): 355-368.	Title
562	Georgitis, J. W., E. O. Meltzer, M. Kaliner, J. Weiler and R. Berkowitz (2000). "Onset-of-action for antihistamine and decongestant combinations during an outdoor challenge." Annals of Allergy, Asthma & Immunology **84**(4): 451-459.	Title
563	Ghamat, S. N. and Z. Talebpour (2020). "A vinylpyrrolidone-based thin film microextraction in combination with direct solid-state spectrofluorimetry for determination of sartans in human plasma." Analytica Chimica Acta **1124**: 146-155.	Title
564	Ghosal, A., R. Ramanathan, N. S. Kishnani, S. K. Chowdhury and K. B. Alton (2005). Chapter 12 Cytochrome p450 (cyp) and udp-glucuronosyltransferase (ugt) enzymes: role in drug metabolism, polymorphism, and identification of their involvement in drug metabolism. Progress in Pharmaceutical and Biomedical Analysis. S. K. Chowdhury, Elsevier. **6:** 295-336.	Title
565	Gibson, J. K., Y. Yue, J. Bronson, C. Palmer and R. Numann (2014). "Human stem cell-derived cardiomyocytes detect drug-mediated changes in action potentials and ion currents." Journal of Pharmacological and Toxicological Methods **70**(3): 255-267.	Title
566	Gispert, J., R. Antonijoan, M. Barbanoj, I. Gich, E. Garcia, R. Esbrí and X. Luria (2002). "Efficacy of ebastine, cetirizine, and loratadine in histamine cutaneous challenges." Annals of Allergy, Asthma & Immunology **89**(3): 259-264.	Title
567	Gloor, Y., C. Lloret-Linares, M. Bosilkovska, N. Perroud, H. Richard-Lepouriel, J. M. Aubry, Y. Daali, J. A. Desmeules and M. Besson (2022). "Drug metabolic enzyme genotype-phenotype discrepancy: High phenoconversion rate in patients treated with antidepressants." Biomedicine & Pharmacotherapy **152**: 113202.	Title
568	Gniazdowska, E., J. Giebułtowicz and P. J. Rudzki (2023). "How does the order of sample analysis influence the matrix effect during LC-MS bioanalysis?" Journal of Chromatography B **1227**: 123800.	Title
569	Godyń, J., D. Gucwa, T. Kobrlova, M. Novak, O. Soukup, B. Malawska and M. Bajda (2020). "Novel application of capillary electrophoresis with a liposome coated capillary for prediction of blood-brain barrier permeability." Talanta **217**: 121023.	Title
570	Golding, J. F. (2006). "Motion sickness susceptibility." Autonomic Neuroscience **129**(1): 67-76.	Title

571	Gor, P., Y. Alnouti and G. A. Reed (2011). "Buspirone, fexofenadine, and omeprazole: Quantification of probe drugs and their metabolites in human plasma." Journal of Pharmaceutical and Biomedical Analysis **55**(5): 1127-1135.	Abstract
572	Grabic, R., J. Fick, R. H. Lindberg, G. Fedorova and M. Tysklind (2012). "Multi-residue method for trace level determination of pharmaceuticals in environmental samples using liquid chromatography coupled to triple quadrupole mass spectrometry." Talanta **100**: 183-195.	Title
573	Grabicová, K., R. Grabic, G. Fedorova, A. Vojs Staňová, M. Bláha, T. Randák, B. W. Brooks and V. Žlábek (2020). "Water reuse and aquaculture: Pharmaceutical bioaccumulation by fish during tertiary treatment in a wastewater stabilization pond." Environmental Pollution **267**: 115593.	Title
574	Greaves, M. W. (2001). "ANTIHISTAMINES." Dermatologic Clinics **19**(1): 53-62.	Title
575	Gupta, A. K., H. I. Katz and N. H. Shear (1999). "Drug interactions with itraconazole, fluconazole, and terbinafine and their management." Journal of the American Academy of Dermatology **41**(2): 237-249.	Title
576	Han, H., T. Akiyoshi, T. Morita, H. Kataoka, K. Katayama, K. Yajima, A. Imaoka and H. Ohtani (2022). "Comparison of the transport kinetics of fexofenadine and its pH dependency among OATP1A2 genetic variants." Drug Metabolism and Pharmacokinetics **47**: 100470.	Title
577	Hanning, S. M., F. L. Lopez, I. C. K. Wong, T. B. Ernest, C. Tuleu and M. Orlu Gul (2016). "Patient centric formulations for paediatrics and geriatrics: Similarities and differences." International Journal of Pharmaceutics **512**(2): 355-359.	Title
578	Harmsen, S., I. Meijerman, R. F. Maas-Bakker, J. H. Beijnen and J. H. M. Schellens (2013). "PXR-mediated P-glycoprotein induction by small molecule tyrosine kinase inhibitors." European Journal of Pharmaceutical Sciences **48**(4): 644-649.	Title
579	Hassan, N. J., D. J. Pountney, C. Ellis and D. E. Mossakowska (2006). "BacMam recombinant baculovirus in transporter expression: A study of BCRP and OATP1B1." Protein Expression and Purification **47**(2): 591-598.	Title
580	Hawksworth, G. M. (2003). "Drug Metabolism." Medicine **31**(8): 16-17.	Title
581	Herédi-Szabó, K., J. E. Palm, T. B. Andersson, Á. Pál, D. Méhn, Z. Fekete, E. Beéry, K. T. Jakab, M. Jani and P. Krajcsi (2013). "A P-gp vesicular transport inhibition assay – Optimization and validation for drug–drug interaction testing." European Journal of Pharmaceutical Sciences **49**(4): 773-781.	Title

582	Hersh, E. V. (1999). "ADVERSE DRUG INTERACTIONS IN DENTAL PRACTICE: INTERACTIONS INVOLVING ANTIBIOTICS: PART II OF A SERIES." The Journal of the American Dental Association **130**(2): 236-251.	Title
583	Hill, S. L. and J. H. Krouse (2003). "The effects of montelukast on intradermal wheal and flare." Otolaryngology - Head and Neck Surgery **129**(3): 199-203.	Title
584	Hishinuma, S., Y. Sato, Y. Kobayashi, H. Komazaki and M. Saito (2008). "Intact Cell Binding for In Vitro Prediction of Sedative and Non-sedative Histamine H1–Receptor Antagonists Based on Receptor Internalization." Journal of Pharmacological Sciences **107**(1): 66-79.	Title
585	Hithamani, G. and K. Srinivasan (2017). "Bioavailability of finger millet (Eleusine coracana) phenolic compounds in rat as influenced by co-administered piperine." Food Bioscience **19**: 101-109.	Title
586	Ho, R. J. Y. and J. Y. Chien (2009). "Drug delivery trends in clinical trials and translational medicine: Updated analysis of ClinicalTrials.gov database." Journal of Pharmaceutical Sciences **98**(6): 1928-1934.	Title
587	Ho, R. J. Y. and J. Y. Chien (2012). "Drug Delivery Trends in Clinical Trials and Translational Medicine: Growth in Biologic Molecule Development and Impact on Rheumatoid Arthritis, Crohn's Disease, and Colitis." Journal of Pharmaceutical Sciences **101**(8): 2668-2674.	Title
588	Hochman, J., C. Tang and T. Prueksaritanont (2015). "Drug–Drug Interactions Related to Altered Absorption and Plasma Protein Binding: Theoretical and Regulatory Considerations, and an Industry Perspective." Journal of Pharmaceutical Sciences **104**(3): 916-929.	Title
589	Hop, C. E. C. A. and C. Prakash (2005). Chapter 6 Metabolite identification by lc-ms: applications in drug discovery and development. Progress in Pharmaceutical and Biomedical Analysis. S. K. Chowdhury, Elsevier. **6:** 123-158.	Title
590	Hu, D., X. Chen, D. Li, H. Zhang, Y. Duan and Y. Huang (2023). "Tranilast-matrine co-amorphous system: Strong intermolecular interactions, improved solubility, and physiochemical stability." International Journal of Pharmaceutics **635**: 122707.	Title
591	Hu, D., Y. Wang, Z. Chen, Z. Ma, Q. You, X. Zhang, Q. Liang, H. Tan, C. Xiao, X. Tang and Y. Gao (2015). "The protective effect of piperine on dextran sulfate sodium induced inflammatory bowel disease and its relation with pregnane X receptor activation." Journal of Ethnopharmacology **169**: 109-123.	Title
592	Huang, K., J. Hu, X. Li and Y. Li (2012). "Role of P-Glycoprotein in Intestinal Absorption of FB2, a Promising Abl/Src Dual Tyrosine Kinase Inhibitor." Drug Metabolism and Pharmacokinetics **27**(5): 486-494.	Title

593	Huang, X., Z. Wu, X. Qiaolongbatu, H. Qu, C. Ma, Y. Xiang, M. Jia, Y. Liu, S. Wang, L. Jing, X. Jiang, Y. Lou and G. Fan (2023). "Recycling preparative isolation of six bicyclol active metabolites from SD rat urine using macroporous resin, offline 2D LPLC/HPLC, and prep-HPLC combined with pharmacodynamic evaluation of two active metabolites." Arabian Journal of Chemistry **16**(10): 105107.	Title
594	Huh, Y., H.-J. Cho, I.-S. Yoon, M.-K. Choi, J. S. Kim, E. Oh, S.-J. Chung, C.-K. Shim and D.-D. Kim (2010). "Preparation and evaluation of spray-dried hyaluronic acid microspheres for intranasal delivery of fexofenadine hydrochloride." European Journal of Pharmaceutical Sciences **40**(1): 9-15.	Abstract
595	Hussner, J., A. Foletti, I. Seibert, A. Fuchs, E. Schuler, V. Malagnino, M. Grube and H. E. Meyer zu Schwabedissen (2021). "Differences in transport function of the human and rat orthologue of the Organic Anion Transporting Polypeptide 2B1 (OATP2B1)." Drug Metabolism and Pharmacokinetics **41**: 100418.	Animal
596	Hyo, S., S. Fujieda, R. Kawada, S. Kitazawa and H. Takenaka (2005). "The efficacy of short-term administration of 3 antihistamines vs placebo under natural exposure to Japanese cedar pollen." Annals of Allergy, Asthma & Immunology **94**(4): 457-464.	Title
597	Ikeda, M., D. Tsuji, K. Yamamoto, Y.-I. Kim, T. Daimon, Y. Iwabe, M. Hatori, R. Makuta, H. Hayashi, K. Inoue, H. Nakamichi, M. Shiokawa and K. Itoh (2015). "Relationship between ABCB1 gene polymorphisms and severe neutropenia in patients with breast cancer treated with doxorubicin/cyclophosphamide chemotherapy." Drug Metabolism and Pharmacokinetics **30**(2): 149-153.	Title
598	Imhof, A. (2003). Miscellaneous antibacterial drugs. Side Effects of Drugs Annual. J. K. Aronson, Elsevier. **26:** 271-301.	Title
599	Incaudo, G. A. (2000). "THE DIAGNOSIS AND TREATMENT OF RHINOSINUSITIS DURING PREGNANCY AND LACTATION." Radiologic Clinics of North America **20**(4): 807-830.	Title
600	Itohda, A., K. Tsutsumi, H. Imai, M. Iwao, T. Kotegawa and K. Ohashi (2012). "Determination of celiprolol in human plasma using high performance liquid chromatography with fluorescence detection for clinical application." Journal of Chromatography B **904**: 88-92.	Title
601	Izabel da Silva Hage-Melim, L., J. G. Curtolo Poiani, C. H. Tomich de Paula da Silva and F. Boylan (2019). "In silico study of the mechanism of action, pharmacokinetic and toxicological properties of some N-methylanthranilates and their analogs." Food and Chemical Toxicology **131**: 110556.	Title
602	Izumi, S., Y. Nozaki, T. Komori, O. Takenaka, K. Maeda, H. Kusuhara and Y. Sugiyama (2017). "Comparison of the Predictability of Human Hepatic Clearance for Organic Anion Transporting Polypeptide Substrate Drugs Between Different In Vitro–In Vivo Extrapolation Approaches." Journal of Pharmaceutical Sciences **106**(9): 2678-2687.	Abstract
603	Jaanus, P. D. S. D. (1999). "Ocular inflammatory disease: pharmacologic update." Clinical Eye and Vision Care **11**(1): 3-12.	Title

604	Jantzen, C. E., F. Toor, K. A. Annunziato and K. R. Cooper (2017). "Effects of chronic perfluorooctanoic acid (PFOA) at low concentration on morphometrics, gene expression, and fecundity in zebrafish (Danio rerio)." Reproductive Toxicology **69**: 34-42.	Title
605	Jeong, S.-H., J.-H. Jang and Y.-B. Lee (2023). "P-glycoprotein mechanical functional analysis using in silico molecular modeling: Pharmacokinetic variability according to ABCB1 c.2677G > T/A genetic polymorphisms." International Journal of Biological Macromolecules **249**: 126777.	Abstract
606	Jeong, W., J. H. Doroshow and S. Kummar (2013). "United States Food and Drug Administration approved oral kinase inhibitors for the treatment of malignancies." Current Problems in Cancer **37**(3): 110-144.	Title
607	Jeong, Y.-S. and W. J. Jusko (2023). "A Complete Extension of Classical Hepatic Clearance Models Using Fractional Distribution Parameter fd in Physiologically Based Pharmacokinetics." Journal of Pharmaceutical Sciences.	Title
608	Jin, Y., A. Nair and H. W. van Veen (2014). "Multidrug Transport Protein NorM from Vibrio cholerae Simultaneously Couples to Sodium- and Proton-Motive Force*." Journal of Biological Chemistry **289**(21): 14624-14632.	Title
609	Jung, S. J., S. O. Choi, S. Y. Um, J. I. Kim, H. Y. P. Choo, S. Y. Choi and S. Y. Chung (2006). "Prediction of the permeability of drugs through study on quantitative structure–permeability relationship." Journal of Pharmaceutical and Biomedical Analysis **41**(2): 469-475.	Title
610	Juniper, E. F., E. Ståhl, R. L. Doty, F. E. R. Simons, D. B. Allen and P. H. Howarth (2005). "Clinical outcomes and adverse effect monitoring in allergic rhinitis." Journal of Allergy and Clinical Immunology **115**(3, Supplement): S390-S413.	Abstract
611	Kalgutkar, A. S., K. S. Frederick, J. Chupka, B. Feng, S. Kempshall, R. J. Mireles, K. S. Fenner and M. D. Troutman (2009). "N-(3,4-dimethoxyphenethyl)-4-(6,7-dimethoxy-3,4-dihydroisoquinolin-2[1H]-yl)-6,7-dimethoxyquinazolin-2-amine (CP-100,356) as a “chemical knock-out equivalent” to assess the impact of efflux transporters on oral drug absorption in the rat." Journal of Pharmaceutical Sciences **98**(12): 4914-4927.	Animal
612	Kamphuis, A. E. M., L. C. P. Borra, R. van der Hulst, D.-P. Kloos, D. J. Rijken, I. J. Bosman and D. J. Touw (2021). "Postmortem redistribution of morphine in humans: Important variables that might be influencing the central blood/peripheral blood ratio." Forensic Science International **329**: 111094.	Title
613	Karakuş, S., İ. Küçükgüzel and Ş. G. Küçükgüzel (2008). "Development and validation of a rapid RP-HPLC method for the determination of cetirizine or fexofenadine with pseudoephedrine in binary pharmaceutical dosage forms." Journal of Pharmaceutical and Biomedical Analysis **46**(2): 295-302.	Title

614	Karlsson, L., U. Schmitt, M. Josefsson, B. Carlsson, J. Ahlner, F. Bengtsson, F. C. Kugelberg and C. Hiemke (2010). "Blood–brain barrier penetration of the enantiomers of venlafaxine and its metabolites in mice lacking P-glycoprotein." European Neuropsychopharmacology **20**(9): 632-640.	Title
615	Kashihara, Y., I. Ieiri, T. Yoshikado, K. Maeda, M. Fukae, M. Kimura, T. Hirota, S. Matsuki, S. Irie, N. Izumi, H. Kusuhara and Y. Sugiyama (2017). "Small-Dosing Clinical Study: Pharmacokinetic, Pharmacogenomic (SLCO2B1 and ABCG2), and Interaction (Atorvastatin and Grapefruit Juice) Profiles of 5 Probes for OATP2B1 and BCRP." Journal of Pharmaceutical Sciences **106**(9): 2688-2694.	Title
616	Kataoka, M., C. Kojima, K. Ueda, K. Minami, H. Higashino, S. Sakuma, K. Togashi, K. Mutaguchi and S. Yamashita (2016). "Quantitative analysis of pharmacokinetic profiles of verapamil and drug–drug interactions induced by a CYP inhibitor using a stable isotope-labeled compound." Drug Metabolism and Pharmacokinetics **31**(6): 405-410.	Title
617	Kato, Y., A. Y. Lim, C. Sakolish, A. Valdiviezo, H. L. Moyer, P. Hewitt, P. Bajaj, G. Han and I. Rusyn (2022). "Analysis of reproducibility and robustness of OrganoPlate® 2-lane 96, a liver microphysiological system for studies of pharmacokinetics and toxicological assessment of drugs." Toxicology in Vitro **85**: 105464.	Title
618	Kato, Y., T. Miyazaki, T. Kano, T. Sugiura, Y. Kubo and A. Tsuji (2009). "Involvement of influx and efflux transport systems in gastrointestinal absorption of celiprolol." Journal of Pharmaceutical Sciences **98**(7): 2529-2539.	Title
619	Keiser, M., L. Kaltheuner, C. Wildberg, J. Müller, M. Grube, L. I. Partecke, C.-D. Heidecke and S. Oswald (2017). "The Organic Anion–Transporting Peptide 2B1 Is Localized in the Basolateral Membrane of the Human Jejunum and Caco-2 Monolayers." Journal of Pharmaceutical Sciences **106**(9): 2657-2663.	Title
620	Keogh, J. P. and J. R. Kunta (2006). "Development, validation and utility of an in vitro technique for assessment of potential clinical drug–drug interactions involving P-glycoprotein." European Journal of Pharmaceutical Sciences **27**(5): 543-554.	Title
621	Khan, M. Z., R. I. Yousuf, M. H. Shoaib, F. R. Ahmed, M. T. Saleem, F. Siddiqui and S. A. Rizvi (2023). "A hybrid framework of artificial intelligence-based neural network model (ANN) and central composite design (CCD) in quality by design formulation development of orodispersible moxifloxacin tablets: Physicochemical evaluation, compaction analysis, and its in-silico PBPK modeling." Journal of Drug Delivery Science and Technology **82**: 104323.	Title
622	Kido, Y., I. Nanchi, Y. Fusamae, T. Matsuzaki, T. Akazawa, H. Sawada, M. Iwasaki, K. Nishida, E. Tsuchiya and T. Okuda (2022). "Species difference in brain penetration of P-gp and BCRP substrates among monkey, dog and mouse." Drug Metabolism and Pharmacokinetics **42**: 100426.	Animal
623	Kikuchi, A., T. Nozawa, T. Wakasawa, T. Maeda and I. Tamai (2006). "Transporter-mediated Intestinal Absorption of Fexofenadine in Rats." Drug Metabolism and Pharmacokinetics **21**(4): 308-314.	Animal

624	Kim, D.-S., Y. Kim, J.-Y. Jeon and M.-G. Kim (2016). "Effect of Red Ginseng on cytochrome P450 and P-glycoprotein activities in healthy volunteers." Journal of Ginseng Research **40**(4): 375-381.	Abstract
625	Kim, H.-R., S.-W. Park, H.-J. Cho, K.-A. Chae, J.-M. Sung, J.-S. Kim, C. P. Landowski, D. Sun, A. M. Abd El-Aty, G. L. Amidon and H.-C. Shin (2007). "Comparative gene expression profiles of intestinal transporters in mice, rats and humans." Pharmacological Research **56**(3): 224-236.	Animal
626	Kim, J.-E., I.-S. Yoon, H.-J. Cho, D.-H. Kim, Y.-H. Choi and D.-D. Kim (2014). "Emulsion-based colloidal nanosystems for oral delivery of doxorubicin: Improved intestinal paracellular absorption and alleviated cardiotoxicity." International Journal of Pharmaceutics **464**(1): 117-126.	Title
627	Kim, R. B. (2002). "Transporters and xenobiotic disposition." Toxicology **181-182**: 291-297.	Title
628	Kim, S.-J., S. Choi, M. Kim, C. Park, G.-L. Kim, S.-O. Lee, W. Kang and D.-K. Rhee (2018). "Effect of Korean Red Ginseng extracts on drug-drug interactions." Journal of Ginseng Research **42**(3): 370-378.	Title
629	Kimoto, E., Y.-A. Bi, R. E. Kosa, L. M. Tremaine and M. V. S. Varma (2017). "Hepatobiliary Clearance Prediction: Species Scaling From Monkey, Dog, and Rat, and In Vitro–In Vivo Extrapolation of Sandwich-Cultured Human Hepatocytes Using 17 Drugs." Journal of Pharmaceutical Sciences **106**(9): 2795-2804.	Animal
630	Kissei, M., T. Itoh and T. Narawa (2014). "Effect of Epigallocatechin Gallate on Drug Transport Mediated by the Proton-coupled Folate Transporter." Drug Metabolism and Pharmacokinetics **29**(5): 367-372.	Title
631	Kitanaka, J., N. Kitanaka, T. Tatsuta, A. Miyoshi, A. Koumoto, K.-i. Tanaka, N. Nishiyama, Y. Morita and M. Takemura (2010). "Pretreatment with l-histidine produces a shift from methamphetamine-induced stereotypical biting to persistent locomotion in mice." Pharmacology Biochemistry and Behavior **94**(3): 464-470.	Animal
632	Kobori, T., S. Harada, K. Nakamoto and S. Tokuyama (2014). "Mechanisms of P-Glycoprotein Alteration During Anticancer Treatment: Role in the Pharmacokinetic and Pharmacological Effects of Various Substrate Drugs." Journal of Pharmacological Sciences **125**(3): 242-254.	Title
633	Koenen, A., K. Köck, M. Keiser, W. Siegmund, H. K. Kroemer and M. Grube (2012). "Steroid hormones specifically modify the activity of organic anion transporting polypeptides." European Journal of Pharmaceutical Sciences **47**(4): 774-780.	Title
634	Koitabashi, Y., T. Kumai, N. Matsumoto, M. Watanabe, S. Sekine, Y. Yanagida and S. Kobayashi (2006). "Orange juice increased the bioavailability of pravastatin, 3-hydroxy-3-methylglutaryl CoA reductase inhibitor, in rats and healthy human subjects." Life Sciences **78**(24): 2852-2859.	Title

635	Komoto, C., T. Nakamura, T. Sakaeda, D. L. Kroetz, T. Yamada, H. Omatsu, T. Koyama, N. Okamura, I. Miki, T. Tamura, N. Aoyama, M. Kasuga and K. Okumura (2006). "MDR1 Haplotype Frequencies in Japanese and Caucasian, and in Japanese Patients with Colorectal Cancer and Esophageal Cancer." Drug Metabolism and Pharmacokinetics **21**(2): 126-132.	Title
636	Koziolek, M., S. Alcaro, P. Augustijns, A. W. Basit, M. Grimm, B. Hens, C. L. Hoad, P. Jedamzik, C. M. Madla, M. Maliepaard, L. Marciani, A. Maruca, N. Parrott, P. Pávek, C. J. H. Porter, C. Reppas, D. van Riet-Nales, J. Rubbens, M. Statelova, N. L. Trevaskis, K. Valentová, M. Vertzoni, D. V. Čepo and M. Corsetti (2019). "The mechanisms of pharmacokinetic food-drug interactions – A perspective from the UNGAP group." European Journal of Pharmaceutical Sciences **134**: 31-59.	Title
637	Krans, H. M. J. (2004). Insulin, glucagon, and hypoglycemic drugs. Side Effects of Drugs Annual. J. K. Aronson, Elsevier. **27:** 446-464.	Title
638	Kristofco, L. A. and B. W. Brooks (2017). "Global scanning of antihistamines in the environment: Analysis of occurrence and hazards in aquatic systems." Science of The Total Environment **592**: 477-487.	Title
639	Krouse, J. H., P. S. Roland, B. F. Marple, G. M. Wall, M. Hannley, S. Golla and D. Hunsaker (2005). "Optimal Duration of Allergic Rhinitis Clinical Trials." Otolaryngology - Head and Neck Surgery **133**(4): 467-487.	Title
640	Kubota, K., H. Kurebayashi, H. Miyachi, M. Tobe, M. Onishi and Y. Isobe (2011). "Synthesis and structure–activity relationship of tricyclic carboxylic acids as novel anti-histamines." Bioorganic & Medicinal Chemistry **19**(9): 3005-3021.	Title
641	Kunze, A., J. Huwyler, B. Poller, H. Gutmann and G. Camenisch (2014). "In Vitro–In Vivo Extrapolation Method to Predict Human Renal Clearance of Drugs." Journal of Pharmaceutical Sciences **103**(3): 994-1001.	Title
642	Kurkov, S. V., D. E. Madden, D. Carr and T. Loftsson (2012). "The Effect of Parenterally Administered Cyclodextrins on the Pharmacokinetics of Coadministered Drugs." Journal of Pharmaceutical Sciences **101**(12): 4402-4408.	Title
643	Kuroda, T., S.-i. Nagata, Y. Takizawa, N. Tamura, K. Kusano, F. Mizobe and K. Hariu (2013). "Pharmacokinetics and pharmacodynamics of d-chlorpheniramine following intravenous and oral administration in healthy Thoroughbred horses." The Veterinary Journal **197**(2): 433-437.	Animal
644	Kusuhara, H. and Y. Sugiyama (2005). "Active Efflux across the Blood-Brain Barrier: Role of the Solute Carrier Family." NeuroRX **2**(1): 73-85.	Title
645	Kuypers, D. R. J., H. de Jonge, M. Naesens and Y. Vanrenterghem (2010). "A prospective, open-label, observational clinical cohort study of the association between delayed renal allograft function, tacrolimus exposure, and CYP3A5 genotype in adult recipients." Clinical Therapeutics **32**(12): 2012-2023.	Title

646	Lai, M.-L., Y.-E. Tien, Y.-S. Huang and J.-D. Huang (2013). "Studies on pharmacokinetic mechanism of phenytoin resistance in refractory epilepsy." Journal of Pharmaceutical Sciences **102**(9): 3189-3195.	Title
647	Lanevskij, K., J. Dapkunas, L. Juska, P. Japertas and R. Didziapetris (2011). "QSAR Analysis of Blood–Brain Distribution: The Influence of Plasma and Brain Tissue Binding." Journal of Pharmaceutical Sciences **100**(6): 2147-2160.	Title
648	Lappin, G., Y. Shishikura, R. Jochemsen, R. J. Weaver, C. Gesson, J. Brian Houston, B. Oosterhuis, O. J. Bjerrum, G. Grynkiewicz, J. Alder, M. Rowland and C. Garner (2011). "Comparative pharmacokinetics between a microdose and therapeutic dose for clarithromycin, sumatriptan, propafenone, paracetamol (acetaminophen), and phenobarbital in human volunteers." European Journal of Pharmaceutical Sciences **43**(3): 141-150.	Title
649	Layton, A. and D. Thiboutot (2013). "Emerging therapies in rosacea." Journal of the American Academy of Dermatology **69**(6, Supplement 1): S57-S65.	Title
650	Lee, K.-R., H.-J. Maeng, J.-B. Chae, S. Chong, D.-D. Kim, C.-K. Shim and S.-J. Chung (2010). "Lack of a Primary Physicochemical Determinant in the Direct Transport of Drugs to the Brain after Nasal Administration in Rats: Potential Involvement of Transporters in the Pathway." Drug Metabolism and Pharmacokinetics **25**(5): 430-441.	Abstract
651	Leishman, D. J. (2020). "Improving prediction of torsadogenic risk in the CiPA in silico model by appropriately accounting for clinical exposure." Journal of Pharmacological and Toxicological Methods **101**: 106654.	Title
652	Leow, J. W. H., Y. Gu and E. C. Y. Chan (2023). "Investigating the relevance of CYP2J2 inhibition for drugs known to cause intermediate to high risk torsades de pointes." European Journal of Pharmaceutical Sciences **187**: 106475.	Title
653	Li, H., C. Zhang, J. Wang, Y. Jiang, J. P. Fawcett and J. Gu (2010). "Simultaneous quantitation of paracetamol, caffeine, pseudoephedrine, chlorpheniramine and cloperastine in human plasma by liquid chromatography–tandem mass spectrometry." Journal of Pharmaceutical and Biomedical Analysis **51**(3): 716-722.	Title
654	Li, J., C. A. Larregieu and L. Z. Benet (2016). "Classification of natural products as sources of drugs according to the biopharmaceutics drug disposition classification system (BDDCS)." Chinese Journal of Natural Medicines **14**(12): 888-897.	Title
655	Li, W., D. Iusuf, R. W. Sparidans, E. Wagenaar, Y. Wang, D. R. de Waart, M. L. F. Martins, S. van Hoppe, M. C. Lebre, O. van Tellingen, J. H. Beijnen and A. H. Schinkel (2023). "Organic anion-transporting polypeptide 2B1 knockout and humanized mice; insights into the handling of bilirubin and drugs." Pharmacological Research **190**: 106724.	Animal
656	Li, Y., J. Song, N. Tian, J. Cai, M. Huang, Q. Xing, Y. Wang, C. Wu and H. Hu (2014). "Improving oral bioavailability of metformin hydrochloride using water-in-oil microemulsions and analysis of phase behavior after dilution." International Journal of Pharmaceutics **473**(1): 316-325.	Title

657	Li, Z., Y. Zhang, Y. Gao, Y. Xiang, W. Zhang, C. Lu and X. Zhuang (2019). "Atipamezole is a promising non-discriminative inhibitor against pan-CYP450 including diclofenac 4′-hydroxylation: A comparison with ABT for drug ADME optimization and mechanism study." European Journal of Pharmaceutical Sciences **130**: 156-165.	Title
658	Liccardi, G., M. Cazzola, G. W. Canonica, M. D'Amato, G. D'Amato and G. Passalacqua (2003). "General strategy for the management of bronchial asthma in pregnancy." Respiratory Medicine **97**(7): 778-789.	Title
659	Lichter, J. B. and J. H. Kurth (1997). "The impact of pharmacogenetics on the future of healthcare." Current Opinion in Biotechnology **8**(6): 692-695.	Title
660	Lin, J. H. (2003). "Drug–drug interaction mediated by inhibition and induction of P-glycoprotein." Advanced Drug Delivery Reviews **55**(1): 53-81.	Abstract
661	Liu, A., W. Lin, R. Ming, W. Guan, X. Wang, N. Hu and Y. Ren (2022). "Stability of 28 typical prescription drugs in sewer systems and interaction with the biofilm bacterial community." Journal of Hazardous Materials **436**: 129142.	Title
662	Liu, T., B. S. Brown, Y. Wu, C. Antzelevitch, P. R. Kowey and G.-X. Yan (2006). "Blinded validation of the isolated arterially perfused rabbit ventricular wedge in preclinical assessment of drug-induced proarrhythmias." Heart Rhythm **3**(8): 948-956.	Animal
663	Liu, W., M. Nakano, T. Nakanishi, M. Nakajima and I. Tamai (2020). "Post-transcriptional regulation of OATP2B1 transporter by a microRNA, miR-24." Drug Metabolism and Pharmacokinetics **35**(6): 515-521.	Title
664	Lledó-García, R., A. Nácher, L. Prats-García, V. G. Casabó and M. Merino-Sanjuán (2007). "Bioavailability and Pharmacokinetic Model for Ritonavir in the Rat." Journal of Pharmaceutical Sciences **96**(3): 633-643.	Animal
665	Longhurst, H. J., M. Gonçalo, K. Godse and L. F. Ensina (2021). "Managing Chronic Urticaria and Recurrent Angioedema Differently with Advancing Age." The Journal of Allergy and Clinical Immunology: In Practice **9**(6): 2186-2194.	Title
666	Longo, N., D. Dimmock, H. Levy, K. Viau, H. Bausell, D. A. Bilder, B. Burton, C. Gross, H. Northrup, F. Rohr, S. Sacharow, A. Sanchez-Valle, M. Stuy, J. Thomas, J. Vockley, R. Zori and C. O. Harding (2019). "Evidence- and consensus-based recommendations for the use of pegvaliase in adults with phenylketonuria." Genetics in Medicine **21**(8): 1851-1867.	Title
667	Lu, C., K. Fu, K. Cao, J. Wei, J. Zhou, D. Zhao, N. Li, Y. Lu, X. Chen and Y. Zhang (2020). "Permeability and transport mechanism of trihexyphenidyl hydrochloride in Caco-2 cell monolayer model with a validated UPLC-MS/MS method." Journal of Pharmaceutical and Biomedical Analysis **178**: 112924.	Title

668	Luskin, A. T. and M. A. Lipkowitz (2000). "THE DIAGNOSIS AND MANAGEMENT OF ASTHMA DURING PREGNANCY." Radiologic Clinics of North America **20**(4): 745-761.	Title
669	Ma, Y., M. Zhang, J. Yang, L. Zhu, J. Dai and X. Wu (2023). "Characterization of the renal tubular transport of creatinine by activity-based protein profiling and transport kinetics." European Journal of Pharmaceutical Sciences **180**: 106342.	Title
670	Maeda, K., Y. Tian, T. Fujita, Y. Ikeda, Y. Kumagai, T. Kondo, K. Tanabe, H. Nakayama, S. Horita, H. Kusuhara and Y. Sugiyama (2014). "Inhibitory effects of p-aminohippurate and probenecid on the renal clearance of adefovir and benzylpenicillin as probe drugs for organic anion transporter (OAT) 1 and OAT3 in humans." European Journal of Pharmaceutical Sciences **59**: 94-103.	Title
671	Mandery, K., B. Balk, K. Bujok, I. Schmidt, M. F. Fromm and H. Glaeser (2012). "Inhibition of hepatic uptake transporters by flavonoids." European Journal of Pharmaceutical Sciences **46**(1): 79-85.	Title
672	Mandery, K., K. Bujok, I. Schmidt, M. Keiser, W. Siegmund, B. Balk, J. König, M. F. Fromm and H. Glaeser (2010). "Influence of the flavonoids apigenin, kaempferol, and quercetin on the function of organic anion transporting polypeptides 1A2 and 2B1." Biochemical Pharmacology **80**(11): 1746-1753.	Title
673	Manikkath, J., H. S. Parekh and S. Mutalik (2020). "Surface-engineered nanoliposomes with lipidated and non-lipidated peptide-dendrimeric scaffold for efficient transdermal delivery of a therapeutic agent: Development, characterization, toxicological and preclinical performance analyses." European Journal of Pharmaceutics and Biopharmaceutics **156**: 97-113.	Title
674	Mantha, K. B. (2023). "An Opinion on H1-Antihistamines as a Potential Avenue for Endometriosis Management." AJOG Global Reports: 100274.	Title
675	Matsui, K., S. Takeuchi, Y. Haruna, M. Yamane, T. Shimizu, Y. Hatsuma, N. Shimono, M. Sunada, M. Hayakawa, T. Nishida, S. Ito, M. Ide, M. Seino, M. Sugihara, Y. Minagawa and H. Tachiki (2020). "Transverse comparison of mannitol content in marketed drug products: Implication for no-effect dose of sugar alcohols on oral drug absorption." Journal of Drug Delivery Science and Technology **57**: 101728.	Title
676	May, B. C. and K. H. Gallivan (2022). "Levocetirizine and montelukast in the COVID-19 treatment paradigm." International Immunopharmacology **103**: 108412.	Title
677	Meeves, S. G. and S. Appajosyula (2003). "Efficacy and safety profile of fexofenadine HCL: A unique therapeutic option in H1-receptor antagonist treatment." Journal of Allergy and Clinical Immunology **112**(4, Supplement): S69-S77.	Full text
678	Milojkovic, M., S. Stojnev, I. Jovanovic, S. Ljubisavljevic, V. Stefanovic and R. Sunder-Plassman (2011). "Frequency of the C1236T, G2677T/A and C3435T MDR1 gene polymorphisms in the Serbian population." Pharmacological Reports **63**(3): 808-814.	Title

679	Mimura, N., Y. Nagata, T. Kuwabara, N. Kubo and E. Fuse (2008). "P-glycoprotein Limits the Brain Penetration of Olopatadine Hydrochloride, H1-Receptor Antagonist." Drug Metabolism and Pharmacokinetics **23**(2): 106-114.	Title
680	Miyazaki, N., S. Misaka, H. Ogata, T. Fukushima and J. Kimura (2013). "Effects of Itraconazole, Dexamethasone and Naringin on the Pharmacokinetics of Nadolol in Rats." Drug Metabolism and Pharmacokinetics **28**(4): 356-361.	Animal
681	Morganroth, J. (2004). "A definitive or thorough phase 1 QT ECG trial as a requirement for drug safety assessment." Journal of Electrocardiology **37**(1): 25-29.	Title
682	Morita, M. R., D. Berton, R. Boldin, F. A. P. Barros, E. C. Meurer, A. R. Amarante, D. R. Campos, S. A. Calafatti, R. Pereira, E. Abib and J. Pedrazolli (2008). "Determination of levocetirizine in human plasma by liquid chromatography–electrospray tandem mass spectrometry: Application to a bioequivalence study." Journal of Chromatography B **862**(1): 132-139.	Title
683	Morita, T., T. Akiyoshi, R. Sato, K. Katayama, K. Yajima, H. Kataoka, A. Imaoka, Y. Sugimoto and H. Ohtani (2020). "pH-dependent transport kinetics of the human organic anion-transporting polypeptide 1A2." Drug Metabolism and Pharmacokinetics **35**(2): 220-227.	Title
684	Morris, M. E. and S. Zhang (2006). "Flavonoid–drug interactions: Effects of flavonoids on ABC transporters." Life Sciences **78**(18): 2116-2130.	Title
685	Mouez, M. A., M. Nasr, M. Abdel-Mottaleb, A. S. Geneidi and S. Mansour (2016). "Composite chitosan-transfersomal vesicles for improved transnasal permeation and bioavailability of verapamil." International Journal of Biological Macromolecules **93**: 591-599.	Title
686	Mouly, S., C. Lloret-Linares, P.-O. Sellier, D. Sene and J. F. Bergmann (2017). "Is the clinical relevance of drug-food and drug-herb interactions limited to grapefruit juice and Saint-John's Wort?" Pharmacological Research **118**: 82-92.	Title
687	Musther, H., A. Olivares-Morales, O. J. D. Hatley, B. Liu and A. Rostami Hodjegan (2014). "Animal versus human oral drug bioavailability: Do they correlate?" European Journal of Pharmaceutical Sciences **57**: 280-291.	Title
688	Na, L., S. Mao, J. Wang and W. Sun (2010). "Comparison of different absorption enhancers on the intranasal absorption of isosorbide dinitrate in rats." International Journal of Pharmaceutics **397**(1): 59-66.	Animal
689	Nabekura, T., T. Kawasaki, M. Jimura, K. Mizuno and Y. Uwai (2020). "Microtubule-targeting anticancer drug eribulin induces drug efflux transporter P-glycoprotein." Biochemistry and Biophysics Reports **21**: 100727.	Title

690	Naicker, P., S. Anoopkumar-Dukie, G. D. Grant and J. J. Kavanagh (2013). "The effects of antihistamines with varying anticholinergic properties on voluntary and involuntary movement." Clinical Neurophysiology **124**(9): 1840-1845.	Title
691	Neerati, P. and S. K. Bedada (2015). "Effect of diosmin on the intestinal absorption and pharmacokinetics of fexofenadine in rats." Pharmacological Reports **67**(2): 339-344.	Animal
692	Nguyen, M. A., P. Staubach, I. Tamai and P. Langguth (2015). "High-dose short-term administration of naringin did not alter talinolol pharmacokinetics in humans." European Journal of Pharmaceutical Sciences **68**: 36-42.	Title
693	Nguyen, M. A., P. Staubach, S. Wolffram and P. Langguth (2014). "Effect of single-dose and short-term administration of quercetin on the pharmacokinetics of talinolol in humans – Implications for the evaluation of transporter-mediated flavonoid–drug interactions." European Journal of Pharmaceutical Sciences **61**: 54-60.	Title
694	Nicolas, J.-M., R. Whomsley, P. Collart and J. Roba (1999). "In vitro inhibition of human liver drug metabolizing enzymes by second generation antihistamines." Chemico-Biological Interactions **123**(1): 63-79.	Abstract
695	Nieminen, T. H., N. M. Hagelberg, T. I. Saari, M. Neuvonen, K. Laine, P. J. Neuvonen and K. T. Olkkola (2010). "St John's wort greatly reduces the concentrations of oral oxycodone." European Journal of Pain **14**(8): 854-859.	Title
696	Niggemann, B. and C. Grüber (2006). "Does unconventional medicine work through conventional modes of action?" Journal of Allergy and Clinical Immunology **118**(3): 569-573.	Title
697	Nomura, H., H. Mizuta, H. Norimoto, F. Masuda, Y. Miura, A. Kubo, H. Kojima, A. Ashizuka, N. Matsukawa, Z. Baraki, N. Hitora-Imamura, D. Nakayama, T. Ishikawa, M. Okada, K. Orita, R. Saito, N. Yamauchi, Y. Sano, H. Kusuhara, M. Minami, H. Takahashi and Y. Ikegaya (2019). "Central Histamine Boosts Perirhinal Cortex Activity and Restores Forgotten Object Memories." Biological Psychiatry **86**(3): 230-239.	Title
698	Nyberg, L., W. Månsson, B. Abrahamsson, J. Seidegård and O. Borgå (2007). "A convenient method for local drug administration at predefined sites in the entire gastrointestinal tract: Experiences from 13 phase I studies." European Journal of Pharmaceutical Sciences **30**(5): 432-440.	Title
699	Ohmura, T., M. Chachin, S. Tarui, A. Nagakura, T. Igarashi, H. Ikeda, K. Ikegami, H. Kitagawa and S. Uchida (1999). "Effects of terfenadine, astemizole and epinastine on electrocardiogram in conscious cynomolgus monkeys." European Journal of Pharmacology **378**(2): 169-175.	Animal

700	Ohura, K., T. Soejima, R. Nogata, Y. Adachi, S.-I. Ninomiya and T. Imai (2012). "Effect of Intestinal First-Pass Hydrolysis on the Oral Bioavailability of an Ester Prodrug of Fexofenadine." Journal of Pharmaceutical Sciences **101**(9): 3264-3274.	Animal
701	Okubo, K., M. Gotoh, S. Fujieda, M. Okano, H. Yoshida, H. Morikawa, K. Masuyama, Y. Okamoto and M. Kobayashi (2008). "A Randomized Double-Blind Comparative Study of Sublingual Immunotherapy for Cedar Pollinosis." Allergology International **57**(3): 265-275.	Title
702	Olsén, L., U. Bondesson, H. Broström, H. Tjälve and C. Ingvast-Larsson (2008). "Cetirizine in horses: Pharmacokinetics and pharmacodynamics following repeated oral administration." The Veterinary Journal **177**(2): 242-249.	Animal
703	Oswald, S., E. Scheuch, I. Cascorbi and W. Siegmund (2006). "A LC–MS/MS method to quantify the novel cholesterol lowering drug ezetimibe in human serum, urine and feces in healthy subjects genotyped for SLCO1B1." Journal of Chromatography B **830**(1): 143-150.	Abstract
704	Ousler, G. W., K. A. Wilcox, G. Gupta, M. B. Abelson and K. Fink (2004). "An evaluation of the ocular drying effects of 2 systemic antihistamines: loratadine and cetirizine hydrochloride." Annals of Allergy, Asthma & Immunology **93**(5): 460-464.	Abstract
705	Öztaş, E., A. Parejo Garcia-Saavedra, F. Yanar, B. Özçinar, N. Aksakal, S. Purisa and G. Özhan (2018). "P-glycoprotein polymorphism and levothyroxine bioavailability in hypothyroid patients." Saudi Pharmaceutical Journal **26**(2): 274-278.	Title
706	Padowski, J. M. and G. M. Pollack (2010). "Examination of the Ability of the Nasal Administration Route to Confer a Brain Exposure Advantage for Three Chemical Inhibitors of P-Glycoprotein." Journal of Pharmaceutical Sciences **99**(7): 3226-3233.	Title
707	Paine, M. F., W. W. Widmer, H. L. Hart, S. N. Pusek, K. L. Beavers, A. B. Criss, S. S. Brown, B. F. Thomas and P. B. Watkins (2006). "A furanocoumarin-free grapefruit juice establishes furanocoumarins as the mediators of the grapefruit juice–felodipine interaction2." The American Journal of Clinical Nutrition **83**(5): 1097-1105.	Title
708	Paine, M. F., W. W. Widmer, S. N. Pusek, K. L. Beavers, A. B. Criss, J. Snyder and P. B. Watkins (2008). "Further characterization of a furanocoumarin-free grapefruit juice on drug disposition: studies with cyclosporine1." The American Journal of Clinical Nutrition **87**(4): 863-871.	Title
709	Paixão, P., L. F. Gouveia and J. A. G. Morais (2010). "Prediction of the in vitro permeability determined in Caco-2 cells by using artificial neural networks." European Journal of Pharmaceutical Sciences **41**(1): 107-117.	Title
710	Pal, D. and A. K. Mitra (2006). "MDR- and CYP3A4-mediated drug–herbal interactions." Life Sciences **78**(18): 2131-2145.	Title

711	Pala Kara, Z., D. Ozturk Civelek, N. Ozturk and A. Okyar (2021). "The effects of P-glycoprotein inhibitor zosuquidar on the sex and time-dependent pharmacokinetics of parenterally administered talinolol in mice." European Journal of Pharmaceutical Sciences **156**: 105589.	Animal
712	Patel, R. D., S. P. Kumar, C. N. Patel, S. S. Shankar, H. A. Pandya and H. A. Solanki (2017). "Parallel screening of drug-like natural compounds using Caco-2 cell permeability QSAR model with applicability domain, lipophilic ligand efficiency index and shape property: A case study of HIV-1 reverse transcriptase inhibitors." Journal of Molecular Structure **1146**: 80-95.	Title
713	Pathak, S. M., A. R. Kumar, P. Musmade and N. Udupa (2008). "A simple and rapid high performance liquid chromatographic method with fluorescence detection for the estimation of fexofenadine in rat plasma—Application to preclinical pharmacokinetics." Talanta **76**(2): 338-346.	Animal
714	Pawar, V. K., Y. Singh, K. Sharma, A. Shrivastav, A. Sharma, A. Singh, J. G. Meher, P. Singh, K. Raval, A. Kumar, H. K. Bora, D. Datta, J. Lal and M. K. Chourasia (2019). "Improved chemotherapy against breast cancer through immunotherapeutic activity of fucoidan decorated electrostatically assembled nanoparticles bearing doxorubicin." International Journal of Biological Macromolecules **122**: 1100-1114.	Title
715	Peng, S. X., D. M. Ritchie, M. Cousineau, E. Danser, R. DeWire and J. Floden (2006). "Altered Oral Bioavailability and Pharmacokinetics of P-Glycoprotein Substrates by Coadministration of Biochanin A." Journal of Pharmaceutical Sciences **95**(9): 1984-1993.	Animal
716	Peng, Y., P. Yadava, A. T. Heikkinen, N. Parrott and A. Railkar (2014). "Applications of a 7-day Caco-2 cell model in drug discovery and development." European Journal of Pharmaceutical Sciences **56**: 120-130.	Title
717	Pereira da Silva, V., L. de Carvalho Brito, A. Mesquita Marques, F. da Cunha Camillo and M. Raquel Figueiredo (2023). "Bioactive limonoids from Carapa guianensis seeds oil and the sustainable use of its by-products." Current Research in Toxicology **4**: 100104.	Title
718	Piao, H.-M., P. Balakrishnan, H.-J. Cho, H. Kim, Y.-S. Kim, S.-J. Chung, C.-K. Shim and D.-D. Kim (2010). "Preparation and evaluation of fexofenadine microemulsions for intranasal delivery." International Journal of Pharmaceutics **395**(1): 309-316.	Animal
719	Polli, J. W., T. M. Baughman, J. E. Humphreys, K. H. Jordan, A. L. Mote, J. A. Salisbury, T. K. Tippin and C. J. Serabjit-Singh (2003). "P-glycoprotein Influences the Brain Concentrations of Cetirizine (Zyrtec®), a Second-Generation Non-Sedating Antihistamine." Journal of Pharmaceutical Sciences **92**(10): 2082-2089.	Title
720	Pondugula, S. R., P. C. Flannery, K. L. Abbott, E. S. Coleman, S. Mani, T. Samuel and W. Xie (2015). "Diindolylmethane, a naturally occurring compound, induces CYP3A4 and MDR1 gene expression by activating human PXR." Toxicology Letters **232**(3): 580-589.	Title
721	Popov, T. A. (2011). "Challenges in the Management of Chronic Urticaria." World Allergy Organization Journal **4**: S28-S31.	Title

722	Popovic, M., R. Zaja and T. Smital (2010). "Organic anion transporting polypeptides (OATP) in zebrafish (Danio rerio): Phylogenetic analysis and tissue distribution." Comparative Biochemistry and Physiology Part A: Molecular & Integrative Physiology **155**(3): 327-335.	Title
723	Poulin, P. (2013). "Prediction of Total Hepatic Clearance by Combining Metabolism, Transport, and Permeability Data in the In Vitro–In Vivo Extrapolation Methods: Emphasis on an Apparent Fraction Unbound in Liver for Drugs." Journal of Pharmaceutical Sciences **102**(7): 2085-2095.	Title
724	Poulin, P., S. H. Collet, N. Atrux-Tallau, J.-M. Linget, L. Hennequin and C. E. Wilson (2019). "Application of the Tissue Composition–Based Model to Minipig for Predicting the Volume of Distribution at Steady State and Dermis-to-Plasma Partition Coefficients of Drugs Used in the Physiologically Based Pharmacokinetics Model in Dermatology." Journal of Pharmaceutical Sciences **108**(1): 603-619.	Animal
725	Poulin, P. and S. Haddad (2021). "A New Guidance for the Prediction of Hepatic Clearance in the Early Drug Discovery and Development from the in Vitro-to-in Vivo Extrapolation Method and an Approach for Exploring Whether an Albumin-Mediated Hepatic Uptake Phenomenon Could be Present Under in Vivo Conditions." Journal of Pharmaceutical Sciences **110**(7): 2841-2858.	Title
726	Purohit, A., A. S. N'Gom, B. Deslandes, G. Pauli and N. Frossard (2004). "Similar rapid onset of action and magnitude of effect of fexofenadine and cetirizine as assessed by inhibition of histamine-induced wheal-and-flare reaction." Annals of Allergy, Asthma & Immunology **93**(6): 562-567.	Abstract
727	Qiang, F., B.-J. Lee, W. Lee and H.-K. Han (2009). "Pharmacokinetic drug interaction between fexofenadine and fluvastatin mediated by organic anion-transporting polypeptides in rats." European Journal of Pharmaceutical Sciences **37**(3): 413-417.	Animal
728	Raikar, P., B. Gurupadayya, S. P. Mandal, R. Narhari, S. Subramanyam, G. Srinivasu, S. Rajan, M. Saikumar and S. Koganti (2020). "Bioanalytical chiral chromatographic technique and docking studies for enantioselective separation of meclizine hydrochloride: Application to pharmacokinetic study in rabbits." Chirality **32**(8): 1091-1106.	Animal
729	Ramage, S., D. Camacho-Muñoz and B. Petrie (2019). "Enantioselective LC-MS/MS for anthropogenic markers of septic tank discharge." Chemosphere **219**: 191-201.	Title
730	Rankin, A. C. (1998). "Cardiac adverse effects of H1-antihistamines." Revue Française d'Allergologie et d'Immunologie Clinique **38**(10): 931-934.	Abstract
731	Reid, M. J., J. A. Baz-Lomba, Y. Ryu and K. V. Thomas (2014). "Using biomarkers in wastewater to monitor community drug use: A conceptual approach for dealing with new psychoactive substances." Science of The Total Environment **487**: 651-658.	Title
732	Ren, T., M. Yang, M. Xiao, J. Zhu, W. Xie and Z. Zuo (2019). "Time-dependent inhibition of carbamazepine metabolism by piperine in anti-epileptic treatment." Life Sciences **218**: 314-323.	Title

733	Rodrigues, A. C., R. Curi, L. R. G. Britto, I. M. M. Rebbechi, M. H. Hirata, M. C. Bertolami, M. M. S. Bernik, E. L. Dorea and R. D. C. Hirata (2006). "Down-regulation of ABCB1 transporter by atorvastatin in a human hepatoma cell line and in human peripheral blood mononuclear cells." Biochimica et Biophysica Acta (BBA) - General Subjects **1760**(12): 1866-1873.	Title
734	Roma, P. G., J. S. Schneiderman, J. M. Schorn, S. E. Whiting, L. B. Landon and T. J. Williams (2021). "Assessment of Spaceflight Medical Conditions' and Treatments' Potential Impacts on Behavioral Health and Performance." Life Sciences in Space Research **30**: 72-81.	Title
735	Rowland, M., C. R. Noe, D. A. Smith, G. T. Tucker, D. J. A. Crommelin, C. C. Peck, M. L. Rocci Jr, L. Besançon and V. P. Shah (2012). "Impact of the Pharmaceutical Sciences on Health Care: A Reflection over the Past 50 Years." Journal of Pharmaceutical Sciences **101**(11): 4075-4099.	Title
736	Russo, G., G. Ermondi, G. Caron, D. Verzele and F. Lynen (2021). "Into the first biomimetic sphingomyelin stationary phase: Suitability in drugs' biopharmaceutic profiling and block relevance analysis of selectivity." European Journal of Pharmaceutical Sciences **156**: 105585.	Title
737	Russo, G., L. Grumetto, M. Baert and F. Lynen (2021). "Comprehensive two-dimensional liquid chromatography as a biomimetic screening platform for pharmacokinetic profiling of compound libraries in early drug development." Analytica Chimica Acta **1142**: 157-168.	Title
738	Ryu, S., D. Tess, G. Chang, C. Keefer, W. Burchett, G. S. Steeno, J. J. Novak, R. Patel, K. Atkinson, K. Riccardi and L. Di (2020). "Evaluation of Fraction Unbound Across 7 Tissues of 5 Species." Journal of Pharmaceutical Sciences **109**(2): 1178-1190.	Title
739	Saeed Arayne, M., N. Sultana, H. Shehnaz, S.-u.-R. Mandukhail, A. H. Gilani and A. Haider (2017). "Antihistaminic and other biological activities of 2-methylpropanamide and benzamide derivatives of carboxyterfenadine." Arabian Journal of Chemistry **10**(1): 114-120.	Abstract
740	Sahi, J., M. W. Sinz, S. Campbell, R. Mireles, X. Zheng, K. A. Rose, S. Raeissi, M. F. Hashim, Y. Ye, S. M. de Morais, C. Black, M. Tugnait and L. H. Keller (2006). "Metabolism and transporter-mediated drug–drug interactions of the endothelin-A receptor antagonist CI-1034." Chemico-Biological Interactions **159**(2): 156-168.	Abstract
741	Sakai, Y., T. Iwao, T. Susukida, T. Nukaga, A. Takemura, S. Sekine, K. Ito and T. Matsunaga (2019). "In vitro bile acid-dependent hepatocyte toxicity assay system using human induced pluripotent stem cell-derived hepatocytes: Current status and disadvantages to overcome." Drug Metabolism and Pharmacokinetics **34**(4): 264-271.	Title
742	Sakata, T., N. Anzai, H. J. Shin, R. Noshiro, T. Hirata, H. Yokoyama, Y. Kanai and H. Endou (2004). "Novel single nucleotide polymorphisms of organic cation transporter 1 (SLC22A1) affecting transport functions." Biochemical and Biophysical Research Communications **313**(3): 789-793.	Title
743	Sakolish, C., C. E. Reese, Y.-S. Luo, A. Valdiviezo, M. E. Schurdak, A. Gough, D. L. Taylor, W. A. Chiu, L. A. Vernetti and I. Rusyn (2021). "Analysis of reproducibility and robustness of a human microfluidic four-cell liver acinus microphysiology system (LAMPS)." Toxicology **448**: 152651.	Abstract

744	Salama, N. N., Z. Yang, T. o. t. Bui and R. J. Y. Ho (2006). "MDR1 haplotypes significantly minimize intracellular uptake and transcellular P-gp substrate transport in recombinant LLC-PK1 cells." Journal of Pharmaceutical Sciences **95**(10): 2293-2308.	Abstract
745	Saleh, H. M., M. M. El-Henawee, G. H. Ragab and S. S. A. El-Hay (2007). "Utility of NBD-Cl for the spectrophotometric determination of some skeletal muscle relaxant and antihistaminic drugs." Spectrochimica Acta Part A: Molecular and Biomolecular Spectroscopy **67**(5): 1284-1289.	Abstract
746	Sampey, C. S. and S. L. Follin (2001). "Second-Generation Antihistamines: The OTC Debate." Journal of the American Pharmaceutical Association (1996) **41**(3): 454-457.	Title
747	Sapir, E., R. Pfeffer, M. Wygoda, O. Purim, A. Levy, B. Corn, Y. Amitay, P. Ohana and A. Gabizon (2023). "Pegylated Liposomal Mitomycin C Lipidic Prodrug in Combination With External Beam Radiation Therapy in Patients With Advanced Cancer: A Phase 1B Study." International Journal of Radiation Oncology*Biology*Physics **117**(1): 64-73.	Title
748	Sato, R., T. Akiyoshi, T. Morita, K. Katayama, K. Yajima, H. Kataoka, A. Imaoka and H. Ohtani (2021). "Dual kinetics of OATP2B1: Inhibitory potency and pH-dependence of OATP2B1 inhibitors." Drug Metabolism and Pharmacokinetics **41**: 100416.	Abstract
749	Savoji, H., M. H. Mohammadi, N. Rafatian, M. K. Toroghi, E. Y. Wang, Y. Zhao, A. Korolj, S. Ahadian and M. Radisic (2019). "Cardiovascular disease models: A game changing paradigm in drug discovery and screening." Biomaterials **198**: 3-26.	Title
750	Sawangrat, K., M. Morishita, K. Kusamori, H. Katsumi, T. Sakane and A. Yamamoto (2018). "Effects of Various Pharmaceutical Excipients on the Intestinal Transport and Absorption of Sulfasalazine, a Typical Substrate of Breast Cancer Resistance Protein Transporter." Journal of Pharmaceutical Sciences **107**(11): 2946-2956.	Title
751	Sawangrat, K., S. Yamashita, A. Tanaka, M. Morishita, K. Kusamori, H. Katsumi, T. Sakane and A. Yamamoto (2019). "Modulation of Intestinal Transport and Absorption of Topotecan, a BCRP Substrate, by Various Pharmaceutical Excipients and Their Inhibitory Mechanisms of BCRP Transporter." Journal of Pharmaceutical Sciences **108**(3): 1315-1325.	Abstract
752	Schafer, A., H. Cheng, R. Xiong, V. Soloveva, C. Retterer, F. Mo, S. Bavari, G. Thatcher and L. Rong (2018). "Repurposing potential of 1st generation H1-specific antihistamines as anti-filovirus therapeutics." Antiviral Research **157**: 47-56.	Abstract
753	Scheinfeld, N. S., W. D. Tutrone, O. Torres and J. M. Weinberg (2003). "Macrolides in dermatology." Clinics in Dermatology **21**(1): 40-49.	Title
754	Schmidt, A., A. Schmidt and U. R. Markert (2021). "The road (not) taken – Placental transfer and interspecies differences." Placenta **115**: 70-77.	Title
755	Schmutzler, W. (1999). "New aspects in the pharmacotherapy of allergic diseases." Revue Française d'Allergologie et d'Immunologie Clinique **39**: 18-21.	Title

756	Seeland, S., A. Treiber, M. Hafner and J. Huwyler (2011). "On-line identification of P-glycoprotein substrates by monitoring of extracellular acidification and respiration rates in living cells." Biochimica et Biophysica Acta (BBA) - Biomembranes **1808**(7): 1827-1831.	Abstract
757	Shafi, H., R. Rashid, S.-u. Rather, D. V. Siva Reddy, L. Azmi, A. Abdal-hay, S. H. Alrokayan, H. A. Khan, N. Ahmad Khan and F. A. Sheikh (2023). "Super disintegrating oromucosal nanofiber patch of zolmitriptan for rapid delivery and efficient brain targeting." Chemical Engineering Journal **463**: 142481.	Title
758	Shapiro, L. E. and N. H. Shear (2001). "Drug interactions/P450." Current Problems in Dermatology **13**(3): 141-152.	Title
759	Shapiro, L. E. and N. H. Shear (2002). "Drug interactions: Proteins, pumps, and P-450s." Journal of the American Academy of Dermatology **47**(4): 467-488.	Title
760	Shirasaka, Y., M. Kawasaki, T. Sakane, H. Omatsu, Y. Moriya, T. Nakamura, T. Sakaeda, K. Okumura, P. Langguth and S. Yamashita (2006). "Induction of Human P-Glycoprotein in Caco-2 cells: Development of a Highly Sensitive Assay System for P-Glycoprotein-Mediated Drug Transport." Drug Metabolism and Pharmacokinetics **21**(5): 414-423.	Abstract
761	Shrinet, K., R. K. Singh, R. Saxena, A. K. Chaurasia and A. Kumar (2023). "Ab initio modeling and ligand docking of quercetin and the MC-LR transporter protein Oatp1b2/OATP1B3." Pharmaceutical Science Advances **1**(2): 100011.	Title
762	Simons, F. E. (1999). "H1-receptor antagonists: safety issues." Annals of Allergy, Asthma & Immunology **83**(5): 481-488.	Title
763	Simons, F. E. R. (2003). "H1-antihistamines: More relevant than ever in the treatment of allergic disorders." Journal of Allergy and Clinical Immunology **112**(4, Supplement): S42-S52.	Title
764	Simons, F. E. R., J. N. Bergman, W. T. A. Watson and K. J. Simons (1996). "The clinical pharmacology of fexofenadine in children." Journal of Allergy and Clinical Immunology **98**(6, Part 1): 1062-1064.	Abstract
765	Simons, F. E. R., L. Johnston, X. Gu and K. J. Simons (2001). "Suppression of the early and late cutaneous allergic responses using fexofenadine and montelukast." Annals of Allergy, Asthma & Immunology **86**(1): 44-50.	Abstract
766	Simons, F. E. R., J. R. Roberts, X. Gu, S. Kapur and K. J. Simons (1999). "The clinical pharmacology of brompheniramine in children." Journal of Allergy and Clinical Immunology **103**(2): 223-226.	Title

767	Simons, F. E. R. and K. J. Simons (2005). "Levocetirizine: Pharmacokinetics and pharmacodynamics in children age 6 to 11 years." Journal of Allergy and Clinical Immunology **116**(2): 355-361.	Title
768	Simons, R. and F. Estelle (1998). "Clinical pharmacology of new H1-receptor antagonists, and why it matters." Revue Française d'Allergologie et d'Immunologie Clinique **38**(10): 921-924.	Title
769	Simšič, T., B. Nolimal, J. Minova, A. Baumgartner and O. Planinšek (2021). "A straw for paediatrics: How to administer highly dosed, bitter tasting paracetamol granules." International Journal of Pharmaceutics **602**: 120615.	Title
770	Singer, M. I., L. E. Shapiro and N. H. Shear (1997). "Cytochrome P-450 3A: Interactions with dermatologic therapies." Journal of the American Academy of Dermatology **37**(5, Part 1): 765-771.	Title
771	Sjöberg, Å., M. Lutz, C. Tannergren, C. Wingolf, A. Borde and A.-L. Ungell (2013). "Comprehensive study on regional human intestinal permeability and prediction of fraction absorbed of drugs using the Ussing chamber technique." European Journal of Pharmaceutical Sciences **48**(1): 166-180.	Abstract
772	Sjögren, E., B. Abrahamsson, P. Augustijns, D. Becker, M. B. Bolger, M. Brewster, J. Brouwers, T. Flanagan, M. Harwood, C. Heinen, R. Holm, H.-P. Juretschke, M. Kubbinga, A. Lindahl, V. Lukacova, U. Münster, S. Neuhoff, M. A. Nguyen, A. v. Peer, C. Reppas, A. R. Hodjegan, C. Tannergren, W. Weitschies, C. Wilson, P. Zane, H. Lennernäs and P. Langguth (2014). "In vivo methods for drug absorption – Comparative physiologies, model selection, correlations with in vitro methods (IVIVC), and applications for formulation/API/excipient characterization including food effects." European Journal of Pharmaceutical Sciences **57**: 99-151.	Title
773	Sjögren, E., M. Hedeland, U. Bondesson and H. Lennernäs (2014). "Effects of verapamil on the pharmacokinetics and hepatobiliary disposition of fexofenadine in pigs." European Journal of Pharmaceutical Sciences **57**: 214-223.	Animal
774	Sjögren, E., J. Westergren, I. Grant, G. Hanisch, L. Lindfors, H. Lennernäs, B. Abrahamsson and C. Tannergren (2013). "In silico predictions of gastrointestinal drug absorption in pharmaceutical product development: Application of the mechanistic absorption model GI-Sim." European Journal of Pharmaceutical Sciences **49**(4): 679-698.	Title
775	Sourkes, B., L. Frankel, M. Brown, N. Contro, W. Benitz, C. Case, J. Good, L. Jones, J. Komejan, J. Modderman-Marshall, W. Reichard, S. Sentivany-Collins and C. Sunde (2005). "Food, Toys, and Love: Pediatric Palliative Care." Current Problems in Pediatric and Adolescent Health Care **35**(9): 350-386.	Title
776	Sparreboom, A., R. Danesi, Y. Ando, J. Chan and W. D. Figg (2003). "Pharmacogenomics of ABC transporters and its role in cancer chemotherapy." Drug Resistance Updates **6**(2): 71-84.	Title

777	Stokes, J. R., F. A. Romero, R. J. Allan, P. G. Phillips, F. Hackman, J. Misfeldt and T. B. Casale (2012). "The effects of an H3 receptor antagonist (PF-03654746) with fexofenadine on reducing allergic rhinitis symptoms." Journal of Allergy and Clinical Immunology **129**(2): 409-412.e402.	Abstract
778	Storch, C. H., D. Theile, H. Lindenmaier, W. E. Haefeli and J. Weiss (2007). "Comparison of the inhibitory activity of anti-HIV drugs on P-glycoprotein." Biochemical Pharmacology **73**(10): 1573-1581.	Title
779	Strauch, K., U. Lutz, N. Bittner and W. K. Lutz (2009). "Dose–response relationship for the pharmacokinetic interaction of grapefruit juice with dextromethorphan investigated by human urinary metabolite profiles." Food and Chemical Toxicology **47**(8): 1928-1935.	Title
780	Strelevitz, T. J., R. S. Foti and M. B. Fisher (2006). "In Vivo use of the P450 inactivator 1-aminobenzotriazole in the rat: Varied dosing route to elucidate gut and liver contributions to first-pass and systemic clearance." Journal of Pharmaceutical Sciences **95**(6): 1334-1341.	Animal
781	Suchanova, B., L. Sispera and V. Wsol (2006). "Liquid chromatography–tandem mass spectrometry in chiral study of amlodipine biotransformation in rat hepatocytes." Analytica Chimica Acta **573-574**: 273-283.	Animal
782	Sugimoto, H., S.-I. Matsumoto, M. Tachibana, S.-I. Niwa, H. Hirabayashi, N. Amano and T. Moriwaki (2011). "Establishment of In Vitro P-Glycoprotein Inhibition Assay and Its Exclusion Criteria to Assess the Risk Of Drug–Drug Interaction at the Drug Discovery Stage." Journal of Pharmaceutical Sciences **100**(9): 4013-4023.	Title
783	Sugiura, T., Y. Kato and A. Tsuji (2006). "Role of SLC xenobiotic transporters and their regulatory mechanisms PDZ proteins in drug delivery and disposition." Journal of Controlled Release **116**(2): 238-246.	Title
784	Sugiura, T., T. Otake, T. Shimizu, T. Wakayama, D. L. Silver, R. Utsumi, T. Nishimura, S. Iseki, N. Nakamichi, Y. Kubo, A. Tsuji and Y. Kato (2010). "PDZK1 Regulates Organic Anion Transporting Polypeptide Oatpla in Mouse Small Intestine." Drug Metabolism and Pharmacokinetics **25**(6): 588-598.	Animal
785	Sun, P., C. Wang, Q. Liu, Q. Meng, A. Zhang, X. Huo, H. Sun and K. Liu (2014). "OATP and MRP2-mediated hepatic uptake and biliary excretion of eprosartan in rat and human." Pharmacological Reports **66**(2): 311-319.	Title
786	Sun, X., Y. Ding, B. Niu and Q. Chen (2021). "Evaluation of a composite nanomaterial consist of gold nanoparticles and graphene-carbon nitride as capillary electrochromatography stationary phase for enantioseparation." Microchemical Journal **169**: 106613.	Title
787	Suroowan, S., H. H. Abdallah and M. F. Mahomoodally (2021). "Herb-drug interactions and toxicity: Underscoring potential mechanisms and forecasting clinically relevant interactions induced by common phytoconstituents via data mining and computational approaches." Food and Chemical Toxicology **156**: 112432.	Title

788	Suzuki, K., T. Morokata, K. Morihira, I. Sato, S. Takizawa, M. Kaneko, K. Takahashi and Y. Shimizu (2007). "A dual antagonist for chemokine CCR3 receptor and histamine H1 receptor." European Journal of Pharmacology **563**(1): 224-232.	Title
789	Suzuki, K., Y. Shitara, K. Fukuda and T. Horie (2012). "Long-Lasting Inhibition of the Intestinal Absorption of Fexofenadine by Cyclosporin a in Rats." Journal of Pharmaceutical Sciences **101**(7): 2606-2615.	Animal
790	Suzuki, Y., Y. Sasamoto, C. Yoshijima, R. Tanaka, H. Ono, T. Ando, T. Shin, H. Mimata, H. Itoh and K. Ohno (2020). "Simultaneous quantification of coproporphyrin-I and 3-carboxy-4-methyl-5-propyl-2-furanpropanoic acid in human plasma using ultra-high performance liquid chromatography coupled to tandem mass spectrometry." Journal of Pharmaceutical and Biomedical Analysis **184**: 113202.	Title
791	Swales, J. G., G. Hamm, M. R. Clench and R. J. A. Goodwin (2019). "Mass spectrometry imaging and its application in pharmaceutical research and development: A concise review." International Journal of Mass Spectrometry **437**: 99-112.	Title
792	Sziráki, I., F. Erdő, E. Beéry, P. M. Molnár, C. Fazakas, I. Wilhelm, I. Makai, E. Kis, K. Herédi-Szabó, T. Abonyi, I. Krizbai, G. K. Tóth and P. Krajcsi (2011). "Quinidine as an ABCB1 Probe for Testing Drug Interactions at the Blood–Brain Barrier: An In Vitro In Vivo Correlation Study." SLAS Discovery **16**(8): 886-894.	Title
793	Takahashi, G., F. Asanuma, N. Suzuki, M. Hattori, S. Sakamoto, A. Kugimiya, Y. Tomita, G. Kuwajima, W. M. Abraham, M. Deguchi, A. Arimura and M. Shichijo (2015). "Effect of the potent and selective DP1 receptor antagonist, asapiprant (S-555739), in animal models of allergic rhinitis and allergic asthma." European Journal of Pharmacology **765**: 15-23.	Animal
794	Takayama, K., R. Negoro, T. Yamashita, K. Kawai, M. Ichikawa, T. Mori, N. Nakatsu, K. Harada, S. Ito, H. Yamada, Y. Yamaura, K. Hirata, S. Ishida and H. Mizuguchi (2019). "Generation of Human iPSC–Derived Intestinal Epithelial Cell Monolayers by CDX2 Transduction." Cellular and Molecular Gastroenterology and Hepatology **8**(3): 513-526.	Title
795	Tamura, T. and M. Komai (2008). "Effect of olopatadine hydrochloride, an anti-histamine drug, on rhinitis induced by intranasal instillation of toluene-2,4-diisocyanate in rats." International Immunopharmacology **8**(6): 916-921.	Animal
796	Tamura, T., H. Sato, I. Miki, K. Suzuki, K. Ohmori and A. Karasawa (2003). "Effects of orally administered olopatadine hydrochloride on the ocular allergic reaction in rats." Allergology International **52**(2): 77-83.	Animal
797	Tan, H. L., L. W. T. Tang, S. Y. Chin and E. C. Y. Chan (2021). "Investigation of the arcane inhibition of human organic anion transporter 3 by benzofuran antiarrhythmic agents." Drug Metabolism and Pharmacokinetics **38**: 100390.	Title
798	Tang, W., Y. Lu, K. H. Row, S. H. Baeck, Y. Zhang and G. Sun (2022). "Novel bovine serum album and β-cyclodextrin-based mixed chiral stationary phase for the enantioseparation in capillary electrochromatography." Microchemical Journal **181**: 107763.	Title

799	Tannergren, C., H. Jadhav, E. Eckernäs, J. Fagerberg, P. Augustijns and E. Sjögren (2023). "Physiologically based biopharmaceutics modeling of regional and colon absorption in humans." European Journal of Pharmaceutics and Biopharmaceutics **186**: 144-159.	Title
800	Tarantino, P., N. Appleton and K. Lansdell (2005). "Effect of trazodone on hERG channel current and QT-interval." European Journal of Pharmacology **510**(1): 75-85.	Title
801	Tareq, A. M., M. M. Hossain, M. Uddin, F. Islam, Z. Khan, M. M. Karim, C. Lyzu, D. Ağagündüz, A. S. M. A. Reza, T. B. Emran and R. Capasso (2023). "Chemical profiles and pharmacological attributes of Apis cerana indica beehives using combined experimental and computer-aided studies." Heliyon **9**(4): e15016.	Title
802	Teague, S. and K. Valko (2017). "How to identify and eliminate compounds with a risk of high clinical dose during the early phase of lead optimisation in drug discovery." European Journal of Pharmaceutical Sciences **110**: 37-50.	Title
803	Ten Eick, A. P., H. Nakamura and M. D. Reed (1998). "DRUG–DRUG INTERACTIONS IN PEDIATRIC PSYCHOPHARMACOLOGY." Pediatric Clinics of North America **45**(5): 1233-1264.	Title
804	Tomaru, A., M. Takeda-Morishita, H. Banba and K. Takayama (2013). "Analysis of the Pharmacokinetic Boosting Effects of Ritonavir on Oral Bioavailability of Drugs in Mice." Drug Metabolism and Pharmacokinetics **28**(2): 144-152.	Animal
805	Tozuka, Z., S. Aoyama, K. Nozawa, S. Akita, T. Oh-Hara, Y. Adachi and S.-I. Ninomiya (2011). "Comprehensive Quantitative and Qualitative Liquid Chromatography–Radioisotope–Mass Spectrometry Analysis for Safety Testing of Tolbutamide Metabolites Without Standard Samples." Journal of Pharmaceutical Sciences **100**(9): 4024-4036.	Title
806	Turner, J. V., B. D. Glass and S. Agatonovic-Kustrin (2003). "Prediction of drug bioavailability based on molecular structure." Analytica Chimica Acta **485**(1): 89-102.	Title
807	Turner, L. (1998). Antihistamines. Side Effects of Drugs Annual. J. K. Aronson, Elsevier. **21:** 170-178.	Title
808	Uesawa, Y., S. Hishinuma and M. Shoji (2014). "Molecular Determinants Responsible for Sedative and Non-sedative Properties of Histamine H1–Receptor Antagonists." Journal of Pharmacological Sciences **124**(2): 160-168.	Title
809	Ujie, K., M. Oda, M. Kobayashi and H. Saitoh (2008). "Relative contribution of absorptive and secretory transporters to the intestinal absorption of fexofenadine in rats." International Journal of Pharmaceutics **361**(1): 7-11.	Animal

810	Usui, S., Q. Zhu, H. Komori, Y. Iwamoto, T. Nishiuchi, Y. Shirasaka and I. Tamai (2023). "Apple-derived extracellular vesicles modulate the expression of human intestinal bile acid transporter ASBT/SLC10A2 via downregulation of transcription factor RARα." Drug Metabolism and Pharmacokinetics **52**: 100512.	Title
811	Valko, K., E. Chiarparin, S. Nunhuck and D. Montanari (2012). "In Vitro Measurement of Drug Efficiency Index to Aid Early Lead Optimization." Journal of Pharmaceutical Sciences **101**(11): 4155-4169.	Title
812	van de Steeg, E., J. Venhorst, H. T. Jansen, I. H. G. Nooijen, J. DeGroot, H. M. Wortelboer and M. L. H. Vlaming (2015). "Generation of Bayesian prediction models for OATP-mediated drug–drug interactions based on inhibition screen of OATP1B1, OATP1B1∗15 and OATP1B3." European Journal of Pharmaceutical Sciences **70**: 29-36.	Title
813	van Gelder, M. M. H. J., L. A. A. de Jong, B. te Winkel, E. J. H. Olyslager, S. Vorstenbosch, E. P. van Puijenbroek, A. L. M. Verbeek and N. Roeleveld (2019). "Assessment of medication use during pregnancy by Web-based questionnaires, pharmacy records and serum screening." Reproductive Toxicology **84**: 93-97.	Title
814	Varma, M. V. S. and R. Panchagnula (2005). "Prediction of in vivo intestinal absorption enhancement on P-glycoprotein inhibition, from rat in situ permeability." Journal of Pharmaceutical Sciences **94**(8): 1694-1704.	Animal
815	Velický, M., K. Y. Tam and R. A. W. Dryfe (2011). "In situ artificial membrane permeation assay under hydrodynamic control: Correlation between drug in vitro permeability and fraction absorbed in humans." European Journal of Pharmaceutical Sciences **44**(3): 299-309.	Title
816	Venkataramanan, R., B. Komoroski and S. Strom (2006). "In vitro and in vivo assessment of herb drug interactions." Life Sciences **78**(18): 2105-2115.	Title
817	Vermeeren, A. and J. F. O'Hanlon (1998). "Fexofenadine's effects, alone and with alcohol, on actual driving and psychomotor performance." Journal of Allergy and Clinical Immunology **101**(3): 306-311.	Abstract
818	Verster, J. C., E. R. Volkerts, A. W. A. A. van Oosterwijck, M. Aarab, S. I. R. Bijtjes, A. M. De Weert, E. J. E. Eijken and M. N. Verbaten (2003). "Acute and subchronic effects of levocetirizine and diphenhydramine on memory functioning, psychomotor performance, and mood." Journal of Allergy and Clinical Immunology **111**(3): 623-627.	Title
819	Vozmediano, V., J. C. Lukas, E. Encinas, S. Schmidt, A. Sologuren, R. Valiente, L. Labeaga, C. Campo and M. Rodriguez (2019). "Model-informed pediatric development applied to bilastine: Analysis of the clinical PK data and confirmation of the dose selected for the target population." European Journal of Pharmaceutical Sciences **128**: 180-192.	Title
820	Vraka, C., S. Mijailovic, V. Fröhlich, M. Zeilinger, E.-M. Klebermass, W. Wadsak, K.-H. Wagner, M. Hacker and M. Mitterhauser (2018). "Expanding LogP: Present possibilities." Nuclear Medicine and Biology **58**: 20-32.	Title

821	Wada, A., T. Sakaeda, K. Takara, M. Hirai, T. Kimura, N. Ohmoto, J. Zhou, T. Nakamura, H. Kobayashi, N. Okamura, T. Yagami and K. Okumura (2002). "Effects of St John's Wort and Hypericin on Cytotoxicity of Anticancer Drugs." Drug Metabolism and Pharmacokinetics **17**(5): 467-474.	Title
822	Wagner, D., H. Spahn-Langguth, A. Hanafy, A. Koggel and P. Langguth (2001). "Intestinal drug efflux: formulation and food effects." Advanced Drug Delivery Reviews **50**: S13-S31.	Title
823	Walsh, G. M. (2003). Antihistamines (H1 receptor antagonists). Side Effects of Drugs Annual. J. K. Aronson, Elsevier. **26:** 180-185.	Title
824	Wang, Q., R. Strab, P. Kardos, C. Ferguson, J. Li, A. Owen and I. J. Hidalgo (2008). "Application and limitation of inhibitors in drug–transporter interactions studies." International Journal of Pharmaceutics **356**(1): 12-18.	Title
825	Wang, X.-D., J.-L. Li, Y. Lu, X. Chen, M. Huang, B. Chowbay and S.-F. Zhou (2007). "Rapid and simultaneous determination of nifedipine and dehydronifedipine in human plasma by liquid chromatography–tandem mass spectrometry: Application to a clinical herb–drug interaction study." Journal of Chromatography B **852**(1): 534-544.	Title
826	Wang, Y., H. Wu, P. Chen, W. Su, W. Peng and P. Li (2021). "Fertility and early embryonic development toxicity assessment of naringin in Sprague-Dawley rats." Regulatory Toxicology and Pharmacology **123**: 104938.	Animal
827	Wang, Y.-M., W. Lin, S. C. Chai, J. Wu, S. S. Ong, E. G. Schuetz and T. Chen (2013). "Piperine activates human pregnane X receptor to induce the expression of cytochrome P450 3A4 and multidrug resistance protein 1." Toxicology and Applied Pharmacology **272**(1): 96-107.	Title
828	Wason, S., J. L. DiGiacinto and M. W. Davis (2012). "Effects of Grapefruit and Seville Orange Juices on the Pharmacokinetic Properties of Colchicine in Healthy Subjects." Clinical Therapeutics **34**(10): 2161-2173.	Title
829	Watanabe, A., R. Watari, K. Ogawa, R. Shimizu, Y. Tanaka, N. Takai, K.-I. Nezasa and Y. Yamaguchi (2015). "Using Improved Serial Blood Sampling Method of Mice to Study Pharmacokinetics and Drug–Drug Interaction." Journal of Pharmaceutical Sciences **104**(3): 955-961.	Animal
830	Watson, W. A., T. L. Litovitz, G. C. Rodgers, W. Klein-Schwartz, N. Reid, J. Youniss, A. Flanagan and K. M. Wruk (2005). "2004 Annual report of the American Association of Poison Control Centers Toxic Exposure Surveillance System." The American Journal of Emergency Medicine **23**(5): 589-666.	Title
831	Wieland, K., A. M. T. Laak, M. J. Smit, R. Kühne, H. Timmerman and R. Leurs (1999). "Mutational Analysis of the Antagonist-binding Site of the Histamine H1 Receptor*." Journal of Biological Chemistry **274**(42): 29994-30000.	Title

832	Wilk-Zasadna, I., C. Bernasconi, O. Pelkonen and S. Coecke (2015). "Biotransformation in vitro: An essential consideration in the quantitative in vitro-to-in vivo extrapolation (QIVIVE) of toxicity data." Toxicology **332**: 8-19.	Title
833	Wilson, C. G., L. Aarons, P. Augustijns, J. Brouwers, A. S. Darwich, T. De Waal, G. Garbacz, S. Hansmann, D. Hoc, A. Ivanova, M. Koziolek, C. Reppas, P. Schick, M. Vertzoni and J. A. García-Horsman (2022). "Integration of advanced methods and models to study drug absorption and related processes: An UNGAP perspective." European Journal of Pharmaceutical Sciences **172**: 106100.	Title
834	Winek, C. L., W. W. Wahba, C. L. Winek and T. W. Balzer (2001). "Drug and chemical blood-level data 2001." Forensic Science International **122**(2): 107-123.	Title
835	Wiśniowska, B. and S. Polak (2016). "Virtual Clinical Trial Toward Polytherapy Safety Assessment: Combination of Physiologically Based Pharmacokinetic/Pharmacodynamic-Based Modeling and Simulation Approach With Drug-Drug Interactions Involving Terfenadine as an Example." Journal of Pharmaceutical Sciences **105**(11): 3415-3424.	Title
836	Wolfson, A. R., D. Wong, E. M. Abrams, S. Waserman and G. L. Sussman (2022). "Diphenhydramine: Time to Move on?" The Journal of Allergy and Clinical Immunology: In Practice **10**(12): 3124-3130.	Title
837	Wu, K.-M. and J. G. Farrelly (2007). "Regulatory perspectives of Type II prodrug development and time-dependent toxicity management: Nonclinical Pharm/Tox analysis and the role of comparative toxicology." Toxicology **236**(1): 1-6.	Title
838	Wu, X., J. Ma, Y. Ye and G. Lin (2016). "Transporter modulation by Chinese herbal medicines and its mediated pharmacokinetic herb–drug interactions." Journal of Chromatography B **1026**: 236-253.	Title
839	Xia, C., J. Sun, G. Wang, L. Shang, X. Zhang, R. Zhang, X. Wang, H. Hao and L. Xie (2009). "Differential effect of Shenmai injection, a herbal preparation, on the cytochrome P450 3A-mediated 1′-hydroxylation and 4-hydroxylation of midazolam." Chemico-Biological Interactions **180**(3): 440-448.	Title
840	Xu, M., Y. Chen, X. Xi, C. Jiang, Q. Zhang, T. Wu, J. Chu, G. Dai, Y. Bai, Q. Yu, J. Zou and W. Ju (2022). "In vitro inhibitory effects of components from Salvia miltiorrhiza on catalytic activity of three human Arachidonic acid ω-hydroxylases." Drug Metabolism and Pharmacokinetics **43**: 100402.	Title
841	Yamane, N., T. Takami, Z. Tozuka, Y. Sugiyama, A. Yamazaki and Y. Kumagai (2009). "Microdose Clinical Trial: Quantitative Determination of Nicardipine and Prediction of Metabolites in Human Plasma." Drug Metabolism and Pharmacokinetics **24**(4): 389-403.	Title
842	Yang, C.-W., Y.-T. Cho and C.-Y. Chu (2017). "Efficacy of omalizumab treatment for patients with chronic idiopathic urticaria (CIU)/chronic spontaneous urticaria (CSU) in Taiwan." Dermatologica Sinica **35**(4): 182-186.	Title

843	Yano, K., S. Mita, K. Morimoto, T. haraguchi, H. arakawa, M. Yoshida, F. Yamashita, T. Uchida and T. Ogihara (2015). "Multiple Linear Regression Analysis Indicates Association of P-Glycoprotein Substrate or Inhibitor Character with Bitterness Intensity Measured with a Sensor." Journal of Pharmaceutical Sciences **104**(9): 2789-2794.	Title
844	Yen, C.-C., Y.-T. Liu, Y.-J. Lin, Y.-C. Yang, C.-C. Chen, H.-T. Yao, H.-W. Chen and C.-K. Lii (2019). "Bioavailability of the diterpenoid 14-deoxy-11,12-didehydroandrographolide in rats and up-regulation of hepatic drug-metabolizing enzyme and drug transporter expression." Phytomedicine **61**: 152841.	Animal
845	Yoshida, S., T. Honjo, K. Iino, R. Ishibe, S. Leo, T. Shimada, T. Watanabe, M. Ishikawa, K. Maeda, H. Kusuhara, N. Shiraki and S. Kume (2021). "Generation of Human-Induced Pluripotent Stem Cell-Derived Functional Enterocyte-Like Cells for Pharmacokinetic Studies." Stem Cell Reports **16**(2): 295-308.	Title
846	Yoshimatsu, H., K. Ishii and S. Yamashita (2020). "Extent and rate of oral drug absorption in minipigs: Comparison with mice, rats and humans." Journal of Drug Delivery Science and Technology **58**: 101743.	Animal
847	Yoshimatsu, H., Y. Konno, K. Ishii, M. Satsukawa and S. Yamashita (2016). "Usefulness of minipigs for predicting human pharmacokinetics: Prediction of distribution volume and plasma clearance." Drug Metabolism and Pharmacokinetics **31**(1): 73-81.	Animal
848	Yoshizato, T., K. Tsutsumi, T. Kotegawa, H. Imai and S. Nakano (2014). "Determination of domperidone in human plasma using high performance liquid chromatography with fluorescence detection for clinical application." Journal of Chromatography B **961**: 86-90.	Title
849	Yu, J., H. Chen, L. Jiang, J. Wang, J. Dai and J. Wang (2019). "Codelivery of Adriamycin and P-gp Inhibitor Quercetin Using PEGylated Liposomes to Overcome Cancer Drug Resistance." Journal of Pharmaceutical Sciences **108**(5): 1788-1799.	Title
850	zhang, A., Y. Jia, Q. Xu, C. Wang, Q. Liu, Q. Meng, J. Peng, H. Sun, P. Sun, X. Huo and K. Liu (2016). "Dioscin protects against ANIT–induced cholestasis via regulating Oatps, Mrp2 and Bsep expression in rats." Toxicology and Applied Pharmacology **305**: 127-135.	Animal
851	Zhang, B. and V. M. Lauschke (2019). "Genetic variability and population diversity of the human SLCO (OATP) transporter family." Pharmacological Research **139**: 550-559.	Title
852	Zhang, J., M. T. Empl, M. Schneider, B. Schröder, J. Stadnicka-Michalak, G. Breves, P. Steinberg and S. J. Sturla (2019). "Gut microbial transformation of the dietary mutagen MeIQx may reduce exposure levels without altering intestinal transport." Toxicology in Vitro **59**: 238-245.	Title
853	Zhang, L., J.-P. Zhou and J. Yao (2015). "Improved anti-tumor activity and safety profile of a paclitaxel-loaded glycyrrhetinic acid-graft-hyaluronic acid conjugate as a synergistically targeted drug delivery system." Chinese Journal of Natural Medicines **13**(12): 915-924.	Title

854	Zhao, R. and G. M. Pollack (2009). "Regional differences in capillary density, perfusion rate, and P-glycoprotein activity: A quantitative analysis of regional drug exposure in the brain." Biochemical Pharmacology **78**(8): 1052-1059.	Title
855	Zheng, H., S. Webber, A. Zeevi, E. Schuetz, J. Zhang, J. Lamba, P. Bowman and G. J. Burckart (2002). "The MDR1 polymorphisms at exons 21 and 26 predict steroid weaning in pediatric heart transplant patients." Human Immunology **63**(9): 765-770.	Title
856	Zhong, L., C.-Y. Ma, H. Zhang, L.-J. Yang, H.-L. Wan, Q.-Q. Xie, L.-L. Li and S.-Y. Yang (2011). "A prediction model of substrates and non-substrates of breast cancer resistance protein (BCRP) developed by GA–CG–SVM method." Computers in Biology and Medicine **41**(11): 1006-1013.	Title
857	Zhu, B., M. Xue, B. Liu, Q. Li and X. Guo (2019). "Enantioselective separation of eight antihistamines with α1-acid glycoprotein-based chiral stationary phase by HPLC: Development and validation for the enantiomeric quality control." Journal of Pharmaceutical and Biomedical Analysis **176**: 112803.	Title
858	Zhu, Y., Q. Meng, C. Wang, Q. Liu, H. Sun, T. Kaku and K. Liu (2012). "Organic anion transporters involved in the excretion of bestatin in the kidney." Peptides **33**(2): 265-271.	Title
859	Zolk, O., R. Schnepf, M. Muschler, M. F. Fromm, O. Wendler, M. Traxdorf, H. Iro and J. Zenk (2013). "Transporter Gene Expression in Human Head and Neck Squamous Cell Carcinoma and Associated Epigenetic Regulatory Mechanisms." The American Journal of Pathology **182**(1): 234-243.	Title
860	Zou, P., X. Liu, S. Wong, M. R. Feng and B. M. Liederer (2013). "Comparison of In Vitro-In Vivo Extrapolation of Biliary Clearance Using an Empirical Scaling Factor Versus Transport-Based Scaling Factors in Sandwich-Cultured Rat Hepatocytes." Journal of Pharmaceutical Sciences **102**(8): 2837-2850.	Animal
861	Zuo, X.-c., W.-l. Zhang, H. Yuan, J. S. Barrett, Y. Hua, Z.-j. Huang, H.-h. Zhou, Q. Pei, C.-x. Guo, J.-l. Wang and G.-p. Yang (2014). "ABCB1 Polymorphism and Gender Affect the Pharmacokinetics of Amlodipine in Chinese Patients with Essential Hypertension: A Population Analysis." Drug Metabolism and Pharmacokinetics **29**(4): 305-311.	Title

Supplementary Table S3. JADAD Scoring

JADAD Questions						
Reference	Was the study described as randomized	Was the method used to generate the sequence of randomization described and appropriate	Was the study described as double blind	Was the method of double blinding described and appropriate	Was there a description of withdrawls and dropouts	Total score
Simons et al., 1996 [38]	1	1	0	0	0	2
Russell et al., 1998 [1]	1	1	1	1	0	4
Robbins et al., 1998 [2]	1	1	0	0	1	3
Gupta et al., 2001 [3]	1	1	0	0	0	2
Hamman et al., 2001 [4]	0	0	0	0	0	0
Drescher et al., 2002 [5]	0	0	0	0	0	0
Wang et al., 2002 [6]	0	0	0	0	0	0
Banfield et al., 2002 [7]	1	1	0	0	1	3
Dresser et al., 2002 [8]	1	1	0	0	0	2
Hofmann et al., 2002 [9]	0	0	0	0	0	0
Tannergren et al., 2003 [10]	0	0	0	0	0	0
Dresser et al., 2003 [11]	0	0	0	0	0	0
So-Young Yi et al., 2004 [12]	0	0	0	0	0	0
Shon et al., 2005 [13]	1	1	1	1	0	4
Boyle et al., 2005 [14]	1	1	1	1	0	4
Xie et al., 2005 [15]	0	0	0	0	0	0
Yasui-Furukori et al., 2005 [16]	0	0	0	0	0	0
Dresser et al., 2005 [17]	1	1	0	0	0	2

Rolf et al., 2006 [18]	1	1	0	0	0	2
Lemma et al., 2006 [19]	0	0	0	0	0	0
M. Shimizu et al., 2006 [20]	1	1	0	0	1	3
Uno et al., 2006 [21]	1	1	0	0	0	2
Shimizu et al., 2006 [22]	1	1	0	0	0	2
Mendoza et al., 2007 [23]	1	1	0	0	0	2
Miura et al., 2007 [24]	0	0	0	0	0	0
Bailey et al., 2007 [25]	1	0	0	0	0	1
Teng et al., 2007 [26]	1	0	0	0	0	1
Robertson et al., 2008 [27]	0	0	0	0	1	1
Bharathi et al., 2008 [28]	0	0	0	0	0	0
Kharasch et al., 2008 [29]	0	0	0	0	0	0
Tateishi et al., 2008 [30]	1	1	1	1	0	4
Segall et al., 2008 [31]	0	0	0	0	1	1
Liu et al., 2009 [32]	1	1	0	0	1	3
Kharasch et al., 2009 [33]	0	0	0	0	0	0
Kim et al., 2009 [34]	1	1	0	0	0	2
Kharasch et al., 2009 [35]	0	0	0	0	0	0
Valizadeh et al., 2009 [36]	1	1	0	0	0	2
Sakugawa et al., 2009 [37]	1	1	0	0	0	2
Nolin et al., 2009 [38]	0	0	0	0	0	0
Yamada et al., 2009 [39]	1	1	0	0	0	0
Kim et al., 2010 [40]	1	1	0	0	0	2
Akamine et al., 2010 [41]	0	0	0	0	0	0
Guo et al., 2010 [42]	1	1	0	0	0	2

Yamazaki et al., 2010 [43]	1	1	0	0	0	2
Penzak et al., 2010 [44]	0	0	0	0	1	1
Lappin et al., 2010 [45]	0	0	0	0	0	0
Imanaga et al., 2010 [46]	1	1	0	0	0	2
Malati et al., 2012 [47]	0	0	0	0	1	1
Saruwatari et al., 2012 [48]	1	1	0	0	1	3
Akamine et al., 2012 [49]	1	1	0	0	0	2
Kharasch et al., 2012 [50]	0	0	0	0	0	0
Croft et al., 2012 [51]	0	0	0	0	0	0
Kharasch et al., 2013 [52]	0	0	0	0	0	0
Won et al., 2013 [53]	1	1	0	0	0	2
Zhou et al., 2013 [54]	1	1	0	0	0	2
Kusuhara et al., 2013 [55]	1	1	0	0	0	2
Ieiri et al., 2013 [56]	0	0	0	0	0	0
Muppavarapu et al., 2014 [57]	1	1	0	0	0	2
Bedada et al., 2014 [58]	0	0	0	0	0	0
Kim et al., 2014 [59]	0	0	0	0	0	0
Akamine et al., 2014 [60]	1	1	0	0	0	2
Chen et al., 2014 [61]	0	0	0	0	0	0
Qiu et al., 2014 [62]	0	0	0	0	0	0
Joy et al., 2014 [63]	0	0	0	0	0	0
Tomaru et al., 2015 [64]	0	0	0	0	0	0
Akamine et al., 2015 [65]	1	1	1	1	0	4
Akamine et al., 2015 [66]	1	1	0	0	0	2

Yehia et al., 2015 [67]	1	1	0	0	0	2
Thomson et al., 2015 [68]	0	0	0	0	0	0
Kullak-Ublick et al., 2016 [69]	0	0	0	0	0	0
Helmy et al., 2016 [70]	0	0	0	0	0	0
Luo et al., 2016 [71]	1	1	0	0	0	2
Bedada et al., 2017 [72]	0	0	0	0	0	0
Bedada et al., 2017 [73]	1	1	0	0	0	2
Cusinato et al., 2019 [74]	0	0	0	0	0	0
Calvo et al., 2019 [75]	0	0	0	0	1	1
Cusinato et al., 2019 [76]	0	0	0	0	0	0
Bosilkovska et al., 2019 [77]	1	1	0	0	0	2
Pinto et al., 2020 [79]	0	0	0	0	0	0
Egeland et al., 2020 [80]	0	0	0	0	0	0
Zhao et al., 2021 [81]	1	1	0	0	0	2
Everardo et al., 2021 [82]	1	1	0	0	1	3
Misaka et al., 2022 [83]	1	1	0	0	0	2
Rauch et al., 2023 [84]	1	1	0	0	1	3
Chretien et al., 2023 [85]	1	1	0	0	1	3

Supplementary Table S4. Critical Appraisal Skill Program (CASP)

CASP Questions											
Reference	Was there a clear statement of the aims of the research?	Is a qualitative methodology appropriate?	Was the research design appropriate to address the aims of the research?	Are the study's theoretical underpinnings clear, concictent and conceptually coherent?	Was the recruitment strategy appropriate to the aims of the search?	Was the data collected in a way that addressed the research issue?	Has the relationship between the researcher and participants been adequately considered?	Have ethical issues been taken into consideration?	Was the data analysis sufficiently rigorous?	Is there a clear statement of findings?	CASP Score
Russell et al., 1998 [1]	Y	Y	Y	Y	Y	Y	CT	Y	Y	Y	9
Robbins et al., 1998 [2]	Y	Y	Y	Y	Y	Y	CT	Y	Y	Y	9
Gupta et al., 2001 [3]	Y	Y	Y	Y	Y	Y	N	Y	Y	Y	9
Hamman et al., 2001 [4]	Y	Y	Y	Y	Y	Y	CT	Y	Y	Y	9
Drescher et al., 2002 [5]	Y	Y	CT	Y	Y	Y	CT	Y	Y	Y	8
Wang et al., 2002 [6]	N	Y	Y	Y	Y	Y	CT	Y	Y	Y	8
Banfield et al., 2002 [7]	Y	Y	Y	Y	Y	Y	CT	CT	Y	Y	8
Dresser et al., 2002 [8]	Y	Y	Y	Y	Y	Y	N	Y	Y	Y	9
Hofmann et al., 2002 [9]	Y	Y	Y	Y	CT	Y	CT	Y	Y	CT	7
Tannergren et al., 2003 [10]	Y	Y	Y	Y	Y	Y	CT	Y	Y	Y	9
Dresser et al., 2003 [11]	Y	Y	Y	Y	Y	Y	N	Y	Y	Y	9
So-Young Yi et al., 2004 [12]	Y	Y	Y	Y	Y	Y	CT	Y	Y	Y	9
Shon et al., 2005 [13]	Y	Y	Y	Y	Y	Y	CT	Y	Y	Y	9
Boyle et al., 2005 [14]	Y	Y	Y	Y	Y	Y	CT	Y	Y	Y	9

Xie et al., 2005 [15]	Y	Y	CT	Y	Y	Y	CT	Y	Y	Y	8
Yasui-Furukori et al., 2005 [16]	Y	Y	Y	Y	Y	Y	CT	Y	Y	Y	9
Dresser et al., 2005 [17]	Y	Y	Y	Y	Y	Y	CT	Y	Y	Y	9
Rolf et al., 2006 [18]	Y	Y	Y	Y	Y	Y	CT	Y	Y	Y	9
Lemma et al., 2006 [19]	Y	Y	Y	Y	Y	Y	CT	Y	Y	Y	9
M. Shimizu et al., 2006 [20]	Y	Y	Y	Y	Y	Y	CT	Y	Y	Y	9
Uno et al., 2006 [21]	Y	Y	Y	Y	Y	Y	CT	Y	Y	Y	9
Shimizu et al., 2006 [22]	Y	Y	Y	Y	Y	Y	CT	Y	Y	Y	9
Mendoza et al., 2007 [23]	Y	Y	Y	Y	Y	CT	CT	Y	Y	Y	8
Miura et al., 2007 [24]	Y	Y	CT	Y	Y	Y	CT	Y	Y	Y	8
Bailey et al., 2007 [25]	N	CT	Y	Y	Y	Y	CT	Y	Y	Y	8
Teng et al., 2007 [26]	Y	Y	Y	Y	Y	Y	CT	CT	Y	Y	8
Robertson et al., 2008 [27]	Y	Y	Y	Y	Y	Y	CT	Y	Y	Y	9
Bharathi et al., 2008 [28]	Y	Y	CT	Y	Y	Y	CT	Y	Y	Y	8
Kharasch et al., 2008 [29]	Y	Y	CT	Y	CT	Y	CT	Y	Y	Y	7
Tateishi et al., 2008 [30]	Y	Y	Y	Y	Y	Y	CT	Y	Y	Y	9
Segall et al., 2008 [31]	Y	Y	Y	Y	Y	Y	Y	Y	Y	Y	10
Liu et al., 2009 [32]	Y	Y	Y	Y	Y	Y	CT	Y	Y	Y	9
Kharasch et al., 2009 [33]	Y	Y	Y	CT	Y	Y	CT	Y	Y	Y	8
Kim et al., 2009 [34]	Y	Y	Y	Y	Y	Y	CT	Y	Y	Y	9
Kharasch et al., 2009 [35]	Y	Y	Y	CT	Y	Y	CT	Y	Y	Y	8
Valizadeh et al., 2009 [36]	Y	Y	Y	Y	Y	Y	CT	Y	Y	Y	9
Sakugawa et al., 2009 [37]	Y	Y	Y	Y	Y	Y	CT	Y	Y	Y	9
Nolin et al., 2009 [38]	Y	Y	Y	Y	Y	Y	CT	Y	Y	Y	9
Yamada et al., 2009 [39]	Y	Y	Y	Y	Y	Y	CT	Y	Y	Y	9

Kim et al., 2010 [40]	Y	Y	Y	Y	Y	Y	CT	Y	Y	Y	9
Akamine et al., 2010 [41]	Y	Y	CT	Y	Y	Y	CT	Y	Y	Y	8
Guo et al., 2010 [42]	Y	Y	CT	Y	Y	Y	CT	Y	Y	Y	8
Yamazaki et al., 2010 [43]	Y	Y	Y	Y	Y	Y	CT	Y	Y	Y	9
Penzak et al., 2010 [44]	Y	Y	Y	Y	Y	Y	CT	Y	Y	Y	9
Lappin et al., 2010 [45]	N	Y	CT	Y	Y	Y	CT	Y	Y	Y	7
Imanaga et al., 2010 [46]	Y	Y	Y	Y	Y	Y	CT	Y	Y	Y	9
Malati et al., 2012 [47]	Y	Y	Y	Y	Y	Y	CT	Y	Y	Y	9
Saruwatari et al., 2012 [48]	Y	Y	Y	Y	Y	Y	CT	Y	Y	Y	9
Akamine et al., 2012 [49]	Y	Y	Y	Y	Y	Y	CT	Y	Y	Y	9
Kharasch et al., 2012 [50]	Y	Y	Y	Y	Y	Y	CT	Y	Y	Y	9
Croft et al., 2012 [51]	Y	Y	Y	Y	Y	Y	CT	Y	Y	Y	9
Kharasch et al., 2013 [52]	Y	Y	Y	Y	Y	Y	CT	Y	Y	Y	9
Won et al., 2013 [53]	Y	Y	Y	CT	Y	Y	CT	Y	Y	Y	8
Zhou et al., 2013 [54]	Y	Y	Y	Y	Y	Y	CT	Y	Y	Y	9
Kusuhara et al., 2013 [55]	Y	Y	Y	Y	Y	Y	CT	Y	Y	Y	9
Ieiri et al., 2013 [56]	Y	Y	Y	CT	Y	Y	CT	Y	Y	Y	8
Muppavarapu et al., 2014 [57]	Y	Y	Y	CT	Y	Y	CT	Y	Y	Y	8
Bedada et al., 2014 [58]	Y	Y	Y	Y	Y	Y	CT	Y	Y	Y	9
Kim et al., 2014 [59]	Y	Y	Y	Y	Y	Y	CT	Y	Y	Y	9
Akamine et al., 2014 [60]	Y	Y	Y	Y	Y	Y	CT	Y	Y	Y	9
Chen et al., 2014 [61]	Y	Y	CT	Y	Y	Y	CT	Y	Y	Y	8
Qiu et al., 2014 [62]	Y	Y	Y	Y	Y	Y	CT	Y	Y	Y	9
Joy et al., 2014 [63]	Y	Y	CT	Y	Y	Y	CT	Y	Y	Y	8

Tomaru et al., 2015 [64]	Y	Y	Y	Y	Y	Y	CT	Y	Y	Y	9
Akamine et al., 2015 [65]	Y	Y	Y	Y	Y	Y	CT	Y	Y	Y	9
Akamine et al., 2015 [66]	Y	Y	Y	Y	Y	Y	CT	Y	Y	Y	9
Yehia et al., 2015 [67]	Y	Y	Y	Y	Y	Y	CT	Y	Y	Y	9
Thomson et al., 2015 [68]	Y	Y	CT	Y	Y	Y	CT	Y	Y	Y	8
Kullak-Ublick et al., 2016 [69]	Y	Y	Y	Y	Y	Y	CT	Y	Y	Y	9
Helmy et al., 2016 [70]	Y	CT	CT	Y	Y	Y	CT	Y	Y	Y	7
Luo et al., 2016 [71]	Y	Y	Y	Y	Y	Y	CT	Y	Y	Y	9
Bedada et al., 2017 [72]	Y	Y	Y	Y	Y	Y	CT	Y	Y	Y	9
Bedada et al., 2017 [73]	Y	Y	Y	Y	Y	Y	CT	Y	Y	Y	9
Cusinato et al., 2019 [74]	Y	Y	Y	Y	Y	Y	CT	Y	Y	Y	9
Calvo et al., 2019 [75]	Y	Y	CT	Y	Y	Y	CT	Y	Y	Y	8
Cusinato et al., 2019 [76]	Y	Y	CT	Y	Y	Y	CT	Y	Y	Y	8
Bosilkovska et al., 2019 [77]	Y	Y	Y	Y	Y	Y	CT	Y	Y	Y	9
Simons et al., 1996 [38]	Y	Y	Y	Y	Y	Y	CT	Y	Y	Y	9
Pinto et al., 2020 [79]	Y	Y	Y	Y	Y	Y	CT	Y	Y	Y	9
Egeland et al., 2020 [80]	Y	Y	Y	Y	Y	Y	CT	Y	Y	Y	9
Zhao et al., 2021 [81]	Y	Y	Y	Y	Y	Y	CT	Y	Y	Y	9
Everardo et al., 2021 [82]	Y	Y	Y	Y	Y	Y	CT	Y	Y	Y	9
Misaka et al., 2022 [83]	Y	Y	Y	Y	Y	Y	CT	Y	CT	Y	8
Rauch et al., 2023	Y	Y	Y	Y	Y	Y	CT	Y	Y	Y	9
Chretien et al., 2023 [85]	Y	Y	Y	Y	Y	Y	CT	Y	Y	Y	9

Y= Yes; N=No; CT= Can’t tell

Supplementary Table S5. Critical Appraisal Clinical Pharmacokinetic (CACPK) Tool

**Critical Appraisal Clinical Pharmacokinetic Tool**																						
Reference	Q 1	Q 2	Q 3	Q 4	Q 5	Q 6	Q 7	Q 8	Q 9	Q 10	Q 11	Q 12	Q 13	Q 14	Q 15	Q 16	Q 17	Q 18	Q 19	Q 20	Q 21	Total Score
Russell et al., 1998 [1]	Y	Y	Y	Y	Y	Y	Y	Y	Y	Y	Y	IDK	Y	N	IDK	Y	N	Y	Y	IDK	Y	16
Robbins et al., 1998 [2]	Y	Y	Y	Y	Y	N	Y	Y	Y	Y	Y	IDK	Y	IDK	IDK	Y	N	Y	Y	IDK	Y	15
Gupta et al., 2001 [3]	Y	Y	Y	Y	Y	Y	Y	Y	Y	Y	Y	IDK	Y	N	IDK	Y	N	Y	Y	IDK	Y	16
Hamman et al., 2001 [4]	Y	Y	Y	Y	Y	N	Y	N	Y	Y	Y	IDK	Y	N	IDK	Y	N	Y	Y	IDK	Y	14
Drescher et al., 2002 [5]	Y	Y	N	Y	Y	N	Y	Y	Y	Y	Y	N	Y	N	IDK	Y	Y	Y	Y	IDK	Y	15
Wang et al., 2002 [6]	Y	Y	Y	Y	Y	Y	Y	Y	Y	Y	N	IDK	N	N	N	Y	Y	Y	Y	IDK	Y	15
Banfield et al., 2002 [7]	Y	Y	Y	Y	Y	Y	Y	Y	Y	Y	Y	IDK	IDK	N	N	Y	N	IDK	Y	IDK	Y	14
Dresser et al., 2002 [8]	Y	Y	Y	Y	Y	N	Y	N	Y	Y	Y	IDK	Y	N	IDK	Y	N	Y	Y	IDK	Y	14
Hofmann et al., 2002 [9]	Y	Y	N	Y	Y	N	Y	N	Y	Y	Y	IDK	N	N	N	Y	N	Y	Y	IDK	Y	12
Tannergren et al., 2003 [10]	Y	Y	Y	Y	Y	N	Y	Y	Y	Y	Y	IDK	Y	N	N	Y	Y	Y	Y	IDK	Y	16
Dresser et al., 2003 [11]	Y	Y	Y	Y	Y	Y	Y	Y	Y	Y	Y	IDK	Y	N	N	Y	Y	IDK	Y	IDK	Y	16

So-Young Yi et al., 2004 [12]	Y	Y	Y	Y	Y	N	Y	Y	Y	Y	Y	IDK	Y	IDK	IDK	Y	N	Y	Y	IDK	Y	15
Shon et al., 2005 [13]	Y	Y	Y	Y	Y	N	Y	Y	Y	Y	Y	IDK	Y	IDK	IDK	Y	N	IDK	Y	IDK	Y	14
Boyle et al., 2005 [14]	Y	Y	Y	Y	Y	Y	Y	Y	Y	Y	Y	IDK	Y	IDK	IDK	Y	N	Y	Y	IDK	Y	16
Xie et al., 2005 [15]	Y	Y	IDK	Y	Y	Y	Y	N	Y	Y	N	IDK	N	IDK	IDK	Y	Y	Y	Y	IDK	Y	13
Yasui-Furukori et al., 2005 [16]	Y	Y	Y	Y	Y	N	Y	N	Y	Y	Y	IDK	Y	IDK	IDK	Y	N	Y	Y	IDK	Y	14
Dresser et al., 2005 [17]	Y	Y	Y	Y	Y	N	Y	IDK	Y	Y	Y	IDK	N	IDK	IDK	Y	N	Y	Y	IDK	Y	13
Rolf et al., 2006 [18]	Y	Y	Y	Y	Y	N	Y	Y	Y	Y	Y	IDK	Y	IDK	IDK	Y	N	Y	Y	IDK	Y	15
Lemma et al., 2006 [19]	Y	Y	Y	Y	Y	Y	Y	N	Y	Y	Y	IDK	Y	IDK	IDK	Y	N	IDK	Y	IDK	Y	14
M. Shimizu et al., 2006 [20]	Y	Y	Y	Y	Y	N	Y	N	Y	Y	Y	IDK	Y	N	IDK	Y	Y	Y	Y	IDK	Y	15
Uno et al., 2006 [21]	Y	Y	Y	Y	Y	N	Y	N	Y	Y	Y	IDK	Y	N	IDK	Y	Y	Y	Y	IDK	Y	15
Shimizu et al., 2006 [22]	Y	Y	Y	Y	Y	N	Y	N	Y	Y	Y	IDK	N	N	IDK	Y	Y	Y	Y	IDK	Y	14
Mendoza et al., 2007 [23]	Y	Y	Y	Y	Y	N	Y	N	Y	Y	Y	IDK	Y	N	IDK	Y	Y	Y	Y	IDK	Y	15
Miura et al., 2007 [24]	Y	Y	IDK	Y	Y	N	Y	N	Y	Y	N	IDK	Y	N	IDK	Y	Y	Y	Y	IDK	Y	13
Bailey et al., 2007 [25]	Y	Y	IDK	Y	Y	N	Y	N	Y	Y	Y	IDK	N	N	IDK	Y	Y	IDK	Y	IDK	Y	12
Teng et al., 2007 [26]	Y	Y	Y	Y	Y	N	N	N	Y	Y	Y	IDK	Y	N	IDK	Y	N	IDK	Y	IDK	Y	12

Robertson et al., 2008 [27]	Y	Y	Y	Y	Y	Y	Y	Y	Y	Y	Y	IDK	Y	N	IDK	Y	N	Y	Y	IDK	Y	16
Bharathi et al., 2008 [28]	Y	Y	IDK	Y	Y	N	N	N	Y	Y	Y	IDK	Y	N	IDK	Y	N	Y	Y	IDK	Y	12
Kharasch et al., 2008 [29]	Y	Y	IDK	Y	Y	Y	Y	N	IDK	Y	Y	IDK	Y	N	IDK	Y	Y	Y	Y	IDK	Y	14
Tateishi et al., 2008 [30]	Y	Y	Y	Y	Y	N	Y	N	Y	Y	Y	IDK	Y	N	IDK	Y	Y	Y	Y	IDK	Y	15
Segall et al., 2008 [31]	Y	Y	Y	Y	Y	Y	Y	Y	Y	Y	Y	IDK	IDK	N	IDK	Y	N	IDK	Y	IDK	Y	14
Liu et al., 2009 [32]	Y	Y	Y	Y	Y	Y	Y	N	Y	Y	Y	IDK	Y	N	IDK	Y	N	Y	Y	IDK	Y	15
Kharasch et al., 2009 [33]	Y	Y	Y	Y	Y	Y	Y	N	Y	Y	IDK	IDK	Y	N	IDK	Y	Y	IDK	Y	IDK	Y	14
Kim et al., 2009 [34]	Y	Y	Y	Y	Y	N	Y	N	Y	Y	Y	IDK	Y	N	IDK	Y	Y	Y	Y	IDK	Y	15
Kharasch et al., 2009 [35]	Y	Y	Y	Y	IDK	Y	N	N	Y	Y	IDK	IDK	IDK	N	IDK	Y	Y	Y	Y	IDK	Y	12
Valizadeh et al., 2009 [36]	Y	Y	Y	Y	Y	Y	Y	N	Y	Y	Y	IDK	Y	N	IDK	Y	Y	Y	Y	IDK	Y	16
Sakugawa et al., 2009 [37]	Y	Y	Y	Y	Y	N	Y	N	Y	Y	Y	IDK	Y	N	IDK	Y	Y	Y	Y	IDK	Y	15
Nolin et al., 2009 [38]	Y	Y	Y	Y	Y	N	Y	N	Y	Y	Y	IDK	Y	N	IDK	Y	N	Y	Y	IDK	Y	14
Yamada et al., 2009 [39]	Y	Y	Y	Y	Y	N	Y	N	Y	Y	Y	IDK	Y	N	IDK	Y	Y	Y	Y	IDK	Y	15
Kim et al., 2010 [40]	Y	Y	Y	Y	Y	N	Y	N	Y	Y	Y	IDK	Y	N	IDK	Y	N	Y	Y	IDK	Y	14

Akamine et al., 2010 [41]	Y	Y	IDK	Y	Y	N	Y	N	Y	Y	Y	IDK	Y	N	IDK	Y	N	Y	Y	IDK	Y	13
Guo et al., 2010 [42]	Y	Y	IDK	Y	Y	N	Y	N	Y	Y	IDK	IDK	Y	N	IDK	Y	N	Y	Y	IDK	Y	12
Yamazaki et al., 2010 [43]	Y	Y	Y	Y	Y	Y	Y	N	Y	Y	Y	IDK	N	N	IDK	Y	N	IDK	Y	IDK	Y	13
Penzak et al., 2010 [44]	Y	Y	Y	Y	Y	Y	Y	N	Y	Y	Y	IDK	Y	N	IDK	Y	N	Y	Y	IDK	Y	15
Lappin et al., 2010 [45]	N	N	IDK	Y	Y	N	Y	N	Y	Y	Y	IDK	Y	N	IDK	Y	N	IDK	Y	IDK	Y	10
Imanaga et al., 2010 [46]	Y	Y	Y	Y	Y	Y	Y	N	Y	Y	Y	IDK	Y	N	IDK	Y	N	Y	Y	IDK	Y	15
Malati et al., 2012 [47]	Y	Y	Y	Y	Y	Y	Y	N	Y	Y	Y	IDK	Y	N	IDK	Y	N	Y	Y	IDK	Y	15
Saruwatari et al., 2012 [48]	Y	Y	Y	Y	Y	Y	Y	N	Y	Y	Y	IDK	Y	N	IDK	Y	Y	Y	Y	IDK	Y	16
Akamine et al., 2012 [49]	Y	Y	Y	Y	Y	N	Y	N	Y	Y	IDK	IDK	Y	N	IDK	Y	Y	Y	Y	IDK	Y	14
Kharasch et al., 2012 [50]	Y	Y	Y	Y	Y	N	Y	N	Y	Y	Y	IDK	Y	N	IDK	Y	Y	Y	Y	IDK	Y	15
Croft et al., 2012 [51]	Y	Y	Y	Y	Y	N	Y	N	Y	Y	Y	IDK	Y	N	IDK	Y	N	Y	Y	IDK	Y	14
Kharasch et al., 2013 [52]	Y	IDK	Y	Y	Y	N	Y	N	Y	Y	Y	IDK	Y	N	IDK	Y	N	Y	Y	IDK	Y	13
Won et al., 2013 [53]	Y	Y	Y	Y	Y	N	Y	N	IDK	IDK	IDK	IDK	Y	N	IDK	Y	N	Y	Y	IDK	Y	11
Zhou et al., 2013 [54]	Y	Y	Y	Y	Y	Y	Y	N	Y	Y	Y	IDK	Y	N	IDK	Y	N	Y	Y	IDK	Y	15

Kusuhara et al., 2013 [55]	Y	Y	Y	Y	Y	N	Y	N	Y	Y	Y	IDK	Y	N	IDK	Y	Y	Y	Y	IDK	Y	15
Ieiri et al., 2013 [56]	Y	Y	Y	Y	Y	N	Y	N	Y	Y	Y	IDK	Y	N	IDK	Y	N	Y	Y	IDK	Y	14
Muppavarapu et al., 2014 [57]	Y	Y	Y	Y	Y	N	Y	N	Y	Y	Y	IDK	Y	N	IDK	Y	N	Y	Y	IDK	Y	14
Bedada et al., 2014 [58]	Y	Y	Y	Y	Y	N	Y	N	Y	Y	Y	IDK	Y	N	IDK	Y	N	Y	Y	IDK	Y	14
Kim et al., 2014 [59]	Y	Y	Y	Y	Y	N	Y	N	Y	Y	Y	IDK	Y	N	IDK	Y	N	Y	Y	IDK	Y	14
Akamine et al., 2014 [60]	Y	Y	Y	Y	Y	N	Y	N	Y	Y	Y	IDK	Y	N	IDK	Y	Y	Y	Y	IDK	Y	15
Chen et al., 2014 [61]	Y	Y	IDK	Y	Y	N	Y	N	Y	Y	IDK	IDK	Y	N	IDK	Y	N	IDK	Y	IDK	Y	11
Qiu et al., 2014 [62]	Y	Y	Y	Y	Y	N	Y	N	Y	Y	Y	IDK	Y	N	IDK	Y	N	Y	Y	IDK	Y	14
Joy et al., 2014 [63]	Y	Y	IDK	Y	Y	N	Y	N	Y	Y	Y	IDK	Y	N	IDK	Y	N	Y	Y	IDK	Y	13
Tomaru et al., 2015 [64]	Y	Y	Y	Y	Y	N	Y	N	Y	Y	Y	IDK	Y	N	IDK	Y	N	Y	Y	IDK	Y	14
Akamine et al., 2015 [65]	Y	Y	Y	Y	Y	N	Y	N	Y	Y	Y	IDK	Y	N	IDK	Y	Y	Y	Y	IDK	Y	15
Akamine et al., 2015 [66]	Y	Y	Y	Y	Y	N	Y	N	Y	Y	Y	IDK	Y	N	IDK	Y	N	Y	Y	IDK	Y	14
Yehia et al., 2015 [67]	Y	Y	Y	Y	Y	N	Y	N	Y	Y	Y	IDK	Y	N	IDK	Y	Y	Y	Y	IDK	Y	15
Thomson et al., 2015 [68]	Y	Y	Y	Y	Y	Y	Y	N	Y	Y	Y	IDK	Y	N	IDK	Y	Y	Y	Y	IDK	Y	16
Kullak-Ublick et al., 2016 [69]	Y	Y	Y	Y	Y	N	Y	N	Y	Y	Y	IDK	Y	N	IDK	Y	N	IDK	Y	IDK	Y	13

Helmy et al., 2016 [70]	Y	Y	IDK	Y	Y	N	Y	N	Y	Y	Y	IDK	Y	N	IDK	Y	N	IDK	Y	IDK	Y	12
Luo et al., 2016 [71]	Y	N	Y	Y	Y	Y	Y	N	Y	Y	Y	IDK	Y	N	IDK	Y	N	Y	Y	IDK	Y	14
Bedada et al., 2017 [72]	Y	N	Y	Y	Y	Y	Y	N	Y	Y	Y	IDK	Y	N	IDK	Y	N	Y	Y	IDK	Y	14
Bedada et al., 2017 [73]	Y	Y	Y	Y	Y	Y	Y	N	Y	Y	Y	IDK	Y	N	IDK	Y	N	Y	Y	IDK	Y	15
Cusinato et al., 2019 [74]	Y	Y	Y	Y	Y	Y	Y	N	IDK	Y	Y	IDK	Y	N	IDK	Y	N	Y	Y	IDK	Y	14
Calvo et al., 2019 [75]	Y	IDK	Y	Y	Y	N	Y	N	Y	Y	Y	IDK	Y	N	IDK	Y	N	Y	Y	IDK	Y	13
Cusinato et al., 2019 [76]	Y	Y	IDK	Y	Y	N	Y	N	Y	Y	IDK	IDK	Y	N	IDK	Y	N	Y	Y	IDK	Y	12
Bosilkovska et al., 2019 [77]	Y	Y	Y	Y	Y	N	Y	N	IDK	Y	Y	IDK	Y	N	IDK	Y	N	Y	Y	IDK	Y	13
Simons et al., 1996 [38]	Y	Y	Y	Y	Y	N	Y	N	Y	Y	Y	IDK	Y	N	IDK	Y	Y	Y	Y	IDK	Y	15
Pinto et al., 2020 [79]	Y	IDK	IDK	Y	Y	N	Y	N	Y	Y	Y	IDK	Y	N	IDK	Y	N	Y	Y	IDK	Y	12
Egeland et al., 2020 [80]	Y	Y	Y	Y	Y	N	Y	N	Y	Y	Y	IDK	Y	N	IDK	Y	Y	Y	Y	IDK	Y	15
Zhao et al., 2021 [81]	Y	Y	Y	Y	Y	N	Y	N	Y	Y	Y	IDK	Y	N	IDK	Y	N	Y	Y	IDK	Y	14
Everardo et al., 2021 [82]	Y	Y	Y	Y	Y	Y	Y	N	IDK	Y	Y	IDK	Y	N	IDK	Y	N	Y	Y	IDK	Y	14
Misaka et al., 2022 [83]	Y	Y	Y	Y	Y	N	Y	N	IDK	Y	Y	IDK	Y	N	IDK	Y	N	Y	Y	IDK	Y	13

Rauch et al., 2023 [84]	Y	IDK	Y	Y	Y	N	Y	N	IDK	Y	Y	IDK	IDK	N	IDK	Y	N	Y	Y	IDK	Y	11
Chretien et al., 2023 [85]	Y	Y	Y	Y	Y	N	Y	N	Y	Y	Y	IDK	IDK	N	IDK	Y	N	Y	Y	IDK	Y	13

Y= Yes; N= No; IDK= I don't know

Background
Q1: Was a clear description of the objectives of the study provided?
Q2: Was a valid and comprehensive rationale provided to support the purpose of the study?
Design
Q3: Was the chosen study design appropriately selected and justified?
Q4: Was the dosing (dose, route of administration, dosing interval) of the drug in the study justified for the intended study?
Q5: Were the endpoints of the study appropriate to answer the objectives of the study?
Q6: Were the exclusion criteria of participants included AND appropriate for the intended outcomes of the study?
Q7: Where applicable, were the relevant baseline characteristics of the participants adequately described?
Q8: Were plausible interacting covariates described a priori or in post hoc evaluation?
Q9: Was the description of the used sample analysis methods or citations of prior validation studies provided in the publication or affiliated appendix?
Sampling
Q10: Was the method of data sampling appropriate for the study?
Q11: Was a clear description of the sampling site and the sampling interval (the exact times at which samples are obtained) provided and justifIed?
Q12: Was the number of half-lives elapsed within the sampling period appropriate for the analyzed drug?
Q13: Were sample storage conditions appropriate and described in a manner that could be accurately replicated?
Q14: If applicable, was there a clear description of the pharmacokinetic model, its development, validation and justifcation for use?
Q15: Was the described population pharmacokinetic approach validation method appropriate for the analysis?
Q16: Were the essential pharmacokinetic parameters required to make the results applicable in clinical settings addressed?
Q17: Were the pharmacokinetic equations used to calculate patient pharmacokinetic parameters disclosed or cited within the article?
Applied Statistics
Q18: Were the chosen statistical tests and software to perform the statistical analysis appropriate to achieve the study objectives?
Results
Q19: Were all patients enrolled in the study accounted for?
Q20: In the event of missing data or outliers, was the process for analysis justifed and appropriate?
Q21: Were appropriate summary statistics to describe centrality and variance used to document the pharmacokinetic results?

Supplementary Table S6: Cochrane Collaboration Tool

Cochrane Collaboration Questions								
Reference	Random sequence (Selection bias)	Allocation concealment (Selection bias)	Blinding of participants and researchers (Performed bias)	Blinding of outcome assessment (Detection bias)	Incomplete outcome data (Attrition bias)	Selective reporting (Reporting bias)	Other bias	Total score
Simons et al., 1996 [38]	LR	LR	HR	HR	UR	LR	LR	4
Russell et al., 1998 [1]	LR	LR	LR	LR	LR	LR	UR	5
Robbins et al., 1998 [2]	LR	LR	HR	UR	LR	LR	LR	5
Gupta et al., 2001 [3]	LR	HR	HR	LR	LR	LR	LR	5
Hamman et al., 2001 [4]	HR	HR	HR	UR	LR	LR	LR	3
Drescher et al., 2002 [5]	HR	LR	HR	UR	UR	LR	LR	3
Wang et al., 2002 [6]	HR	HR	HR	HR	LR	LR	LR	3
Banfield et al., 2002 [7]	LR	LR	HR	UR	LR	LR	LR	5
Dresser et al., 2002 [8]	LR	LR	UR	UR	LR	LR	LR	5
Hofmann et al., 2002 [9]	HR	HR	UR	LR	LR	LR	LR	4
Tannergren et al., 2003 [10]	UR	UR	LR	LR	UR	LR	LR	4
Dresser et al., 2003 [11]	HR	LR	HR	HR	LR	LR	LR	4
So-Young Yi et al., 2004 [12]	HR	LR	UR	UR	LR	LR	LR	4
Shon et al., 2005 [13]	LR	LR	LR	LR	UR	LR	LR	6
Boyle et al., 2005 [14]	LR	LR	LR	LR	UR	LR	LR	6
Xie et al., 2005 [15]	UR	LR	UR	UR	LR	LR	LR	4
Yasui-Furukori et al., 2005 [16]	LR	LR	UR	UR	LR	LR	LR	5
Dresser et al., 2005 [17]	LR	LR	HR	HR	LR	LR	LR	5
Rolf et al., 2006 [18]	LR	LR	UR	LR	UR	LR	LR	5

Lemma et al., 2006 [19]	HR	HR	HR	LR	LR	LR	LR	4
M. Shimizu et al., 2006 [20]	LR	LR	HR	UR	LR	LR	LR	5
Uno et al., 2006 [21]	LR	LR	HR	UR	UR	LR	LR	4
Shimizu et al., 2006 [22]	LR	LR	HR	HR	LR	LR	LR	5
Mendoza et al., 2007 [23]	LR	LR	HR	UR	LR	LR	LR	5
Miura et al., 2007 [24]	HR	HR	HR	UR	LR	LR	LR	3
Bailey et al., 2007 [25]	LR	LR	HR	UR	LR	LR	LR	5
Teng et al., 2007 [26]	LR	LR	HR	HR	UR	LR	LR	4
Robertson et al., 2008 [27]	HR	UR	HR	HR	HR	LR	LR	2
Bharathi et al., 2008 [28]	HR	HR	HR	UR	UR	LR	LR	2
Kharasch et al., 2008 [29]	HR	HR	HR	UR	LR	LR	LR	3
Tateishi et al., 2008 [30]	LR	LR	LR	UR	HR	LR	LR	5
Segall et al., 2008 [31]	HR	HR	HR	HR	LR	LR	LR	3
Liu et al., 2009 [32]	LR	HR	UR	HR	LR	LR	LR	4
Kharasch et al., 2009 [33]	HR	HR	UR	UR	LR	LR	LR	3
Kim et al., 2009 [34]	LR	LR	HR	HR	UR	LR	LR	4
Kharasch et al., 2009 [35]	HR	HR	UR	UR	LR	LR	LR	3
Valizadeh et al., 2009 [36]	LR	LR	HR	UR	UR	LR	LR	4
Sakugawa et al., 2009 [37]	LR	LR	HR	HR	UR	LR	LR	4
Nolin et al., 2009 [38]	HR	HR	HR	HR	UR	LR	LR	2
Yamada et al., 2009 [39]	LR	LR	HR	UR	UR	LR	LR	4
Kim et al., 2010 [40]	LR	LR	HR	UR	UR	LR	LR	4
Akamine et al., 2010 [41]	HR	HR	UR	UR	UR	LR	LR	2
Guo et al., 2010 [42]	LR	UR	UR	UR	UR	LR	LR	3
Yamazaki et al., 2010 [43]	LR	LR	HR	HR	LR	LR	LR	5

Penzak et al., 2010 [44]	UR	UR	HR	HR	LR	LR	LR	3
Lappin et al., 2010 [45]	UR	UR	UR	UR	UR	LR	LR	2
Imanaga et al., 2010 [46]	LR	LR	HR	HR	LR	LR	LR	5
Malati et al., 2012 [47]	LR	LR	HR	HR	LR	LR	LR	5
Saruwatari et al., 2012 [48]	LR	LR	HR	HR	LR	LR	LR	5
Akamine et al., 2012 [49]	LR	LR	HR	HR	UR	LR	LR	4
Kharasch et al., 2012 [50]	UR	UR	UR	UR	UR	LR	LR	2
Croft et al., 2012 [51]	UR	UR	UR	UR	UR	LR	LR	2
Kharasch et al., 2013 [52]	UR	UR	UR	UR	UR	LR	LR	2
Won et al., 2013 [53]	LR	LR	UR	UR	UR	LR	LR	4
Zhou et al., 2013 [54]	LR	LR	UR	UR	UR	LR	LR	4
Kusuhara et al., 2013 [55]	LR	LR	UR	UR	UR	LR	LR	4
Ieiri et al., 2013 [56]	UR	UR	HR	HR	UR	LR	LR	2
Muppavarapu et al., 2014 [57]	LR	LR	UR	LR	UR	LR	LR	5
Bedada et al., 2014 [58]	UR	UR	UR	UR	UR	LR	LR	2
Kim et al., 2014 [59]	UR	UR	UR	UR	UR	LR	LR	2
Akamine et al., 2014 [60]	LR	LR	UR	UR	UR	LR	LR	4
Chen et al., 2014 [61]	UR	UR	UR	UR	UR	LR	LR	2
Qiu et al., 2014 [62]	UR	UR	HR	HR	UR	LR	LR	2
Joy et al., 2014 [63]	UR	UR	UR	UR	UR	LR	LR	2
Tomaru et al., 2015 [64]	UR	UR	HR	HR	UR	LR	LR	2
Akamine et al., 2015 [65]	LR	LR	LR	UR	UR	LR	LR	5
Akamine et al., 2015 [66]	LR	LR	HR	HR	UR	LR	LR	4
Yehia et al., 2015 [67]	LR	LR	HR	HR	UR	LR	LR	4

Thomson et al., 2015 [68]	UR	UR	UR	UR	LR	LR	LR	3
Kullak-Ublick et al., 2016 [69]	UR	UR	UR	UR	UR	LR	LR	2
Helmy et al., 2016 [70]	UR	UR	UR	UR	UR	LR	LR	2
Luo et al., 2016 [71]	LR	LR	HR	UR	LR	LR	LR	5
Bedada et al., 2017 [72]	HR	UR	HR	HR	LR	LR	LR	3
Bedada et al., 2017 [73]	HR	UR	HR	HR	LR	LR	LR	3
Cusinato et al., 2019 [74]	HR	UR	HR	HR	LR	LR	LR	3
Calvo et al., 2019 [75]	HR	HR	HR	HR	UR	LR	LR	2
Cusinato et al., 2019 [76]	UR	UR	UR	UR	UR	LR	LR	2
Bosilkovska et al., 2019 [77]	LR	LR	HR	HR	UR	LR	LR	4
Pinto et al., 2020 [79]	UR	UR	UR	UR	UR	LR	LR	2
Egeland et al., 2020 [80]	HR	HR	HR	HR	UR	LR	LR	2
Zhao et al., 2021 [81]	LR	LR	HR	HR	UR	LR	LR	4
Everardo et al., 2021 [82]	LR	LR	HR	HR	LR	LR	LR	5
Misaka et al., 2022 [83]	LR	LR	HR	HR	UR	LR	LR	4
Rauch et al., 2023 [84]	LR	LR	HR	HR	UR	LR	LR	4
Chretien et al., 2023 [85]	LR	LR	HR	HR	LR	LR	LR	5

LR= Low risk; HR= High risk; UR= Unclear risk

REFERENCES

1. Russell T, Stoltz M, Weir S. Pharmacokinetics, pharmacodynamics, and tolerance of single-and multiple-dose fexofenadine hydrochloride in healthy male volunteers. Clinical pharmacology & therapeutics. 1998;64(6):612-621.
2. Robbins DK, Castles MA, Pack DJ, et al. Dose proportionality and comparison of single and multiple dose pharmacokinetics of fexofenadine (MDL 16455) and its enantiomers in healthy male volunteers. Biopharm Drug Dispos. 1998 Oct;19(7):455-63.
3. Gupta S, Banfield C, Kantesaria B, et al. Pharmacokinetic and safety profile of desloratadine and fexofenadine when coadministered with azithromycin: a randomized, placebo-controlled, parallel-group study. Clin Ther. 2001 Mar;23(3):451-66.
4. Hamman MA, Bruce MA, Haehner-Daniels BD, et al. The effect of rifampin administration on the disposition of fexofenadine. Clin Pharmacol Ther. 2001 Mar;69(3):114-21.
5. Drescher S, Schaeffeler E, Hitzl M, et al. MDR1 gene polymorphisms and disposition of the P-glycoprotein substrate fexofenadine. Br J Clin Pharmacol. 2002 May;53(5):526-34.
6. Wang Z, Hamman MA, Huang SM, et al. Effect of St John's wort on the pharmacokinetics of fexofenadine. Clin Pharmacol Ther. 2002 Jun;71(6):414-20.
7. Banfield C, Gupta S, Marino M, et al. Grapefruit juice reduces the oral bioavailability of fexofenadine but not desloratadine. Clin Pharmacokinet. 2002;41(4):311-8.
8. Dresser GK, Bailey DG, Leake BF, et al. Fruit juices inhibit organic anion transporting polypeptide-mediated drug uptake to decrease the oral availability of fexofenadine. Clin Pharmacol Ther. 2002 Jan;71(1):11-20.
9. Hofmann U, Seiler M, Drescher S, et al. Determination of fexofenadine in human plasma and urine by liquid chromatography-mass spectrometry. J Chromatogr B Analyt Technol Biomed Life Sci. 2002 Jan 25;766(2):227-33.
10. Tannergren C, Petri N, Knutson L, et al. Multiple transport mechanisms involved in the intestinal absorption and first-pass extraction of fexofenadine. Clinical Pharmacology & Therapeutics. 2003;74(5):423-436.
11. Dresser GK, Schwarz UI, Wilkinson GR, et al. Coordinate induction of both cytochrome P4503A and MDR1 by St John's wort in healthy subjects. Clin Pharmacol Ther. 2003 Jan;73(1):41-50.
12. Yi SY, Hong KS, Lim HS, et al. A variant 2677A allele of the MDR1 gene affects fexofenadine disposition. Clin Pharmacol Ther. 2004 Nov;76(5):418-27.
13. Shon JH, Yoon YR, Hong WS, et al. Effect of itraconazole on the pharmacokinetics and pharmacodynamics of fexofenadine in relation to the MDR1 genetic polymorphism. Clin Pharmacol Ther. 2005 Aug;78(2):191-201.
14. Boyle J, Ridout F, Meadows R, et al. Suppression of the histamine-induced wheal and flare response by fexofenadine HCl 60 mg twice daily, loratadine 10 mg once daily and placebo in healthy Japanese volunteers. Curr Med Res Opin. 2005 Sep;21(9):1495-503.

15. Xie R, Tan LH, Polasek EC, et al. CYP3A and P-glycoprotein activity induction with St. John's Wort in healthy volunteers from 6 ethnic populations. The Journal of Clinical Pharmacology. 2005;45(3):352-356.
16. Yasui-Furukori N, Uno T, Sugawara K, et al. Different effects of three transporting inhibitors, verapamil, cimetidine, and probenecid, on fexofenadine pharmacokinetics. Clin Pharmacol Ther. 2005 Jan;77(1):17-23.
17. Dresser GK, Kim RB, Bailey DG. Effect of grapefruit juice volume on the reduction of fexofenadine bioavailability: possible role of organic anion transporting polypeptides. Clin Pharmacol Ther. 2005 Mar;77(3):170-7.
18. Rolf P, Bourbeau M, Campbell P, et al. Time-dependent interaction between lopinavir/ritonavir and fexofenadine. The Journal of Clinical Pharmacology. 2006;46(7):758-767.
19. Lemma GL, Wang Z, Hamman MA, et al. The effect of short- and long-term administration of verapamil on the disposition of cytochrome P450 3A and P-glycoprotein substrates. Clin Pharmacol Ther. 2006 Mar;79(3):218-30.
20. Shimizu M, Uno T, Sugawara K, et al. Effects of itraconazole and diltiazem on the pharmacokinetics of fexofenadine, a substrate of P-glycoprotein. Br J Clin Pharmacol. 2006 May;61(5):538-44.
21. Uno T, Shimizu M, Sugawara K, et al. Lack of dose-dependent effects of itraconazole on the pharmacokinetic interaction with fexofenadine. Drug metabolism and disposition. 2006;34(11):1875-1879.
22. Shimizu M, Uno T, Sugawara K, et al. Effects of single and multiple doses of itraconazole on the pharmacokinetics of fexofenadine, a substrate of P-glycoprotein. British journal of clinical pharmacology. 2006;62(3):372-376.
23. Mendoza L, Begany P, Dyrhonova M, et al. Bioequivalence of two fexofenadine formulations in healthy human volunteers after single oral administration. Biomed Pap Med Fac Univ Palacky Olomouc Czech Repub. 2007 Jun;151(1):65-7.
24. Miura M, Uno T, Tateishi T, et al. Pharmacokinetics of fexofenadine enantiomers in healthy subjects. Chirality. 2007 Mar;19(3):223-7.
25. Bailey DG, Dresser GK, Leake BF, et al. Naringin is a major and selective clinical inhibitor of organic anion-transporting polypeptide 1A2 (OATP1A2) in grapefruit juice. Clin Pharmacol Ther. 2007 Apr;81(4):495-502.
26. Teng G-s, Teng L-s, Wu Y, et al. Rapid and Sensitive LC-MS/MS Method for Quantification of Fexofenadine in Human Plasma —— Application to a Bioequivalence Study in Chinese Volunteers. Chemical Research in Chinese Universities. 2007 2007/09/01/;23(5):514-517.
27. Robertson SM, Davey RT, Voell J, et al. Effect of Ginkgo biloba extract on lopinavir, midazolam and fexofenadine pharmacokinetics in healthy subjects. Current medical research and opinion. 2008;24(2):591-599.

28. Bharathi VD, Radharani K, Jagadeesh B, et al. LC–MS–MS assay for simultaneous quantification of fexofenadine and pseudoephedrine in human plasma. Chromatographia. 2008;67:461-466.
29. Kharasch ED, Bedynek PS, Walker A, et al. Mechanism of ritonavir changes in methadone pharmacokinetics and pharmacodynamics: II. Ritonavir effects on CYP3A and P-glycoprotein activities. Clin Pharmacol Ther. 2008 Oct;84(4):506-12.
30. Tateishi T, Miura M, Suzuki T, et al. The different effects of itraconazole on the pharmacokinetics of fexofenadine enantiomers. British journal of clinical pharmacology. 2008;65(5):693-700.
31. Segall N, Grubbe RE, Levy AL, et al. Pharmacokinetics, safety and tolerability of an oral suspension of fexofenadine for children with allergic rhinitis. Allergy Asthma Proc. 2008 Jul-Aug;29(4):380-5.
32. Liu S, Beringer PM, Hidayat L, et al. Probenecid, but not cystic fibrosis, alters the total and renal clearance of fexofenadine. The Journal of Clinical Pharmacology. 2008;48(8):957-965.
33. Kharasch ED, Walker A, Whittington D, et al. Methadone metabolism and clearance are induced by nelfinavir despite inhibition of cytochrome P4503A (CYP3A) activity. Drug and alcohol dependence. 2009;101(3):158-168.
34. Kim K-A, Park P-W, Park J-Y. Short-term effect of quercetin on the pharmacokinetics of fexofenadine, a substrate of P-glycoprotein, in healthy volunteers. European journal of clinical pharmacology. 2009;65:609-614.
35. Kharasch ED, Hoffer C, Whittington D, et al. Methadone pharmacokinetics are independent of cytochrome P4503A (CYP3A) activity and gastrointestinal drug transport: insights from methadone interactions with ritonavir/indinavir. Anesthesiology. 2009 Mar;110(3):660-72.
36. Valizadeh H, Leila B, Jalilian H, et al. Bioequivalence of fexofenadine tablet formulations assessed in healthy Iranian volunteers. Arzneimittelforschung. 2009;59(07):345-349.
37. Sakugawa T, Miura M, Hokama N, et al. Enantioselective disposition of fexofenadine with the P-glycoprotein inhibitor verapamil. British journal of clinical pharmacology. 2009;67(5):535-540.
38. Nolin TD, Frye RF, Le P, et al. ESRD impairs nonrenal clearance of fexofenadine but not midazolam. J Am Soc Nephrol. 2009 Oct;20(10):2269-76.
39. Yamada S, Yasui-Furukori N, Akamine Y, et al. Effects of the P-glycoprotein inducer carbamazepine on fexofenadine pharmacokinetics. Ther Drug Monit. 2009 Dec;31(6):764-8.
40. Kim K-A, Park J-Y, editors. Effect of metronidazole on the pharmacokinetics of fexofenadine in healthsy subjects. DRUG METABOLISM REVIEWS; 2010: TAYLOR & FRANCIS INC 325 CHESTNUT ST, SUITE 800, PHILADELPHIA, PA 19106 USA.

41. Akamine Y, Miura M, Sunagawa S, et al. Influence of drug-transporter polymorphisms on the pharmacokinetics of fexofenadine enantiomers. Xenobiotica. 2010;40(11):782-789.
42. Guo D, Zou J, Zhu Y, et al. Measurement of fexofenadine concentration in micro-sample human plasma by a rapid and sensitive LC-MS/MS employing protein precipitation: application to a clinical pharmacokinetic study. Biomed Chromatogr. 2010 Mar;24(3):335-41.
43. Yamazaki A, Kumagai Y, Yamane N, et al. Microdose study of a P-glycoprotein substrate, fexofenadine, using a non-radioisotope-labelled drug and LC/MS/MS. Journal of clinical pharmacy and therapeutics. 2010;35(2):169-175.
44. Penzak SR, Robertson SM, Hunt JD, et al. Echinacea purpurea significantly induces cytochrome P450 3A activity but does not alter lopinavir-ritonavir exposure in healthy subjects. Pharmacotherapy. 2010 Aug;30(8):797-805.
45. Lappin G, Shishikura Y, Jochemsen R, et al. Pharmacokinetics of fexofenadine: evaluation of a microdose and assessment of absolute oral bioavailability. Eur J Pharm Sci. 2010 May 12;40(2):125-31.
46. Imanaga J, Kotegawa T, Imai H, et al. The effects of the SLCO2B1 c.1457C > T polymorphism and apple juice on the pharmacokinetics of fexofenadine and midazolam in humans. Pharmacogenet Genomics. 2011 Feb;21(2):84-93.
47. Malati CY, Robertson SM, Hunt JD, et al. Influence of Panax ginseng on cytochrome P450 (CYP)3A and P-glycoprotein (P-gp) activity in healthy participants. J Clin Pharmacol. 2012 Jun;52(6):932-9.
48. Saruwatari J, Yasui-Furukori N, Niioka T, et al. Different effects of the selective serotonin reuptake inhibitors fluvoxamine, paroxetine, and sertraline on the pharmacokinetics of fexofenadine in healthy volunteers. J Clin Psychopharmacol. 2012 Apr;32(2):195-9.
49. Akamine Y, Miura M, Yasui-Furukori N, et al. Carbamazepine differentially affects the pharmacokinetics of fexofenadine enantiomers. Br J Clin Pharmacol. 2012 Mar;73(3):478-81.
50. Kharasch ED, Bedynek PS, Hoffer C, et al. Lack of indinavir effects on methadone disposition despite inhibition of hepatic and intestinal cytochrome P4503A (CYP3A). Anesthesiology. 2012 Feb;116(2):432-47.
51. Croft M, Keely B, Morris I, et al. Predicting drug candidate victims of drug-drug interactions, using microdosing. Clin Pharmacokinet. 2012 Apr 1;51(4):237-46.
52. Kharasch ED, Stubbert K. Cytochrome P4503A does not mediate the interaction between methadone and ritonavir-lopinavir. Drug Metab Dispos. 2013 Dec;41(12):2166-74.
53. Won CS, Lan T, VanderMolen KM, et al. A Modified Grapefruit Juice Eliminates Two Compound Classes as Major Mediators of the Grapefruit Juice–Fexofenadine Interaction: An In Vitro–In Vivo “Connect”. The Journal of Clinical Pharmacology. 2013;53(9):982-990.

54. Zhou Q, Ye Z, Ruan Z, et al. Investigation on modulation of human P-gp by multiple doses of Radix Astragali extract granules using fexofenadine as a phenotyping probe. Journal of ethnopharmacology. 2013;146(3):744-749.
55. Kusuhara H, Miura M, Yasui-Furukori N, et al. Effect of coadministration of single and multiple doses of rifampicin on the pharmacokinetics of fexofenadine enantiomers in healthy subjects. Drug Metabolism and Disposition. 2013;41(1):206-213.
56. Ieiri I, Tsunemitsu S, Maeda K, et al. Mechanisms of pharmacokinetic enhancement between ritonavir and saquinavir; micro/small dosing tests using midazolam (CYP3A4), fexofenadine (p-glycoprotein), and pravastatin (OATP1B1) as probe drugs. J Clin Pharmacol. 2013 Jun;53(6):654-61.
57. Muppavarapu R, Guttikar S, Rajappan M, et al. Sensitive LC-MS/MS-ESI method for simultaneous determination of montelukast and fexofenadine in human plasma: application to a bioequivalence study. Biomed Chromatogr. 2014 Aug;28(8):1048-56.
58. Bedada SK, Yakkanti SA, Neerati P. Resveratrol enhances the bioavailability of fexofenadine in healthy human male volunteers: involvement of P-glycoprotein inhibition. J Bioequiv Availab. 2014;6:158-163.
59. Kim MG, Kim Y, Jeon JY, et al. Effect of fermented red ginseng on cytochrome P450 and P-glycoprotein activity in healthy subjects, as evaluated using the cocktail approach. Br J Clin Pharmacol. 2016 Dec;82(6):1580-1590.
60. Akamine Y, Miura M, Komori H, et al. Effects of one-time apple juice ingestion on the pharmacokinetics of fexofenadine enantiomers. European journal of clinical pharmacology. 2014;70:1087-1095.
61. Chen Y-A, Hsu K-Y. Pharmacokinetics of fexofenadine in healthy Taiwanese volunteers. Pakistan Journal of Pharmaceutical Sciences. 2014;27(5):1261-1264.
62. Qiu F, Zeng J, Liu S, et al. Effects of danshen ethanol extract on the pharmacokinetics of fexofenadine in healthy volunteers. Evidence-Based Complementary and Alternative Medicine. 2014;2014.
63. Joy MS, Frye RF, Nolin TD, et al. In vivo alterations in drug metabolism and transport pathways in patients with chronic kidney diseases. Pharmacotherapy: The Journal of Human Pharmacology and Drug Therapy. 2014;34(2):114-122.
64. Tomaru A, Takeda-Morishita M, Maeda K, et al. Effects of Cremophor EL on the absorption of orally administered saquinavir and fexofenadine in healthy subjects. Drug Metab Pharmacokinet. 2015 Jun;30(3):221-6.
65. Akamine Y, Miura M, Yasui-Furukori N, et al. Effects of multiple-dose rifampicin 450 mg on the pharmacokinetics of fexofenadine enantiomers in Japanese volunteers. Journal of clinical pharmacy and therapeutics. 2015;40(1):98-103.
66. Akamine Y, Miura M, Komori H, et al. The change of pharmacokinetics of fexofenadine enantiomers through the single and simultaneous grapefruit juice ingestion. Drug metabolism and pharmacokinetics. 2015;30(5):352-357.

67. Yehia SA, El-Ridi MS, Tadros MI, et al. Phenylalanine-free taste-masked orodispersible tablets of fexofenadine hydrochloride: development, in vitro evaluation and in vivo estimation of the drug pharmacokinetics in healthy human volunteers. Pharmaceutical development and technology. 2015;20(5):528-539.

68. Thomson BKA, Nolin TD, Velenosi TJ, et al. Effect of CKD and Dialysis Modality on Exposure to Drugs Cleared by Nonrenal Mechanisms. American Journal of Kidney Diseases. 2015 2015/04/01/;65(4):574-582.

69. Kullak-Ublick GA, Gubler C, Spanaus K, et al. No major effects of vitamin D3 (1,25 dihydroxyvitamin D3) on absorption and pharmacokinetics of folic acid and fexofenadine in healthy volunteers. Eur J Clin Pharmacol. 2016 Jul;72(7):797-805.

70. Helmy SA, El Bedaiwy HM. HPLC Determination of Fexofenadine in Human Plasma For Therapeutic Drug Monitoring and Pharmacokinetic Studies. Biomed Chromatogr. 2016 Jul;30(7):1059-1064.

71. Luo J, Imai H, Ohyama T, et al. The Pharmacokinetic Exposure to Fexofenadine is Volume-Dependently Reduced in Healthy Subjects Following Oral Administration With Apple Juice. Clin Transl Sci. 2016 Aug;9(4):201-6.

72. Bedada SK, Boga PK, Kotakonda HK. The effect of diosmin on the pharmacokinetics of fexofenadine in healthy human volunteers. Xenobiotica. 2017 Mar;47(3):230-235.

73. Bedada SK, Boga PK. The influence of piperine on the pharmacokinetics of fexofenadine, a P-glycoprotein substrate, in healthy volunteers. Eur J Clin Pharmacol. 2017 Mar;73(3):343-349.

74. Cusinato DAC, Martinez EZ, Cintra MTC, et al. Evaluation of potential herbal-drug interactions of a standardized propolis extract (EPP-AF®) using an in vivo cocktail approach. J Ethnopharmacol. 2019 Dec 5;245:112174.

75. Calvo E, Lee JS, Kim SW, et al. Modulation of Fexofenadine Pharmacokinetics by Osimertinib in Patients With Advanced EGFR-Mutated Non-Small Cell Lung Cancer. J Clin Pharmacol. 2019 Aug;59(8):1099-1109.

76. Cusinato DAC, de Oliveira Filgueira GC, Rocha A, et al. LC-MS/MS analysis of the plasma concentrations of a cocktail of 5 cytochrome P450 and P-glycoprotein probe substrates and their metabolites using subtherapeutic doses. Journal of Pharmaceutical and Biomedical Analysis. 2019;164:430-441.

77. Bosilkovska M, Magliocco G, Desmeules J, et al. Interaction between fexofenadine and CYP phenotyping probe drugs in Geneva cocktail. Journal of personalized medicine. 2019;9(4):45.

78. Helmy SA, El-Bedaiwy HM, El-Masry SM. Applying Biopharmaceutical Classification System criteria to predict the potential effect of Cremophor(®) RH 40 on fexofenadine bioavailability at higher doses. Ther Deliv. 2020 Jul;11(7):447-464.

79. Pinto LSR, Vale GTD, Moreira FL, et al. Direct chiral LC-MS/MS analysis of fexofenadine enantiomers in plasma and urine with application in a maternal-fetal pharmacokinetic study. J Chromatogr B Analyt Technol Biomed Life Sci. 2020 May 15;1145:122094.

80. Egeland EJ, Witczak BJ, Zaré HK, et al. Chronic Inhibition of CYP3A is Temporarily Reduced by Each Hemodialysis Session in Patients With End-Stage Renal Disease. Clin Pharmacol Ther. 2020 Oct;108(4):866-873.
81. Zhao Y, Miao Z, Jiang M, et al. Effects of breviscapine and C3435T MDR1 gene polymorphism on the pharmacokinetics of fexofenadine, a P-glycoprotein substrate, in healthy volunteers. Xenobiotica. 2021 Mar;51(3):366-372.
82. Everardo P-G, Magdalena G-S, Elena G-PM, et al. Bioavailability assessment of fexofenadine and montelukast in a fixed-dose combination tablet versus the components administered simultaneously. Allergologia et Immunopathologia. 2021;49(4):15-25.
83. Misaka S, Ono Y, Taudte RV, et al. Exposure of fexofenadine, but not pseudoephedrine, is markedly decreased by green tea extract in healthy volunteers. Clinical Pharmacology & Therapeutics. 2022;112(3):627-634.
84. Rauch C, Lucio L, De Fer BB, et al. Bioequivalence of 2 Pediatric Formulations of Fexofenadine Hydrochloride Oral Suspension. Clinical Pharmacology in Drug Development. 2023.
85. Chretien ML, Bailey DG, Asher L, et al. Severity of coeliac disease and clinical management study when using a non-metabolised medication: a phase I pharmacokinetic study. BMJ Open. 2023 Feb 24;13(2):e057151.
